# Unified Access to Pyrimidines and Quinazolines Enabled by N–N Cleaving Carbon Atom Insertion

**DOI:** 10.1021/jacs.2c09616

**Published:** 2022-10-14

**Authors:** Ethan E. Hyland, Patrick Q. Kelly, Alexander M. McKillop, Balu D. Dherange, Mark D. Levin

**Affiliations:** Department of Chemistry, University of Chicago, Chicago, Illinois 60637, United States

## Abstract

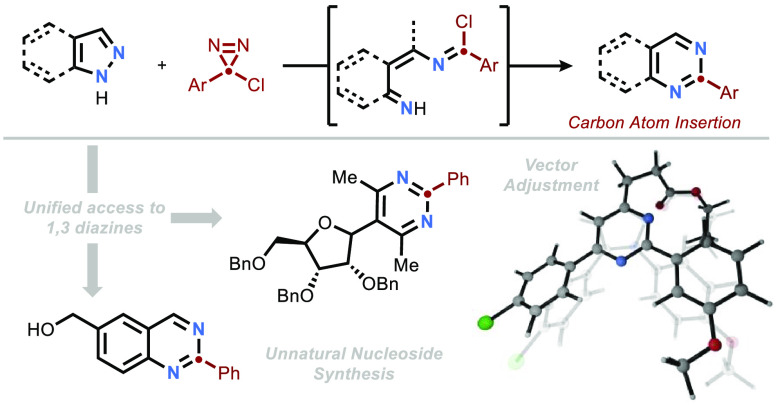

Given the ubiquity of heterocycles in biologically active molecules, transformations with the capacity to modify such molecular skeletons with modularity remain highly desirable. Ring expansions that enable interconversion of privileged heterocyclic motifs are especially interesting in this regard. As such, the known mechanisms for ring expansion and contraction determine the classes of heterocycle amenable to skeletal editing. Herein, we report a reaction that selectively cleaves the N–N bond of pyrazole and indazole cores to afford pyrimidines and quinazolines, respectively. This chlorodiazirine-mediated reaction provides a unified route to a related pair of heterocycles that are otherwise typically prepared by divergent approaches. Mechanistic experiments and DFT calculations support a pathway involving pyrazolium ylide fragmentation followed by cyclization of the ring-opened diazahexatriene intermediate to yield the new diazine core. Beyond enabling access to valuable heteroarenes from easily prepared starting materials, we demonstrate the synthetic utility of skeletal editing in the synthesis of a Rosuvastatin analog as well as in an aryl vector-adjusting direct scaffold hop.

Heterocycles are highly valuable scaffolds for medicinal chemistry, as evidenced by their presence in a majority of biologically active compounds.^1,2^ More specifically, pyrimidines and quinazolines are frequently featured substructures in drug discovery campaigns and remain popular, appearing, for example, in the recently approved kinase inhibitor Belumosudil and the classic HMG-CoA reductase inhibitor Rosuvastatin (Figure 1A).^3−10^

**Figure 1:**
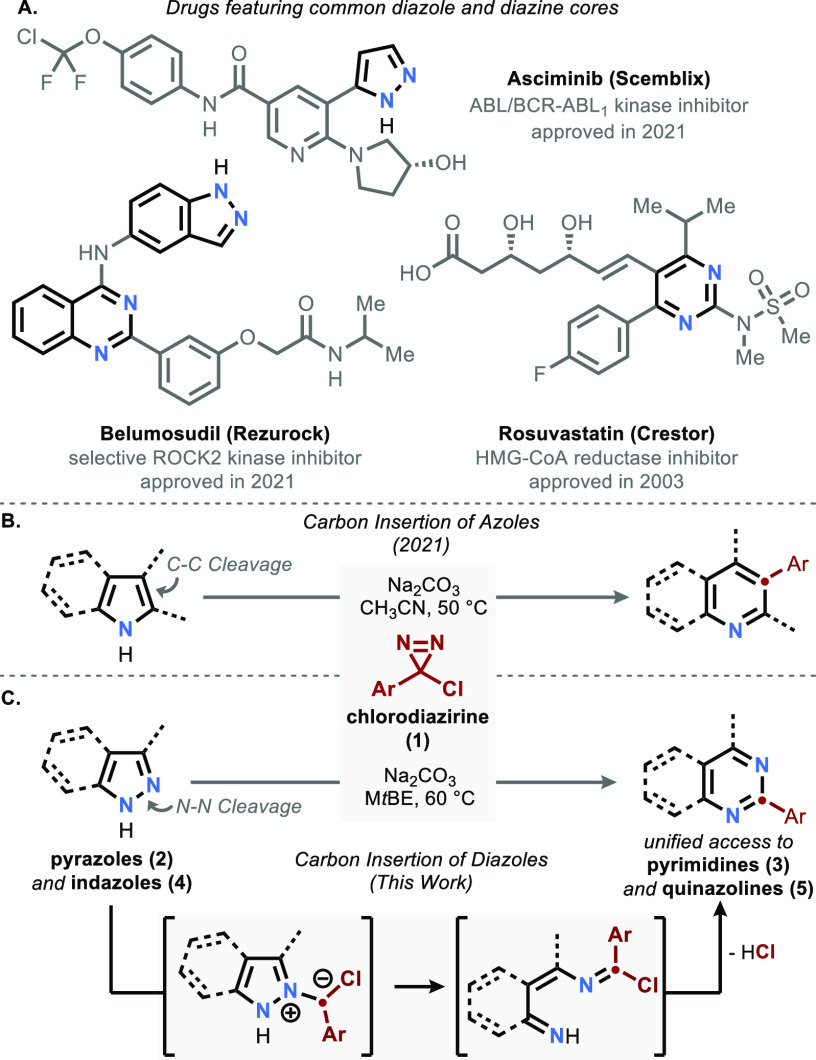
Introduction. (A) Selected examples of azoles and azines in drugs. (B) Chlorodiazirines for ring expansion of pyrroles and indoles. (C) Ring expansion of pyrazoles and indazoles to pyrimidines and quinazolines

Despite the popularity of these targets, they remain challenging to prepare in a modular fashion, with syntheses often limited by substitutional constraints and the use of strong oxidants.^11−13^ To this point, the apparent structural similarity between pyrimidines and quinazolines is deceptive, as it does not translate to similarity in synthesis. These two heterocycles require surprisingly divergent retrosynthetic strategies, with pyrimidines typically prepared from dicarbonyl condensations whereas quinazolines are more commonly prepared from 2-aminophenyl carbonyl compounds.^14−16^ To date, there remain few strategies enabling access to both pyrimidines and quinazolines from analogous precursors,^16−18^ an unfortunate fact given that the enabling retrosynthetic simplicity of such unified methods is a common feature of workhorse transformations in medicinal chemistry.^19,20^ Herein, we report a strategy to access pyrimidines and quinazolines from pyrazoles and indazoles (also frequent scaffolds in medicinal chemistry), respectively, offering an intuitive, common carbon-insertion retrosynthetic disconnection to both motifs.

Our group’s recent work employing chlorodiazirines to promote ring expansion of indoles and pyrroles (Figure 1B) inspired us to continue investigating their reaction with other aromatic heterocycles.^21,22^ These reagents can be easily prepared from commercially available amidine salts in one step and serve as convenient halocarbene precursors.^23−25^ The energetic properties of these compounds have been experimentally determined.^26^

We hypothesized that pyrazoles would demonstrate analogous reactivity to that of pyrroles in the presence of chlorocarbene intermediates, originally envisioning that a [2 + 1] cycloaddition could occur in a similar fashion and provide access to the corresponding pyridazine adducts.^27−29^ To our surprise, the anticipated reaction was not observed, instead affording pyrimidine products through an overall insertion into the N–N bond (Figure 1C). We discuss the mechanism of this serendipitous finding at greater length below; there is, however, a surprising dearth of literature surrounding functionalization of the relatively weak pyrazole N–N bond, especially toward the productive formation of other valuable products.^30−33^

Under similar conditions to those optimized for indoles and pyrroles (60 °C in acetonitrile, excess Na_2_CO_3_), model substrate **2a** afforded the corresponding pyrimidine **3a** in 67% yield alongside formation of the dimeric bis(pyrazolyl)methane side product **6** in 28% yield (Figure 2). Despite this promising start, these same conditions afforded dramatically lower yields for most pyrazoles and indazoles. For example, the unsubstituted pyrazole **2b** afforded the corresponding pyrimidine in a mere 37% yield and quinazoline **5a** was obtained in only 28% from the corresponding indazole **4a**.

**Figure 2:**
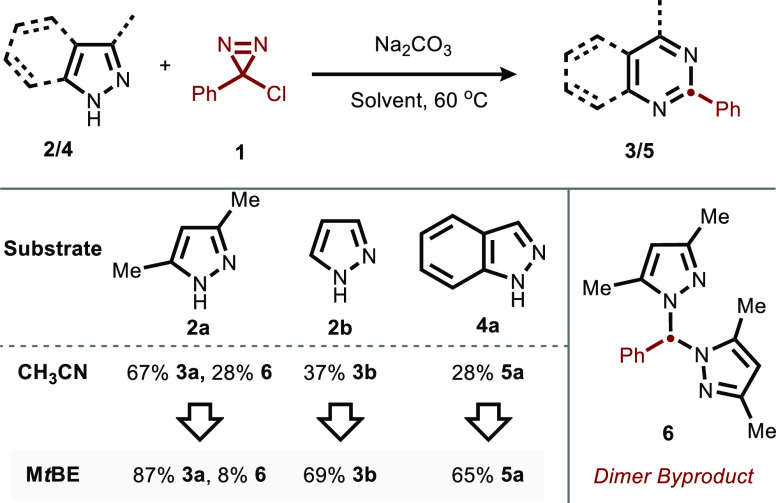
Solvent Effect. Reactions were carried out on 0.1–0.3 mmol scale. Yield by ^1^H-NMR using mesitylene as an internal standard.

We noted, however, that most indazoles were largely insoluble in acetonitrile even at elevated temperatures, prompting us to reexamine the reaction medium. Our prior studies had employed acetonitrile due to the absence of competitive side reactions with the chlorocarbene intermediate (e.g., O–H or C–H insertion).^34^ Ethereal solvents had initially been avoided for this reason, but the observation that indazoles are highly soluble in such solvents encouraged a more thorough survey. We discovered that methyl *tert*-butyl ether (M*t*BE) was a far more general solvent, affording higher yields for both pyrazole and indazole substrates and decreasing the extent of dimer formation. Unlike tetrahydrofuran, which forms substantial amounts of α-functionalized products in the presence of chlorodiazirine, M*t*BE affords only a trace of such side-products, likely a function of steric protection coupled with its marginally stronger α-CH bonds.^35,36^ Under these conditions, lower loadings of diazirine resulted in diminished yields (see Figure S1 for details).

With these conditions in hand, we began to explore the scope of this skeletal transformation, beginning with pyrazoles (Figure 3). A wide variety of *ortho*-, *meta*-, and *para*-substituted aryl chlorodiazirines were found to be suitable coupling partners (**3a**–**3an**). An interesting divergence from our previously reported chemistry was observed: whereas indoles and pyrroles did not react with *p*-methoxyphenyl chlorodiazirine (leading instead to the corresponding benzaldehyde side product^21^), pyrazoles underwent productive reactivity with this substrate. This enhanced reactivity can be attributed to the increased nucleophilicity of pyrazoles relative to pyrroles (Mayr *N* = 8.8 vs 4.6).^37,38^ No major constraints in substitution pattern on the pyrazole were observed (**3a**–**3f**), allowing one to decorate pyrimidines with any desired alkyl substitution pattern, in contrast to many existing pyrimidine syntheses.^39,40^ In addition, esters (**3n**), protected amines (**3l**), alcohols (**3i**), and bromides (**3m**) were all well-tolerated. Many of these functionalities were not compatible with our prior pyrrole chemistry.

**Figure 3:**
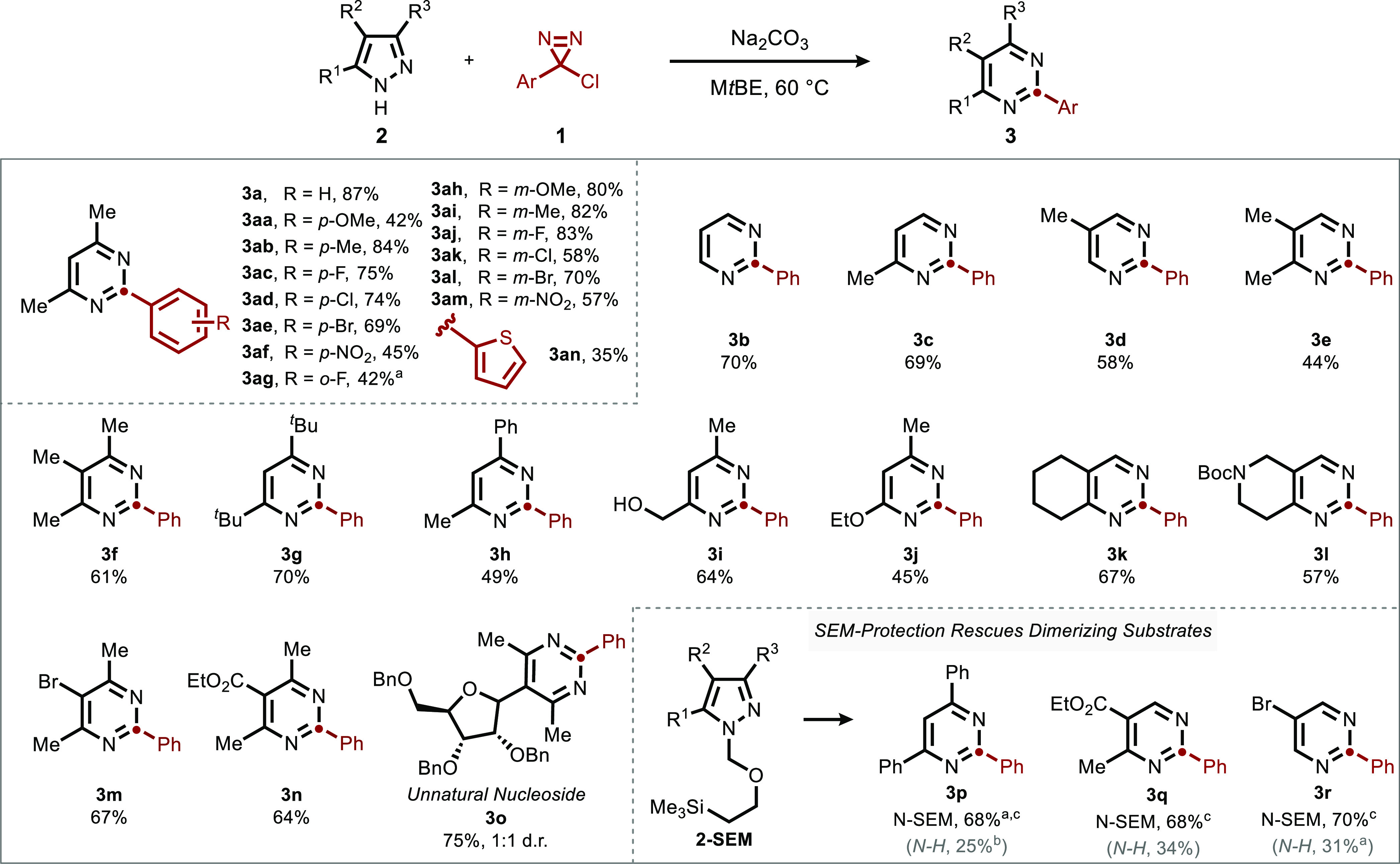
Scope of pyrazole-to-pyrimidine ring expansion. Conditions: **2** (1 equiv), **1** (3 equiv), Na_2_CO_3_ (3 equiv), M*t*BE (0.1M), 60 °C, 12 h. Isolated yields unless otherwise noted, 0.1–0.3 mmol scale. ^a^6 equiv of diazirine were added over 24 h. ^b^Yield by ^1^H-NMR using mesitylene as an internal standard. ^c^TBAF (3 equiv) added during workup.

When the pyrazole is rendered sufficiently electron poor (e.g., **2p** and **2q**), the reaction often instead preferentially forms the bis(pyrazolyl)methane side-product even in M*t*BE solvent. This limitation can be overcome by employing a 2-(Trimethylsilyl)ethoxymethyl (SEM) protecting group, which prevents dimer formation and rescues the pyrimidine product. This group is easily cleaved with a TBAF workup prior to isolation.

Unnatural nucleosides are recognized as a useful pharmacophore, with many high-profile antiviral and oncologic applications employing *C*-bound nucleoside analogs (e.g., Remdesivir, Galidesivir).^41−43^ Inspired by the potential of such unnatural nucleosides and the difficulty of their preparation, we synthesized a C-pyrazole nucleoside (as a mixture of anomers) and subjected it to our standard reaction conditions, offering pyrimidine **3o** in high yield.^44,45^

We next turned to the indazole substrate class (Figure 4). Similarly to pyrazoles, indazoles were tolerant of a wide variety of alkyl substitutions with no substituent requirement at the C3 position. Notably, a free alcohol (**5q**) and a thiophene (**5r**) were all well tolerated. Lastly, indazoles featuring a variety of halogen substituents in various positions were competent reaction partners (**5v**–**5z**), again in contrast to the poor reactivity of haloindoles in our prior report.

**Figure 4:**
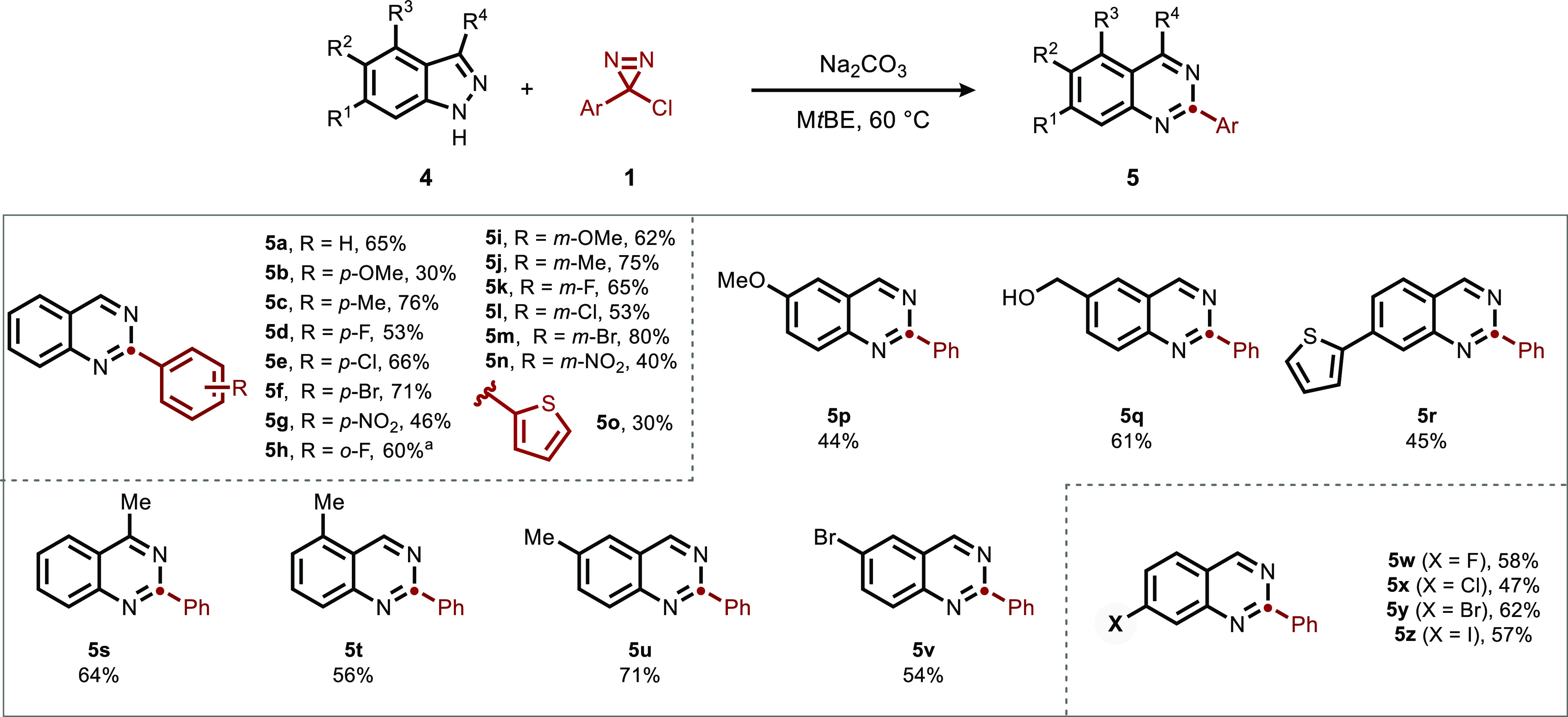
Scope of indazole-to-quinazoline ring expansion. Conditions: **4** (1 equiv), **1** (3 equiv), Na_2_CO_3_ (3 equiv), M*t*BE (0.1M), 60 °C, 12 h. Isolated yields, 0.3 mmol scale. ^a^6 equiv of diazirine were added over 24 h.

For both substrate classes, two major limitations were observed (see Figure S2 for additional examples). First, substrates with low solubility in refluxing M*t*BE were typically poorly reactive—examples include tertiary amine and amide substituents which tended to produce poorly soluble diazoles. Second, inductive withdrawal can deactivate the nucleophilicity of the diazole and preclude productive reactivity, in a manner that is sensitive to substitution pattern. For example, whereas esters were generally tolerated (e.g., **3n** or **3q**), introduction of an ester substituent onto C3 of a pyrazole or C6 of an indazole impeded the reaction in a manner that was not rescued by SEM-protection.

In order to further demonstrate the potential for this method to prepare medicinally relevant compounds, we prepared a C2-aryl analog of the pyrimidine-containing HMG-CoA reductase inhibitor Rosuvastatin (Figure 5A). For this synthesis, the pyrazole precursor **2s-SEM** could be rapidly prepared in high yield over three steps: (i) Claisen condensation of the requisite benzoyl chloride and acetoacetate followed by direct hydrazine condensation to the pyrazole without further purification, (ii) SEM protection, and (iii) reduction of the ester moiety by DIBAL-H. Each step en-route to **2s-SEM** afforded >90% yield, showcasing the simplicity with which highly substituted pyrazoles can be obtained (see Supporting Information (SI) for details). After carbon insertion, the statin was subsequently completed through formation of the phosphonium salt and olefination to install the side chain, affording the corresponding protected form of the Rosuvastatin analog **3t**.

**Figure 5:**
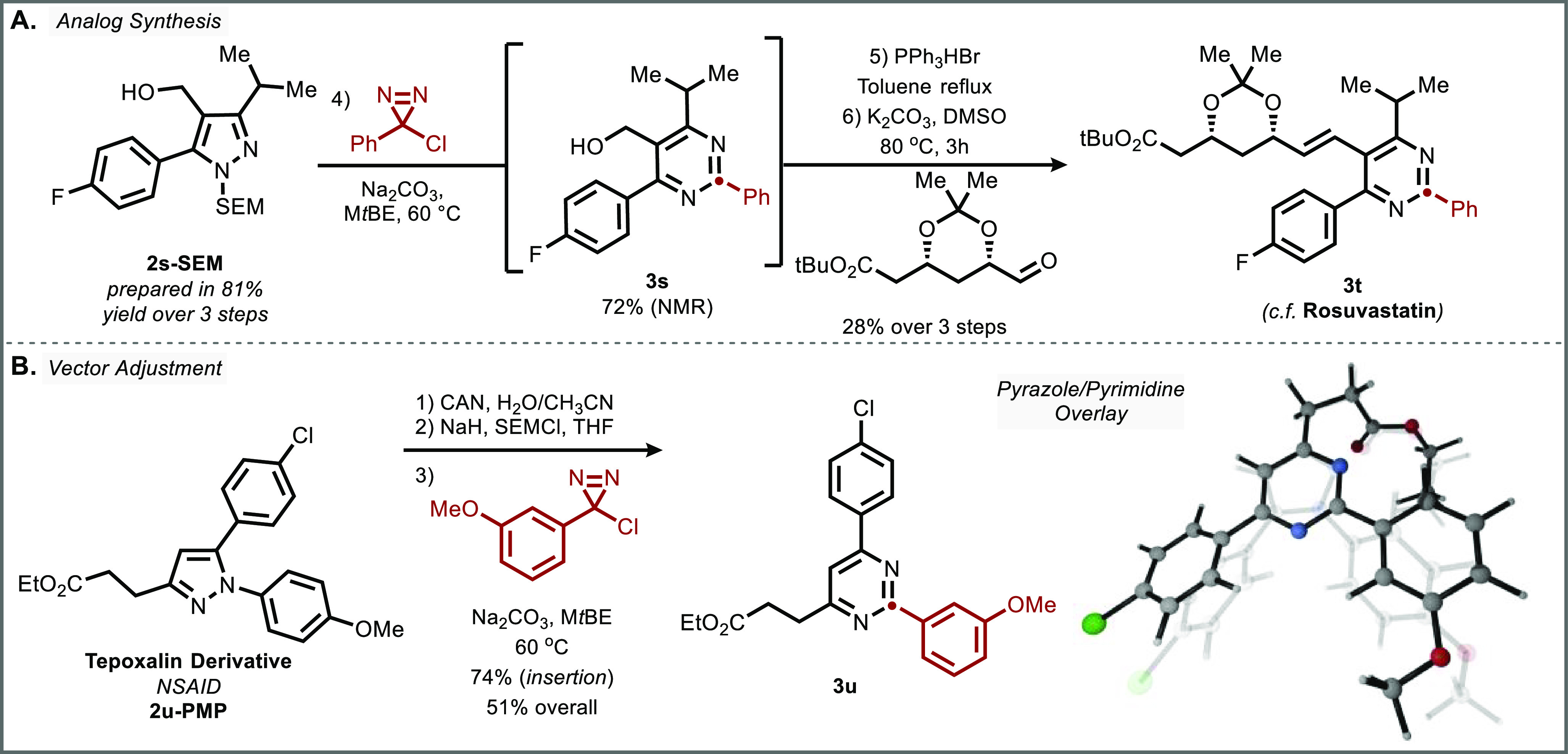
Applications. (A) Synthesis of Rosuvastatin analogue **3t** in 6 steps. (B) Vector adjustment of Tepoxalin ester **2u-PMP** via dearylation and carbon atom insertion. Visualization of this adjustment in overlay of **2u** with pyrimidine **3u**.

We were additionally motivated to showcase the potential of this transformation for direct scaffold hopping (Figure 5B).^46,47^ More specifically, we envisioned that *N*-aryl pyrazoles and 2-aryl pyrimidines would serve as interesting analogs of one another, with a direct interconversion enabled by our method. To this end, initial removal of the *para*-methoxyphenyl (PMP) vector of an ester derivative of the NSAID Tepoxalin (**2u-PMP**) can be achieved using cerium ammonium nitrate in good yield.^48^ Though carbon insertion can be conducted directly on the N–H analog **2u**, prior SEM protection of the dearylated pyrazole afforded a higher yield of isosteric pyrimidine **3u**. As shown in the computed overlay, this skeletal edit enables subtle adjustment of the aryl vectors in the scaffold hop from pyrazole **2u-PMP** to pyrimidine **3u** while maintaining the displayed functionality.

Having demonstrated the synthetic potential of this method, we sought an understanding of its underlying mechanism. Our mechanistic proposal, as supported by Density Functional Theory calculations at the B3LYP-D3BJ/6-311+g(d,p)-PCM(Et_2_O)//B3LYP-D3BJ/6-31g(d)-PCM(Et_2_O) level of theory, is shown in Figure 6A. Dinitrogen extrusion from the chlorodiazirine is proposed to generate free chlorocarbene,^25^ which is attacked by N-2 of the azole to form ylide **INT1**.^49^ The ylide fragments with cleavage of the N–N bond to form diazahexatriene **INT**2, reminiscent of the intermediates formed during ANRORC substitutions.^50−52^ This ring-opened intermediate is primed to undergo ring closure, in what we initially expected to proceed by a 6π-electrocyclic ring-closing followed by rearomatization through loss of chloride.^53^

**Figure 6:**
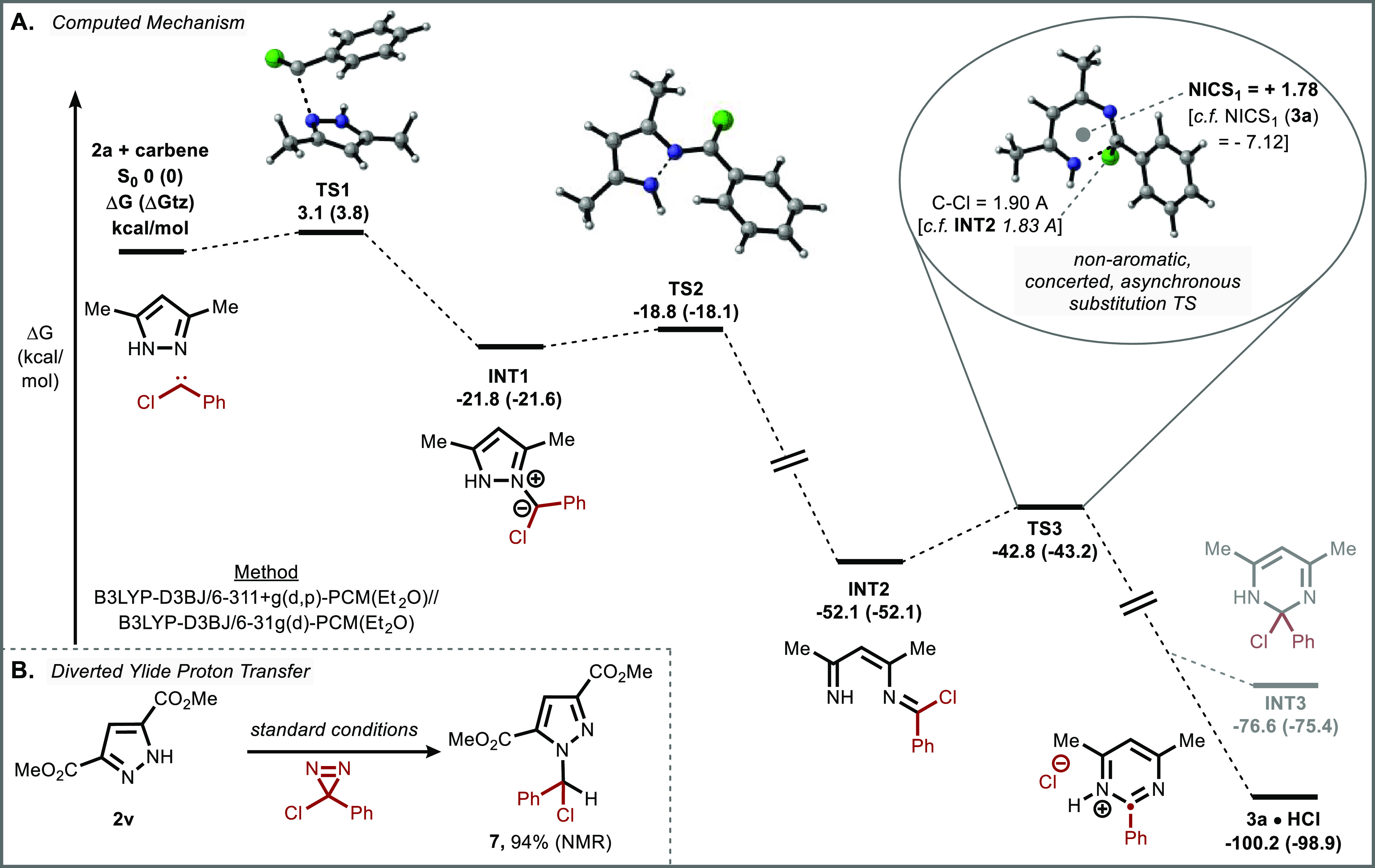
Mechanistic Investigation. (A) Computed fragmentation/ring-closing mechanism for the ring expansion of pyrazoles to pyrimidines. (B) Isolation of a diverted side product. See Supporting Information for details.

Unexpectedly, this last step proceeds directly from **TS3** to the HCl salt of the pyrimidine **3a**, bypassing the chloride-bound dihydropyrimidine intermediate **INT3** altogether, as confirmed by an intrinsic reaction coordinate computation. In fact, **INT3** could only be located as a stationary point when the C–Cl bond length was frozen during optimization; scanning elongation of its C–Cl distance leads to monotonic stabilization with no further transition state prior to aromatization (see SI for details). Indeed, the 1.83 Å C–Cl σ-bond in **INT2** (*cf.* 1.80 Å for acetyl chloride, experimental) further extends to 1.90 Å in **TS3** (*cf.* 1.89 Å for cumyl chloride, computed) and the imidoyl chloride carbon undergoes significant pyramidalization (P = 0.38), consistent with the transition state developing meaningful *sp*^3^ character.^54,55^

This analysis is further supported by measurement of **TS3** aromaticity by its Nucleus Independent Chemical Shift, which revealed a positive NICS_1_ value of 1.78, in contrast to the NICS_1_ value of pyrimidine **3a** (−7.12), suggesting that the transition state lacks aromatic character.^56^ Together, these measurements indicate that **TS3** is best described as a concerted, asynchronous nucleophilic substitution with a dihydropyrimidine-like structure.^57−60^

We suspect that the observed bis(pyrazolyl)methane side product is a result of competitive proton transfer of **INT1** followed by subsequent substitution of the chloride with a second equivalent of starting material. This is supported by the observation that **6** predominates in cases where pyrazoles were more electron poor (i.e., acidic), and by the observation of a fragile *N*-chloroalkyl pyrazole species (**7**) as the sole product in the case of extremely electron-poor pyrazole **2v** (Figure 6B).

In conclusion, we have demonstrated the application of α-chlorodiazirine reagents as competent carbon atom insertion reagents to promote ring-expansion of pyrazoles and indazoles to their respective pyrimidine and quinazoline products through N–N bond cleavage. This method provides rapid access to valuable heteroaromatic cores from easily prepared starting materials in a synthetically intuitive fashion. Interrogation of the mechanism by Density Functional Theory supports an ylide fragmentation–cyclization sequence initiated by trapping of the chlorocarbene at the N-2 terminus of the azole and proceeding via an unusual, concerted ring-closing substitution. This method can be adopted in the synthesis of complex molecules, such as a statin analog and unnatural nucleoside. It is also useful for the purposes of scaffold hopping to enable quick interrogation of a vector adjusted scaffold, as shown with a derivative of Tepoxalin. This novel skeletal editing technique should prove valuable in the interrogation of heterocyclic structure–activity relationships in a wide variety of contexts.

## Supporting Information Available

Experimental procedures, supporting characterization data and spectra, computational methods, and optimized geometries. (PDF)

## References

[ref1] Jampilek J. Heterocycles in Medicinal Chemistry. Molecules 2019, 24 (21), 3839–3843. 10.3390/molecules24213839. PMC6864827 31731387

[ref2] Vitaku E.; Smith D. T.; Njardarson J. T. Analysis of the Structural Diversity, Substitution Patterns, and Frequency of Nitrogen Heterocycles among U.S. FDA Approved Pharmaceuticals. J. Med. Chem. 2014, 57 (24), 10257–10274. 10.1021/jm501100b. 25255204

[ref3] Lauritsen I.; Frendorf P. O.; Capucci S.; Heyde S. A. H.; Blomquist S. D.; Wendel S.; Fischer E. C.; Sekowska A.; Danchin A.; Nørholm M. H. H. Temporal Evolution of Master Regulator Crp Identifies Pyrimidines as Catabolite Modulator Factors. Nat. Commun. 2021, 12 (1), 5880–5893. 10.1038/s41467-021-26098-x. 34620864 PMC8497467

[ref4] Schoepfer J.; Jahnke W.; Berellini G.; Buonamici S.; Cotesta S.; Cowan-Jacob S. W.; Dodd S.; Drueckes P.; Fabbro D.; Gabriel T.; Groell J.-M.; Grotzfeld R. M.; Hassan A. Q.; Henry C.; Iyer V.; Jones D.; Lombardo F.; Loo A.; Manley P. W.; Pellé X.; Rummel G.; Salem B.; Warmuth M.; Wylie A. A.; Zoller T.; Marzinzik A. L.; Furet P. Discovery of Asciminib (ABL001), an Allosteric Inhibitor of the Tyrosine Kinase Activity of BCR-ABL1. J. Med. Chem. 2018, 61 (18), 8120–8135. 10.1021/acs.jmedchem.8b01040. 30137981

[ref5] Watanabe M.; Koike H.; Ishiba T.; Okada T.; Seo S.; Hirai K. Synthesis and Biological Activity of Methanesulfonamide Pyrimidine- and N-Methanesulfonyl Pyrrole-Substituted 3,5-Dihydroxy-6-Heptenoates, a Novel Series of HMG-CoA Reductase Inhibitors. Bioorg. Med. Chem. 1997, 5 (2), 437–444. 10.1016/S0968-0896(96)00248-9. 9061208

[ref6] Li D.; Deng Y.; Achab A.; Bharathan I.; Hopkins B. A.; Yu W.; Zhang H.; Sanyal S.; Pu Q.; Zhou H.; Liu K.; Lim J.; Fradera X.; Lesburg C. A.; Lammens A.; Martinot T. A.; Cohen R. D.; Doty A. C.; Ferguson H.; Nickbarg E. B.; Cheng M.; Spacciapoli P.; Geda P.; Song X.; Smotrov N.; Abeywickrema P.; Andrews C.; Chamberlin C.; Mabrouk O.; Curran P.; Richards M.; Saradjian P.; Miller J. R.; Knemeyer I.; Otte K. M.; Vincent S.; Sciammetta N.; Pasternak A.; Bennett D. J.; Han Y. Carbamate and N-Pyrimidine Mitigate Amide Hydrolysis: Structure-Based Drug Design of Tetrahydroquinoline IDO1 Inhibitors. ACS Med. Chem. Lett. 2021, 12 (3), 389–396. 10.1021/acsmedchemlett.0c00525. 33738066 PMC7957919

[ref7] Alagarsamy V.; Chitra K.; Saravanan G.; Solomon V. R.; Sulthana M. T.; Narendhar B. An Overview of Quinazolines: Pharmacological Significance and Recent Developments. Eur. J. Med. Chem. 2018, 151, 628–685. 10.1016/j.ejmech.2018.03.076. 29656203

[ref8] Smits R. A.; de Esch I. J. P.; Zuiderveld O. P.; Broeker J.; Sansuk K.; Guaita E.; Coruzzi G.; Adami M.; Haaksma E.; Leurs R. Discovery of Quinazolines as Histamine H4 Receptor Inverse Agonists Using a Scaffold Hopping Approach. J. Med. Chem. 2008, 51 (24), 7855–7865. 10.1021/jm800876b. 19053770

[ref9] Kumar S.; Narasimhan B. Therapeutic Potential of Heterocyclic Pyrimidine Scaffolds. Chem. Cent. J. 2018, 12 (1), 38–67. 10.1186/s13065-018-0406-5. 29619583 PMC5884769

[ref10] Drewry D. H.; Annor-Gyamfi J. K.; Wells C. I.; Pickett J. E.; Dederer V.; Preuss F.; Mathea S.; Axtman A. D. Identification of Pyrimidine-Based Lead Compounds for Understudied Kinases Implicated in Driving Neurodegeneration. J. Med. Chem. 2022, 65 (2), 1313–1328. 10.1021/acs.jmedchem.1c00440. 34333981 PMC8802302

[ref11] Quiñones R. E.; Wu Z.-C.; Boger D. L. Reaction Scope of Methyl 1,2,3-Triazine-5-Carboxylate with Amidines and the Impact of C4/C6 Substitution. J. Org. Chem. 2021, 86 (19), 13465–13474. 10.1021/acs.joc.1c01553. 34499494 PMC8488013

[ref12] Deibl N.; Ament K.; Kempe R. A Sustainable Multicomponent Pyrimidine Synthesis. J. Am. Chem. Soc. 2015, 137 (40), 12804–12807. 10.1021/jacs.5b09510. 26414993

[ref13] Yan Y.; Xu Y.; Niu B.; Xie H.; Liu Y. I2-Catalyzed Aerobic Oxidative C(Sp3)–H Amination/C–N Cleavage of Tertiary Amine: Synthesis of Quinazolines and Quinazolinones. J. Org. Chem. 2015, 80 (11), 5581–5587. 10.1021/acs.joc.5b00474. 25942678

[ref14] Hill M. D.; Movassaghi M. New Strategies for the Synthesis of Pyrimidine Derivatives. Chem. – Eur. J. 2008, 14 (23), 6836–6844. 10.1002/chem.200800014. 18384023

[ref15] Kirinde Arachchige P. T.; Yi C. S. Synthesis of Quinazoline and Quinazolinone Derivatives via Ligand-Promoted Ruthenium-Catalyzed Dehydrogenative and Deaminative Coupling Reaction of 2-Aminophenyl Ketones and 2-Aminobenzamides with Amines. Org. Lett. 2019, 21 (9), 3337–3341. 10.1021/acs.orglett.9b01082. 31002524

[ref16] Ahmad O. K.; Hill M. D.; Movassaghi M. Synthesis of Densely Substituted Pyrimidine Derivatives. J. Org. Chem. 2009, 74 (21), 8460–8463. 10.1021/jo9017149. 19810691

[ref17] Movassaghi M.; Hill M. D. Single-Step Synthesis of Pyrimidine Derivatives. J. Am. Chem. Soc. 2006, 128 (44), 14254–14255. 10.1021/ja066405m. 17076488

[ref18] Kim D. Y.; Quang Dao P. D.; Cho C. S. Synthesis of Pyrimidine- and Quinazoline-Fused Benzimidazole-4,7-Diones Using Combinatorial Cyclocondensation and Oxidation. ACS Omega 2018, 3 (12), 17456–17465. 10.1021/acsomega.8b02755. 31458351 PMC6643376

[ref19] Jurczyk J.; Woo J.; Kim S. F.; Dherange B. D.; Sarpong R.; Levin M. D. Single Atom Logic for Skeletal Editing. Nat. Synth. 2022, 1, 352–364. 10.1038/s44160-022-00052-1. 35935106 PMC9355079

[ref20] Brown D. G.; Boström J. Analysis of Past and Present Synthetic Methodologies on Medicinal Chemistry: Where Have All the New Reactions Gone?. J. Med. Chem. 2016, 59 (10), 4443–4458. 10.1021/acs.jmedchem.5b01409. 26571338

[ref21] Dherange B. D.; Kelly P. Q.; Liles J. P.; Sigman M. S.; Levin M. D. Carbon Atom Insertion into Pyrroles and Indoles Promoted by Chlorodiazirines. J. Am. Chem. Soc. 2021, 143 (30), 11337–11344. 10.1021/jacs.1c06287. 34286965 PMC8343525

[ref22] Ma D.; Martin B. S.; Gallagher K. S.; Saito T.; Dai M. One-Carbon Insertion and Polarity Inversion Enabled a Pyrrole Strategy to the Total Syntheses of Pyridine-Containing Lycopodium Alkaloids: Complanadine A and Lycodine. J. Am. Chem. Soc. 2021, 143 (40), 16383–16387. 10.1021/jacs.1c08626. 34570487 PMC9123642

[ref23] Liu M. T. H. The Thermolysis and Photolysis of Diazirines. Chem. Soc. Rev. 1982, 11 (2), 127–140. 10.1039/cs9821100127.

[ref24] Graham W. H. The Halogenation of Amidines. I. Synthesis of 3-Halo- and Other Negatively Substituted Diazirines. J. Am. Chem. Soc. 1965, 87 (19), 4396–4397. 10.1021/ja00947a040.

[ref25] Moss R. A. Diazirines: Carbene Precursors Par Excellence. Acc. Chem. Res. 2006, 39 (4), 267–272. 10.1021/ar050155h. 16618094

[ref26] Musolino S. F.; Pei Z.; Bi L.; DiLabio G. A.; Wulff J. E. Structure–Function Relationships in Aryl Diazirines Reveal Optimal Design Features to Maximize C–H Insertion. Chem. Sci. 2021, 12 (36), 12138–12148. 10.1039/D1SC03631A. 34667579 PMC8457397

[ref27] Moss R. A. Carbenic Reactivity Revisited. Acc. Chem. Res. 1989, 22 (1), 15–21. 10.1021/ar00157a003.

[ref28] Jones R. L.; Rees C. W. Mechanism of Heterocyclic Ring Expansions. Part IV. Reaction of an Imidazole, Pyrazole, and 1,2,4-Triazole with Dichlorocarbene. J. Chem. Soc. C Org. 1969, 18, 2251–2255. 10.1039/j39690002251.

[ref29] Bhatti I. A.; Busby R. E.; Mohamed M. bin; Parrick J.; Shaw C. J. G. Pyrolysis of 1-Substituted Pyrazoles and Chloroform at 550 °C: Formation of α-Carboline from 1-Benzylpyrazoles. J. Chem. Soc. Perkin 1 1997, 24, 3581–3586. 10.1039/A705364I.

[ref30] Guan Z.; Nieger M.; Schmidt A. Organic Synthesis with N-Heterocyclic Carbenes of Indazole: Synthesis of Benzo(Thio)Imidates, Benzo[d][1,3]Thiazines and Quinazoline-4-Thiones. Eur. J. Org. Chem. 2015, 2015 (21), 4710–4719. 10.1002/ejoc.201500331.

[ref31] Chen Q.; Liu X.; Guo F.; Chen Z. An Unexpected Rearrangement of Pyrazolium Halides Based on N–N Bond Cleavage: Synthesis of 1,2-Dihydropyrimidines. Chem. Commun. 2017, 53 (50), 6792–6795. 10.1039/C7CC02525D. 28597892

[ref32] Koronatov A. N.; Rostovskii N. V.; Khlebnikov A. F.; Novikov M. S. Rh(II)-Catalyzed Ring Expansion of Pyrazoles with Diazocarbonyl Compounds as a Method for the Preparation of 1,2-Dihydropyrimidines. J. Org. Chem. 2018, 83 (16), 9210–9219. 10.1021/acs.joc.8b01228. 29916705

[ref33] Zandi S.; Sharafi-Kolkeshvandi M.; Nikpour F. Electrochemically Catalyzed N–N Coupling and Ring Cleavage Reaction of 1H-Pyrazoles. Synthesis 2021, 53 (19), 3591–3596. 10.1055/s-0040-1706050.

[ref34] Rosenberg M. G.; Brinker U. H. Inter- and Innermolecular Reactions of Chloro(Phenyl)Carbene. J. Org. Chem. 2003, 68 (12), 4819–4832. 10.1021/jo026521h. 12790587

[ref35] Tian M.; McCormick R. L.; Luecke J.; de Jong E.; van der Waal J. C.; van Klink G. P. M.; Boot M. D. Anti-Knock Quality of Sugar Derived Levulinic Esters and Cyclic Ethers. Fuel 2017, 202, 414–425. 10.1016/j.fuel.2017.04.027.

[ref36] Agapito F.; Cabral B. J. C.; Simões J. A. M. Carbon–Hydrogen Bond Dissociation Enthalpies in Ethers: A Theoretical Study. J. Mol. Struct. THEOCHEM 2005, 719 (1), 109–114. 10.1016/j.theochem.2005.01.028.

[ref37] Kempf B.; Hampel N.; Ofial A. R.; Mayr H. Structure–Nucleophilicity Relationships for Enamines. Chem. – Eur. J. 2003, 9 (10), 2209–2218. 10.1002/chem.200204666. 12772295

[ref38] Baidya M. Nucleophilicities and Lewis Basicities of Tertiary Amines: A Key to Rationalize Nucleophilic Organocatalysis. PhD Thesis. Der Ludwig-Maximilians-Universität München, Munich, Germany, 2009. pp 9–10. https://core.ac.uk/download/pdf/11031425.pdf.

[ref39] Glinkerman C. M.; Boger D. L. Cycloadditions of 1,2,3-Triazines Bearing C5-Electron Donating Substituents: Robust Pyrimidine Synthesis. Org. Lett. 2015, 17 (16), 4002–4005. 10.1021/acs.orglett.5b01870. 26172042 PMC4546508

[ref40] Jadhav S. D.; Singh A. Oxidative Annulations Involving DMSO and Formamide: K2S2O8Mediated Syntheses of Quinolines and Pyrimidines. Org. Lett. 2017, 19 (20), 5673–5676. 10.1021/acs.orglett.7b02838. 28980820

[ref41] Seley-Radtke K. L.; Yates M. K. The Evolution of Nucleoside Analogue Antivirals: A Review for Chemists and Non-Chemists. Part 1: Early Structural Modifications to the Nucleoside Scaffold. Antiviral Res. 2018, 154, 66–86. 10.1016/j.antiviral.2018.04.004. 29649496 PMC6396324

[ref42] Liang C.; Tian L.; Liu Y.; Hui N.; Qiao G.; Li H.; Shi Z.; Tang Y.; Zhang D.; Xie X.; Zhao X. A Promising Antiviral Candidate Drug for the COVID-19 Pandemic: A Mini-Review of Remdesivir. Eur. J. Med. Chem. 2020, 201, 112527–112539. 10.1016/j.ejmech.2020.112527. 32563812 PMC7834743

[ref43] Julander J. G.; Demarest J. F.; Taylor R.; Gowen B. B.; Walling D. M.; Mathis A.; Babu Y. S. An Update on the Progress of Galidesivir (BCX4430), a Broad-Spectrum Antiviral. Antiviral Res. 2021, 195, 105180–105186. 10.1016/j.antiviral.2021.105180. 34551346 PMC8483777

[ref44] Roy B.; Depaix A.; Périgaud C.; Peyrottes S. Recent Trends in Nucleotide Synthesis. Chem. Rev. 2016, 116 (14), 7854–7897. 10.1021/acs.chemrev.6b00174. 27319940

[ref45] Suzuki K.; Matsumoto T.; Yamauchi T.; Shigeta M. Sc(OTf)3-Catalyzed C-Glycosylation of β-Diketones. A Facile Access to Useful Precursors of Heteroaromatic C-Glycosides. Heterocycles 2005, 66 (1), 153–160. 10.3987/COM-05-S(K)65.

[ref46] Hu Y.; Stumpfe D.; Bajorath J. Recent Advances in Scaffold Hopping. J. Med. Chem. 2017, 60 (4), 1238–1246. 10.1021/acs.jmedchem.6b01437. 28001064

[ref47] Woo J.; Christian A. H.; Burgess S. A.; Jiang Y.; Mansoor U. F.; Levin M. D. Scaffold Hopping by Net Photochemical Carbon Deletion of Azaarenes. Science 2022, 376 (6592), 527–532. 10.1126/science.abo4282. 35482853 PMC9107930

[ref48] Butler R. N.; Hanniffy J. M.; Stephens J. C.; Burke L. A. A Ceric Ammonium Nitrate N-Dearylation of N-p-Anisylazoles Applied to Pyrazole, Triazole, Tetrazole, and Pentazole Rings: Release of Parent Azoles. Generation of Unstable Pentazole, HN5/N5-, in Solution. J. Org. Chem. 2008, 73 (4), 1354–1364. 10.1021/jo702423z. 18198892

[ref49] Jackson J. E.; Soundararajan N.; Platz M. S.; Liu M. T. H. Pyridine Ylide Formation by Capture of Phenylchlorocarbene and Tert-Butylchlorocarbene. Reaction Rates of an Alkylchlorocarbene by Laser Flash Photolysis. J. Am. Chem. Soc. 1988, 110 (16), 5595–5596. 10.1021/ja00224a068.

[ref50] König W. Über Eine Neue, Vom Pyridin Derivierende Klasse von Farbstoffen. J. Für Prakt. Chem. 1904, 69 (1), 105–137. 10.1002/prac.19040690107.

[ref51] Van der Plas H. C. The S_N_(ANRORC) Mechanism: A New Mechanism for Nucleophilic Substitution. Acc. Chem. Res. 1978, 11 (12), 462–468. 10.1021/ar50132a005.

[ref52] Boyle B. T.; Levy J. N.; Lescure L. de.; Paton R. S.; McNally A. 3-Selective Halogenation of Pyridines via Zincke Imine Intermediates. July 11th, 2022. Chem RXiv 10.26434/chemrxiv-2022-88802-v3 (accessed 2022-09-04).

[ref53] Maynard D. F.; Okamura W. H. 6.Pi.-Electrocyclization of 1-Azatrienes to 1,2-Dihydropyridines. J. Org. Chem. 1995, 60 (6), 1763–1771. 10.1021/jo00111a039.

[ref54] Johnson R. D. I. List of Experimental Bond Lengths for Bond Type RCCl. In NIST Computational Chemistry Comparison and Benchmark Database; NIST Standard Reference Database; 2022. https://cccbdb.nist.gov/listbondexp3x.asp?descript=rCCl&mi=14&bi=38.

[ref55] Radhakrishnan T. P.; Agranat I. Measures of Pyramidalization. Struct. Chem. 1991, 2 (2), 107–115. 10.1007/BF00676621.

[ref56] Schleyer P. von R.; Maerker C.; Dransfeld A.; Jiao H.; van Eikema Hommes N. J. R. Nucleus-Independent Chemical Shifts: A Simple and Efficient Aromaticity Probe. J. Am. Chem. Soc. 1996, 118 (26), 6317–6318. 10.1021/ja960582d. 28872872

[ref57] Williams A. Concerted Mechanisms of Acyl Group Transfer Reactions in Solution. Acc. Chem. Res. 1989, 22 (11), 387–392. 10.1021/ar00167a003.

[ref58] Bentley T. W.; Llewellyn G.; McAlister J. A. S_N_2Mechanism for Alcoholysis, Aminolysis, and Hydrolysis of Acetyl Chloride. J. Org. Chem. 1996, 61 (22), 7927–7932. 10.1021/jo9609844. 11667754

[ref59] Fox J. M.; Dmitrenko O.; Liao L.; Bach R. D. Computational Studies of Nucleophilic Substitution at Carbonyl Carbon: The S_N_2Mechanism versus the Tetrahedral Intermediate in Organic Synthesis. J. Org. Chem. 2004, 69 (21), 7317–7328. 10.1021/jo049494z. 15471486

[ref60] Kwan E. E.; Zeng Y.; Besser H. A.; Jacobsen E. N. Concerted Nucleophilic Aromatic Substitutions. Nat. Chem. 2018, 10 (9), 917–923. 10.1038/s41557-018-0079-7. 30013193 PMC6105541

