# Supplementary material for: Unified Access to Pyrimidines and Quinazolines Enabled by N–N Cleaving Carbon Atom Insertion
Source: J Am Chem Soc. 2022 Oct 14;144(42):19258–64. doi: 10.1021/jacs.2c09616 (PMC9619406; doi:10.1021/jacs.2c09616)
Supplement: Supplementary file 1 — ja2c09616_si_001.pdf [file ja2c09616_si_001.pdf]

Supporting Information

Unified Access to Pyrimidines and Quinazolines Enabled by N-N Cleaving Carbon Atom Insertion

Ethan E. Hyland, Patrick Q, Kelly, Alexander M. McKillop, Balu D. Dherange, Mark D. Levin

[1]Department of Chemistry, University of Chicago, Chicago, IL 60637, United States

Experimental Details and Spectra

Table of Contents

I. Material and Methods:... S-2

II. General Procedure for Synthesis of Pyrimidines and Quinazolines:... S-3

III. Synthesis of Starting Materials

IIIA. General Procedure for the Synthesis of Chlorodiazirines:…..…..…………………………..S-15

IIIB. Synthesis of Pyrazoles and Indazoles:...……………………………………..…………...S-18

IIIC. Synthesis of SEM-Protected Pyrazoles:.....………………………………………………..S-20

IV. Synthesis of Rosuvastatin Analogue:…………………………………………………………...S-21

V. Synthesis, Dearylation, and Expansion of Tepoxalin Ester:………………………………………....S-25

VI. Mechanistic Experiments

VA: Arrested Chloromethyl Product from Electron Poor Substrates..………………………….S-28

VB: Effect of Removing N-H bond:………………………………..………………………..S-28

VII. Additional Data and Limitations:...S-30

VIII. Computational Methods.……………………………………………………………………..S-31

IX. References:……………………………………………………………………………...…S-57

X. Spectra:…………………………………………………………………………..…………..…S-60

I. Material and Methods

Unless noted otherwise, all reactions were performed in oven-dried or flame-dried glassware under an atmosphere of dry N_2. CH_3CN, THF, Et_2O, CH_2Cl_2, toluene, and Et_3N were dried by passing these previously degassed solvents through a PPT Solvent Purification System, and all other solvents were dried over molecular sieves (4A) and degassed prior to use or purchased anhydrous and sealed under N_2 (e.g., VWR Dri-solv or equivalent). Reaction temperatures were reported as the temperatures of the bath surrounding the flasks or vials. Sensitive reagents and solvents were transferred under nitrogen into a nitrogen-filled glovebox with standard techniques. Unless otherwise noted, all reagents were used as received. Analytical thin-layer chromatography (TLC) was carried out using 0.2 mm commercial silica gel plates (silica gel 60, F254) and visualized by UV irradiation or staining as indicated.

High resolution mass spectra were recorded on either an Agilent 6224 TOF High Resolution Accurate MS with electrospray ionization or an Agilent 7200B QTOF High Resolution Accurate Mass GCMS using an Agilent HP-5MS column with a temperature gradient of 50 ^oC to 200 ^oC over 15 minutes and electron impact ionization. All mass spectra were processed with an Agilent MassHunter Operating System. Nuclear magnetic resonance spectra (1H-NMR, 13C-NMR and 19F-NMR) were recorded with Bruker spectrometers operating at 400 or 500 MHz for 1H. Chemical shifts are reported in parts per million (ppm, δ), downfield from tetramethylsilane (TMS, δ=0.00 ppm) and are referenced to residual solvent (CDCl3, δ=7.26 ppm (1H) and 77.160 ppm (13C)). Coupling constants were reported in Hertz (Hz). Data for 1H-NMR spectra were reported as follows: chemical shift (ppm, s = singlet, d = doublet, t = triplet, q = quartet, quin = quintet, dd = doublet of doublets, td = triplet of doublets, ddd = doublet of doublet of doublets, m = multiplet, coupling constant (Hz), and integration).

CAUTION! Diazirines are reactive compounds that release gaseous byproducts. Though they are typically less reactive than the isomeric diazos and we have not encountered any stability issues with arylchlorodiazirines to date, lower molecular weight diazirines (e.g., methylchlorodiazirine) have been reported to detonate. Operations should be conducted behind a blast shield and scales should be limited whenever possible. Caution should also be applied when concentrating these compounds. Their energetic properties have previously been experimentally determined.[1]

II. General Procedure for the Synthesis of Pyrimidines (3) and Quinazolines (5) from Pyrazoles (2) and Indazoles (4)

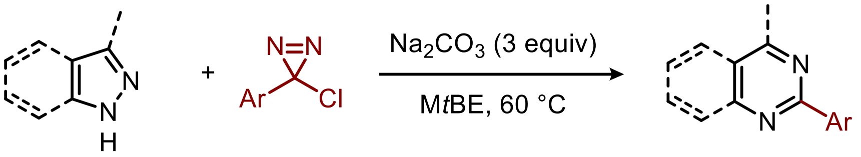

To an oven dried 2-dram screw cap vial equipped with a stir bar and PTFE/white silicone septum cap, pyrazole **2**/indazole **4** (0.3 mmol.), diazirine **1** (0.9 mmol) and Na_2CO_3 (0.9 mmol) were added, followed by a 5-minute nitrogen sparge. Dry M*t*BE (3 mL) was then added, and the vial was sealed with electric tape. The mixture was then allowed to stir at 60 °C for 12 h and then allowed to cool to 25 °C. In cases where the corresponding benzaldehyde byproduct eluted closely with the desired product (see below), $NaBH_4$ in methanol was added to the crude mixture to consume the benzaldehyde byproduct, The reaction mixture was quenched by the addition of water (3 mL), and the two phases were separated. The aqueous layer was extracted with EtOAc (3×5 mL), and the combined organic layers were dried over anhydrous Na_2SO_4, filtered, and concentrated under reduced pressure. The obtained residue was purified by flash column chromatography to afford pyrimidine **3**/quinazoline **5**.

5.4,6-dimethyl-2-phenylpyrimidine (3a):

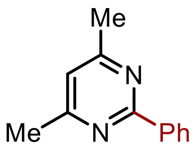

Following general procedure II, product **3a** was isolated in 87% yield as a white solid. R_f =0.4 (silica gel, 10% EtOAc in hexanes): **^{1}H NMR** (400 MHz, $CDCl_3$) δ 8.48 – 8.38 (m, 2H), 7.52 – 7.41 (m, 3H), 6.93 (s, 1H), 2.54 (s, 6H). Spectroscopic data are in agreement with those in the literature. [2]

2-(4-methoxyphenyl)-4,6-dimethylpyrimidine (3aa):

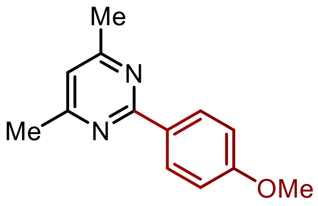

Following general procedure II, modified by the use of Et_3N-neutralized silica during the purification process, product **3aa** was isolated in 42% yield as a white solid. R_f =0.3 (silica gel, 10% EtOAc in hexanes): **^{1}H NMR** (500 MHz, $CDCl_3$) δ 8.42 – 8.34 (m, 2H), 6.98 – 6.95 (m, 2H), 6.86 (s, 1H), 3.87 (s, 3H), 2.51 (s, 6H). Spectroscopic data are in agreement with those in the literature.[3]

4,6-dimethyl-2-(p-tolyl)pyrimidine (3ab):

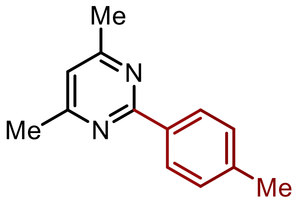

Following general procedure II, product **3ab** was isolated in 84% yield as a white solid. R_f =0.4 (silica gel, 10% EtOAc in hexanes): **^{1}H NMR** (500 MHz, $CDCl_3$) δ 8.34 – 8.28 (m, 2H), 7.24 (s, 2H), 6.88 (s, 1H), 2.51 (s, 6H), 2.39 (s, 3H). Spectroscopic data are in agreement with those in the literature.[3]

2-(4-fluorophenyl)-4,6-dimethylpyrimidine (3ac):

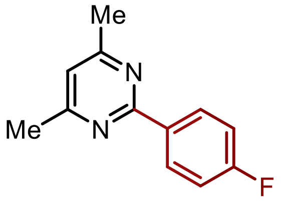

Following general procedure II, product **3ac** was isolated in 75% yield as a white solid. R_f =0.35 (silica gel, 10% EtOAc in hexanes): **^{1}H NMR** (400 MHz, $CDCl_3$) δ 8.47 – 8.41 (m, 2H), 7.16 – 7.10 (m, 2H), 6.92 (s, 1H), 2.53 (s, 6H). Spectroscopic data are in agreement with those in the literature.[3]

2-(4-chlorophenyl)-4,6-dimethylpyrimidine (3ad):

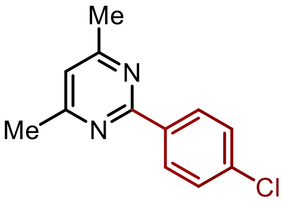

Following general procedure II, modified by addition of $NaBH_4$ in methanol to the crude mixture after heating to consume the benzaldehyde byproduct before workup, product **3ad** was isolated in 74% yield as a white solid. R_f =0.4 (silica gel, 5% EtOAc in hexanes): **^{1}H NMR** (400 MHz, $CDCl_3$) δ 8.43 – 8.35 (m, 2H), 7.47 – 7.39 (m, 2H), 6.92 (s, 1H), 2.52 (s, 6H). **^{13}C NMR** (101 MHz, $CDCl_3$) δ 166.85, 163.13, 136.62, 136.46, 129.59, 128.62, 118.15,24.14. **HRMS** (ESI-TOF) calcd for $C_{12}H_{11}ClN_2^+$ $[M+H]^+$ 219.0689, found 219.0691.

2-(4-bromophenyl)-4,6-dimethylpyrimidine (3ae):

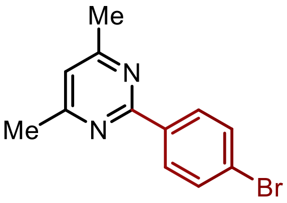

Following general procedure II, modified by addition of $NaBH_4$ in methanol to the crude mixture after heating to consume the benzaldehyde byproduct before workup, product **3ae** was isolated in 69% yield as a white solid. R_f =0.35 (silica gel, 5% EtOAc in hexanes): **^{1}H NMR** (400 MHz, $CDCl_3$) δ 8.37 – 8.29 (m, 2H), 7.63 – 7.51 (m, 2H), 6.94 (s, 1H), 2.53 (s, 6H). Spectroscopic data are in agreement with those in the literature.[4]

4,6-dimethyl-2-(4-nitrophenyl)pyrimidine (3af):

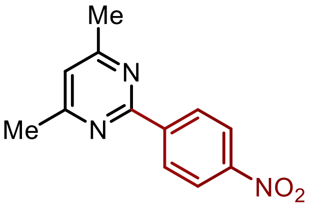

Following general procedure II, product **3af** was isolated in 45% yield as a white solid. R_f =0.25 (silica gel, 20% EtOAc in hexanes): **^{1}H NMR** (500 MHz, $CDCl_3$) δ 8.64 (d, J = 8.9 Hz, 2H), 8.31 (d, J = 8.9 Hz, 2H), 7.02 (s, 1H), 2.57 (s, 6H). Spectroscopic data are in agreement with those in the literature.[4]

2-(2-fluorophenyl)-4,6-dimethylpyrimidine (3ag):

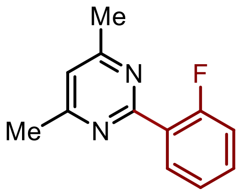

Following general procedure II, modified by the use of 2 x 3 equiv. of diazirine added over 24 h, with addition of 3 equiv. at t = 0 h then 3 equiv. at t = 12 h, product **3ag** was isolated in 42% yield as a white solid. R_f =0.25 (silica gel, 10% EtOAc in hexanes): **^{1}H NMR** (400 MHz, $CDCl_3$) δ 7.93 (tt, J = 7.7, 1.3 Hz, 1H), 7.41 (tdd, J = 6.6, 5.0, 1.5 Hz, 1H), 7.29 – 7.21 (m, 1H), 7.17 (dd, J = 10.9, 8.3 Hz, 1H), 7.00 (s, 1H), 2.57 (s, 6H).**^{13}C NMR** δ 166.93, 163.03 (d, J = 3.9 Hz), 160.86 (d, J = 253.4 Hz), 131.74 (d, J = 2.0 Hz), 131.21 (d, J = 8.5 Hz), 127.32 (d, J = 10.7 Hz), 124.10 (d, J = 3.8 Hz), 118.23, 116.70 (d, J = 22.2 Hz), 24.18.**^{19}F NMR** (377 MHz, $CDCl_3$) δ -115.54. **HRMS** (ESI-TOF) calcd for $C_{12}H_{11}FN_2^+$ $[M]^+$ 202.0906, found 202.0913.

2-(3-methoxyphenyl)-4,6-dimethylpyrimidine (3ah):

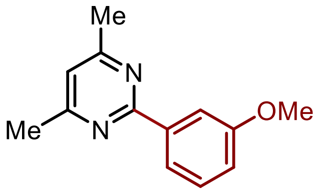

Following general procedure II, product **3ah** was isolated in 80% yield as a white solid. R_f =0.3 (silica gel, 10% EtOAc in hexanes): **^{1}H NMR** (400 MHz, $CDCl_3$) δ 8.07 – 7.98 (m, 2H), 7.38 (t, *J* = 7.9 Hz, 1H), 7.01 (dd, *J* = 8.2, 2.7 Hz, 1H), 6.90 (s, 1H), 3.90 (s, 3H), 2.52 (s, 6H). **^{13}C NMR** (101 MHz, $CDCl_3$) δ 166.73, 163.94, 159.88, 139.64, 129.46, 120.80, 118.07, 116.75, 112.88, 55.39, 24.16. **HRMS** (ESI-TOF) calcd for $C_{13}H_{14}N_2O^+$ $[M+H]^+$ 215.1179, found 215.1198.

4,6-dimethyl-2-(m-tolyl)pyrimidine (3ai):

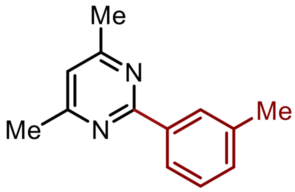

Following general procedure II, product **3ai** was isolated in 82% yield as a white solid. R_f =0.5 (silica gel, 5% EtOAc in hexanes): **^{1}H NMR** (400 MHz, $CDCl_3$) δ 8.26 – 8.18 (m, 2H), 7.36 (t, *J* = 7.6 Hz, 1H), 7.29 – 7.26 (m, 1H), 6.92 (s, 1H), 2.54 (d, *J* = 0.5 Hz, 6H), 2.44 (s, 3H). Spectroscopic data are in agreement with those in the literature.[5]

2-(3-fluorophenyl)-4,6-dimethylpyrimidine (3aj):

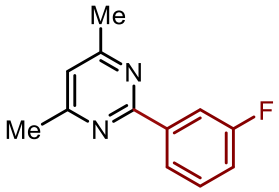

Following general procedure II, modified by addition of $NaBH_4$ in methanol to the crude mixture after heating to consume the benzaldehyde byproduct before workup, product **3aj** was isolated in 83% yield as a white solid. R_f =0.3 (silica gel, 10% EtOAc in hexanes): **^{1}H NMR** (500 MHz, $CDCl_3$) δ 8.23 (dt, *J* = 7.8, 1.3 Hz, 1H), 8.15 (ddd, *J* = 10.4, 2.7, 1.5 Hz, 1H), 7.42 (td, *J* = 8.0, 5.8 Hz, 1H), 7.14 (tdd, *J* = 8.3, 2.7, 1.0 Hz, 1H), 6.93 (s, 1H), 2.53 (s, 6H). **^{13}C NMR** δ 166.91, 163.16 (d, *J* = 244.6 Hz), 162.96 (d, *J* = 3.2 Hz), 140.56 (d, *J* = 7.8 Hz), 129.88 (d, *J* = 8.0 Hz), 123.82 (d, *J* = 2.9 Hz), 118.42, 117.13 (d, *J* = 21.5 Hz), 115.09 (d, *J* = 23.1 Hz), 24.10.**^{19}F NMR** (376 MHz, $CDCl_3$) δ -113.50. **HRMS** (ESI-TOF) calcd for $C_{12}H_{11}FN_2^+$ $[M+H]^+$ 203.098, found 203.980.

2-(3-chlorophenyl)-4,6-dimethylpyrimidine (3ak):

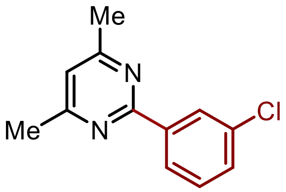

Following general procedure II, modified by addition of $NaBH_4$ in methanol to the crude mixture after heating to consume the benzaldehyde byproduct before workup, product **3ak** was isolated in 58% yield as a white solid. R_f =0.5 (silica gel, 10% EtOAc in hexanes): **^{1}H NMR** (400 MHz, $CDCl_3$) δ 8.45 (td, *J* = 1.8, 0.6 Hz, 1H), 8.33 (dt, *J* = 7.0, 1.7 Hz, 1H), 7.46 – 7.35 (m, 2H), 6.94 (s, 1H), 2.53 (s, 6H). **^{13}C NMR** (101 MHz, $CDCl_3$) δ 166.92, 162.82, 139.99, 134.57, 130.25, 129.68, 128.34, 126.32, 118.45, 24.13. **HRMS** (ESI-TOF) calcd for $C_{12}H_{11}ClN_2^+$ $[M+H]^+$ 219.0689, found 219.0694.

2-(3-bromophenyl)-4,6-dimethylpyrimidine (3al):

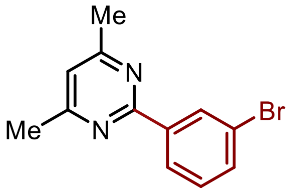

Following general procedure II, modified by addition of $NaBH_4$ in methanol to the crude mixture after heating to consume the benzaldehyde byproduct before workup, product **3al** was isolated in 70% yield as a white solid. R_f =0.4 (silica gel, 10% EtOAc in hexanes): **^{1}H NMR** (400 MHz, $CDCl_3$) δ 8.61 (t, *J* = 1.8 Hz, 1H), 8.38 (dt, *J* = 7.9, 1.3 Hz, 1H), 7.57 (ddd, *J* = 7.9, 2.1, 1.1 Hz, 1H), 7.33 (t, *J* = 7.9 Hz, 1H), 6.94 (s, 1H), 2.53 (s, 6H). **^{13}C NMR** (101 MHz, $CDCl_3$) δ 166.93, 162.70, 140.21, 133.17, 131.26, 129.97, 126.78, 122.75, 118.46, 24.14. **HRMS** (ESI-TOF) calcd for $C_{12}H_{11}BrN_2^+$ [M+H]$^+$ 263.0179 and 265.0158, found 263.0180 and 265.0161.

4,6-dimethyl-2-(3-nitrophenyl)pyrimidine (3am):

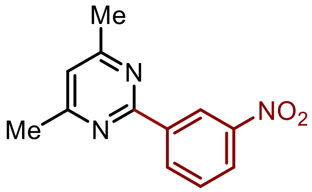

Following general procedure II, product **3am** was isolated in 57% yield as a white solid. R_f =0.25 (silica gel, 10% EtOAc in hexanes): **^{1}H NMR** (400 MHz, $CDCl_3$) δ 9.32 (t, *J* = 2.0 Hz, 1H), 8.80 (dt, *J* = 7.7, 1.4 Hz, 1H), 8.30 (ddd, *J* = 8.2, 2.4, 1.1 Hz, 1H), 7.64 (t, *J* = 8.0 Hz, 1H), 7.01 (s, 1H), 2.57 (s, 6H). **^{13}C NMR** (101 MHz, $CDCl_3$) δ 167.22, 161.81, 148.72, 139.99, 133.99, 129.34, 124.79, 123.34, 118.99, 24.11. **HRMS** (ESI-TOF) calcd for $C_{12}H_{11}N_3O^+$ [M+H]$^+$ 230.0925, found 230.0943.

4,6-dimethyl-2-(thiophen-2-yl)pyrimidine (3an):

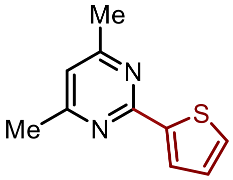

Following general procedure II, product **3an** was isolated in 35% yield as a white solid. R_f =0.4 (silica gel, 10% EtOAc in hexanes): **^{1}H NMR** (400 MHz, $CDCl_3$) δ 7.99 (dd, *J* = 3.7, 1.3 Hz, 1H), 7.43 (dd, *J* = 5.0, 1.3 Hz, 1H), 7.12 (dd, *J* = 5.0, 3.7 Hz, 1H), 6.84 (s, 1H), 2.49 (d, *J* = 0.5 Hz, 6H). Spectroscopic data are in agreement with those in the literature.[3]

2-phenylpyrimidine (3b):

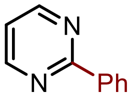

Following general procedure II, product **3b** was isolated in 70% yield as a white solid. R_f =0.3 (silica gel, 10% EtOAc in hexanes): **^{1}H NMR** (400 MHz, $CDCl_3$) δ 8.82 (d, *J* = 4.8 Hz, 2H), 8.49 – 8.41 (m, 2H), 7.55 – 7.47 (m, 3H), 7.19 (t, *J* = 4.8 Hz, 1H). Spectroscopic data are in agreement with those in the literature.[6]

4-methyl-2-phenylpyrimidine (3c):

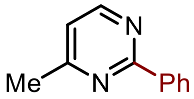

Following general procedure II, product **3c** was isolated in 69% yield as a white solid. R_f =0.3 (silica gel, 10% EtOAc in hexanes): **^{1}H NMR** (400 MHz, $CDCl_3$) δ 8.65 (d, *J* = 5.0 Hz, 1H), 8.47 – 8.40 (m, 2H), 7.48 (dd, *J* = 5.1, 2.0 Hz, 3H), 7.05 (d, *J* = 5.1 Hz, 1H), 2.59 (s, 3H). Spectroscopic data are in agreement with those in the literature.[7]

5-methyl-2-phenylpyrimidine (3d):

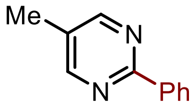

Following general procedure II, product **3d** was isolated in 58% yield as a white solid. R_f =0.3 (silica gel, 10% EtOAc in hexanes): **^{1}H NMR** (400 MHz, $CDCl_3$) δ 8.65 – 8.60 (m, 2H), 8.45 – 8.35 (m, 2H), 7.54 – 7.42 (m, 3H), 2.32 (s, 3H). **^{13}C NMR** (101 MHz, $CDCl_3$) δ 162.48, 157.40,

137.66, 130.35, 128.58, 128.31, 127.87, 15.51. **HRMS** (ESI-TOF) calcd for $C_{11}H_{10}N_2^+$ $[M+H]^+$ 171.0917, found 171.0927.

4,5-dimethyl-2-phenylpyrimidine (3e):

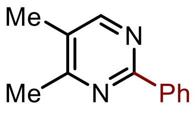

Following general procedure II, product **3e** was isolated in 44% yield as a white solid. R_f =0.4 (silica gel, 15% EtOAc in hexanes): **^{1}H NMR** (400 MHz, $CDCl_3$) δ 8.45 (d, *J* = 0.9 Hz, 1H), 8.44 – 8.38 (m, 2H), 7.52 – 7.40 (m, 3H), 2.54 (s, 3H), 2.27 (s, 3H). **^{13}C NMR** (101 MHz, $CDCl_3$) δ 165.80, 162.40, 156.77, 137.96, 130.10, 128.49, 127.86, 126.70, 22.33, 15.65. **HRMS** (ESI-TOF) calcd for $C_{12}H_{12}N_2^+$ $[M+H]^+$ 185.1034, found 185.1062.

4,5,6-trimethyl-2-phenylpyrimidine (3f):

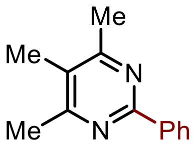

Following general procedure II, product **3f** was isolated in 61% yield as a white solid. R_f =0.4 (silica gel, 5% EtOAc in hexanes): **^{1}H NMR** (400 MHz, $CDCl_3$) δ 8.36 – 8.29 (m, 2H), 7.42 – 7.34 (m, 3H), 2.48 (s, 6H), 2.18 (s, 3H). **^{13}C NMR** (101 MHz, $CDCl_3$) δ 164.60, 161.07, 138.28, 129.83, 128.41, 127.84, 124.84, 22.71, 13.95. **HRMS** (ESI-TOF) calcd for $C_{13}H_{14}N_2^+$ $[M+H]^+$ 199.1191, found 199.1173.

4,6-di-tert-butyl-2-phenylpyrimidine (3g):

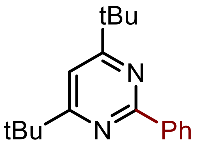

Following general procedure II, product **3g** was isolated in 70% yield as a colorless oil. R_f =0.6 (silica gel, 10% EtOAc in hexanes): ^{1}H NMR (400 MHz, $CDCl_3$) δ 8.53 – 8.46 (m, 2H), 7.37 (d, *J* = 7.6 Hz, 3H), 7.10 (s, 1H), 1.32 (s, 18H). **^{13}C NMR** (101 MHz, $CDCl_3$) δ 177.57, 162.48, 138.94, 130.03, 128.29, 128.29, 109.06, 37.82, 29.65.**[^{1}H, ^{13}C]-HSQC** (400 MHz, 101 MHz, $CDCl_3$) δ (8.50, 128.11), (7.38, 128.46), (7.10, 109.06), (1.32, 29.59). **HRMS** (ESI-TOF) calcd for $C_{18}H_{24}N_2^+$ $[M+]^+$ 268.1939, found 268.1932.

4-methyl-2,6-diphenylpyrimidine (3h):

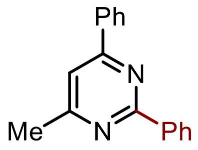

Following general procedure II, product **3h** was isolated in 49% yield as a white solid. R_f =0.5 (silica gel, 10% EtOAc in hexanes): **^{1}H NMR** (400 MHz, $CDCl_3$) δ 8.66 – 8.55 (m, 2H), 8.26 – 8.17 (m, 2H), 7.55 – 7.48 (m, 7H), 2.66 (s, 3H). Spectroscopic data are in agreement with those in the literature.[4]

(6-methyl-2-phenylpyrimidin-4-yl)methanol (3i):

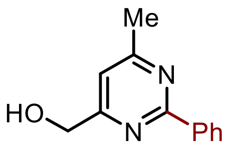

Following general procedure II, product **3i** was isolated in 64% yield as a white solid. R_f =0.25 (silica gel, 30% EtOAc in hexanes): **^{1}H NMR** (400 MHz, $CDCl_3$) δ 8.53 – 8.40 (m, 2H), 7.49 (tt, *J* = 3.9, 2.3 Hz, 3H), 7.02 (s, 1H), 4.77 (d, *J* = 3.8 Hz, 2H), 3.66 (s, 1H), 2.60 (s, 3H). Spectroscopic data are in agreement with those in the literature.[6]

4-ethoxy-6-methyl-2-phenylpyrimidine (3j):

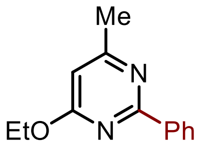

Following general procedure II, product **3j** was isolated in 45% yield as a white solid. R_f =0.3 (silica gel, 10% EtOAc in hexanes): **^{1}H NMR** (400 MHz, $CDCl_3$) δ 8.48 – 8.38 (m, 2H), 7.46 (p, *J* = 3.4 Hz, 3H), 6.45 (s, 1H), 4.54 (q, *J* = 7.1 Hz, 2H), 2.50 (s, 3H), 1.44 (t, *J* = 7.1 Hz, 3H). **^{13}C NMR** (101 MHz, $CDCl_3$) δ 169.77, 167.52, 163.84, 137.89, 130.43, 128.35, 128.23, 104.61, 62.02, 24.08, 14.51. **HRMS** (ESI-TOF) calcd for $C_{13}H_{14}N_2O^+$ $[M+H]^+$ 215.1140, found 215.1173

2-phenyl-5,6,7,8-tetrahydroquinazoline (3k):

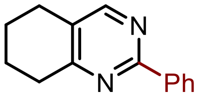

Following general procedure II, product **3k** was isolated in 67% yield as a white solid. R_f =0.4 (silica gel, 15% EtOAc in hexanes): **^{1}H NMR** (400 MHz, $CDCl_3$) δ 8.46 (s, 1H), 8.43 – 8.32 (m, 2H), 7.51 – 7.39 (m, 3H), 2.98 – 2.90 (m, 2H), 2.77 (t, *J* = 6.2 Hz, 2H), 1.99 – 1.81 (m, 4H). Spectroscopic data are in agreement with those in the literature.[2]

tert-butyl 2-phenyl-7,8-dihydropyrido[4,3-d]pyrimidine-6(5H)-carboxylate (3l):

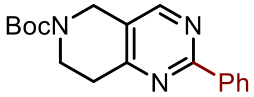

Following general procedure II, product **3l** was isolated in 57% yield as a white solid. R_f =0.3 (silica gel, 20% EtOAc in hexanes): **^{1}H NMR** (400 MHz, $CDCl_3$) δ 8.52 (s, 1H), 8.39 (dd, *J* = 6.7, 3.0 Hz, 2H), 7.47 (dd, *J* = 5.3, 2.0 Hz, 3H), 4.61 (s, 2H), 3.78 (t, *J* = 6.0 Hz, 2H), 3.01 (t, *J* = 6.0 Hz, 2H), 1.51 (s, 9H). **^{13}C NMR** (101 MHz, $CDCl_3$) δ 162.57, 161.81, 153.80, 153.62, 136.51, 129.47, 127.52, 127.01, 123.67, 79.53, 41.91, 38.92, 30.88, 27.40. **HRMS** (ESI-TOF) calcd for $C_{18}H_{21}N_3O_2^+$ $[M+H]^+$ 312.1707, found 312.1717.

5-bromo-4,6-dimethyl-2-phenylpyrimidine (3m):

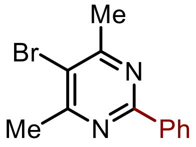

Following general procedure II, product **3m** was isolated in 67% yield as a white solid. R_f=0.5 (silica gel, 10% EtOAc in hexanes): **^{1}H NMR** (500 MHz, $CDCl_3$) δ 8.42 (dt, *J* = 7.2, 3.7 Hz, 2H), 7.57 – 7.39 (m, 3H), 2.70 (d, *J* = 3.4 Hz, 6H). **^{13}C NMR** (126 MHz, $CDCl_3$) δ 165.63, 161.68, 137.05, 130.57, 128.53, 128.16, 120.09, 25.27. **HRMS** (ESI-TOF) calcd for $C_{12}H_{11}BrN_2^+$ $[M+H]^+$ 263.0179 and 265.0158, found 263.0170 and 265.0159.

ethyl 4,6-dimethyl-2-phenylpyrimidine-5-carboxylate (3n):

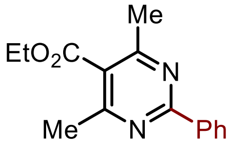

Following general procedure II, product **3n** was isolated in 64% yield as a white solid. R_f =0.2 (silica gel, 20% EtOAc in hexanes): **^{1}H NMR** (400 MHz, $CDCl_3$) δ 8.51 – 8.42 (m, 2H), 7.48 (p, *J* = 3.4 Hz, 3H), 4.45 (q, *J* = 7.1 Hz, 2H), 2.62 (s, 6H), 1.42 (d, *J* = 7.1 Hz, 3H). **^{13}C NMR** (101 MHz, $CDCl_3$) δ 167.91, 164.67, 163.64, 137.23, 130.92, 128.54, 128.53, 124.08, 61.71, 23.16, 14.22. **HRMS** (ESI-TOF) calcd for $C_{15}H_{16}N_2O_2^+$ $[M+H]^+$ 257.1285, found 257.1356.

5-((3S,4R,5R)-3,4-bis(benzyloxy)-5-((benzyloxy)methyl)tetrahydrofuran-2-yl)-4,6-dimethyl-2-phenylpyrimidine (3o):

Following general procedure II, product **3o** was isolated in 75% yield in a 1:1 d.r. as a colorless oil. R_f=0.2 (silica gel, 25% EtOAc in hexanes). The diastereomers were assigned by the diagnostic benzylic peak at 5.36 ppm in which the R stereocenter couples with the syn hydrogen to afford a clean doublet (*J* = 3.4 hz) :

3o – S: **^{1}H NMR** (400 MHz, $CDCl_3$) δ 8.47 – 8.40 (m, 2H), 7.53 – 7.42 (m, 3H), 7.41 – 7.28 (m, 10H), 7.23 – 7.11 (m, 3H), 7.05 (dd, *J* = 7.3, 2.2 Hz, 2H), 5.36 – 5.27 (m, 1H), 4.76 (d, *J* = 12.0 Hz, 1H), 4.63 (d, *J* = 12.0 Hz, 1H), 4.61 – 4.47 (m, 3H), 4.33 (d, *J* = 11.9 Hz, 1H), 4.25 (q, *J* = 3.7 Hz, 1H), 4.16 – 4.05 (m, 2H), 3.67 (dd, *J* = 10.4, 3.5 Hz, 1H), 3.60 (dd, *J* = 10.4, 4.2 Hz, 1H), 2.60 (s, 6H). **^{13}C NMR** (101 MHz, $CDCl_3$) δ 165.99, 162.32, 137.94, 137.89, 137.85, 137.28, 130.27, 128.48, 128.46, 128.44, 128.34, 128.16, 128.16, 127.95, 127.89, 127.78, 127.73, 127.72, 125.16, 83.42, 80.61, 78.13, 76.57, 73.51, 72.85, 72.52, 70.05, 23.60. **HRMS** (ESI-TOF) calcd for $C_{38}H_{38}N_2O_4^+$ $[M+H]^+$ 587.3009, found 587.3009.

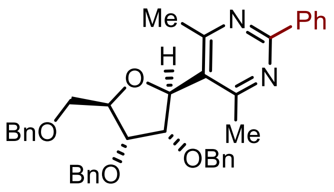

3o – R: **^{1}H NMR** (400 MHz, $CDCl_3$) δ 8.51 – 8.43 (m, 2H), 7.54 – 7.41 (m, 3H), 7.39 – 7.27 (m, 10H), 7.22 – 7.15 (m, 1H), 7.13 (dd, *J* = 8.1, 6.4 Hz, 2H), 6.92 – 6.85 (m, 2H), 5.37 (d, *J* = 3.4 Hz, 1H), 4.62 (dd, *J* = 11.9, 3.1 Hz, 2H), 4.56 – 4.30 (m, 5H), 4.24 (d, *J* = 12.1 Hz, 1H), 4.17 (t, *J* = 3.8 Hz, 1H), 3.83 (dd, *J* = 11.0, 2.3 Hz, 1H), 3.67 (dd, *J* = 11.0, 3.4 Hz, 1H), 2.56 (s, 6H). ^{13}C NMR (101 MHz, $CDCl_3$) δ 165.44, 162.02, 138.18, 138.00, 137.63, 137.17, 130.16, 128.54, 128.46, 128.39, 128.33, 128.10, 128.10, 128.05, 127.86, 127.84, 127.76, 127.69, 125.19, 79.43, 79.36, 78.66, 77.17, 73.54, 73.51, 73.00, 69.27, 23.76. **HRMS** (ESI-TOF) calcd for $C_{38}H_{38}N_2O_4^+$ $[M+H]^+$ 587.3009, found 587.3004.

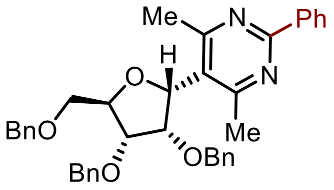

2,4,6-triphenylpyrimidine (3p):

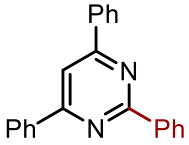

Following general procedure II starting from the N-SEM pyrazole prepared following general procedure IIIC, modified by the use of 2 x 3 equiv. of diazirine added over 24 h, with addition of 3 equiv. at t = 0 h then 3 equiv. at t = 12 h. Additionally, the workup was modified so that 3 equiv. of TBAF were added and allowed to heat at 60 °C for 1 h before isolation. Product **3p** was isolated in 68% yield as a white solid. R_f =0.5 (silica gel, 5% EtOAc in hexanes): **^{1}H NMR** (400 MHz, $CDCl_3$) δ 8.76 – 8.72 (m, 2H), 8.33 – 8.28 (m, 4H), 8.03 (s, 1H), 7.60 – 7.52 (m, 9H). Spectroscopic data are in agreement with those in the literature.[8]

ethyl 4-methyl-2-phenylpyrimidine-5-carboxylate (3q):

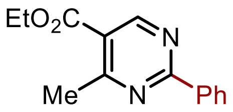

Following general procedure II starting from the N-SEM pyrazole prepared following general procedure IIIC , with a modified workup involving addition of 3 equiv. of TBAF followed by heating at 60 °C for 1 h. Product **3q** was isolated in 68% yield as a white solid. R_f =0.3 (silica gel, 10% EtOAc in hexanes): **^{1}H NMR** (400 MHz, $CDCl_3$) δ 9.22 (s, 1H), 8.56 – 8.47 (m, 2H), 7.57 – 7.46 (m, 3H), 4.43 (q, *J* = 7.1 Hz, 2H), 2.90 (s, 3H), 1.43 (d, *J* = 14.3 Hz, 3H). Spectroscopic data are in agreement with those in the literature.[2]

5-bromo-2-phenylpyrimidine (3r):

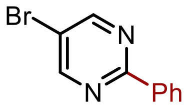

Following general procedure II starting from the N-SEM pyrazole prepared following general procedure IIIC, with a modified workup involving addition of 3 equiv. of TBAF followed by heating at 60 °C for 1 h. Product **3r** was isolated in 70% yield as a white solid. R_f =0.6 (silica gel,

5% EtOAc in hexanes): ^{1}**H NMR** (400 MHz, $CDCl_3$) δ 8.83 (s, 2H), 8.48 – 8.36 (m, 2H), 7.54 – 7.43 (m, 3H). Spectroscopic data are in agreement with those in the literature.[9]

2-phenylquinazoline (5a):

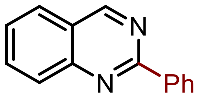

Following general procedure II, product **5a** was isolated in 65% yield as a white solid. R_f =0.4 (silica gel, 10% EtOAc in hexanes): ^{1}**H NMR** (400 MHz, $CDCl_3$) δ 9.48 (d, *J* = 0.8 Hz, 1H), 8.66 – 8.58 (m, 2H), 8.10 (dq, *J* = 8.5, 1.0 Hz, 1H), 7.98 – 7.87 (m, 2H), 7.63 (ddd, *J* = 8.1, 6.9, 1.1 Hz, 1H), 7.58 – 7.48 (m, 4H). Spectroscopic data are in agreement with those in the literature.[10]

2-(4-methoxyphenyl)quinazoline (5b):

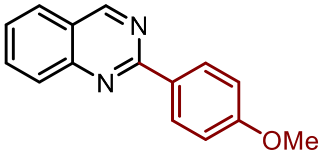

Following general procedure II, modified by the use of Et_3N-neutralized silica during chromatography, product **5b** was isolated in 30% yield as a white solid. R_f =0.2 (silica gel, 10% EtOAc in hexanes): ^{1}**H NMR** (500 MHz, $CDCl_3$) δ 9.42 (s, 1H), 8.61 – 8.55 (m, 2H), 8.04 (d, *J* = 8.4 Hz, 1H), 7.92 – 7.86 (m, 2H), 7.57 (td, *J* = 7.4, 1.1 Hz, 1H), 7.08 – 7.02 (m, 2H), 3.90 (s, 3H). Spectroscopic data are in agreement with those in the literature.[10]

2-(p-tolyl)quinazoline (5c):

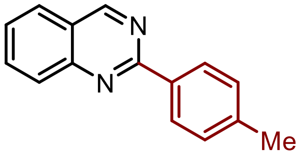

Following general procedure II, product **5c** was isolated in 76% yield as a white solid. R_f =0.4 (silica gel, 10% EtOAc in hexanes): ^{1}**H NMR** (400 MHz, $CDCl_3$) δ 9.45 (d, *J* = 0.9 Hz, 1H), 8.55 – 8.47 (m, 2H), 8.07 (dq, *J* = 8.5, 1.0 Hz, 1H), 7.96 – 7.85 (m, 2H), 7.60 (ddd, *J* = 8.0, 7.0, 1.1 Hz, 1H), 7.38 – 7.31 (m, 2H), 2.45 (s, 3H). Spectroscopic data are in agreement with those in the literature.[11]

2-(4-fluorophenyl)quinazoline (5d):

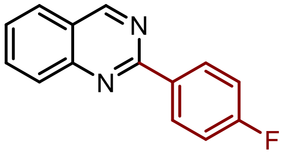

Following general procedure II, product **5d** was isolated in 53% yield as a white solid. R_f =0.4 (silica gel, 15% EtOAc in hexanes): ^{1}**H NMR** (400 MHz, $CDCl_3$) δ 9.45 (d, *J* = 0.9 Hz, 1H), 8.68 – 8.59 (m, 2H), 8.07 (dq, *J* = 8.4, 0.9 Hz, 1H), 7.97 – 7.87 (m, 2H), 7.62 (ddd, *J* = 8.1, 7.0, 1.1 Hz, 1H), 7.24 – 7.17 (m, 2H). Spectroscopic data are in agreement with those in the literature.[11]

2-(4-chlorophenyl)quinazoline (5e):

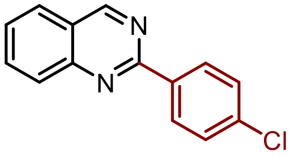

Following general procedure II, modified by addition of $NaBH_4$ in methanol to the crude mixture after heating to consume the benzaldehyde byproduct before workup, product **5e** was isolated in 66% yield as a white solid. R_f =0.4 (silica gel, 10% EtOAc in hexanes): ^{1}**H NMR** (400 MHz, $CDCl_3$) δ 9.46 (d, *J* = 0.8 Hz, 1H), 8.62 – 8.54 (m, 2H), 8.08 (dd, *J* = 8.3, 1.0 Hz, 1H), 7.98 – 7.88 (m, 2H), 7.64 (ddd, *J* = 8.1, 7.0, 1.1 Hz, 1H), 7.54 – 7.47 (m, 2H). Spectroscopic data are in agreement with those in the literature.[10]

2-(4-bromophenyl)quinazoline (5f):

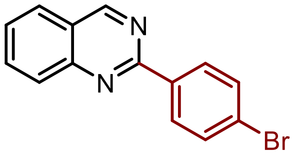

Following general procedure II, product **5f** was isolated in 71% yield as a white solid. R_f =0.3 (silica gel, 10% EtOAc in hexanes): **^{1}H NMR** (400 MHz, $CDCl_3$) δ 9.46 (d, *J* = 0.8 Hz, 1H), 8.55 – 8.47 (m, 2H), 8.08 (dd, *J* = 8.4, 1.0 Hz, 1H), 7.97 – 7.90 (m, 2H), 7.69 – 7.61 (m, 3H). Spectroscopic data are in agreement with those in the literature.[10]

2-(4-nitrophenyl)quinazoline (5g):

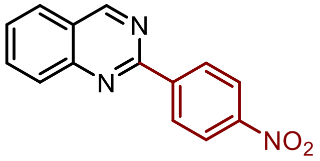

Following general procedure II, product **5g** was isolated in 46% yield as a white solid. R_f =0.2 (silica gel, 25% EtOAc in hexanes): **^{1}H NMR** (400 MHz, $CDCl_3$) δ 9.52 (d, *J* = 0.9 Hz, 1H), 8.85 – 8.80 (m, 2H), 8.40 – 8.37 (m, 2H), 8.17 – 8.12 (m, 1H), 8.02 – 7.95 (m, 2H), 7.71 (ddd, *J* = 8.1, 7.0, 1.1 Hz, 1H). Spectroscopic data are in agreement with those in the literature.[10]

2-(2-fluorophenyl)quinazoline (5h):

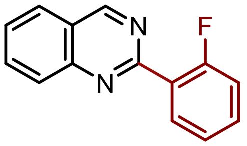

Following general procedure II, modified by the addition of 2 x 3 equiv. of diazirine over 24 h, product **5h** was isolated in 60% yield as a white solid. R_f =0.2 (silica gel, 10% EtOAc in hexanes): **^{1}H NMR** (400 MHz, $CDCl_3$) δ 9.53 (d, *J* = 0.8 Hz, 1H), 8.15 (ddd, *J* = 15.8, 8.0, 1.4 Hz, 2H), 8.02 – 7.91 (m, 2H), 7.68 (ddd, *J* = 8.1, 7.0, 1.1 Hz, 1H), 7.48 (dddd, *J* = 8.2, 7.3, 4.9, 1.9 Hz, 1H), 7.31 (td, *J* = 7.6, 1.2 Hz, 1H), 7.24 (ddd, *J* = 11.1, 8.3, 1.2 Hz, 1H). Spectroscopic data are in agreement with those in the literature.[10]

2-(3-methoxyphenyl)quinazoline (5i):

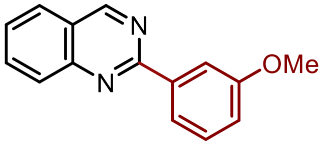

Following general procedure II, product **5i** was isolated in 62% yield as a white solid. R_f =0.2 (silica gel, 10% EtOAc in hexanes): **^{1}H NMR** (400 MHz, $CDCl_3$) δ 9.47 (d, *J* = 0.9 Hz, 1H), 8.27 – 8.16 (m, 2H), 8.10 (dd, *J* = 8.4, 1.1 Hz, 1H), 7.97 – 7.87 (m, 2H), 7.62 (ddd, *J* = 8.0, 6.9, 1.1 Hz, 1H), 7.45 (t, *J* = 7.9 Hz, 1H), 7.07 (ddd, *J* = 8.2, 2.7, 1.0 Hz, 1H), 3.96 (s, 3H). Spectroscopic data are in agreement with those in the literature.[12]

2-(m-tolyl)quinazoline (5j):

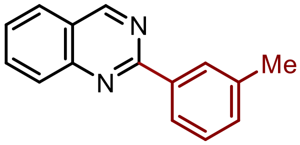

Following general procedure II, product **5j** was isolated in 75% yield as a white solid. R_f =0.4 (silica gel, 10% EtOAc in hexanes): **^{1}H NMR** (400 MHz, $CDCl_3$) δ 9.47 (d, *J* = 0.8 Hz, 1H), 8.46 – 8.38 (m, 2H), 8.10 (dq, *J* = 8.5, 0.9 Hz, 1H), 7.97 – 7.87 (m, 2H), 7.62 (ddd, *J* = 8.1, 7.0, 1.1 Hz, 1H), 7.44 (t, *J* = 7.6 Hz, 1H), 7.33 (dt, *J* = 7.5, 1.8 Hz, 1H), 2.50 (s, 3H). Spectroscopic data are in agreement with those in the literature.[10]

2-(3-fluorophenyl)quinazoline (5k):

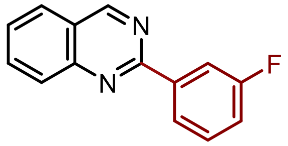

Following general procedure II, modified by addition of $NaBH_4$ in methanol to the crude mixture after heating to consume the benzaldehyde byproduct before workup, product **5k** was isolated in 65% yield as a white solid. R_f =0.3 (silica gel, 10% EtOAc in hexanes): **^{1}H NMR** (400 MHz, $CDCl_3$) δ 9.48 (d, J = 0.9 Hz, 1H), 8.42 (dt, J = 7.9, 1.3 Hz, 1H), 8.34 (ddd, J = 10.4, 2.7, 1.5 Hz, 1H), 8.10 (dq, J = 8.4, 0.9 Hz, 1H), 8.00 – 7.89 (m, 2H), 7.65 (ddd, J = 8.1, 7.0, 1.1 Hz, 1H), 7.50 (td, J = 8.0, 5.8 Hz, 1H), 7.20 (tdd, J = 8.3, 2.7, 1.1 Hz, 1H). Spectroscopic data are in agreement with those in the literature.[13]

2-(3-chlorophenyl)quinazoline (5l):

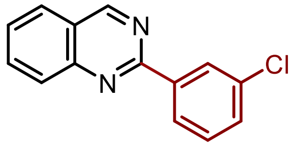

Following general procedure II, product **5l** was isolated in 53% yield as a white solid. R_f =0.3 (silica gel, 15% EtOAc in hexanes): **^{1}H NMR** (500 MHz, $CDCl_3$) δ 9.48 (d, J = 0.9 Hz, 1H), 8.64 (dt, J = 1.8, 1.1 Hz, 1H), 8.52 (ddd, J = 5.9, 2.9, 1.6 Hz, 1H), 8.13 – 8.07 (m, 1H), 7.98 – 7.90 (m, 2H), 7.65 (ddd, J = 8.1, 7.0, 1.1 Hz, 1H), 7.53 – 7.45 (m, 2H). Spectroscopic data are in agreement with those in the literature.[12]

2-(3-bromophenyl)quinazoline (5m):

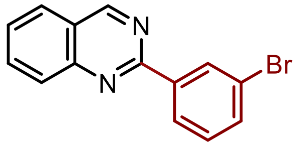

Following general procedure II, product **5m** was isolated in 80% yield as a white solid. R_f =0.3 (silica gel, 10% EtOAc in hexanes): **^{1}H NMR** (400 MHz, $CDCl_3$) δ 9.46 (d, J = 0.8 Hz, 1H), 8.55 – 8.47 (m, 2H), 8.08 (dd, J = 8.4, 1.0 Hz, 1H), 7.97 – 7.90 (m, 2H), 7.69 – 7.61 (m, 3H). Spectroscopic data are in agreement with those in the literature.[14]

2-(3-nitrophenyl)quinazoline (5n):

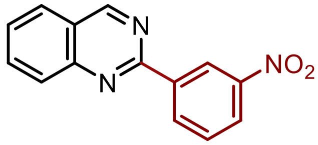

Following general procedure II, product **5n** was isolated in 40% yield as a pale-yellow solid. R_f =0.2 (silica gel, 20% EtOAc in hexanes): **^{1}H NMR** (400 MHz, $CDCl_3$) δ 9.54 – 9.47 (m, 2H), 8.98 (dt, J = 7.8, 1.3 Hz, 1H), 8.35 (ddd, J = 8.2, 2.4, 1.1 Hz, 1H), 8.16 – 8.11 (m, 1H), 7.99 (dt, J = 8.5, 1.3 Hz, 2H), 7.74 – 7.65 (m, 2H). Spectroscopic data are in agreement with those in the literature.[15]

2-(thiophen-2-yl)quinazoline (5o):

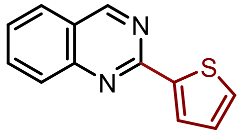

Following general procedure II, product **5o** was isolated in 30% yield as a white solid. R_f =0.25 (silica gel, 10% EtOAc in hexanes): **^{1}H NMR** (400 MHz, $CDCl_3$) δ 9.35 (s, 1H), 8.15 (dd, J = 3.7, 1.3 Hz, 1H), 8.01 (d, J = 8.8 Hz, 1H), 7.91 – 7.83 (m, 2H), 7.60 – 7.49 (m, 2H), 7.20 (d, J = 3.7 Hz, 1H). Spectroscopic data are in agreement with those in the literature.[16]

6-methoxy-2-phenylquinazoline (5p):

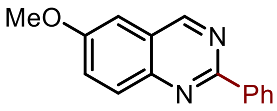

Following general procedure II, product **5p** was isolated in 44% yield as a white solid. R_f =0.3 (silica gel, 30% EtOAc in hexanes): **^{1}H NMR** (400 MHz, $CDCl_3$) δ 9.38 (d, J = 0.8

Hz, 1H), 8.61 – 8.53 (m, 2H), 8.00 (d, *J* = 9.2 Hz, 1H), 7.60 – 7.44 (m, 4H), 7.17 (d, *J* = 2.8 Hz, 1H), 3.98 (s, 3H). Spectroscopic data are in agreement with those in the literature.[17]

(2-phenylquinazolin-6-yl)methanol (5q):

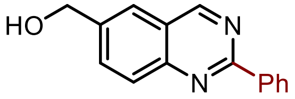

Following general procedure II, product **5q** was isolated in 61% yield as a white solid. R_f =0.2 (silica gel, 50% EtOAc in hexanes): **^{1}H NMR** (400 MHz, $CDCl_3$) δ 9.42 (d, *J* = 0.7 Hz, 1H), 8.64 – 8.56 (m, 2H), 8.09 – 8.03 (m, 1H), 7.87 (d, *J* = 7.9 Hz, 2H), 7.59 – 7.46 (m, 3H), 4.91 (s, 2H), 21.3 (s, 1H). **^{13}C NMR** (101 MHz, $CDCl_3$) δ 161.01, 160.37, 150.34, 140.37, 137.90, 133.29, 130.68, 128.72, 128.69, 128.54, 124.02, 123.37, 64.46. **HRMS** (ESI-TOF) calcd for $C_{15}H_{12}N_2O^+$ [M+H]$^+$ 237.1023, found 237.1012.

2-phenyl-7-(thiophen-2-yl)quinazoline (5r):

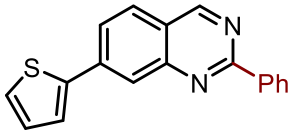

Following general procedure II, product **5r** was isolated in 45% yield as a white solid. R_f =0.25 (silica gel, 10% EtOAc in hexanes): **^{1}H NMR** (400 MHz, $CDCl_3$) δ 9.40 (d, *J* = 0.9 Hz, 1H), 8.62 (dd, *J* = 7.9, 1.9 Hz, 2H), 8.35 (s, 1H), 7.92 (d, *J* = 1.1 Hz, 2H), 7.63 – 7.61 (m, 1H), 7.58 – 7.48 (m, 3H), 6.98 (d, *J* = 3.4 Hz, 1H), 6.59 (dd, *J* = 3.4, 1.8 Hz, 1H). **^{13}C NMR** (101 MHz, $CDCl_3$) δ 161.78, 159.95, 151.36, 142.82, 139.84, 138.02, 130.71, 128.67, 128.60, 128.58, 127.71, 127.23, 125.65, 125.43, 124.02, 122.69. **HRMS** (ESI-TOF) calcd for $C_{18}H_{12}N_2S^+$ [M+H]$^+$ 289.0794, found 289.0806.

4-methyl-2-phenylquinazoline (5s):

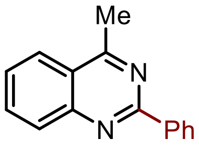

Following general procedure II, product **5s** was isolated in 64% yield as a white solid. R_f =0.4 (silica gel, 5% EtOAc in hexanes): **^{1}H NMR** (400 MHz, $CDCl_3$) δ 8.65 – 8.58 (m, 2H), 8.14 – 8.04 (m, 2H), 7.87 (ddd, *J* = 8.4, 6.9, 1.4 Hz, 1H), 7.63 – 7.54 (m, 1H), 7.54 – 7.44 (m, 3H), 3.03 (s, 3H). Spectroscopic data are in agreement with those in the literature.[18]

5-methyl-2-phenylquinazoline (5t):

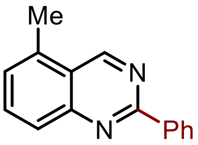

Following general procedure II, product **5t** was isolated in 56% yield as a white solid. R_f =0.4 (silica gel, 10% EtOAc in hexanes): **^{1}H NMR** (400 MHz, $CDCl_3$) δ 9.67 (d, *J* = 0.9 Hz, 1H), 8.65 – 8.58 (m, 2H), 7.97 – 7.90 (m, 1H), 7.78 (dd, *J* = 8.5, 7.0 Hz, 1H), 7.59 – 7.47 (m, 3H), 7.39 (dt, *J* = 7.0, 1.1 Hz, 1H), 2.80 (s, 3H). Spectroscopic data are in agreement with those in the literature.[15]

6-methyl-2-phenylquinazoline (5u):

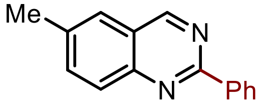

Following general procedure II, product **5u** was isolated in 71% yield as a white solid. R_f =0.4 (silica gel, 15% EtOAc in hexanes): **^{1}H NMR** (400 MHz, $CDCl_3$) δ 9.39 (d, *J* = 0.8 Hz, 1H), 8.63 – 8.55 (m, 2H), 7.99 (d, *J* = 8.6 Hz, 1H), 7.75 (dd, *J* = 8.6, 2.0 Hz, 1H), 7.70 (dt, *J* = 1.9, 0.9 Hz, 1H), 7.60 – 7.45 (m, 3H), 2.58 (s, *J* = 1.0 Hz, 3H). Spectroscopic data are in agreement with those in the literature.[12]

6-bromo-2-phenylquinazoline (5v):

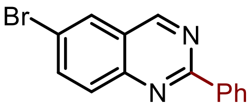

Following general procedure II, product **5v** was isolated in 54% yield as a white solid. R_f =0.4 (silica gel, 5% EtOAc in hexanes): **^{1}H NMR** (400 MHz, $CDCl_3$) δ 9.41 (s, 1H), 8.64 – 8.57 (m, 2H), 8.10 (t, *J* = 1.4 Hz, 1H), 7.97 (d, *J* = 1.3 Hz, 2H), 7.53 (dq, *J* = 4.8, 2.3 Hz, 3H). Spectroscopic data are in agreement with those in the literature.[17]

7-fluoro-2-phenylquinazoline (5w):

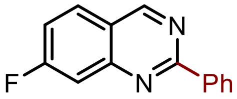

Following general procedure II, product **5w** was isolated in 58% yield as a white solid. R_f =0.3 (silica gel, 10% EtOAc in hexanes): **^{1}H NMR** (400 MHz, $CDCl_3$) δ 9.43 (d, *J* = 0.8 Hz, 1H), 8.66 – 8.56 (m, 2H), 7.95 (dd, *J* = 8.9, 5.9 Hz, 1H), 7.70 (dd, *J* = 9.9, 2.5 Hz, 1H), 7.59 – 7.50 (m, 3H), 7.39 (td, *J* = 8.6, 2.5 Hz, 1H). Spectroscopic data are in agreement with those in the literature.[15]

7-chloro-2-phenylquinazoline (5x):

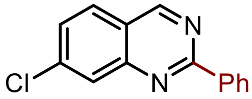

Following general procedure II, product **5x** was isolated in 47% yield as a white solid. R_f =0.3 (silica gel, 10% EtOAc in hexanes): **^{1}H NMR** (400 MHz, $CDCl_3$) δ 9.37 (d, *J* = 0.8 Hz, 1H), 8.56 – 8.51 (m, 2H), 8.03 (dt, *J* = 1.9, 0.7 Hz, 1H), 7.81 (dd, *J* = 8.7, 0.5 Hz, 1H), 7.50 – 7.44 (m, 3H). Spectroscopic data are in agreement with those in the literature.[18]

7-bromo-2-phenylquinazoline (5y):

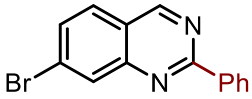

Following general procedure II, product **5y** was isolated in 62% yield as a white solid. R_f =0.25 (silica gel, 5% EtOAc in hexanes): **^{1}H NMR** (400 MHz, $CDCl_3$) δ 9.44 (s, 1H), 8.65 – 8.57 (m, 2H), 8.29 (d, *J* = 1.8 Hz, 1H), 7.80 (d, *J* = 8.6 Hz, 1H), 7.71 (dd, *J* = 8.6, 1.8 Hz, 1H), 7.54 (qd, *J* = 4.6, 1.5 Hz, 3H). **^{13}C NMR** (101 MHz, $CDCl_3$) δ 161.86, 160.34, 151.45, 137.60, 131.19, 131.01, 130.98, 128.93, 128.71, 128.32, 122.19.C-Br ipso peak not detected. **HRMS** (ESI-TOF) calcd for $C_{14}H_9BrN_2^+$ [M+H]$^+$ 285.022 and 287.0002, found 284.9976 and 286.9959.

7-iodo-2-phenylquinazoline (5z):

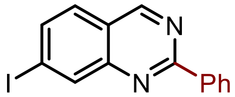

Following general procedure II, product **5z** was isolated in 57% yield as a white solid. R_f =0.3 (silica gel, 10% EtOAc in hexanes): **^{1}H NMR** (400 MHz, $CDCl_3$) δ 9.38 (s, 1H), 8.63 – 8.54 (m, 2H), 8.54 – 8.49 (m, 1H), 7.85 (dd, *J* = 8.5, 1.6 Hz, 1H), 7.58 (d, *J* = 8.5 Hz, 1H), 7.53 (qd, *J* = 4.4, 1.6 Hz, 3H).**^{13}C NMR** (101 MHz, $CDCl_3$) δ 161.55, 160.46, 151.24, 137.83, 137.61, 136.15, 130.99, 128.70, 127.94, 122.47, 101.66. C-I ipso peak not detected**.** **HRMS** (ESI-TOF) calcd for $C_{14}H_{19}IN_2^+$ [M+H]$^+$ 332.9844, found 332.9875.

IIIA. General Procedure for the Synthesis of Chlorodiazirines

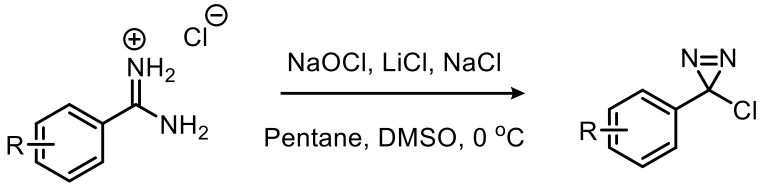

Chlorodiazirines were prepared according to modified literature procedure.[15] A solution of LiCl (5.30 g, 0.125 mol) in DMSO (87 mL) was rapidly stirred within a 1-L Erlenmeyer flask. Meanwhile, NaCl (31.7 g, 0.542 mol) was dissolved in cold 0.56 M NaOCl (296 mL, 0.166 mol) in another 1-L Erlenmeyer flask. The salty chlorine bleach solution was transferred to a 500-mL separatory funnel. Amidine hydrochloride hydrate (23.7 mmol) was added to the 1-L Erlenmeyer reaction flask, and then pentane (50 mL) was poured in. The reaction flask was submerged into an ice bath, and rapid stirring was continued. Addition of the NaOCl/NaCl solution was complete within 20 min. The reaction mixture was stirred for an additional 30 min in the ice bath. The pentane layer was collected using a 1-L separatory funnel. Next, the aqueous DMSO layer was extracted with Et_2O (4 × 25 mL). The combined organic extracts were washed with water (2 × 20 mL) and brine (20 mL). The organic layer was dried over anhydrous $MgSO_4$ and then carefully rotary-evaporated. The residual oil was chromatographed using pentane (or diethyl ether/pentane) as eluant followed by careful rotary evaporation (ca. 30 min) to afford pure diazirine.

3-chloro-3-phenyl-3H-diazirine (1a):

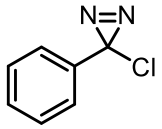

Following the general procedure IIIA, product **1a** was isolated in 64% yield as a colorless liquid. **1a**: R_f =0.5 (silica gel, 5% EtOAc in hexanes); **1H NMR** (400 MHz, CDCl3) δ 7.46 – 7.33 (m, 3H), 7.18 – 7.07 (m, 2H). Spectroscopic data are in agreement with those in the literature.[19]

3-chloro-3-(4-methoxyphenyl)-3H-diazirine (1b):

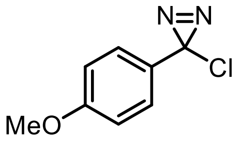

Following the general procedure IIIA, product **1b** was isolated in 60% yield as a yellow liquid. **1b**: R_f =0.5 (silica gel, 5% EtOAc in hexanes); **^{1}H NMR** (400 MHz, $CDCl_3$) δ 7.08 – 6.99 (m, 2H), 6.94 – 6.86 (m, 2H), 3.82 (s, 3H). **^{13}C NMR** (101 MHz, $CDCl_3$) δ 160.50, 127.81, 127.35, 113.96, 55.42, 47.19. **HRMS** (ESI-TOF) calcd for $C_8H_7ClN_2O^+$ $[M]^+$ 182.0247, found 182.0277.

3-chloro-3-(p-tolyl)-3H-diazirine (1c):

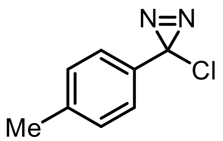

Following the general procedure IIIA, product **1c** was isolated in 58% yield as a colorless liquid. **1c**: R_f =0.6 (silica gel, 5% EtOAc in hexanes); **1H NMR** (400 MHz, CDCl3) δ 7.22 – 7.16 (m, 2H), 7.00 (d, J = 8.3 Hz, 2H), 2.38 (s, 3H). Spectroscopic data are in agreement with those in the literature. [19]

3-chloro-3-(4-fluorophenyl)-3H-diazirine (1d):

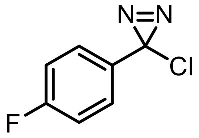

Following the general procedure IIIA, product **1d** was isolated in 62% yield as a colorless liquid. **1d**: R_f = 0.4 (silica gel, 5% EtOAc in hexanes); **1H NMR** (400 MHz, CDCl3) δ 7.14 – 7.04 (m, 4H). Spectroscopic data are in agreement with those in the literature.[19]

3-chloro-3-(4-chlorophenyl)-3H-diazirine (1e):

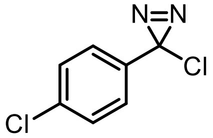

Following the general procedure IIIA, product **1e** was isolated in 64% yield as a colorless liquid. **1e**: R_f =0.6 (silica gel, 5% EtOAc in hexanes); **1H NMR** (400 MHz, CDCl3) δ 7.37 (d, J = 8.0 Hz, 2H), 7.05 (d, J = 8.2 Hz, 2H). Spectroscopic data are in agreement with those in the literature. [19]

3-(4-bromophenyl)-3-chloro-3H-diazirine (1f):

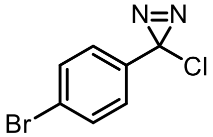

Following the general procedure IIIA, product **1f** was isolated in 55% yield as a colorless liquid. **1f**: R_f =0.5 (silica gel, 5% EtOAc in hexanes); **1H NMR** (400 MHz, CDCl3) δ 7.62 – 7.43 (m, 2H), 7.05 – 6.90 (m, 2H). Spectroscopic data are in agreement with those in the literature.[19]

3-chloro-3-(4-nitrophenyl)-3H-diazirine (1g):

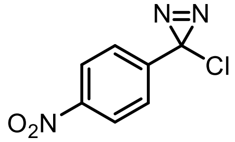

Following the general procedure IIIA, product **1g** was isolated in 60% yield as a pale-yellow solid. **1g**: R_f =0.4 (silica gel, 10% EtOAc in hexanes); **1H NMR** (400 MHz, CDCl3) δ 8.26 (d, J = 8.9 Hz, 2 H), 7.30 (d, J = 9.0 Hz, 2 H). Spectroscopic data are in agreement with those in the literature. [19]

3-chloro-3-(2-fluorophenyl)-3H-diazirine (1h):

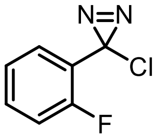

Following the general procedure IIIA, product **1h** was isolated in 52% yield as a colorless liquid. **1h**: R_f =0.4 (silica gel, 5% EtOAc in hexanes); **1H NMR** (400 MHz, CDCl3) δ 7.51 (td, J = 7.6, 1.8 Hz, 1H), 7.43 – 7.35 (m, 1H), 7.18 (td, J = 7.6, 1.2 Hz, 1H), 7.08 (ddd, J = 10.8, 8.3, 1.1 Hz, 1H). Spectroscopic data are in agreement with those in the literature.[19]

3-chloro-3-(3-methoxyphenyl)-3H-diazirine (1i):

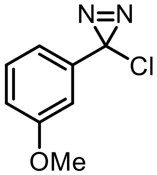

Following the general procedure IIIA, product **1i** was isolated in 64% yield as a pale-yellow liquid. **1i**: R_f =0.5 (silica gel, 5% EtOAc in hexanes); **1H NMR** (400 MHz, CDCl3) δ 7.29 (t, J = 8.0 Hz, 1H), 6.93 (ddd, J = 8.3, 2.5, 0.9 Hz, 1H), 6.69 (t, J = 2.2 Hz, 1H), 6.62 (ddd, J = 7.8, 1.9, 0.9 Hz, 1H), 3.82 (s, 3H). Spectroscopic data are in agreement with those in the literature.[19]

3-chloro-3-(m-tolyl)-3H-diazirine (1j):

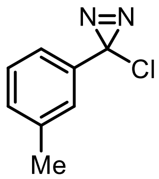

Following the general procedure IIIA, product **1j** was isolated in 59% yield as a colorless liquid. **1j**: R_f = 0.6 (silica gel, 5% EtOAc in hexanes); **1H NMR** (400 MHz, CDCl3) δ 7.29 (d, J = 7.8 Hz, 1H), 7.20 (d, J = 7.6 Hz, 1H), 6.91 (dt, J = 4.0, 1.7 Hz, 2H), 2.37 (s, 3H). Spectroscopic data are in agreement with those in the literature.[19]

3-chloro-3-(3-fluorophenyl)-3H-diazirine (1k):

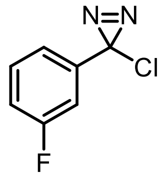

Following the general procedure IIIA, product **1k** was isolated in 60% yield as a colorless liquid. **1k**: R_f =0.5 (silica gel, 5% EtOAc in hexanes); **1H NMR** (400 MHz, CDCl3) δ 7.36 (td, J = 8.1, 5.7 Hz, 1H), 7.10 (tdd, J = 8.3, 2.5, 0.9 Hz, 1H), 6.92 (dt, J = 9.7, 2.2 Hz, 1H), 6.82 (ddd, J = 7.9, 1.9, 1.0 Hz, 1H). Spectroscopic data are in agreement with those in the literature.[19]

3-chloro-3-(3-chlorophenyl)-3H-diazirine (1l):

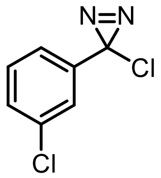

Following the general procedure IIIA, product **1l** was isolated in 62% yield as a pale-yellow liquid. **1l**: R_f =0.4 (silica gel, 5% EtOAc in hexanes); **1H NMR** (400 MHz, CDCl3) δ 7.38 (ddd, J = 8.0, 2.0, 1.2 Hz, 1H), 7.35 – 7.29 (m, 1H), 7.16 (td, J = 1.9, 0.5 Hz, 1H), 6.95 (ddd, J = 7.7, 1.9, 1.2 Hz, 1H). Spectroscopic data are in agreement with those in the literature. [19]

3-(3-bromophenyl)-3-chloro-3H-diazirine (1m):

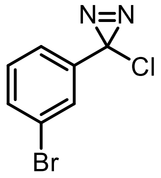

Following the general procedure IIIA, product **1m** was isolated in 49% yield as a pale-yellow liquid. **1m**: R_f =0.5 (silica gel, 5% EtOAc in hexanes); **1H NMR** (400 MHz, CDCl3) δ 7.53 (ddd, J = 8.0, 1.9, 1.0 Hz, 1H), 7.31 (t, J = 1.9 Hz, 1H), 7.29 – 7.23 (m, 1H), 7.00 (ddd, J = 8.0, 1.9, 1.0 Hz, 1H). Spectroscopic data are in agreement with those in the literature.[19]

3-chloro-3-(3-nitrophenyl)-3H-diazirine (1n):

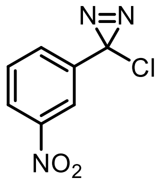

Following the general procedure IIIA, product **1n** was isolated in 45% yield as a colorless liquid. **1n**: R_f =0.4 (silica gel, 10% EtOAc in hexanes); **1H NMR** (400 MHz, CDCl3) δ 8.27 (ddd, J = 8.2, 2.2, 1.0 Hz, 1H), 8.05 (t, J = 2.1 Hz, 1H), 7.61 (t, J = 8.1 Hz, 1H), 7.42 (ddd, J = 7.9, 2.0, 1.0 Hz, 1H). Spectroscopic data are in agreement with those in the literature.[19]

3-chloro-3-(thiophen-2-yl)-3H-diazirine (1o):

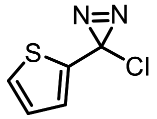

Following the general procedure IIIA, product **1o** was isolated in 49% yield as an orange liquid. **1o**: R_f=0.4 (silica gel, 5% EtOAc in hexanes); ^{1}**H NMR** (400 MHz, $CDCl_3$) δ 7.27 (d, *J*=4.5 Hz, 1H), 6.98 (dd, *J*=4.5, 3.6 Hz, 1H), 6.75 (d, *J*=3.6 Hz, 1H). Spectroscopic data are in agreement with those in the literature.[20]

IIIB. Synthesis of Pyrazoles and Indazoles

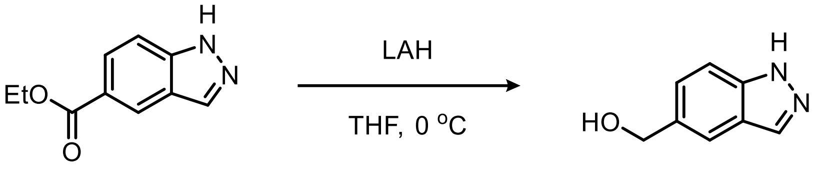

S-1 4q

To an oven dried 100 mL round bottom flask equipped with a stir bar, $LiAlH_4$ (130 mg, 3.4 mmol) was added and sparged with nitrogen. The round bottom flask was cooled to 0 °C in an ice bath and THF (15 mL) was added. To this stirred solution, **S-1** (400 mg, 2.27 mmol) in THF (15 mL) was added dropwise over 10 minutes. The solution was allowed to stir for 3 h. While remaining at 0 °C, aqueous sat. Rochelle's salt solution (20 mL) was added and allowed to stir until complete dissolution of the aluminum emulsion. The layers were separated, and the aqueous layer was extracted with EtOAc (3 x 15mL). The combined organic layers were washed with brine, dried over anhydrous Na_2SO_4, filtered, and concentrated. The resulting crude mixture was purified by silica gel chromatography eluted with Hexane/EtOAc (2:3), affording **4q** in 79% yield as a tan solid. **4q:** ^{1}**H NMR** (400 MHz, DMSO) δ 12.99 (s, 1H), 8.03 (t, *J* = 1.3 Hz, 1H), 7.67 (dt, *J* = 1.6, 0.8 Hz, 1H), 7.49 (dt, *J* = 8.6, 1.0 Hz, 1H), 7.32 (dd, *J* = 8.5, 1.5 Hz, 1H), 5.17 (t, *J* = 5.7 Hz, 1H), 4.58 (dd, *J* = 5.8, 0.9 Hz, 2H). ^{13}**C NMR** (101 MHz, DMSO) δ 139.73, 134.99, 133.79, 126.27, 123.22, 118.24, 110.21, 63.75. **HRMS** (ESI-TOF) calcd for $C_8H_8N_2O^+$ $[M+H]^+$ 149.0710, found 149.0709.

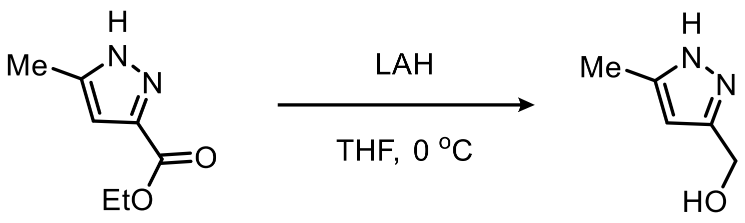

S-2 2i

To an oven dried 100 mL round bottom flask equipped with a stir bar, $LiAlH_4$ (185 mg, 4.86 mmol) was added and sparged with nitrogen. The round bottom flask was cooled to 0 °C in an ice bath and THF (20 mL) was added. To this stirred solution, **S-2** (500 mg, 3.24 mmol) in THF (20 mL) was added dropwise over 10 minutes. The solution was allowed to stir for 3 h. While remaining at 0 °C, aqueous sat. Rochelle's salt solution (20 mL) was added and allowed to stir until complete dissolution of the aluminum emulsion. The layers were separated, and the aqueous layer was extracted with EtOAc (3 x 15mL). The combined organic layers were washed with brine, dried over anhydrous Na_2SO_4, filtered, and concentrated. The resulting crude mixture was purified by silica gel chromatography eluted with CH_2Cl_2/MeOH (9:1), affording **2i** in 79% yield as a tan solid. **^{1}H NMR** (400 MHz, DMSO/1 μL TFA) δ 5.96 (s, 1H), 4.39 (s, 2H), 2.19 (s, 3H). **^{13}C NMR** (101 MHz, DMSO/1 μL TFA) δ 149.96, 142.35, 102.98, 56.73, 11.98. **HRMS** (ESI-TOF) calcd for $C_5H_8N_2O^+$ $[M+H]^+$ 113.0710, found 113.0708.

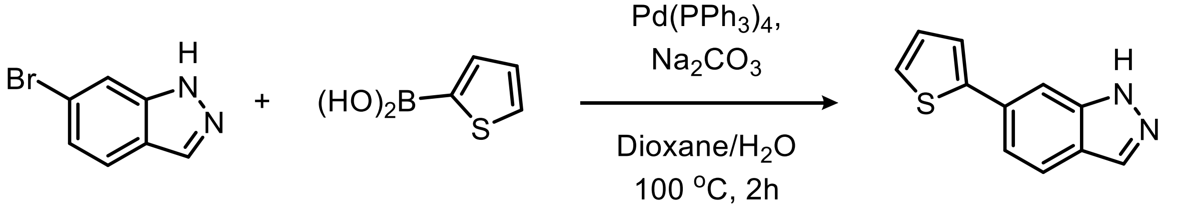

S-3 4u

A mixture of **S-3**, $Pd(PPh_3)_4$, Na_2CO_3, and thiophen-2-ylboronic acid were charged to a 100 mL round bottom flask equipped with a stir bar. The flask was degassed and left under a nitrogen environment. A degassed solution of 1:1 water and dioxane (15 mL : 15 mL) was added to the flask, and the mixture was allowed to stir at 100 °C for 2 h. The resultant crude mixture was diluted with 20 mL EtOAc and sonicated for 10 minutes. The solution was then passed through a silica plug and the aqueous layer was extracted with EtOAc (3 x 10 mL). The combined organic layers were washed with brine, dried over anhydrous Na_2SO_4, filtered, and concentrated. The resulting crude mixture was purified by silica gel chromatography eluted with Hexane/EtOAc (7:3), yielding **4u** in 50% yield as a white solid**.** **^{1}H NMR** (400 MHz, DMSO) δ 13.13 (s, 1H), 8.09 (t, *J* = 1.3 Hz, 1H), 7.83 – 7.74 (m, 2H), 7.57 (ddd, *J* = 11.8, 4.4, 1.2 Hz, 2H), 7.45 (dd, *J* = 8.4, 1.5 Hz, 1H), 7.16 (dd, *J* = 5.1, 3.6 Hz, 1H). **^{13}C NMR** (101 MHz, DMSO) δ 144.28, 140.85, 134.09, 132.12, 129.04, 126.20, 124.58, 122.76, 121.76, 119.52, 106.54. **HRMS** (ESI-TOF) calcd for $C_{11}H_8N_2S^+$ $[M+H]^+$ 201.0481, found 201.0488.

IIIC. Synthesis of SEM-Protected Pyrazoles

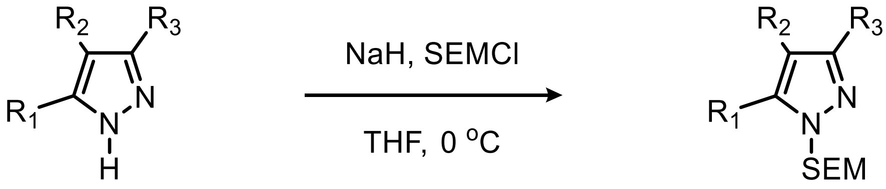

To a stirred solution of NaH (60% dispersion in mineral oil, 2.1 mmol, 1.05 equiv) in THF (6 mL) under N_2 cooled at 0 °C was added dropwise a solution of pyrazole (2.0 mmol, 1.0 equiv) in THF (4 mL). The mixture was allowed to stir for 30 min at 0 °C followed by subsequent addition of SEMCl (2.1 mmol, 1.05 equiv) in THF (2 mL) dropwise over 15 minutes. The resulting mixture was allowed to warm to room temperature and stirred overnight. To quench the reaction, water (2 mL) was added, and the organic layer was separated. The aqueous layer was extracted with EtOAc (3 x 10mL), and the combined organic layers were washed with brine, dried over anhydrous Na_2SO_4, filtered, and concentrated. The resulting crude mixture was purified by silica gel chromatography eluted with Hexane/EtOAc (9:1).

3,5-diphenyl-1-((2-(trimethylsilyl)ethoxy)methyl)-1H-pyrazole (2p-SEM):

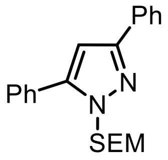

2p-SEM

Following the general procedure IIIc, product **2p-SEM** was isolated in 92% yield as a white solid. **^{1}H NMR** (400 MHz, $CDCl_3$) δ 7.92 – 7.84 (m, 2H), 7.72 – 7.64 (m, 2H), 7.52 – 7.39 (m, 5H), 7.36 – 7.30 (m, 1H), 6.71 (s, 1H), 5.47 (s, 2H), 3.84 – 3.66 (m, 2H), 1.03 – 0.90 (m, 2H), -0.00 (s, 9H). Spectroscopic data are in agreement with those in the literature.[21]

ethyl 5-methyl-1-((2-(trimethylsilyl)ethoxy)methyl)-1H-pyrazole-4-carboxylate (2af) and ethyl 3-methyl-1-((2-(trimethylsilyl)ethoxy)methyl)-1H-pyrazole-4-carboxylate (2q'):

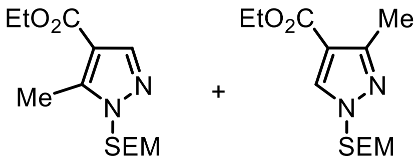

2q-SEM 2q-SEM'

Following the general procedure IIIc, products **2q-SEM** and **2q-SEM'** were isolated as a 1:1.1 mixture in a combined 82% yield as a clear oil**.** Regioisomers were assigned using 1D NOESY experiments. **2q: ^{1}H NMR** (500 MHz, $CDCl_3$) δ 7.83 (s, 1H), 5.42 (s, 2H), 4.28 (qd, *J* = 7.1, 4.9 Hz, 2H), 3.60 – 3.51 (m, 2H), 2.60 (s, 3H), 1.34 (td, *J* = 7.1, 5.1 Hz, 3H), 0.95 – 0.84 (m, 2H), -0.04 (d, *J* = 7.6 Hz, 9H). **2q'**: **^{1}H NMR** (500 MHz, $CDCl_3$) δ 7.97 (s, 1H), 5.33 (s, 2H), 4.28 (qd, *J* = 7.1, 4.9 Hz, 2H), 3.60 – 3.51 (m, 2H), 2.46 (s, 3H), 1.34 (td, *J* = 7.1, 5.1 Hz, 3H), 0.95 – 0.84 (m, 2H), -0.04 (d, *J* = 7.6 Hz, 9H). **2q & 2q': ^{13}C NMR** (101 MHz, $CDCl_3$) δ 165.21, 165.06, 152.84, 145.49, 142.29, 135.62, 114.98, 114.52, 81.70, 79.54, 68.53, 68.10, 61.40, 61.35, 19.23, 15.84, 14.93, 11.73, 0.00, -0.02. **HRMS** (ESI-TOF) calcd for $C_{13}H_{24}N_2O_3Si^+$ $[M+H]^+$ 285.1620, found 285.1631.

2q-SEM: 1D Selective Gradient NOESY – freq: 5.42ppm (500 MHz, $CDCl_3$): δ 3.56, 2.60, -0.04.

2q-SEM': 1D Selective Gradient NOESY – freq: 5.33ppm (500 MHz, $CDCl_3$): δ 7.97, 3.56, -0.04.

4-bromo-1-((2-(trimethylsilyl)ethoxy)methyl)-1H-pyrazole (2r-SEM):

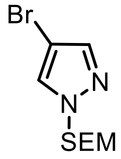

2r

Following the general procedure IIIc, product **2r-SEM** was isolated in 90% yield as a clear oil. ^{1}H NMR (400 MHz, $CDCl_3$) δ 7.61 (d, *J* = 0.7 Hz, 1H), 7.54 – 7.49 (m, 1H), 5.40 (s, 2H), 3.61 – 3.52 (m, 2H), 0.97 – 0.87 (m, 2H), -0.00 (s, 9H). Spectroscopic data are in agreement with those in the literature.[22]

IV. Synthesis of Rosuvastatin Analogue

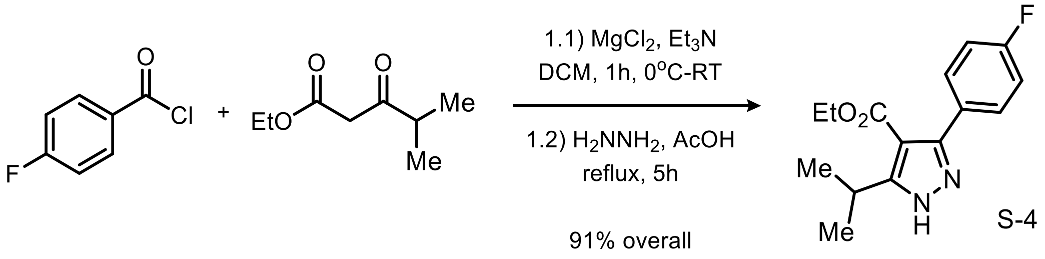

To an oven dried 100 mL round bottom round-bottom flask with a stir bar, dry magnesium chloride was added followed by nitrogen sparging. Dry CH_2Cl_2 (12.5 mL) was added followed by addition of ethyl 4-methyl-3-oxopentanoate (12.5 mmol). The mixture was cooled to 0 °C in an ice bath, with subsequent addition of Et_3N (25 mmol) followed by 15 minutes of stirring at 0 °C. 4-Fluorobenzoyl chloride (12.5 mmol) was then added to the mixture at 0 °C, then stirred at 25 °C for 1 h. The reaction was cooled to 0 °C and quenched with 6M HCl (10 mL). The reaction was extracted with CH_2Cl_2 (3 x 10 mL), and the combined organic layers were dried with Na_2SO_4, filtered, concentrated and carried forward without any further purification. The crude mixture was then added to a round bottom flask with a stir bar and diluted with glacial acetic acid (25 mL). Hydrazine monohydrate (13 mmol) was added dropwise to the solution and was then heated at 50 °C for 5 h. The crude mixture was cooled to room temperature and diluted with water (50 mL) and EtOAc (50 ml) and the layers were separated. The aqueous layer was extracted with EtOAc (3 x 20 mL), and the combined organic layers were washed with sat. Na_2CO_3 until gas evolution ceased. The combined organic layers were washed with brine (1 x 25 mL), dried over Na_2SO_4, filtered, and concentrated. The resulting crude mixture was purified by silica gel chromatography eluted with Hexane/EtOAc (8:2), affording **S-4** in 91% overall yield as a white solid. **^{1}H NMR** (400 MHz, $CDCl_3$) δ 7.61 – 7.51 (m, 2H), 7.15 – 7.04 (m, 2H), 4.21 (q, *J* = 7.1 Hz, 2H), 3.69 (hept, *J* = 7.0 Hz, 1H), 1.36 (d, *J* = 7.0 Hz, 6H), 1.21 (t, *J* = 7.1 Hz, 3H). **^{13}C NMR** (101 MHz, $CDCl_3$) δ 164.07 (d, *J* = 30.0 Hz), 161.75, 156.70, 151.23, 131.18 (d, *J* = 8.3 Hz), 128.25, 114.74 (d, *J* = 21.6 Hz), 107.65, 59.99, 26.30, 21.57, 14.01. **^{19}F NMR** (376 MHz, $CDCl_3$) δ -113.14. **HRMS** (ESI-TOF) calcd for $C_{15}H_{17}FN_2O_2^+$ $[M]^+$ 276.1274, found 276.1280.

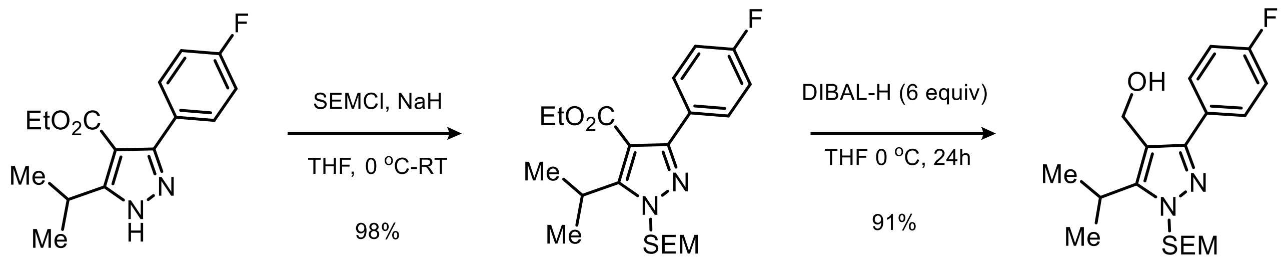

S-4 S-5 2s-SEM

S-4 was SEM-protected using general procedure IIIC in 98% yield as a colorless oil to afford a 1:2.1 mixture consisting of **S-5** and **S-5'**respectively. This product mixture (1.0 g, 2.19 mmol) was then charged to an oven dried 100 mL round bottom flask with a stir bar and was sparged with nitrogen. THF (30 mL) was added, and the resulting mixture was cooled to 0 °C in an ice bath. DIBAL-H (6.57 mmol) was added dropwise to the solution was stirred at 0 °C for 1 h, followed by stirring at 25 °C for 10 h. The solution was cooled again to 0 °C in an ice bath and additional Dibal-H (6.57 mmol) was added dropwise. After 1 h of stirring, the reaction as allowed to warm to 25 °C and stir overnight. Saturated aqueous Rochelle's salt (20 mL) was added to the solution at 0 °C and stirred until the aluminum emulsion was consumed. The layers were separated, and the aqueous layer was extracted with EtOAc (3 x 20 mL). The combined organic layers were washed with brine (1 x 25 mL), dried over Na_2SO_4, filtered and concentrated. The resulting crude mixture was purified by silica gel chromatography eluted with Hexane/EtOAc (7:3), affording **2s-SEM** and **2s-SEM'** as a 1:2 mixture in 91% yield as a white solid.

ethyl 3-(4-fluorophenyl)-5-isopropyl-1-((2-(trimethylsilyl)ethoxy)methyl)-1H-pyrazole-4-carboxylate (S-5):

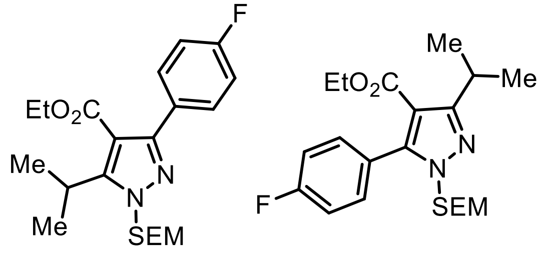

S-5 S-5'

Regioisomers were assigned using 1D NOESY experiments.

S-5: **^{1}H NMR** (500 MHz, $CDCl_3$) δ 7.53 – 7.47 (m, 2H), 7.09 – 7.04 (m, 2H), 5.52 (s, 2H), 4.18 (q, *J* = 7.1 Hz, 2H), 3.59 – 3.53 (m, 1H), 1.43 (d, *J* = 7.1 Hz, 6H), 1.15 (t, *J* = 7.1 Hz, 2H), 1.08 (t, *J* = 7.1 Hz, 6H), 0.96 – 0.90 (m, 2H), -0.01 (s, 9H). **^{19}F NMR** (377 MHz, $CDCl_3$) δ -114.22. **S-5': ^{1}H NMR** (500 MHz, $CDCl_3$) δ 7.46 – 7.39 (m, 2H), 7.17 – 7.11 (m, 2H), 5.17 (s, 2H), 4.10 (q, *J* = 7.1 Hz, 2H), 3.68 – 3.62 (m, 1H), 1.34 (d, *J* = 6.9 Hz, 6H), 1.08 (t, *J* = 7.1 Hz, 3H), 0.90 – 0.85 (m, 2H), -0.02 (s, 9H). **^{19}F NMR** (377 MHz, $CDCl_3$) δ -111.90.

^{13}C NMR (101 MHz, $CDCl_3$) δ 165.85, 165.15, 164.67 (d, *J* = 248.9 Hz), 164.25 (d, *J* = 246.6 Hz), 161.27, 154.54, 152.93, 148.11, 133.76 (d, *J* = 8.4 Hz), 132.29 (d, *J* = 8.1 Hz), 131.13 (d, *J* = 3.4 Hz), 127.11 (d, *J* = 3.5 Hz), 116.50 (d, *J* = 22.0 Hz), 116.15 (d, *J* = 21.6 Hz), 112.08, 111.84, 80.43, 78.83, 68.43, 68.15, 61.74, 61.18, 28.59, 27.46, 23.44, 21.89, 19.36, 19.30, 15.37, 15.34, 0.04, -0.00.

HRMS (ESI-TOF) calcd for $C_{21}H_{31}FN_2O_3Si^+$ $[M]^+$ 406.2088, found 406.2080.2264.

S-5: 1D Selective Gradient NOESY – freq: 5.52ppm (500 MHz, $CDCl_3$): δ 3.59, 1.44, -0.01.

S-5': 1D Selective Gradient NOESY – freq: 5.17ppm (500 MHz, $CDCl_3$): δ 7.43, 3.66, 1.35, 0.87, 0.02.

(5-(4-fluorophenyl)-3-isopropyl-1-((2-(trimethylsilyl)ethoxy)methyl)-1H-pyrazol-4-yl)methanol (2s-SEM):

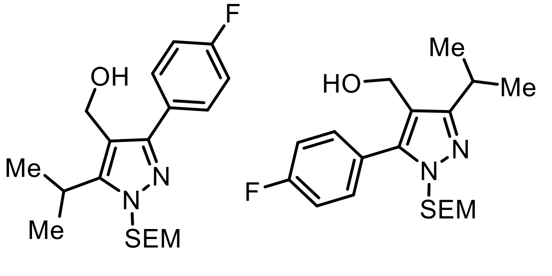

2s-SEM 2s-SEM'

Regioisomers were assigned using 1D NOESY experiments.

2s-SEM: ^{1}H NMR (400 MHz, $CDCl_3$) δ 7.77 – 7.67 (m, 1H), 7.13 – 7.05 (m, 2H), 5.49 (s, 2H), 4.63 (d, *J* = 3.0 Hz, 2H), 3.66 – 3.57 (m, 2H), 3.35 (h, *J* = 7.2 Hz, 1H), 1.43 (d, *J* = 7.2 Hz, 6H), 0.96 – 0.85 (m, 2H), -0.02 (d, *J* = 1.5 Hz, 9H). **^{19}F NMR** (376 MHz, $CDCl_3$) δ -114.41. **2s-SEM'**: **^{1}H NMR** (400 MHz, $CDCl_3$) δ 7.61 – 7.51 (m, 2H), 7.21 – 7.13 (m, 2H), 5.25 (s, 2H), 4.44 (s, 2H), 3.72 – 3.66 (m, 2H), 3.14 (hept, *J* = 7.0 Hz, 1H), 1.36 (d, *J* = 6.9 Hz, 6H), 0.96 – 0.85 (m, 2H), -0.02 (d, *J* = 1.5 Hz, 9H). **^{19}F NMR** (376 MHz, $CDCl_3$) δ -112.28

^{13}C NMR (101 MHz, $CDCl_3$) δ 164.51 (d, *J* = 249.3 Hz), 164.34 (d, *J* = 214.6 Hz), 158.64, 151.93, 150.17, 144.23, 133.44 (d, *J* = 8.2 Hz), 131.43 (d, *J* = 8.1 Hz), 130.78 (d, *J* = 3.1 Hz), 126.90 (d, *J* = 3.2 Hz), 118.17, 117.16 (d, *J* = 21.5 Hz), 116.90 (d, *J* = 21.5 Hz), 116.89, 79.94, 78.81, 68.08, 67.79, 56.18, 28.14, 27.34, 24.11, 23.66, 19.33, 0.05, 0.00.. The ^{13}C-NMR peaks at 56.18ppm and 19.33ppm corresponding to the benzylic methylene and distal SEM methylene are identical in the regioisomeric compounds, confirmed by HSQC. **^{19}F NMR** (376 MHz, $CDCl_3$) δ -114.41. **HRMS** (ESI-TOF) calcd for $C_{19}H_{29}FN_2O_2Si^+$ $[M]^+$ 364.1982, found 364.1993.

[^{1}H, ^{13}C]-HSQC (400 MHz, 101MHz, $CDCl_3$) δ (4.63, 54.68), (4.45, 54.68), (0.91, 18.11) (0.91, 18.11).

2s-SEM: 1D Selective Gradient NOESY – freq: 5.49ppm (500 MHz, $CDCl_3$): δ 3.59, 1.44, -0.02.

2s-SEM': 1D Selective Gradient NOESY – freq: 5.25ppm (500 MHz, $CDCl_3$): δ 7.54, 3.66, 1.36, 0.88, 0.00.

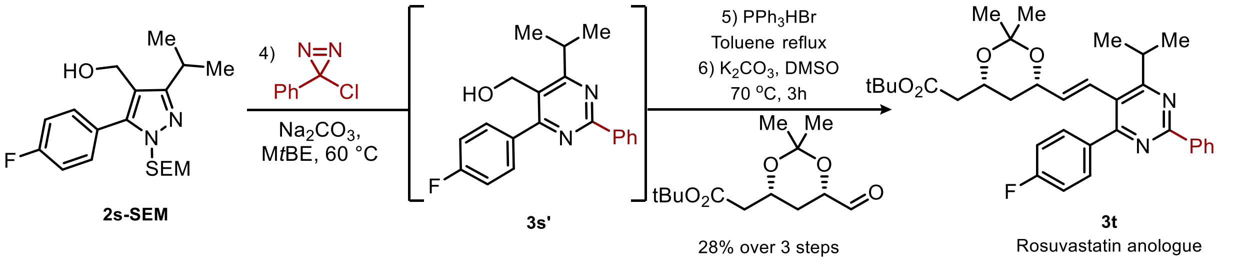

2s-SEM was prepared using general procedure IIA with a slight modification. To an oven dried 2-dram screw cap vial equipped with a stir bar and PTFE/white silicone septum, **2s-SEM** and **2s-SEM'**(0.3 mmol.), diazirine **1a** (0.9 mmol) and Na_2CO_3 (0.9 mmol) were added, followed by a brief nitrogen sparge. Dry M*t*BE (3 mL) was then added, and the vial was sealed with electric tape. The mixture was then allowed to stir at 60 °C for 12 h and then allowed to cool to 25 °C. An additional 3 equiv. of diazirine (0.9 mmol) were added and the mixture was then allowed to stir at 60 °C for an additional 12 hours. The crude mixture was cooled and diluted with CH_2Cl_2 and sonicated for 10

minutes. The solution was filtered and the solid was washed with CH_2Cl_2. The resulting filtrate was concentrated under reduced pressure, yielding **3s'**. The resultant crude mixture was added to an oven dried 2-dram screw cap vial equipped with a dry stir bar and PTFE white silicone septum. Triphenylphosphine hydrobromide (0.3mmol) was added to the vial followed by subsequent sparging of the vial with nitrogen. Dry toluene (3mL) was added to the vial and the vial was sealed with electrical tape. The reaction was allowed to reflux for 12 h and then allowed to cool to room 25 ºC. The resultant precipitate was filtered and washed with hexanes. The precipitate was added to a 1-dram screw cap vial equipped with a dry stir bar and PTFE white silicone septum. K_2CO_3 (0.66 mmol) was added followed by a nitrogen sparge. Anhydrous DMSO (1.5 mL) was added and the mixture was stirred at 25 ºC for 1h. Tert-butyl 2-((4R,6S)-6-formyl-2,2-dimethyl-1,3-dioxan-4-yl)acetate (0.22 mmol) in anhydrous DMSO (0.5 mL) was then added to the solution, followed by stirring at 70 ºC for 3 h. The crude mixture was cooled to 25 ºC, diluted with water (3 mL) and EtOAc (3 mL) and the layers were separated. The aqueous layer was extracted with EtOAc (3 x 3 mL), and the combined organic layers were washed with brine (5 mL), dried over Na_2SO_4, filtered and concentrated. The resulting crude mixture was purified by silica gel chromatography eluted with Hexane/EtOAc gradient from 1% - 5% EtOAc, affording **3t** in 28% yield over 3 steps as a white solid.

Crude Spectrum for NMR Yield of 3s

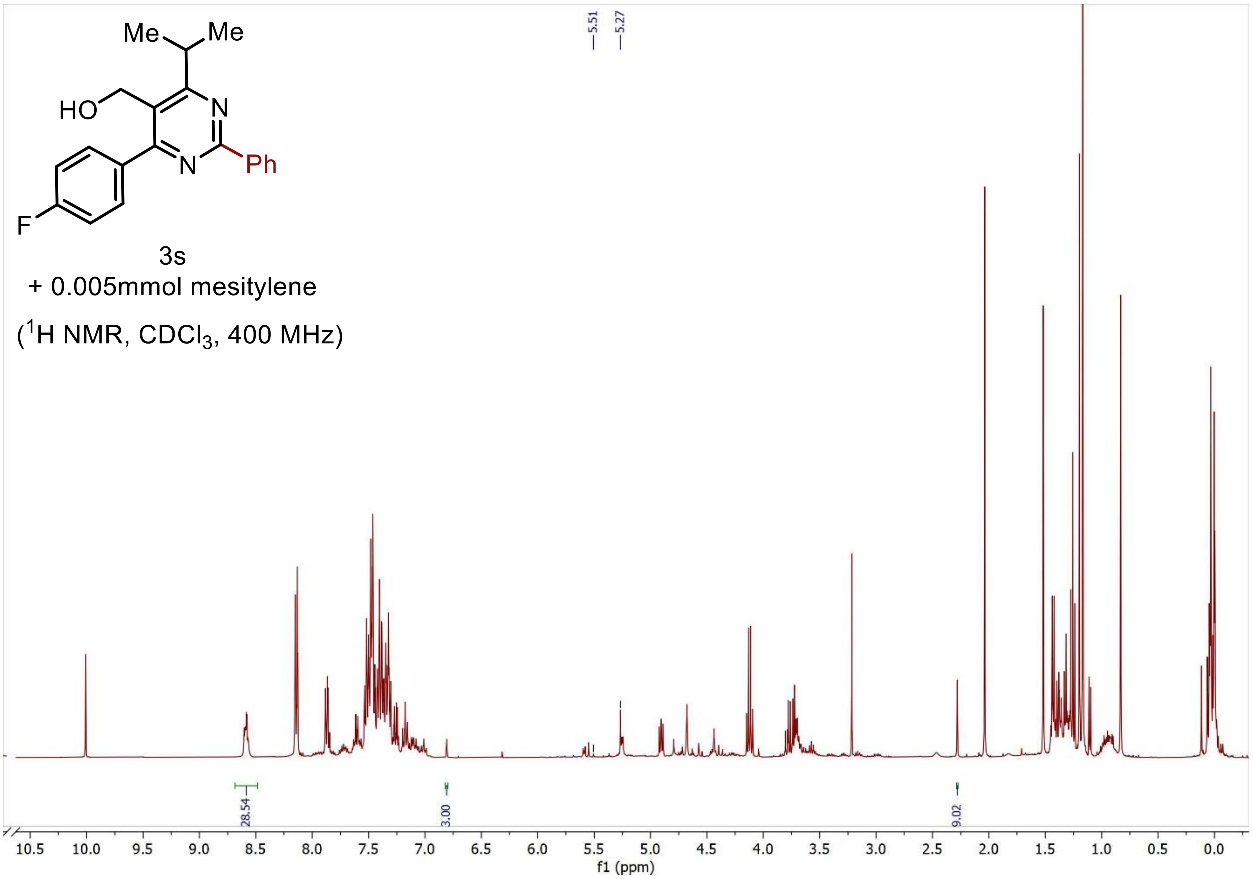

tert-butyl 2-((4R,6S)-6-((E)-2-(4-(4-fluorophenyl)-6-isopropyl-2-phenylpyrimidin-5-yl)vinyl)-2,2-dimethyl-1,3-dioxan-4-yl)acetate (3t):

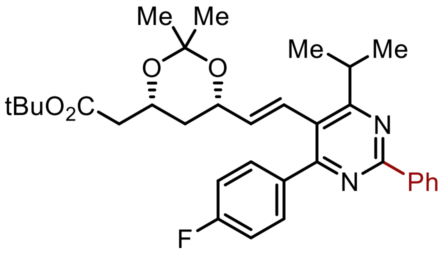

^{1}H NMR (400 MHz, $CDCl_3$) δ 8.66 – 8.52 (m, 2H), 7.75 – 7.67 (m, 2H), 7.54 – 7.43 (m, 3H), 7.12 (td, *J* = 8.5, 4.0 Hz, 2H), 6.69 – 6.52 (dd, *J* = 16.2, 1.4 Hz,, 1H), 5.59-5.51 (dd, *J* = 16.2, 5.4 Hz, 1H), 4.45 (dd, *J* = 11.5, 5.4 Hz, 1H), 4.35 – 4.22 (m, 1H), 3.46 (hept, *J* = 6.7 Hz, 1H), 2.46 (dd, *J* = 15.3, 6.8 Hz, 1H), 2.31 (dd, *J* = 15.3, 6.3 Hz, 1H), 1.59 – 1.26 (m, 21H), 1.15 (q, *J* = 11.9 Hz, 2H). *J*-coupling values of vinyl peaks 6.69-6.52 and 5.59-5.51 (16.2 Hz) indicate a trans configuration. **^{13}C NMR** (101 MHz, $CDCl_3$) δ 173.55, 164.40, 163.03, 161.82, 137.99, 137.62, 135.27, 132.20 (d, *J* = 8.4 Hz), 130.37, 128.30 (d, *J* = 16.5 Hz), 124.60, 123.82, 114.91 (d, *J* = 21.5 Hz), 98.93, 80.76, 69.40, 65.90, 42.54, 36.06, 31.91, 30.05, 28.12, 21.90, 21.81, 19.78.**^{19}F NMR** (376 MHz, $CDCl_3$) δ -112.49. **HRMS** (ESI-TOF) calcd for $C_{33}H_{39}FN_2O_4^+$ $[M]^+$ 546.2894, found 546.2894.

V. Synthesis, Dearylation, and Expansion of Tepoxalin Ester

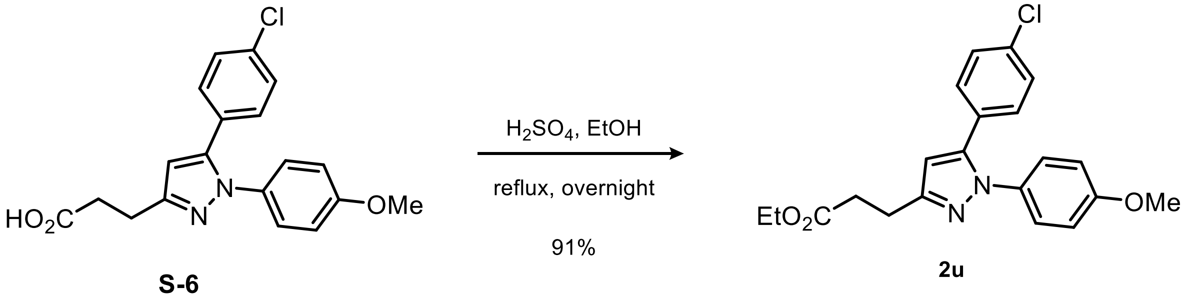

S-6 **2u**

A stirred solution of **S-6** (1.2 mmol) in EtOH (5 mL) was treated dropwise with 0.1 mL of conc. H_2SO_4 and was allowed to stir under refluxing conditions overnight. The resulting solution was then concentrated under reduced pressure and the crude oil was treated with 10 mL of sat. $NaHCO_3$ solution. The aqueous solution was extracted with EtOAc (3 x 10 mL), dried over anhydrous Na_2SO_4, filtered, and concentrated under reduced pressure. The crude mixture was purified by silica gel chromatography eluted with a 15% EtOAc/hexane solution, yielding **2u** in 91% overall yield. **^{1}H NMR** (500 MHz, $CDCl_3$) δ 7.26 (d, *J* = 8.3 Hz, 2H), 7.19 – 7.15 (m, 2H), 7.13 (d, *J* = 8.3 Hz, 2H), 6.87 – 6.83 (m, 2H), 6.32 (s, 1H), 4.18 (q, *J* = 7.1 Hz, 2H), 3.81 (s, 3H), 3.05 (t, *J* = 7.7 Hz, 2H), 2.76 (t, *J* = 7.7 Hz, 2H), 1.27 (t, *J* = 7.1 Hz, 3H). **^{13}C NMR** (101 MHz, $CDCl_3$) δ 173.04, 158.83, 151.87, 142.37, 134.05, 133.13, 129.81, 129.15, 128.66, 126.60, 114.17, 106.29, 60.43, 55.49, 33.97, 23.60, 14.27. **HRMS** (ESI-TOF) calcd for $C_{21}H_{21}ClN_2O_3^+$ $[M+H]^+$ 385.1314, found 385.1313.

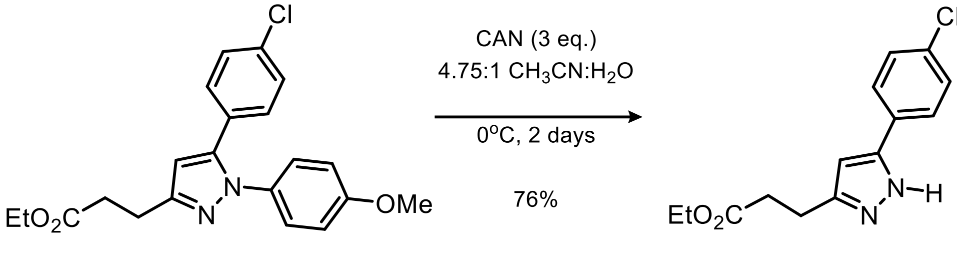

A solution of **2u** (610 mg, 1.58 mmol) in 22 mL CH_3CN was treated dropwise with an aqueous solution (4.5 mL) of CAN (2.61 g, 4.74 mmol) at 0 °C and was allowed to stir for 48 hours. The reaction mixture was then diluted with water (30 mL) and the CH_3CN was removed under reduced pressure. The aqueous solution was extracted with CH_2Cl_2 (4 x 20 mL), and the combined organic layers were washed with saturated aqueous $NaHCO_3$ (4 x 25 mL). The organic layer was dried over Mg_2SO_4, filtered, and concentrated under reduced pressure. The crude mixture was purified by silica gel chromatography eluted with EtOAc/hexane gradient from 15% EtOAc in hexane to 40% EtOAc in hexane, affording **S-7** in 76% as a white solid. **^{1}H NMR** (400 MHz, $CDCl_3$) δ 7.71 – 7.63 (m, 2H), 7.39 – 7.29 (m, 2H), 6.35 (s, 1H), 4.17 (q, *J* = 7.2 Hz, 2H), 3.00 (t, *J* = 6.8 Hz, 2H), 2.69 (dd, *J* = 7.3, 6.3 Hz, 2H), 1.26 (t, *J* = 7.1 Hz, 3H). **^{13}C NMR** (101 MHz, $CDCl_3$) δ 173.39, 149.58, 145.29, 133.54, 131.39, 128.86, 126.80, 101.37, 61.01, 33.70, 21.16, 14.19. **HRMS** (ESI-TOF) calcd for $C_{14}H_{15}ClN_2O_2^+$ $[M+H]^+$ 279.0895, found 279.0901

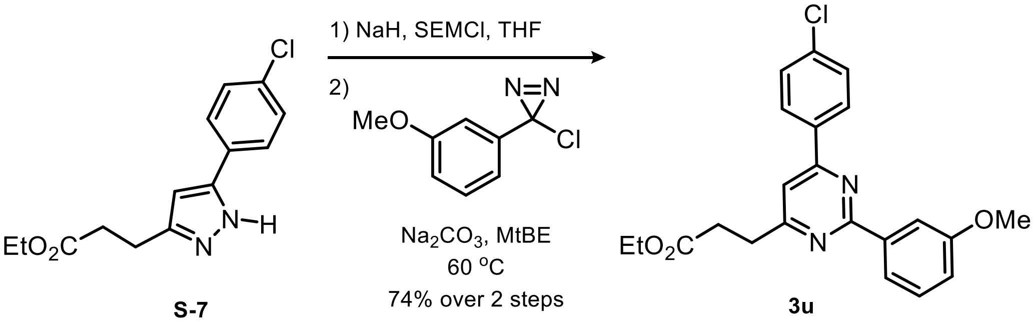

S-7 was SEM-protected and isolated using general procedure IIIC in 91% yield. The resulting 1:1.2 mixture of SEM-protected pyrazoles (**S-8** and **S-8')** was then subject to general procedure II with a slight modification. To an oven dried 2-dram screw cap vial equipped with a stir bar and PTFE/white silicone septum, **S-8 and S-8'** (0.2 mmol.), diazirine **1i** (0.6 mmol) and Na_2CO_3 (0.6 mmol) were added, followed by a brief nitrogen sparge. Dry M*t*BE (2 mL) was then added, and the vial was sealed with electric tape. The mixture was then allowed to stir at 60 °C for 12 h and then allowed to cool to 25 °C. An additional 3 equiv. of diazirine (0.6 mmol) were added and the mixture was then allowed to stir at 60 °C for an additional 12 hours. Upon cooling to room temperature, TBAF (0.6 mmol) was added then stirred at 60 °C for 1 h. The reaction mixture was allowed to cool to 25 °C and quenched by the addition of water (3mL), and the two phases were separated. The aqueous layer was extracted with EtOAc (3×5mL), and the combined organic layers were dried over anhydrous Na_2SO_4, filtered, and concentrated under reduced pressure. The obtained residue was purified by flash column chromatography to afford pyrimidine **3u** in 74% as a white solid.

ethyl 3-(5-(4-chlorophenyl)-1-((2-(trimethylsilyl)ethoxy)methyl)-1H-pyrazol-3-yl)propanoate (S-8):

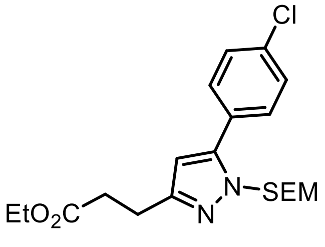

S-8

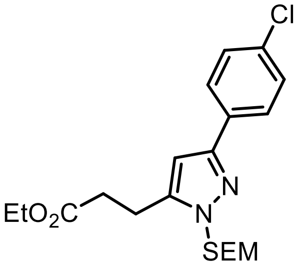

S-8'

Regioisomers were assigned using 1D NOESY experiments.

S-8: **^{1}H NMR** (400 MHz, $CDCl_3$) δ 7.58 – 7.50 (m, 2H), 7.39 – 7.30 (m, 2H), 6.21 (s, 1H), 5.31 (s, 2H), 4.15 (qd, *J* = 7.2, 2.7 Hz, 2H), 3.76 – 3.65 (m, 2H), 3.00-2.96 (t, *J* = 7.7 Hz, 2H), 2.82 – 2.66 (m, 2H), 1.25 (t, *J* = 7.1 Hz, 3H), 0.99 – 0.85 (m, 2H), -0.01 (s, 9H), -0.03 (s, 9H). **S-8': ^{1}H NMR** (400 MHz, $CDCl_3$) δ 7.73 – 7.65 (m, 2H), 7.43 – 7.39 (m, 2H), 6.37 (s, 1H), 5.46 (s, 2H), 4.15 (qd, *J* = 7.2, 2.7 Hz, 2H), 3.65 – 3.56 (m, 2H), 3.11 – 3.02 (m, 2H), 2.82 – 2.66 (m, 2H), 1.25 (t, *J* = 7.1 Hz, 3H), 0.99 – 0.85 (m, 2H), -0.03 (s, 9H).

^{13}C NMR (101 MHz, $CDCl_3$) δ 174.35, 173.56, 152.57, 150.97, 145.42, 145.11, 136.04, 134.80, 133.37, 131.45, 130.33, 130.20, 130.12, 128.30, 107.29, 104.31, 79.33, 79.06, 68.08, 67.69, 62.14, 61.81, 35.35, 34.67, 24.93, 22.10, 19.32, 19.15, 15.67, 15.64, -0.00, -0.02. **HRMS** (ESI-TOF) calcd for $C_{20}H_{29}ClN_2O_3Si^+$ $[M]^+$ 408.1636, found 408.1655.

S8: 1D Selective Gradient NOESY – freq: 5.31ppm (500 MHz, $CDCl_3$): δ 7.52, 3.70, 1.25, 0.93, -0.03.

S8': 1D Selective Gradient NOESY – freq: 5.46ppm (500 MHz, $CDCl_3$): δ 3.65, 3.07, 1.25, -0.03.

ethyl 3-(6-(4-chlorophenyl)-2-(3-methoxyphenyl)pyrimidin-4-yl)propanoate (3u):

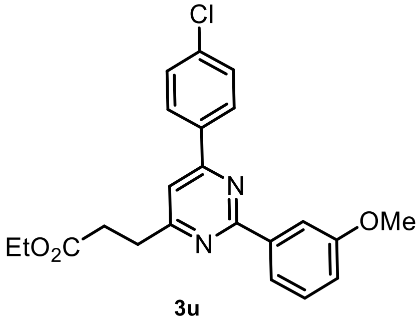

3u

^{1}H NMR (500 MHz, $CDCl_3$) δ 8.21 – 8.12 (m, 4H), 7.52 – 7.45 (m, 3H), 7.42 (t, *J* = 7.9 Hz, 1H), 7.05 (ddd, *J* = 8.2, 2.7, 1.0 Hz, 1H), 4.16 (q, *J* = 7.1 Hz, 2H), 3.93 (s, 3H), 3.21 (t, *J* = 7.1 Hz, 2H), 2.95 (t, *J* = 7.1 Hz, 2H), 1.24 (t, *J* = 7.1 Hz, 3H). **^{13}C NMR** (101 MHz, $CDCl_3$) δ 172.92, 169.34, 164.01, 162.52, 159.89, 139.34, 136.93, 135.64, 129.45, 129.11, 128.52, 120.93, 116.52, 113.59, 113.56, 60.61, 55.44, 32.47, 31.96, 14.23. **HRMS** (ESI-TOF) calcd for $C_{22}H_{21}ClN_2O_3^+$ $[M+H]^+$ 397.1314, found 397.1320.

VI: Mechanistic Experiments

VIA. Arrested Chloromethyl Product from Electron Poor Substrates

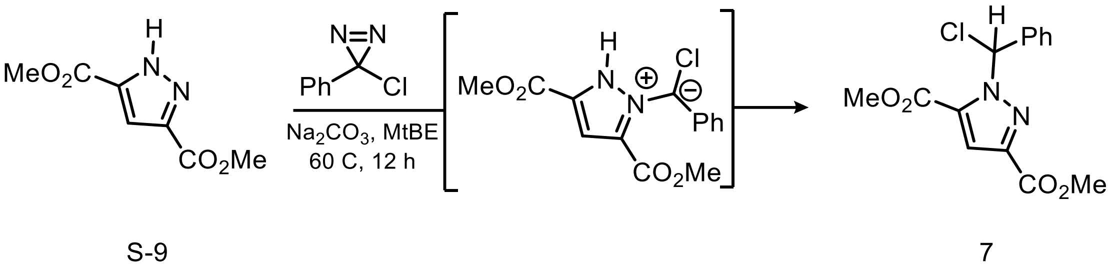

To an oven dried 2-dram screw cap vial equipped with a stir bar and PTFE/white silicone septum, **S-9** (0.1 mmol), diazirine (0.3 mmol) and Na_2CO_3 (0.3 mmol) were added, followed by a brief nitrogen sparge. Dry M*t*BE (1 mL) was then added, and the vial was sealed with electric tape. The mixture was then allowed to stir at 60 °C for 12 h and then allowed to cool to 25 °C. The crude mixture was cooled and diluted with CH_2Cl_2 and sonicated for 10 minutes. The solution was filtered and the solid was washed with CH_2Cl_2. The resulting filtrate was concentrated under reduced pressure, and the resulting residue afforded **S-8** in 94% yield by ^{1}H-NMR yield using mesitylene as an internal standard. The crude mixture was purified by preparatory TLC to afford pyrazole **7** as a clear oil in 61% yield. (R_f=0.4 in 5% EtOAc in hexanes). **7** reacts rapidly with water to afford benzaldehyde and **S-9,** so hygroscopic solvents such as alcohols were avoided in the isolation process but could not fully prevent decomposition of **S-8**. The α-chlorobenzyl C-H (sp^3) appears at 7.39 ppm with a carbon shift of 70.85ppm, in reasonable accordance with a reported α-methoxybenzyl analog (7.43-7.55ppm, 78.3-78.6ppm).[23] **^{1}H NMR** (400 MHz, $CDCl_3$) δ 8.41 (s, 1H), 7.65 (dd, *J* = 7.5, 2.1 Hz, 2H), 7.42 – 7.34 (m, 4H), 3.95 (s, 3H), 3.91 (s, 3H).**^{13}C NMR** (101 MHz, $CDCl_3$) δ 161.56, 159.40, 144.31, 136.43, 133.60, 129.63, 128.56, 127.84, 114.83, 70.85, 52.71, 52.36. **HRMS** (ESI-TOF) calcd for $C_{14}H_{13}ClN_2O_4^+$ $[M+H]^+$ 309.0642, found 309.00621.

VIB: Effect of Removing N-H bond

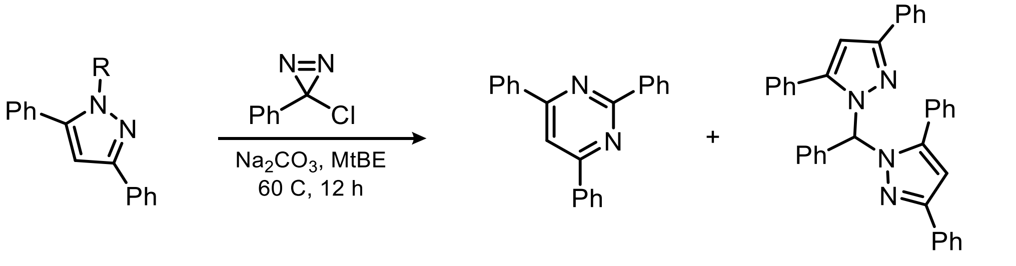

2p	3p	S-10
R = H	R = H, 25%	R = H, 70%
R = SEM	R = SEM, 68%	R = SEM, 0%

To an oven dried 2-dram screw cap vial equipped with a stir bar and PTFE/white silicone septum, **2ae** (0.1 mmol), diazirine (0.3 mmol) and Na_2CO_3 (0.3 mmol) were added, followed by a brief nitrogen sparge. Dry M*t*BE (1 mL) was then added, and the vial was sealed with electric tape. The mixture was then allowed to stir at 60 °C for 12 h

and then allowed to cool to 25 °C. The reaction mixture was quenched by the addition of water (1mL), and the two phases were separated. The aqueous layer was extracted with EtOAc (3×3mL), and the combined organic layers were dried over anhydrous Na_2SO_4, filtered, and concentrated under reduced pressure. The obtained residue was purified by preparatory TLC to afford pyrimidine **3q** and **S-10.**

1,1'-(phenylmethylene)bis(3,5-diphenyl-1H-pyrazole) (S-10):

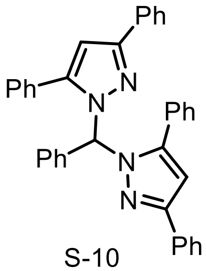

S-10

^{1}H NMR (400 MHz, $CDCl_3$) δ 7.90 – 7.83 (m, 4H), 7.72 (d, *J* = 0.9 Hz, 1H), 7.43 – 7.35 (m, 4H), 7.33 – 7.28 (m, 6H), 7.27 – 7.24 (m, 3H), 7.23 – 7.17 (m, 4H), 7.16 – 7.10 (m, 4H), 6.61 (s, 2H). **^{13}C NMR** (101 MHz, $CDCl_3$) δ 151.48, 146.14, 136.90, 133.40, 130.05, 128.98, 128.59, 128.49, 128.44, 128.44 128.43, 127.86, 127.76, 126.02, 104.54, 72.65. **HRMS** (ESI-TOF) calcd for $C_{37}H_{28}N_4O^+$ $[M+H]^+$ 529.2354, found 529.2387.

VII: Additional Data and Limitations

Fig. S1. Effect of Diazirine Loading on Yield and Conversion

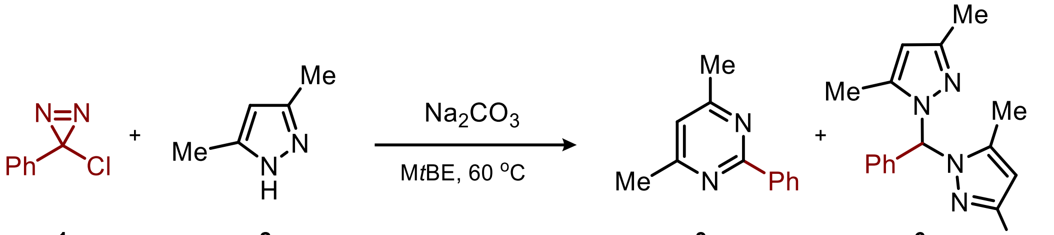

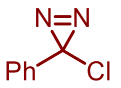

	2a		3a	6
1 eq.	8%		25%	66%
2 eq.	N.D		63%	36%
3 eq.	N.D		87%	8%

Fig. S2. Low yielding substrates

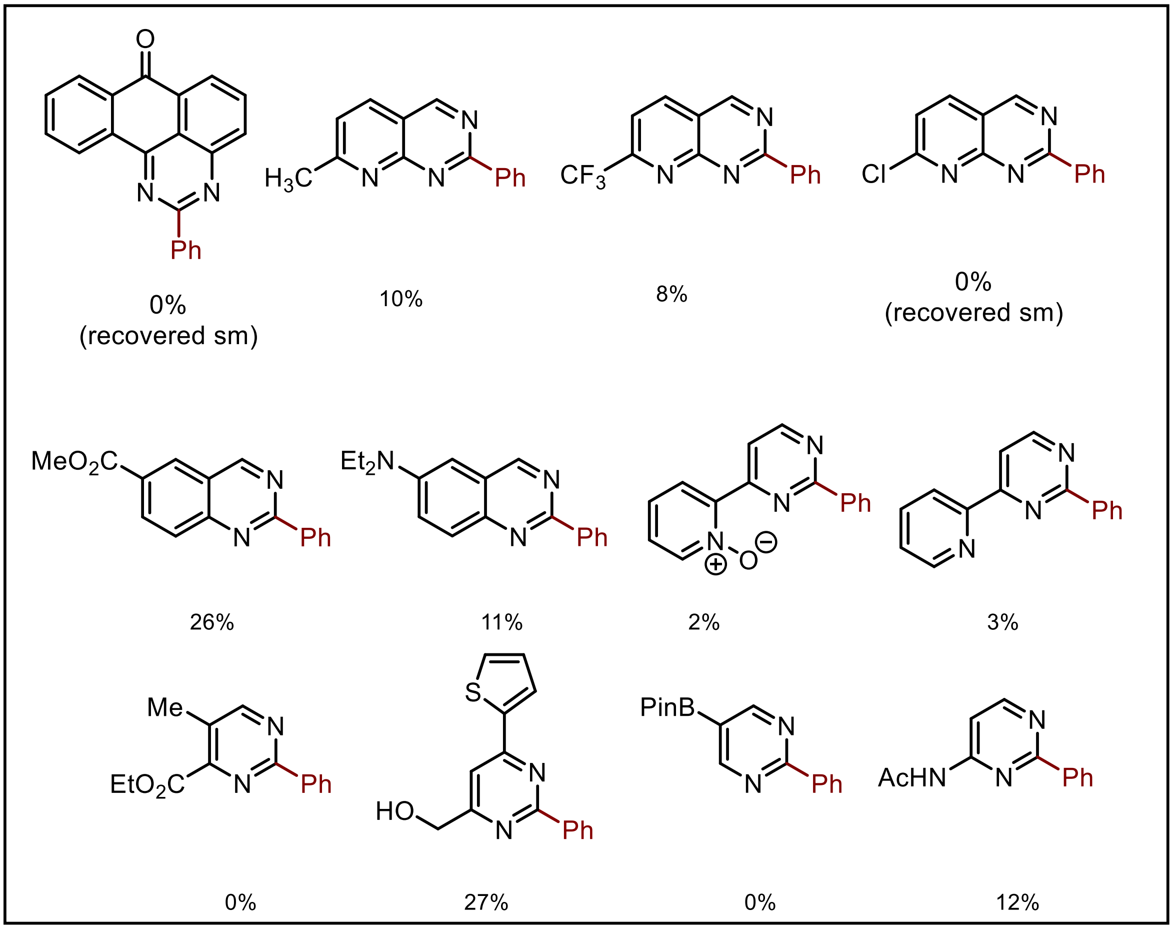

VIII: Computational Methods

Ground and transition state geometries were optimized with frequency calculations using density functional theory (DFT) in Gaussian16 (Revision A.03)[24] at the B3LYP-D3/6-31g(d) level of theory. A polarizable continuum solvation model (PCM) in diethylether (Et_2O) was used to approximate ethereal solvent MTBE. Single point energy calculations for all structures were conducted using PCM(Et_2O) at the B3LYP-D3/6-311+g(d,p) level. All of the optimized geometries were verified by frequency calculations as ground states (zero imaginary frequencies) or transition states (one imaginary frequency). Transition states were verified by intrinsic reaction coordinate (IRC) calculations. Quasi-harmonic corrected free energies (Gtz) for each compound were computed using Grimme entropic corrections[25] and Head-Gordon enthalpic corrections[26] as implemented in Paton's GoodVibes python package.[27] Vector analysis was visualized using Blender.[28] For $NICS_0$ calculations, positions of the ghost atom were calculated as the average of the 6 atoms forming the pyrimidine ring. This atom was offset by one angstrom along the unit vector orthogonal to the plane defined by C4, C5, and C6 for $NICS_1$ calculations.

Some dihydropyrimidine starting points (C-Cl bond length < 2.1Å) were generated by performing a conformational search with Crest.[29] The rest (C-Cl bond length > 2.1Å) were extracted from the IRC leading away from TS3. For each structure, the C-Cl bond length was fixed before DFT optimization.

1. Supplemental figures

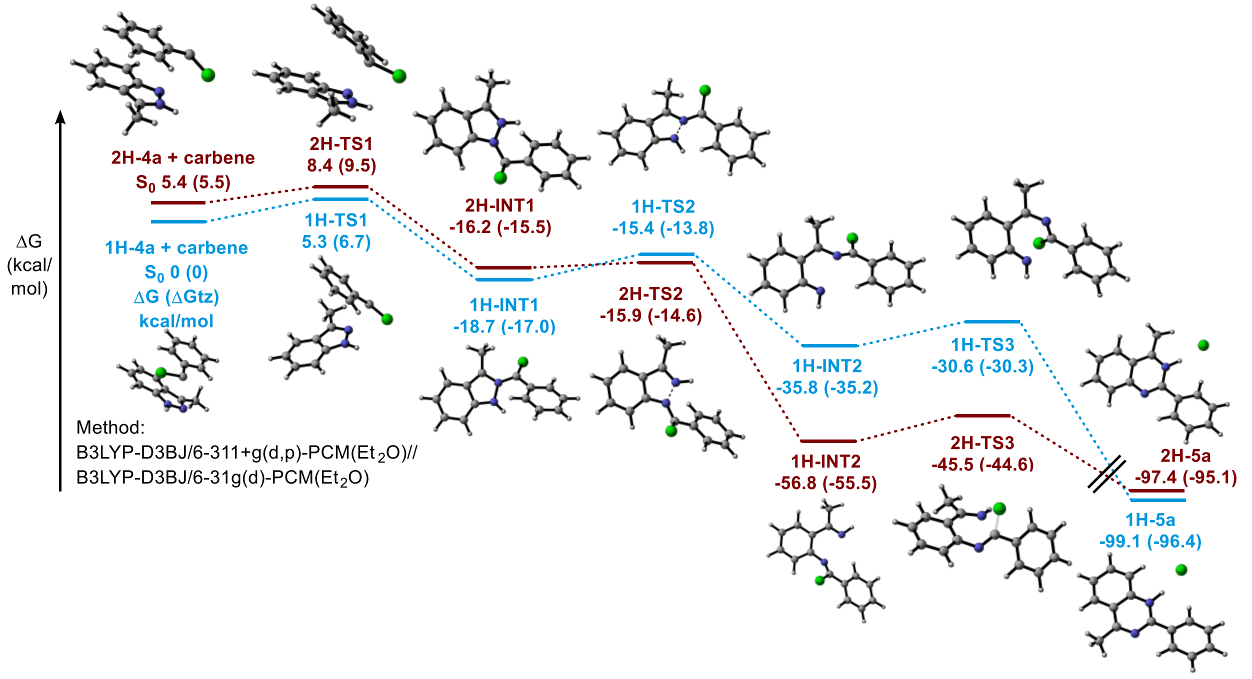

Fig. S1: calculated energy pathways for 1H- and 2H-indazole.

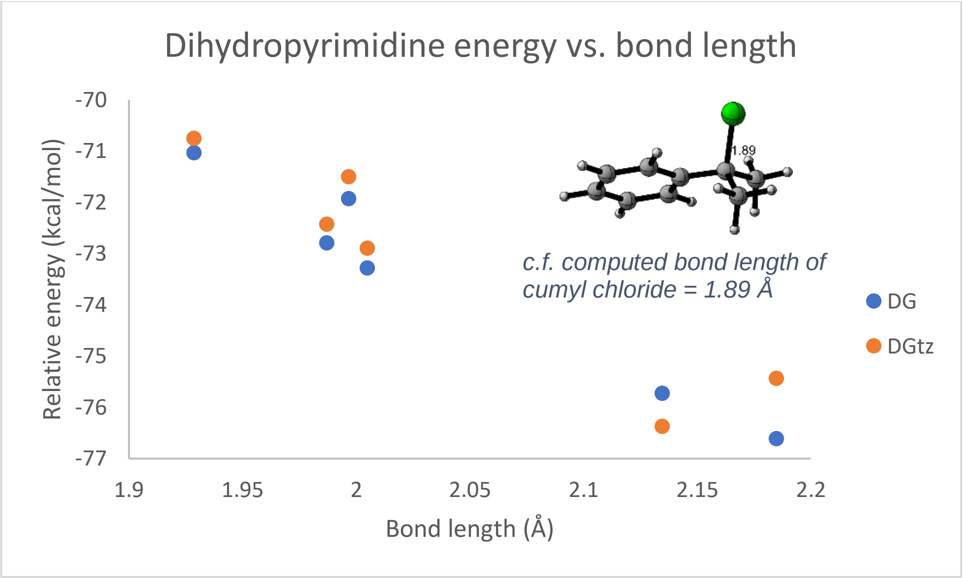

Fig. S2: energy vs. bond length in dihydropyrimidine INT3.

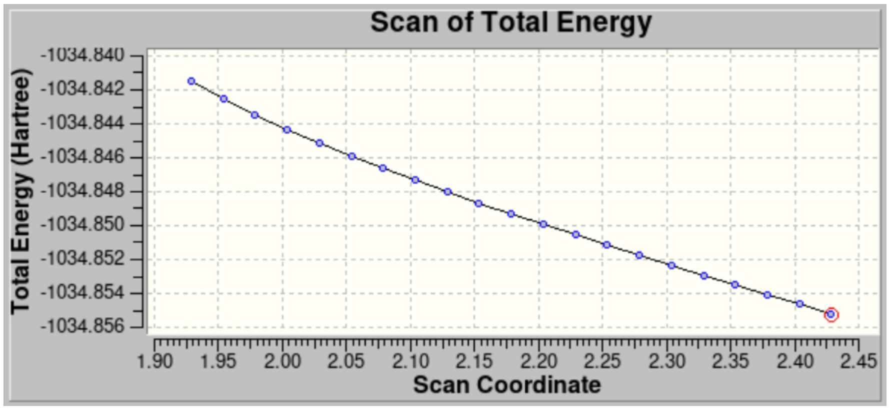

Fig S3: energy vs. bond length from relaxed coordinate scan of C-Cl bond from dihydropyrimidine

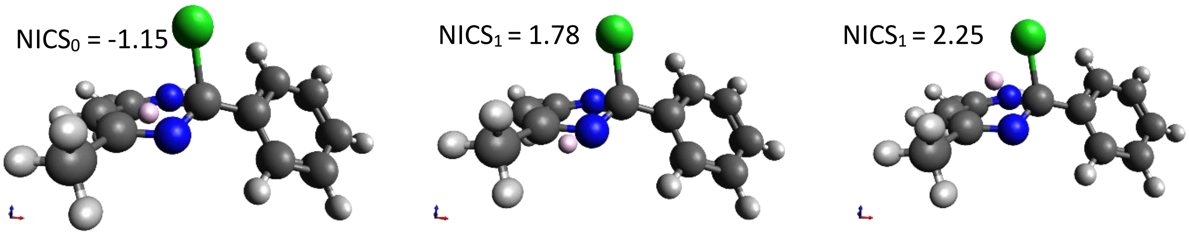

Fig. S4: Computed NICS values for cyclization transition state. c.f. pyrimidine-HCl: $NICS_0$ = -3.86, $NICS_1$ = -7.22 (avg.)

2. DFT optimized coordinates for 3,5-dimethylpyrazole

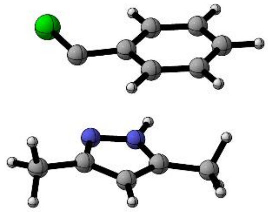

2a-carbene

C	-1.68444	-1.61827	0.13815
C	-0.56719	-2.41908	0.47387
C	0.39205	-2.15167	-0.49128
N	-0.18455	-1.24918	-1.32865
H	0.23887	-0.79889	-2.12675
C	-2.98156	-1.47545	0.87285
H	-3.30172	-2.42948	1.30502
H	-2.87805	-0.74383	1.68244
H	-3.76379	-1.11391	0.19893
Cl	-1.88567	2.56141	-0.39807
C	0.31459	1.29218	0.49060
C	0.95879	0.47705	1.45189
C	1.09047	1.82029	-0.57316
C	2.32580	0.21870	1.37539
H	0.35532	0.06480	2.25305
C	2.44763	1.55248	-0.65488
H	0.61126	2.43914	-1.32449
C	3.06838	0.75606	0.32296
H	2.80808	-0.40218	2.12419
H	3.03651	1.96178	-1.47075
H	4.13497	0.55850	0.25683
C	-1.10111	1.46474	0.76515
N	-1.44329	-0.90164	-0.96784
C	1.77845	-2.66865	-0.69398
H	2.51481	-1.85833	-0.62965
H	-0.46785	-3.10111	1.30685
H	2.01972	-3.40678	0.07508
H	1.89201	-3.14827	-1.67365

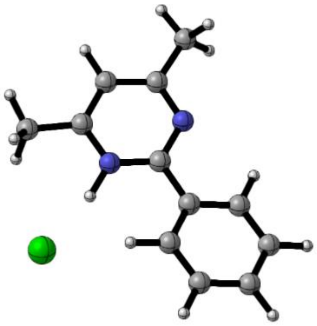

3aHCl

N	0.89607	0.57764	0.03294
H	0.41925	1.53140	-0.05273
N	0.81668	-1.76512	0.01126
C	2.15790	-1.79755	0.02983
C	2.91512	-0.62079	0.08493
C	2.25264	0.59627	0.07294
C	-1.28039	-0.57375	0.03609
C	-1.96310	-1.75174	-0.32449
C	-2.01635	0.56208	0.41717
C	-3.35307	-1.78588	-0.31673
H	-1.39170	-2.62660	-0.61199
C	-3.40889	0.51610	0.42673
H	-1.51851	1.48239	0.70147
C	-4.08025	-0.65190	0.05933
H	-3.87061	-2.69603	-0.60507
H	-3.96771	1.39839	0.72342
H	-5.16624	-0.68029	0.06666
C	2.79571	-3.15447	0.00018
H	3.88536	-3.09306	-0.04065
H	2.50291	-3.71811	0.89340
H	2.43177	-3.71518	-0.86743
C	0.19231	-0.58986	0.02524
Cl	-0.09122	3.42675	-0.26500
C	2.94322	1.92204	0.08827
H	2.60492	2.51928	0.94043
H	3.99726	-0.64977	0.11840
H	4.02498	1.78523	0.13813
H	2.68091	2.49654	-0.80542

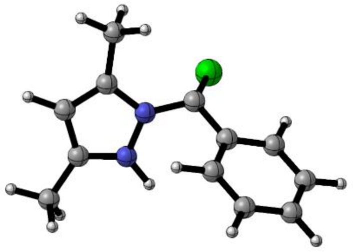

INT1

C	-2.16413	0.50870	0.78320
C	-3.10013	-0.45152	0.38701
C	-2.45173	-1.33482	-0.47479
N	-1.20063	-0.87054	-0.63738
H	-0.40097	-1.29782	-1.08784
C	-2.30109	1.68047	1.68692
H	-2.43983	2.59974	1.10582
H	-3.15979	1.55305	2.35081
H	-1.38043	1.80511	2.26557

Cl	0.07318	2.36607	-1.00155
C	1.44033	0.21562	0.17522
C	1.51127	-1.03386	0.85642
C	2.63615	0.66746	-0.43650
C	2.69002	-1.77052	0.90805
H	0.63060	-1.41081	1.37124
C	3.81221	-0.07636	-0.36957
H	2.63390	1.61316	-0.96729
C	3.85845	-1.30532	0.29454
H	2.69577	-2.71934	1.44072
H	4.70584	0.31042	-0.85520
H	4.77797	-1.88196	0.33828
C	0.21005	0.98229	0.20655
N	-0.98242	0.23888	0.17589
C	-2.93018	-2.59623	-1.11487
H	-2.44971	-3.47062	-0.66056
H	-4.12707	-0.51822	0.71306
H	-4.01025	-2.69191	-0.98422
H	-2.70613	-2.60783	-2.18669

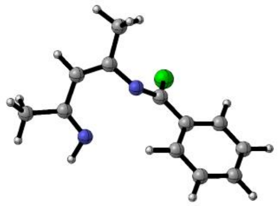

INT2

C	-0.33913	-0.67220	0.04842
Cl	-0.07508	-1.91070	-1.27350
C	-1.68468	-0.06677	0.07835
C	-2.83839	-0.75512	-0.32239
C	-1.78637	1.25543	0.54539
C	-4.08364	-0.13439	-0.23595
H	-2.76225	-1.77248	-0.69058
C	-3.03276	1.87246	0.61663
H	-0.87823	1.78247	0.81633
C	-4.18386	1.17832	0.23201
H	-4.97567	-0.67613	-0.53679
H	-3.10596	2.89826	0.96657
H	-5.15535	1.66133	0.29034
C	1.90394	-0.78468	0.84546
N	0.58763	-0.33618	0.81871
C	2.92778	-0.04947	0.35518
C	2.84764	1.25963	-0.31022
N	1.71573	1.82350	-0.55497
H	1.87423	2.73344	-1.00039
C	2.09991	-2.09977	1.55584

H	3.15736	-2.37209	1.59851
H	1.70700	-2.04297	2.57799
H	1.54885	-2.89398	1.03847
C	4.19757	1.86250	-0.65934
H	4.08761	2.83029	-1.15779
H	3.92427	-0.46368	0.48070
H	4.80436	2.00029	0.24446
H	4.75850	1.19018	-1.32078

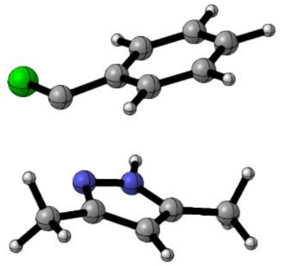

TS1

C	-2.27339	-0.03178	0.39232
C	-2.21696	-1.42417	0.20382
C	-1.32972	-1.63363	-0.85030
N	-0.94842	-0.40040	-1.25162
H	-0.24327	-0.16831	-1.93715
C	-3.01447	0.75463	1.42442
H	-3.43067	0.09445	2.19070
H	-2.32815	1.46997	1.89044
H	-3.83862	1.32332	0.97810
Cl	0.44704	3.00541	-0.68191
C	1.09741	0.63747	0.49760
C	0.88947	-0.36933	1.46575
C	2.17898	0.48659	-0.39727
C	1.70912	-1.49384	1.52428
H	0.06280	-0.25197	2.15897
C	3.00668	-0.62981	-0.33020
H	2.36311	1.25049	-1.14556
C	2.76884	-1.62811	0.62316
H	1.52618	-2.26156	2.27078
H	3.83756	-0.72994	-1.02345
H	3.41477	-2.50084	0.66807
C	0.10776	1.73610	0.57239
N	-1.48170	0.59566	-0.50030
C	-0.79758	-2.88282	-1.47123
H	0.24154	-3.05171	-1.15976
H	-2.74441	-2.18551	0.76087
H	-1.39122	-3.74528	-1.15905
H	-0.81524	-2.82968	-2.56539

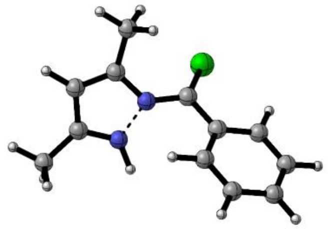

TS2

C	-2.06808	0.61900	0.74916
C	-3.14631	-0.17944	0.38351
C	-2.69585	-1.19991	-0.46510
N	-1.44349	-0.94388	-0.83780
H	-0.78464	-1.67041	-1.10729
C	-2.06559	1.78149	1.68283
H	-1.98894	2.71930	1.12006
H	-2.98268	1.80006	2.27706
H	-1.19037	1.73211	2.33909
Cl	0.20734	2.38387	-0.91817
C	1.51030	0.10794	0.09974
C	1.53686	-1.17518	0.70610
C	2.73225	0.60681	-0.40343
C	2.71414	-1.91054	0.78965
H	0.62099	-1.58411	1.12362
C	3.90782	-0.13675	-0.30911
H	2.75570	1.58438	-0.87216
C	3.91522	-1.40206	0.28195
H	2.69428	-2.89079	1.26077
H	4.82892	0.28069	-0.70948
H	4.83365	-1.97822	0.34972
C	0.26247	0.84632	0.05415
N	-0.90386	0.22174	0.17866
C	-3.42286	-2.45902	-0.83249
H	-4.46644	-2.40508	-0.51239
H	-4.14351	-0.09526	0.79037
H	-3.39615	-2.63135	-1.91338
H	-2.95631	-3.32398	-0.34487

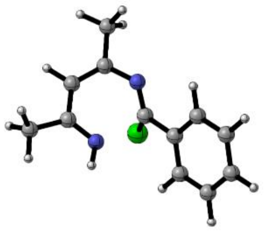

TS3

C	0.00928	-0.40098	0.35491
Cl	-0.37355	-0.09565	2.18448

C	1.44480	-0.20030	0.03608
C	2.20763	0.80488	0.64519
C	2.03439	-1.02075	-0.93793
C	3.54185	0.98602	0.28591
H	1.75021	1.44478	1.39154
C	3.37316	-0.84460	-1.28379
H	1.43520	-1.79250	-1.40832
C	4.13028	0.15967	-0.67547
H	4.12359	1.77163	0.75972
H	3.82430	-1.49219	-2.03042
H	5.17281	0.29791	-0.94849
C	-2.10412	-1.16250	-0.29396
N	-0.73545	-1.21955	-0.28190
C	-2.77144	0.03153	-0.36620
C	-2.11254	1.31582	-0.52826
N	-0.86203	1.46243	-0.23028
H	-0.45092	2.35680	-0.50135
C	-2.80682	-2.48922	-0.36654
H	-2.40242	-3.08291	-1.19491
H	-2.62600	-3.05908	0.55330
H	-3.88516	-2.37346	-0.50265
C	-2.95110	2.43705	-1.10516
H	-3.26323	2.19364	-2.12764
H	-3.84780	0.01117	-0.50248
H	-3.86297	2.56449	-0.50983
H	-2.40706	3.38581	-1.11804

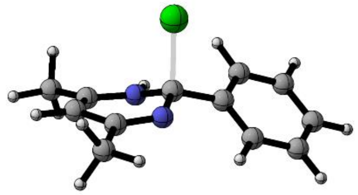

INT3_1p93A

Cl	-0.33648	-0.54905	-2.10042
C	-0.15170	0.00372	-0.26187
C	1.34698	0.07921	-0.00370
C	2.16487	-1.04107	-0.20630
C	3.52221	-0.98960	0.10651
C	4.08032	0.18677	0.61523
C	3.27083	1.30699	0.80792
C	1.90835	1.25368	0.50436
N	-0.74456	-1.00938	0.54466
C	-2.09767	-1.05868	0.72497
C	-2.66621	-2.33001	1.27813

C	-2.81726	0.06537	0.43392
C	-2.08345	1.25355	0.09870
C	-2.80178	2.57747	0.10014
N	-0.80572	1.25558	-0.17001
H	1.74683	-1.94570	-0.63999
H	4.14593	-1.86382	-0.05829
H	5.13958	0.22933	0.85352
H	3.69726	2.22725	1.19795
H	1.27037	2.11688	0.65350
H	-0.19110	-1.83204	0.74800
H	-3.73699	-2.22745	1.46552
H	-2.17175	-2.59717	2.22016
H	-2.51047	-3.15833	0.57640
H	-3.88853	0.09372	0.59062
H	-2.10337	3.38935	-0.11269
H	-3.28915	2.75817	1.06553
H	-3.58955	2.57578	-0.66378

INT3_1p98A

Cl	0.33161	-0.54070	2.11943
C	0.15023	0.01542	0.22046
C	-1.34740	0.07949	-0.01751
C	-1.92572	1.25918	-0.49416
C	-3.29233	1.30580	-0.77955
C	-4.08893	0.17445	-0.59918
C	-3.51402	-1.00663	-0.12044
C	-2.15271	-1.05201	0.17407
N	0.75163	-1.00798	-0.55240
C	2.10624	-1.05959	-0.71802
C	2.68154	-2.33731	-1.24822
C	2.82179	0.06877	-0.43093
C	2.08573	1.25785	-0.11205
C	2.80273	2.58183	-0.10536
N	0.80283	1.25859	0.13972
H	-1.29764	2.13115	-0.63418
H	-3.73197	2.22958	-1.14575
H	-5.15149	0.21190	-0.82307
H	-4.12814	-1.88904	0.03618
H	-1.72216	-1.95937	0.58937
H	0.20009	-1.83210	-0.75583
H	2.52369	-3.15458	-0.53419
H	3.75321	-2.23435	-1.42979
H	2.19430	-2.62058	-2.18918
H	3.89503	0.09502	-0.57391
H	3.31516	2.75573	-1.05883
H	3.56989	2.58624	0.67925

H	2.09849	3.39446	0.08375

INT3_1p99A

Cl	0.29671	0.36673	2.17542
C	0.15244	0.04824	0.20963
C	-1.33872	-0.06492	-0.05098
C	-1.88958	-1.17782	-0.69358
C	-3.26167	-1.23414	-0.95917
C	-4.08962	-0.17219	-0.60105
C	-3.53781	0.95411	0.01992
C	-2.17421	1.00748	0.29111
N	0.85918	-1.15602	-0.01479
C	2.21501	-1.15465	-0.20007
C	2.91410	-2.47402	-0.08011
C	2.81714	0.02889	-0.51474
C	1.98079	1.18134	-0.68781
C	2.57818	2.43769	-1.26311
N	0.70626	1.19304	-0.39492
H	-1.25742	-1.99755	-1.01987
H	-3.67474	-2.10762	-1.45602
H	-5.15518	-0.21475	-0.80903
H	-4.17352	1.79123	0.29481
H	-1.74242	1.87594	0.77573
H	0.43715	-2.00161	0.34921
H	2.84501	-2.85172	0.94749
H	3.96888	-2.37973	-0.34564
H	2.45211	-3.21919	-0.73894
H	3.88002	0.07259	-0.71812
H	1.81328	3.20950	-1.36859
H	3.03451	2.24024	-2.24045
H	3.37369	2.80913	-0.60494

INT3_2p00A

Cl	0.32975	-0.53819	2.12492
C	0.14978	0.01892	0.20733
C	-1.34744	0.07937	-0.02454
C	-2.14881	-1.05570	0.16288
C	-3.51137	-1.01210	-0.12554
C	-4.09158	0.17063	-0.59383
C	-3.29901	1.30549	-0.76973
C	-1.93111	1.26084	-0.49040
N	0.75408	-1.00760	-0.55490
C	2.10914	-1.05973	-0.71567
C	2.68684	-2.33967	-1.23758
C	2.82321	0.07020	-0.43015

C	2.08630	1.25942	-0.11635
C	2.80264	2.58353	-0.10709
N	0.80186	1.25955	0.13026
H	-1.71437	-1.96418	0.57162
H	-4.12246	-1.89714	0.02800
H	-5.15519	0.20661	-0.81288
H	-3.74281	2.23049	-1.12768
H	-1.30615	2.13559	-0.62704
H	0.20332	-1.83237	-0.75807
H	3.75834	-2.23582	-1.41951
H	2.20038	-2.63024	-2.17667
H	2.53051	-3.15223	-0.51785
H	3.89703	0.09598	-0.56875
H	3.56326	2.59000	0.68385
H	2.09658	3.39626	0.07459
H	3.32280	2.75542	-1.05670

INT3_2p13A

C	0.14609	0.04303	0.11560
Cl	0.32136	-0.54426	2.16028
C	-1.34781	0.08170	-0.07112
C	-2.12653	-1.07263	0.09604
C	-1.96272	1.27210	-0.47182
C	-3.49682	-1.03924	-0.15097
H	-1.66927	-1.98632	0.46602
C	-3.33839	1.30530	-0.71004
H	-1.35579	2.16185	-0.59235
C	-4.10787	0.15155	-0.55630
H	-4.09056	-1.93823	-0.01180
H	-3.80641	2.23607	-1.01850
H	-5.17793	0.17885	-0.74248
C	2.09057	1.27055	-0.13633
N	0.79577	1.26618	0.07679
C	2.83073	0.08287	-0.42780
C	2.12513	-1.05519	-0.71013
N	0.76814	-0.99992	-0.58035
H	0.22256	-1.82903	-0.78191
C	2.80340	2.59453	-0.09545
H	2.08664	3.40754	0.03519
H	3.51104	2.60866	0.74318
H	3.38315	2.75845	-1.01105
C	2.71601	-2.34616	-1.18631
H	2.23905	-2.67254	-2.11828
H	3.90788	0.10583	-0.53838
H	3.78799	-2.23968	-1.36286
H	2.56041	-3.13331	-0.43864

INT3_2p18A

C	0.14439	0.05196	0.08174
Cl	0.32032	-0.55278	2.17391
C	-1.34805	0.08344	-0.08750
C	-2.12000	-1.07634	0.07487
C	-1.97253	1.27601	-0.46771
C	-3.49273	-1.04602	-0.15721
H	-1.65576	-1.99137	0.43233
C	-3.35058	1.30566	-0.69129
H	-1.37106	2.16995	-0.58469
C	-4.11313	0.14663	-0.54271
H	-4.08126	-1.94886	-0.02116
H	-3.82591	2.23766	-0.98429
H	-5.18520	0.17120	-0.71717
C	2.09196	1.27469	-0.14133
N	0.79349	1.26863	0.06004
C	2.83250	0.08838	-0.42885
C	2.12955	-1.05214	-0.71137
N	0.77236	-0.99643	-0.59085
H	0.22875	-1.82758	-0.79024
C	2.80370	2.59809	-0.08410
H	2.08395	3.41161	0.02480
H	3.48646	2.61368	0.77492
H	3.40970	2.75955	-0.98282
C	2.72449	-2.34538	-1.17542
H	2.25319	-2.67902	-2.10763
H	3.91051	0.11059	-0.53080
H	3.79730	-2.23868	-1.34638
H	2.56557	-3.12675	-0.42246

3. DFT optimized coordinates for 1H and 2H indazole

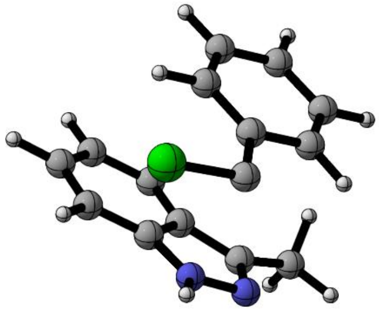

4a-1H_carbene

C	1.84469	0.05977	-1.38519
C	1.49312	0.88627	-0.26550
C	0.25212	1.46939	-0.62013

N	-0.04171	0.97970	-1.86269
H	-0.87960	1.11460	-2.40804
C	3.06147	-0.79388	-1.54053
H	3.12279	-1.52882	-0.72866
H	3.03195	-1.32928	-2.49319
H	3.97756	-0.19171	-1.50678
Cl	-3.30306	0.33578	-0.49469
C	-1.23591	-1.29954	0.05126
C	-0.26695	-2.24724	-0.36804
C	-1.19123	-0.83416	1.39273
C	0.70085	-2.71878	0.51381
H	-0.29865	-2.58312	-1.39889
C	-0.22251	-1.30070	2.26430
H	-1.91497	-0.09974	1.72597
C	0.72349	-2.24167	1.82618
H	1.43657	-3.44502	0.18229
H	-0.18511	-0.93269	3.28497
H	1.48165	-2.60035	2.51722
C	-2.12631	-0.91427	-1.01812
N	0.91478	0.12498	-2.32587
C	2.06949	1.17357	0.98258
H	3.02045	0.73269	1.26816
C	-0.44162	2.33179	0.24228
H	-1.39721	2.76320	-0.03531
C	1.39006	2.02605	1.84147
C	0.14800	2.59384	1.47237
H	1.81157	2.26271	2.81420
H	-0.35908	3.25394	2.17079

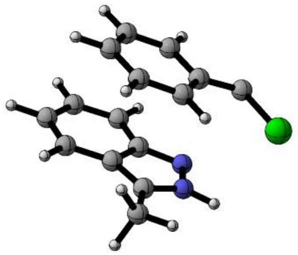

4a-2H_carbene

C	-1.01071	-1.12624	-0.99639
C	-1.23989	-1.11444	0.42021
C	-0.07970	-1.68332	0.97482
N	0.69622	-1.98377	-0.08856
H	1.63485	-2.36036	-0.04799
Cl	3.50072	-0.23139	-0.58372
C	1.24975	1.24053	-0.37827
C	0.15470	1.91866	-0.97543
C	1.32018	1.19718	1.03923
C	-0.82684	2.52008	-0.19892

H	0.10204	1.93874	-2.05893
C	0.34623	1.81103	1.81142
H	2.14976	0.68586	1.51526
C	-0.73215	2.46263	1.19434
H	-1.66931	3.01653	-0.66914
H	0.40960	1.77859	2.89539
H	-1.49900	2.92897	1.80659
C	2.14274	0.66858	-1.35878
N	0.19446	-1.66047	-1.30115
C	0.31633	-1.95598	2.38714
H	-0.26023	-2.78592	2.81319
H	1.37886	-2.20911	2.45941
H	0.13540	-1.07237	3.00832
C	-2.43083	-0.57180	0.95354
H	-2.60493	-0.55887	2.02595
C	-1.97684	-0.58714	-1.88056
H	-1.79895	-0.58607	-2.95159
C	-3.13096	-0.06547	-1.33366
C	-3.36038	-0.05708	0.07317
H	-4.28562	0.36826	0.45186
H	-3.88923	0.35580	-1.98841

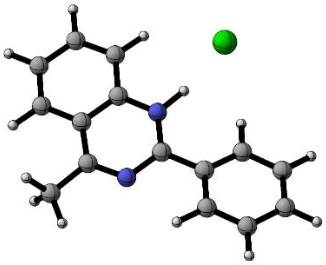

5a-1HCl

N	0.26796	0.50688	-0.00210
H	-0.14183	1.48604	-0.08302
N	0.04740	-1.82971	-0.01889
C	1.36136	-1.97745	-0.01171
C	2.24503	-0.85192	0.04000
C	1.64368	0.43337	0.02731
C	-1.96589	-0.49658	0.04092
C	-2.72256	-1.63663	-0.29541
C	-2.62868	0.68604	0.41869
C	-4.11204	-1.58701	-0.27006
H	-2.20842	-2.54730	-0.57860
C	-4.02022	0.72342	0.44588
H	-2.07191	1.57689	0.68764
C	-4.76483	-0.40727	0.10058
H	-4.68693	-2.46790	-0.53981
H	-4.52204	1.64015	0.73988
H	-5.85036	-0.37061	0.12252

C	1.88885	-3.38164	-0.05784
H	2.54577	-3.52034	-0.92412
H	2.48135	-3.60096	0.83825
H	1.05834	-4.08588	-0.11789
C	-0.49962	-0.59300	0.00503
Cl	-0.54199	3.44203	-0.28620
C	3.65654	-0.94475	0.08102
H	4.13112	-1.91937	0.09454
C	2.42822	1.60362	0.04332
H	1.93739	2.57092	0.02398
C	4.42186	0.20295	0.10315
C	3.80323	1.47403	0.08190
H	5.50441	0.13316	0.13499
H	4.41981	2.36748	0.09736

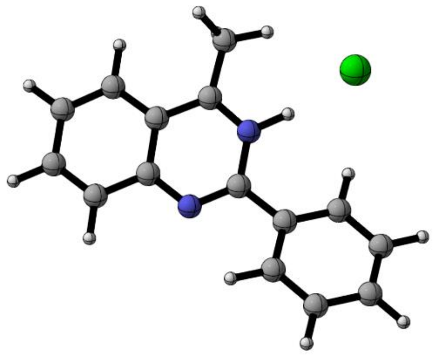

5a-2HCl

N	0.05456	0.86502	0.03377
H	-0.68498	1.63084	-0.06547
N	0.58335	-1.42621	-0.00562
C	1.33111	1.26189	0.05964
C	2.33896	0.26611	0.06285
C	1.90351	-1.09402	0.00155
C	-1.74680	-0.82626	0.04929
C	-2.13294	-2.08356	-0.45171
C	-2.71958	0.02988	0.59079
C	-3.46907	-2.46778	-0.42456
H	-1.37711	-2.74285	-0.86373
C	-4.05608	-0.36589	0.61963
H	-2.44818	1.00182	0.98506
C	-4.43498	-1.60945	0.11152
H	-3.75933	-3.43506	-0.82423
H	-4.80001	0.30358	1.04056
H	-5.47857	-1.91070	0.13217
C	1.63278	2.72743	0.07518
H	2.16690	2.97890	0.99862
H	2.28918	2.98051	-0.76386

H	0.72032	3.32205	0.01075
C	-0.31551	-0.47355	0.02348
Cl	-1.82460	3.24098	-0.35277
C	3.72645	0.56149	0.09747
H	4.06041	1.59175	0.14892
C	2.86908	-2.12829	-0.03612
H	2.52052	-3.15427	-0.08389
C	4.64352	-0.46503	0.06436
C	4.21073	-1.81347	-0.00554
H	5.70574	-0.24451	0.09080
H	4.95089	-2.60755	-0.03266

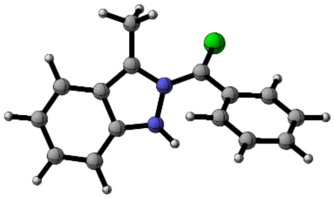

INT1-1H

C	-1.35785	1.08759	0.51638
C	-2.47352	0.27653	0.19134
C	-1.96398	-0.87696	-0.46346
N	-0.62813	-0.67064	-0.66923
H	0.08429	-1.37975	-0.79030
C	-1.27874	2.33988	1.30558
H	-1.18718	3.22378	0.66297
H	-2.17050	2.45055	1.92846
H	-0.37956	2.31281	1.93454
Cl	1.28798	2.50245	-0.79123
C	2.15233	0.00019	0.12486
C	1.99850	-1.20853	0.86253
C	3.41315	0.22051	-0.48143
C	3.02485	-2.14324	0.94494
H	1.07111	-1.39137	1.40050
C	4.43944	-0.71477	-0.37247
H	3.58060	1.13305	-1.04297
C	4.25920	-1.91085	0.32797
H	2.86465	-3.05463	1.51651
H	5.39244	-0.50781	-0.85417
H	5.06252	-2.63788	0.40302
C	1.06468	0.94625	0.07153
N	-0.23863	0.49290	0.03730
C	-2.78112	-1.95869	-0.80776
H	-2.37498	-2.84019	-1.29295
C	-4.13810	-1.83836	-0.52571
H	-4.80784	-2.64838	-0.79912
C	-3.85209	0.37269	0.46395

H	-4.25811	1.25472	0.94937
C	-4.66859	-0.68786	0.10132
H	-5.73468	-0.63851	0.30111

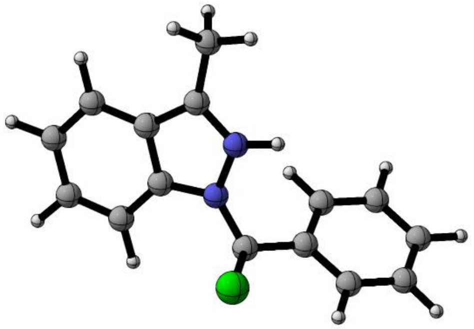

INT1-2H

C	-1.60205	-0.40660	-0.34211
C	-2.46981	0.62756	0.09680
C	-1.67345	1.52442	0.85407
N	-0.44764	1.01222	0.87494
H	0.40102	1.39598	1.27553
Cl	0.81560	-2.06125	1.57998
C	2.04730	-0.28674	-0.21783
C	2.04774	0.85518	-1.06777
C	3.31158	-0.72667	0.24621
C	3.22878	1.49943	-1.42340
H	1.10583	1.22105	-1.46919
C	4.48695	-0.07404	-0.11838
H	3.36242	-1.59040	0.90016
C	4.46611	1.04760	-0.95259
H	3.17964	2.36770	-2.07755
H	5.43501	-0.44698	0.26392
H	5.38640	1.55365	-1.23032
C	0.79867	-0.97815	0.06028
N	-0.33730	-0.16455	0.11317
C	-2.03776	2.81819	1.49957
H	-2.31091	3.55799	0.73872
H	-2.90482	2.67866	2.15307
H	-1.21285	3.21811	2.09450
C	-2.05789	-1.47459	-1.13204
H	-1.36715	-2.24816	-1.44612
C	-3.83522	0.61021	-0.26472
H	-4.50585	1.39813	0.06357
C	-3.39816	-1.46898	-1.46863
C	-4.28271	-0.43939	-1.04092
H	-5.32676	-0.48757	-1.33406
H	-3.79303	-2.27517	-2.07996

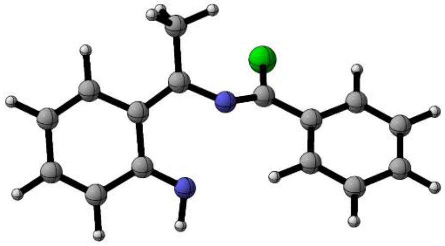

INT2-1H

C	1.09260	0.72410	-0.03121
Cl	1.09854	2.18863	-1.11982
C	2.36693	-0.00726	0.07214
C	3.61287	0.59063	-0.17081
C	2.30586	-1.36461	0.44087
C	4.78240	-0.15402	-0.02903
H	3.66721	1.63395	-0.46095
C	3.47880	-2.10340	0.56773
H	1.33320	-1.81849	0.59391
C	4.71944	-1.50039	0.33907
H	5.74385	0.31823	-0.20863
H	3.42530	-3.15337	0.84123
H	5.63329	-2.07885	0.44239
C	-1.21387	0.89271	0.61345
N	0.03536	0.34822	0.52816
C	-2.32724	0.17094	0.22112
C	-2.21630	-1.18891	-0.36908
N	-1.06370	-1.66347	-0.74663
H	-1.18711	-2.63219	-1.06278
C	-1.30048	2.25779	1.26923
H	-1.94248	2.22907	2.15556
H	-0.30975	2.59893	1.57289
H	-1.71572	2.99628	0.57466
C	-3.64846	0.72724	0.41506
H	-3.73926	1.74618	0.77532
C	-3.47057	-1.92911	-0.51265
H	-3.40085	-2.95464	-0.86945
C	-4.67865	-1.35694	-0.27977
C	-4.77636	0.00709	0.17292
H	-5.59069	-1.92717	-0.43860
H	-5.75552	0.44690	0.33646

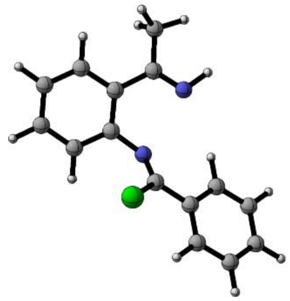

INT2-2H

C	0.87544	-0.61935	0.08417
Cl	0.67874	-1.99119	1.28394
C	2.26193	-0.14153	-0.09602
C	3.38596	-0.94429	0.14689
C	2.43924	1.18046	-0.54421
C	4.66681	-0.43727	-0.06942
H	3.25970	-1.96306	0.49547
C	3.72128	1.68314	-0.74699
H	1.56271	1.79737	-0.70689
C	4.83870	0.87532	-0.51443
H	5.53111	-1.06950	0.11236
H	3.84981	2.70841	-1.08242
H	5.83851	1.26915	-0.67489
C	-1.45528	-0.44057	-0.41428
N	-0.10707	-0.09408	-0.48777
C	-2.42931	0.51392	-0.02089
C	-2.05905	1.87688	0.45497
N	-0.88414	2.08114	0.93049
H	-0.78135	3.06717	1.19318
C	-3.12195	2.95835	0.35588
H	-3.59251	2.96983	-0.63310
H	-3.91634	2.79672	1.09455
H	-2.68213	3.94107	0.54857
C	-1.85566	-1.70911	-0.86442
H	-1.09178	-2.40978	-1.18466
C	-3.78047	0.13173	-0.08690
H	-4.54714	0.83624	0.21631
C	-3.20180	-2.05582	-0.91421
C	-4.17171	-1.13291	-0.51876
H	-5.22553	-1.39296	-0.54993
H	-3.49001	-3.04349	-1.26290

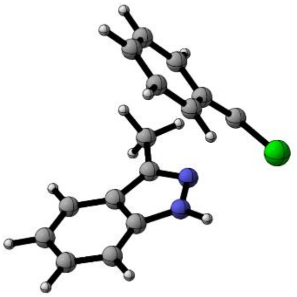

TS1-1H

C	0.89980	-0.35831	1.45931
C	2.04098	-0.00550	0.67070
C	1.90064	-0.74261	-0.53438
N	0.74619	-1.45781	-0.40019
H	0.31608	-2.11260	-1.03787
C	0.49055	0.15728	2.79925
H	0.27318	1.23155	2.74891
H	-0.40887	-0.36134	3.13871
H	1.28719	0.01571	3.53853
Cl	-2.37726	-2.47443	-0.39305
C	-2.11239	0.19509	0.06394
C	-2.26591	1.30072	0.92790
C	-1.94358	0.43890	-1.31596
C	-2.25726	2.60388	0.43523
H	-2.39578	1.10815	1.98839
C	-1.92477	1.73902	-1.80652
H	-1.82121	-0.39823	-1.99488
C	-2.08184	2.82342	-0.93307
H	-2.38399	3.44396	1.11200
H	-1.78755	1.91598	-2.86968
H	-2.06769	3.83765	-1.32290
C	-2.14638	-1.09627	0.77931
N	0.15319	-1.23333	0.80009
C	3.14871	0.84587	0.84412
H	3.26949	1.41698	1.76018
C	2.84098	-0.66166	-1.57449
H	2.72576	-1.23336	-2.49001
C	4.07537	0.93496	-0.18182
C	3.91928	0.18701	-1.37599
H	4.93861	1.58536	-0.07591
H	4.66742	0.28262	-2.15803

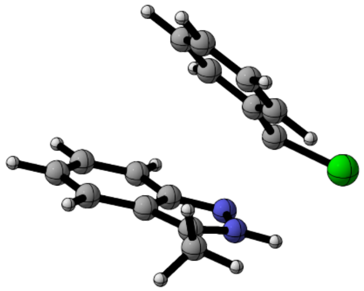

TS1-2H

C	-1.14149	-1.15512	-0.45237
C	-1.62981	-0.56465	0.75089
C	-0.62657	-0.81775	1.71538
N	0.31396	-1.51839	1.06567
H	1.20652	-1.82329	1.43709
Cl	3.05573	-1.53350	-0.57866
C	1.37544	0.62555	-0.72193
C	0.48297	1.44262	-1.44945
C	1.89178	1.12536	0.49202
C	0.11618	2.70305	-0.98464
H	0.08254	1.05826	-2.38199
C	1.52973	2.38715	0.95634
H	2.58423	0.51866	1.06659
C	0.63795	3.17704	0.22197
H	-0.57375	3.31436	-1.55939
H	1.94031	2.75810	1.89177
H	0.35538	4.16028	0.58869
C	1.60642	-0.69333	-1.36405
N	0.07759	-1.73900	-0.25355
C	-0.51954	-0.42385	3.14818
H	-1.41308	-0.72478	3.70510
H	0.35418	-0.87858	3.62377
H	-0.42551	0.66553	3.23016
C	-2.86606	0.11661	0.77098
H	-3.24554	0.55786	1.68798
C	-1.86680	-1.04790	-1.65578
H	-1.47667	-1.47536	-2.57311
C	-3.06951	-0.36802	-1.61487
C	-3.57052	0.20650	-0.41314
H	-4.52418	0.72560	-0.43631
H	-3.65364	-0.26499	-2.52516

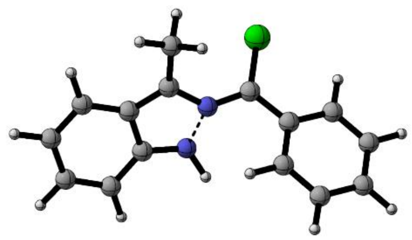

TS2-1H

C	-1.28889	0.99475	0.52823
C	-2.45666	0.29718	0.14538
C	-2.07656	-0.87181	-0.58420
N	-0.78419	-0.78606	-0.97663
H	-0.21920	-1.63486	-0.97543
C	-1.19882	2.17001	1.44040
H	-1.06297	3.09499	0.86672
H	-2.10204	2.26306	2.04829
H	-0.32125	2.06676	2.08832
Cl	1.29963	2.46907	-0.83000
C	2.20867	-0.02150	0.09762
C	2.03371	-1.26417	0.75628
C	3.49526	0.27975	-0.39583
C	3.08763	-2.16221	0.88725
H	1.06439	-1.51086	1.18017
C	4.54763	-0.62276	-0.24958
H	3.66745	1.22435	-0.89968
C	4.35621	-1.85201	0.38505
H	2.91963	-3.10799	1.39664
H	5.52680	-0.36128	-0.64317
H	5.17927	-2.55249	0.49366
C	1.07384	0.87646	-0.02496
N	-0.16440	0.43180	0.05521
C	-3.80221	0.48939	0.53082
H	-4.10152	1.39534	1.04889
C	-3.00944	-1.90851	-0.79240
H	-2.71170	-2.81955	-1.30243
C	-4.72385	-0.49895	0.24160
C	-4.32325	-1.69317	-0.40747
H	-5.76419	-0.37014	0.52414
H	-5.06795	-2.45653	-0.61524

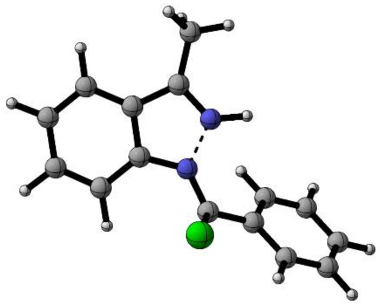

TS2-2H

C	-1.57146	-0.38934	-0.32697
C	-2.51184	0.59094	0.07879
C	-1.79564	1.63023	0.74322
N	-0.54681	1.23893	0.87145
H	0.25103	1.81091	1.12960
C	-2.31135	2.97231	1.15368
H	-2.61800	3.54701	0.27187
H	-3.19289	2.85149	1.79179
H	-1.55785	3.54178	1.70258
Cl	0.71975	-2.04231	1.49741
C	2.10910	-0.23646	-0.15955
C	2.19778	0.90664	-0.99907
C	3.32713	-0.76999	0.32163
C	3.42541	1.47281	-1.32602
H	1.28696	1.33796	-1.40548
C	4.55175	-0.19573	-0.01518
H	3.30658	-1.64221	0.96565
C	4.61997	0.93161	-0.83739
H	3.44898	2.34776	-1.97229
H	5.46546	-0.63665	0.37754
H	5.57731	1.37642	-1.09400
C	0.80912	-0.81739	0.12149
N	-0.29068	-0.05309	0.02576
C	-3.87488	0.45446	-0.25191
H	-4.59349	1.20889	0.05398
C	-1.97467	-1.52726	-1.04518
H	-1.23968	-2.26655	-1.34108
C	-4.26902	-0.66674	-0.95777
C	-3.32024	-1.64573	-1.34895
H	-5.31403	-0.80603	-1.21635
H	-3.66080	-2.51441	-1.90516

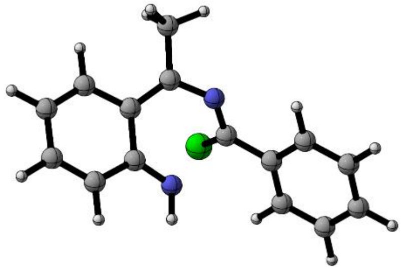

TS3-1H

C	-0.79032	0.55750	0.39733
Cl	-0.46721	0.53104	2.19318
C	-2.15080	0.13865	-0.00013
C	-2.91473	-0.74929	0.77056
C	-2.67081	0.62419	-1.21222
C	-4.17824	-1.14460	0.33552
H	-2.51366	-1.13870	1.69909
C	-3.93914	0.23570	-1.63530
H	-2.07411	1.30825	-1.80544
C	-4.69634	-0.65110	-0.86444
H	-4.75918	-1.83836	0.93632
H	-4.33690	0.62544	-2.56797
H	-5.68460	-0.95564	-1.19747
C	1.30497	1.40532	-0.28667
N	-0.03821	1.26191	-0.35505
C	2.16468	0.31249	-0.13464
C	1.67563	-1.08813	-0.14907
N	0.43023	-1.38612	0.11287
H	0.22257	-2.36947	-0.07864
C	1.80162	2.80550	-0.56258
H	0.95663	3.44769	-0.81874
H	2.28586	3.21998	0.33035
H	2.53006	2.83481	-1.37893
C	3.59002	0.52796	-0.18106
H	3.96325	1.54202	-0.08397
C	2.66678	-2.11026	-0.46040
H	2.30730	-3.13053	-0.57506
C	4.47961	-0.49286	-0.33894
C	3.99761	-1.83287	-0.51224
H	5.54739	-0.29751	-0.35729
H	4.71053	-2.63549	-0.68549

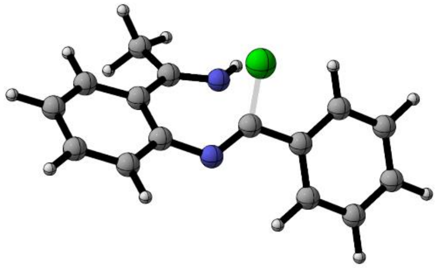

TS3-2H

C	0.60954	-0.33125	0.33123
Cl	0.30120	0.03199	2.23209
C	2.05607	-0.28401	0.00519
C	2.92058	0.65251	0.58645
C	2.55185	-1.18331	-0.95124
C	4.26370	0.68929	0.21623
H	2.53612	1.34793	1.32434
C	3.89928	-1.15214	-1.30710
H	1.87363	-1.90047	-1.40021
C	4.75829	-0.21512	-0.72750
H	4.92570	1.42196	0.66901
H	4.27718	-1.85956	-2.03993
H	5.80741	-0.18980	-1.00888
C	-1.59047	-0.88380	-0.20290
N	-0.22394	-1.07963	-0.26371
C	-2.15887	0.42193	-0.28251
C	-1.31001	1.61607	-0.47789
N	-0.05103	1.54798	-0.22100
H	0.49892	2.37832	-0.44277
C	-1.94140	2.89029	-0.99672
H	-2.61713	3.31300	-0.24437
H	-1.17579	3.63772	-1.22069
H	-2.52884	2.70055	-1.90037
C	-2.43745	-2.00489	-0.16457
H	-1.97970	-2.98870	-0.13211
C	-3.56221	0.54041	-0.28182
H	-4.01751	1.52347	-0.33767
C	-3.81740	-1.85289	-0.14439
C	-4.38734	-0.57382	-0.19462
H	-5.46590	-0.45110	-0.17588
H	-4.45468	-2.73133	-0.09019

4. Cumyl Chloride Coordinates

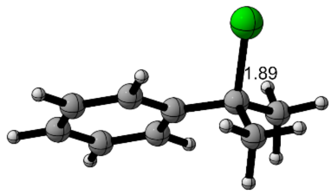

cumyl_chloride_1

C	1.14183	1.22257	-0.23210
C	0.31432	0.13920	0.08919
C	0.91266	-1.11350	0.30594
C	2.29170	-1.27719	0.20795
C	3.10821	-0.18674	-0.10785
C	2.52763	1.06096	-0.32743
H	0.72047	2.20563	-0.40829
H	0.28781	-1.97256	0.53080
H	2.72989	-2.25748	0.37441
H	4.18464	-0.31244	-0.18495
H	3.14893	1.91675	-0.57710
C	-1.19147	0.30263	0.25821
Cl	-2.03290	-0.91831	-0.91769
C	-1.73497	1.67698	-0.12703
H	-1.46795	1.94425	-1.15241
H	-2.82399	1.68340	-0.03794
H	-1.33358	2.43635	0.55376
C	-1.64295	-0.08138	1.67047
H	-2.73025	-0.00531	1.76197
H	-1.33878	-1.09751	1.93103
H	-1.17960	0.60911	2.38530

VIII: References

(1) Musolino, S. F.; Pei, Z.; Bi, L.; DiLabio, G. A.; Wulff, J. E. Structure–Function Relationships in Aryl Diazirines Reveal Optimal Design Features to Maximize C–H Insertion. *Chem. Sci.* **2021**, *12* (36), 12138–12148. https://doi.org/10.1039/D1SC03631A.

(2) Chu, X.-Q.; Cao, W.-B.; Xu, X.-P.; Ji, S.-J. Iron Catalysis for Modular Pyrimidine Synthesis through β-Ammoniation/Cyclization of Saturated Carbonyl Compounds with Amidines. *J. Org. Chem.* **2017**, *82* (2), 1145–1154. https://doi.org/10.1021/acs.joc.6b02767.

(3) Yang, B.; Wang, Z.-X. Transition-Metal-Free Cross-Coupling of Aryl and Heteroaryl Thiols with Arylzinc Reagents. *Org. Lett.* **2017**, *19* (22), 6220–6223. https://doi.org/10.1021/acs.orglett.7b03145.

(4) Rakhi, C.; Ramesh, K.; Darbem, M. P.; Branquinho, T. A.; de Oliveira, A. R.; Manjari, P. S.; Domingues, N. L. C. Novel Multi-Component Syntheses of Pyrimidines Using β-CD in Aqueous Medium. *Tetrahedron Lett.* **2016**, *57* (15), 1656–1660. https://doi.org/10.1016/j.tetlet.2016.02.106.

(5) Su, L.; Sun, K.; Pan, N.; Liu, L.; Sun, M.; Dong, J.; Zhou, Y.; Yin, S.-F. Cyclization of Ketones with Nitriles under Base: A General and Economical Synthesis of Pyrimidines. *Org. Lett.* **2018**, *20* (11), 3399–3402. https://doi.org/10.1021/acs.orglett.8b01324.

(6) Zhu, J.; Chen, P.; Lu, G.; Liu, P.; Dong, G. Ruthenium-Catalyzed Reductive Cleavage of Unstrained Aryl–Aryl Bonds: Reaction Development and Mechanistic Study. *J. Am. Chem. Soc.* **2019**, *141* (46), 18630–18640. https://doi.org/10.1021/jacs.9b11605.

(7) Fandrick, D. R.; Reinhardt, D.; Desrosiers, J.-N.; Sanyal, S.; Fandrick, K. R.; Ma, S.; Grinberg, N.; Lee, H.; Song, J. J.; Senanayake, C. H. General and Rapid Pyrimidine Condensation by Addressing the Rate Limiting Aromatization. *Org. Lett.* **2014**, *16* (11), 2834–2837. https://doi.org/10.1021/ol500886a.

(8) Guo, W.; Li, C.; Liao, J.; Ji, F.; Liu, D.; Wu, W.; Jiang, H. Transition Metal Free Intermolecular Direct Oxidative C–N Bond Formation to Polysubstituted Pyrimidines Using Molecular Oxygen as the Sole Oxidant. *J. Org. Chem.* **2016**, *81* (13), 5538–5546. https://doi.org/10.1021/acs.joc.6b00867.

(9) Anderson, E. D.; Boger, D. L. Inverse Electron Demand Diels–Alder Reactions of 1,2,3-Triazines: Pronounced Substituent Effects on Reactivity and Cycloaddition Scope. *J. Am. Chem. Soc.* **2011**, *133* (31), 12285–12292. https://doi.org/10.1021/ja204856a.

(10) Mondal, A.; Sahoo, M. K.; Subaramanian, M.; Balaraman, E. Manganese(I)-Catalyzed Sustainable Synthesis of Quinoxaline and Quinazoline Derivatives with the Liberation of Dihydrogen. *J. Org. Chem.* **2020**, *85* (11), 7181–7191. https://doi.org/10.1021/acs.joc.0c00561.

(11) Wang, X.; He, D.; Huang, Y.; Fan, Q.; Wu, W.; Jiang, H. Copper-Catalyzed Synthesis of Substituted Quinazolines from Benzonitriles and 2-Ethynylanilines via Carbon–Carbon Bond Cleavage Using Molecular Oxygen. *J. Org. Chem.* **2018**, *83* (10), 5458–5466. https://doi.org/10.1021/acs.joc.8b00378.

(12) Xu, C.; Jia, F.-C.; Zhou, Z.-W.; Zheng, S.-J.; Li, H.; Wu, A.-X. Copper-Catalyzed Multicomponent Domino Reaction of 2-Bromoaldehydes, Benzylamines, and Sodium Azide for the Assembly of Quinazoline Derivatives. *J. Org. Chem.* **2016**, *81* (7), 3000–3006. https://doi.org/10.1021/acs.joc.5b02843.

(13) Kumar, G. R. Y.; Begum, N. S. Mn(Iii)-Mediated Cascade Cyclization of 1-(Azidomethyl)-2-Isocyanoarenes with Organoboronic Acids: Construction of Quinazoline Derivatives. *New J. Chem.* **2021**, *45* (22), 9811–9817. https://doi.org/10.1039/D1NJ01115D.

(14) Han, B.; Yang, X.-L.; Wang, C.; Bai, Y.-W.; Pan, T.-C.; Chen, X.; Yu, W. CuCl/DABCO/4-HO-TEMPO-Catalyzed Aerobic Oxidative Synthesis of 2-Substituted Quinazolines and 4H-3,1-Benzoxazines. *J. Org. Chem.* **2012**, *77* (2), 1136–1142. https://doi.org/10.1021/jo2020399.

(15) Gujjarappa, R.; Vodnala, N.; Reddy, V. G.; Malakar, C. C. Niacin as a Potent Organocatalyst towards the Synthesis of Quinazolines Using Nitriles as C–N Source. *Eur. J. Org. Chem.* **2020**, *2020* (7), 803–814. https://doi.org/10.1002/ejoc.201901651.

(16) Al Mamari, H. H.; Grošelj, U.; Požgan, F.; Brodnik, H. Regioselective Ru(II)/Pd(0) Dual Catalysis: One-Pot C–H Diarylation of Five-Membered Heterocyclic Derivatives. *J. Org. Chem.* **2021**, *86* (4), 3138–3151. https://doi.org/10.1021/acs.joc.0c01983.

(17) Ma, J.-T.; Wang, L.-S.; Chai, Z.; Chen, X.-F.; Tang, B.-C.; Chen, X.-L.; He, C.; Wu, Y.-D.; Wu, A.-X. Access to 2-Arylquinazolines via Catabolism/Reconstruction of Amino Acids with the Insertion of Dimethyl Sulfoxide. *Chem. Commun.* **2021**, *57* (44), 5414–5417. https://doi.org/10.1039/D1CC00623A.

(18) Gopalaiah, K.; Saini, A.; Devi, A. Iron-Catalyzed Cascade Reaction of 2-Aminobenzyl Alcohols with Benzylamines: Synthesis of Quinazolines by Trapping of Ammonia. *Org. Biomol. Chem.* **2017**, *15* (27), 5781–5789. https://doi.org/10.1039/C7OB01159H.

(19) Dherange, B. D.; Kelly, P. Q.; Liles, J. P.; Sigman, M. S.; Levin, M. D. Carbon Atom Insertion into Pyrroles and Indoles Promoted by Chlorodiazirines. *J. Am. Chem. Soc.* **2021**, *143* (30), 11337–11344. https://doi.org/10.1021/jacs.1c06287.

(20) Moss, R. A.; Fu, X.; Ma, Y.; Sauers, R. R. 2-Thienylchlorocarbene: Reactivity toward Alkenes. *Tetrahedron Lett.* **2003**, *44* (4), 773–776. https://doi.org/10.1016/S0040-4039(02)02698-9.

(21) Goikhman, R.; Jacques, T. L.; Sames, D. C−H Bonds as Ubiquitous Functionality: A General Approach to Complex Arylated Pyrazoles via Sequential Regioselective C-Arylation and N-Alkylation Enabled by SEM-Group Transposition. *J. Am. Chem. Soc.* **2009**, *131* (8), 3042–3048. https://doi.org/10.1021/ja8096114.

(22) Jeong, S.; Kim, E.; Kim, M.; Hwang, Y. J.; Padhi, B.; Choi, J.; Lee, Y.; Joo, J. M. Divergent Strategies for the π-Extension of Heteroaryl Halides Using Norbornadiene as an Acetylene Synthon. *Org. Lett.* **2020**, *22* (24), 9670–9676. https://doi.org/10.1021/acs.orglett.0c03732.

(23) Ballesteros, P.; Elguero, J.; Claramunt, R. M. Reactivity of Azoles towards Benzaldehyde and Its Dimethylacetal. Synthesis of N,N'-Diazolylphenylmethanes. *Tetrahedron* **1985**, *41* (24), 5955–5963. https://doi.org/10.1016/S0040-4020(01)91435-8.

(24) M. J. Frisch, G. W. Trucks, H. B. Schlegel, G. E. Scuseria,; M. A. Robb, J. R. Cheeseman, G. Scalmani, V. Barone,; G. A. Petersson, H. Nakatsuji, X. Li, M. Caricato, A. V. Marenich,; J. Bloino, B. G. Janesko, R. Gomperts, B. Mennucci, H. P. Hratchian,; J. V. Ortiz, A. F. Izmaylov, J. L. Sonnenberg, D. Williams-Young,; F. Ding, F. Lipparini, F. Egidi, J. Goings, B. Peng, A. Petrone,; T. Henderson, D. Ranasinghe, V. G. Zakrzewski, J. Gao, N. Rega,; G. Zheng, W. Liang, M. Hada, M. Ehara, K. Toyota, R. Fukuda,; J. Hasegawa, M. Ishida, T. Nakajima, Y. Honda, O. Kitao, H. Nakai,; T. Vreven, K. Throssell, J. A. Montgomery, Jr., J. E. Peralta,; F. Ogliaro, M. J. Bearpark, J. J. Heyd, E. N. Brothers, K. N. Kudin,; V. N. Staroverov, T. A. Keith, R. Kobayashi, J. Normand,; K. Raghavachari, A. P. Rendell, J. C. Burant, S. S. Iyengar,; J. Tomasi, M. Cossi, J. M. Millam, M. Klene, C. Adamo, R. Cammi,; J. W. Ochterski, R. L. Martin, K. Morokuma, O. Farkas,; J. B. Foresman, and D. J. Fox. Gaussian 16, Revision A.03, 2016.

(25) Grimme, S. Supramolecular Binding Thermodynamics by Dispersion-Corrected Density Functional Theory. *Chem. – Eur. J.* **2012**, *18* (32), 9955–9964. https://doi.org/10.1002/chem.201200497.

(26) Li, Y.-P.; Gomes, J.; Mallikarjun Sharada, S.; Bell, A. T.; Head-Gordon, M. Improved Force-Field Parameters for QM/MM Simulations of the Energies of Adsorption for Molecules in Zeolites and a Free Rotor

Correction to the Rigid Rotor Harmonic Oscillator Model for Adsorption Enthalpies. *J. Phys. Chem. C* **2015**, *119* (4), 1840–1850. https://doi.org/10.1021/jp509921r.

(27) Luchini, G.; Alegre-Requena, J. V.; Funes-Ardoiz, I.; Paton, R. S. GoodVibes: Automated Thermochemistry for Heterogeneous Computational Chemistry Data. *F1000Research* **2020**, *9*, 291. https://doi.org/10.12688/f1000research.22758.1.

(28) Community, B. O. *Blender - a 3D Modelling and Rendering Package*; Blender Foundation: Stichting Blender Foundation, Amsterdam, 2018.

(29) Pracht, P.; Bohle, F.; Grimme, S. Automated Exploration of the Low-Energy Chemical Space with Fast Quantum Chemical Methods. *Phys Chem Chem Phys* **2020**, *22* (14), 7169–7192. https://doi.org/10.1039/C9CP06869D.

IX: Spectra

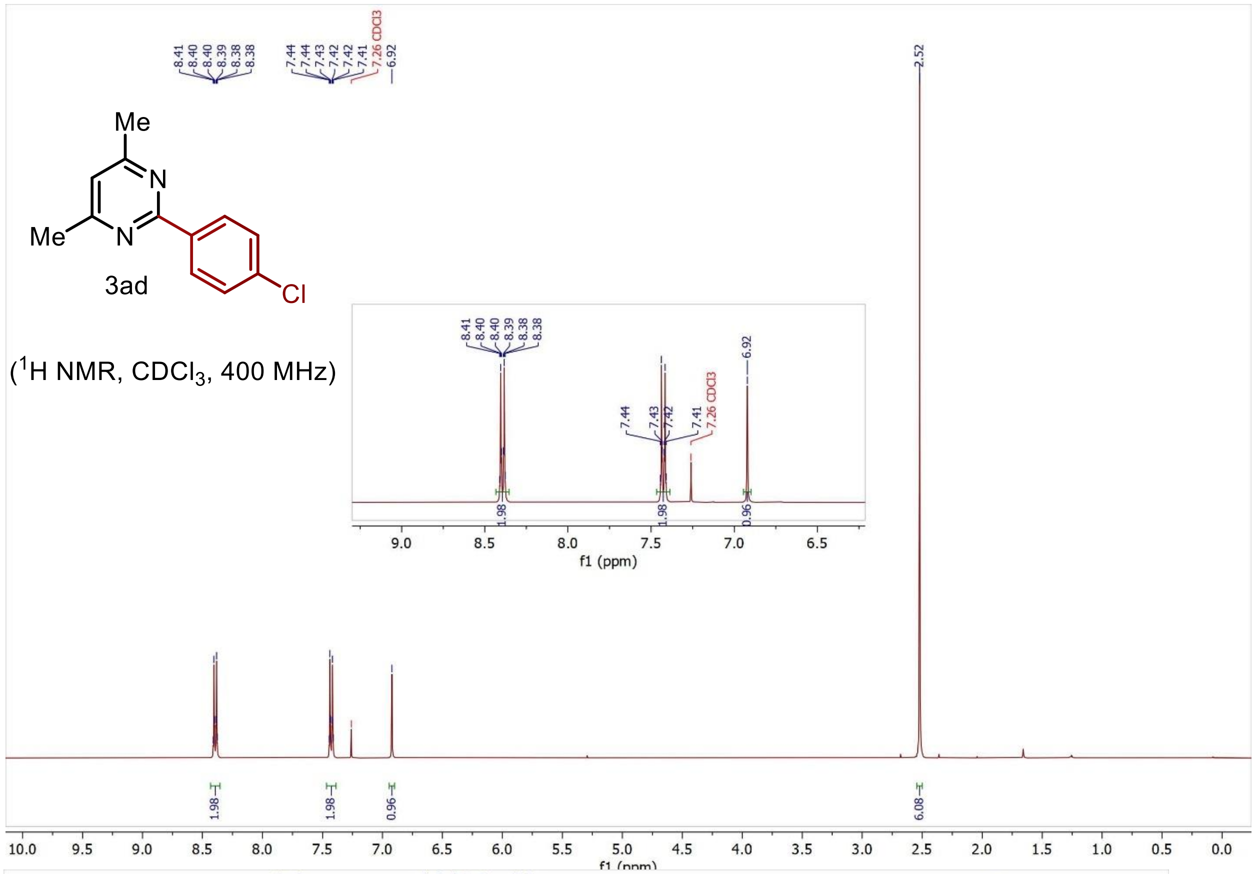

8.41
8.40
8.40
8.39
8.38
8.38
7.44
7.44
7.43
7.42
7.42
7.41
7.26 CDCl3
6.92
2.52
Me
N
Me
N
3ad
Cl
(1H NMR, CDCl3, 400 MHz)
1.98
1.98
0.96
6.08
f1 (ppm)

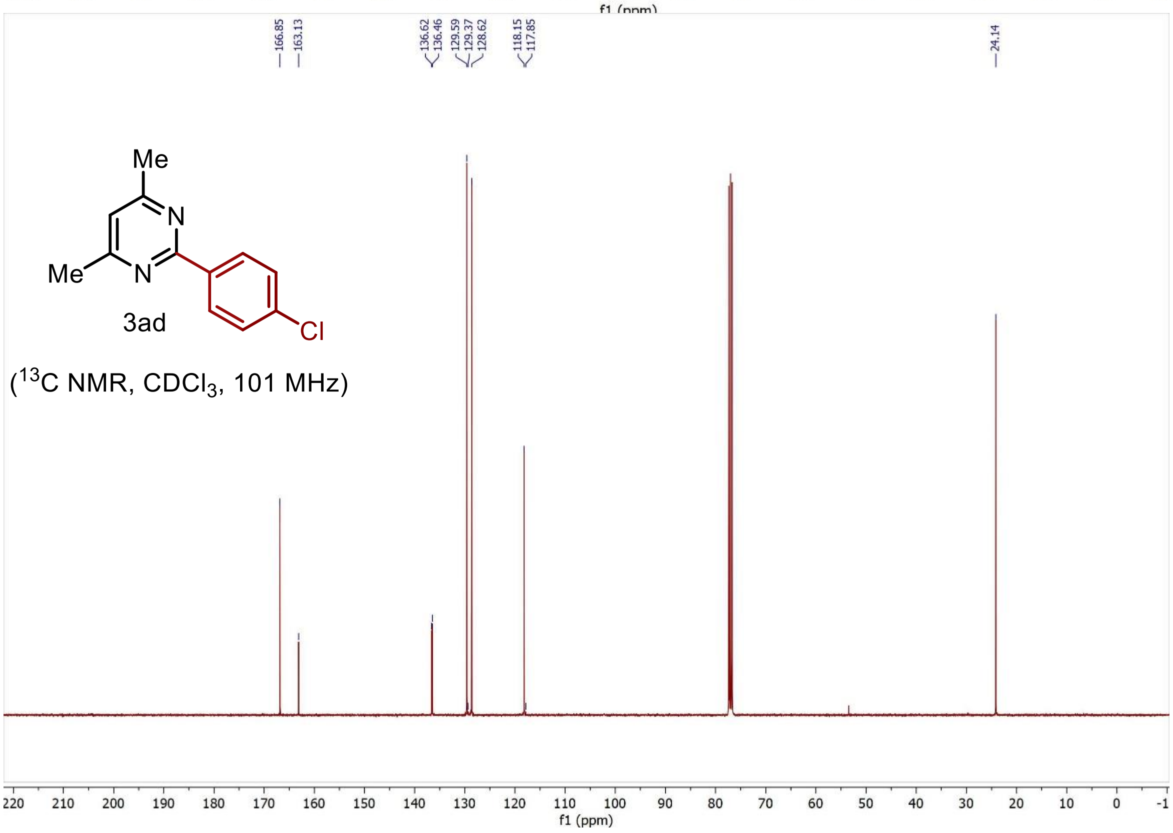

166.85
163.13
136.62
136.46
129.59
129.37
128.62
118.15
117.85
24.14
Me
N
Me
N
3ad
Cl
(13C NMR, CDCl3, 101 MHz)
f1 (ppm)

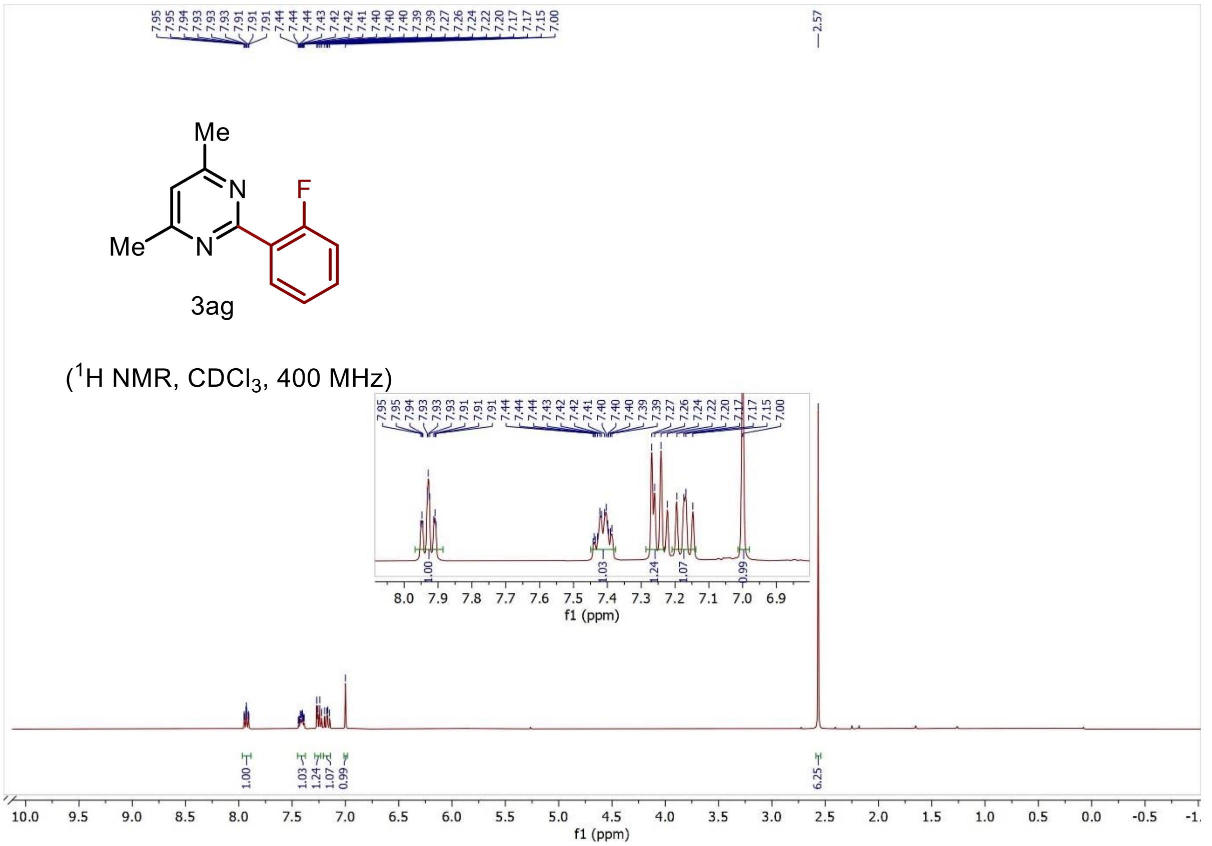
Me
N
F
Me
N
3ag
(1H NMR, CDCl3, 400 MHz)
f1 (ppm)

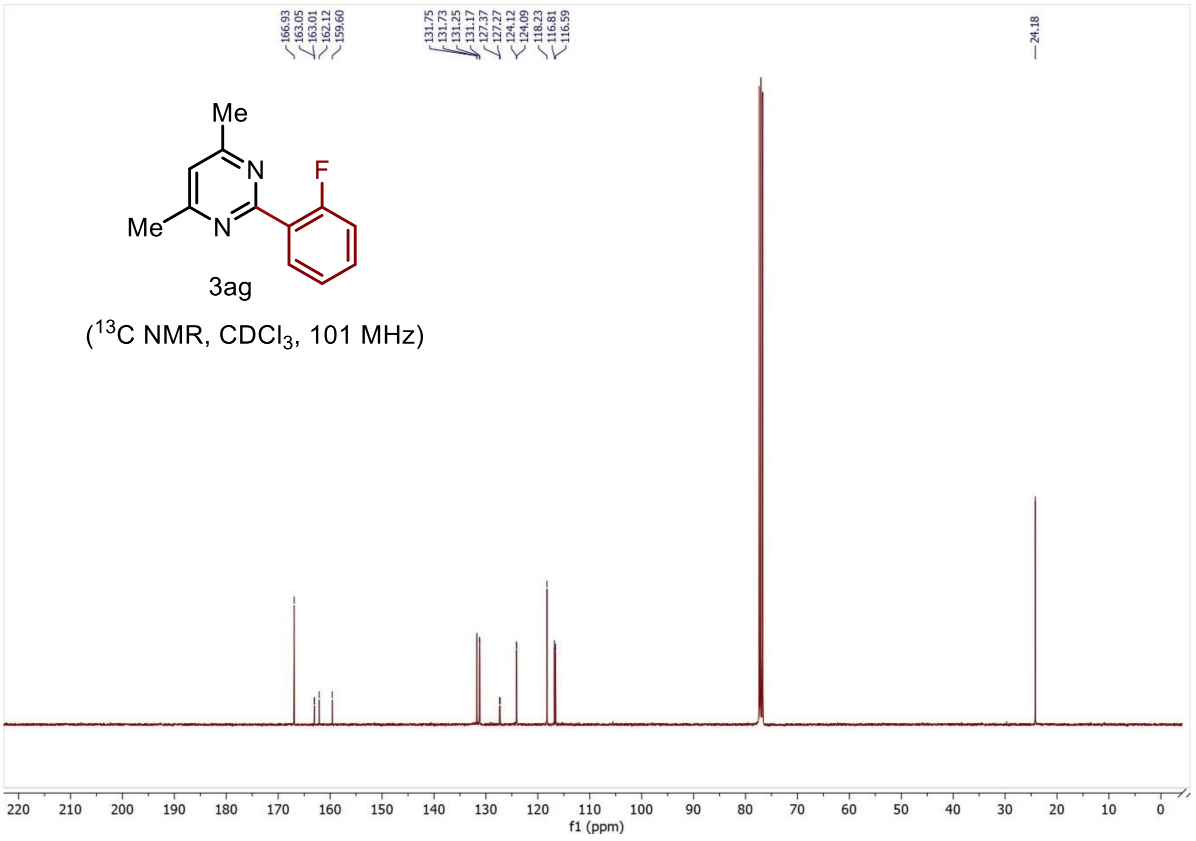
Me
N
F
Me
N
3ag
(13C NMR, CDCl3, 101 MHz)
f1 (ppm)

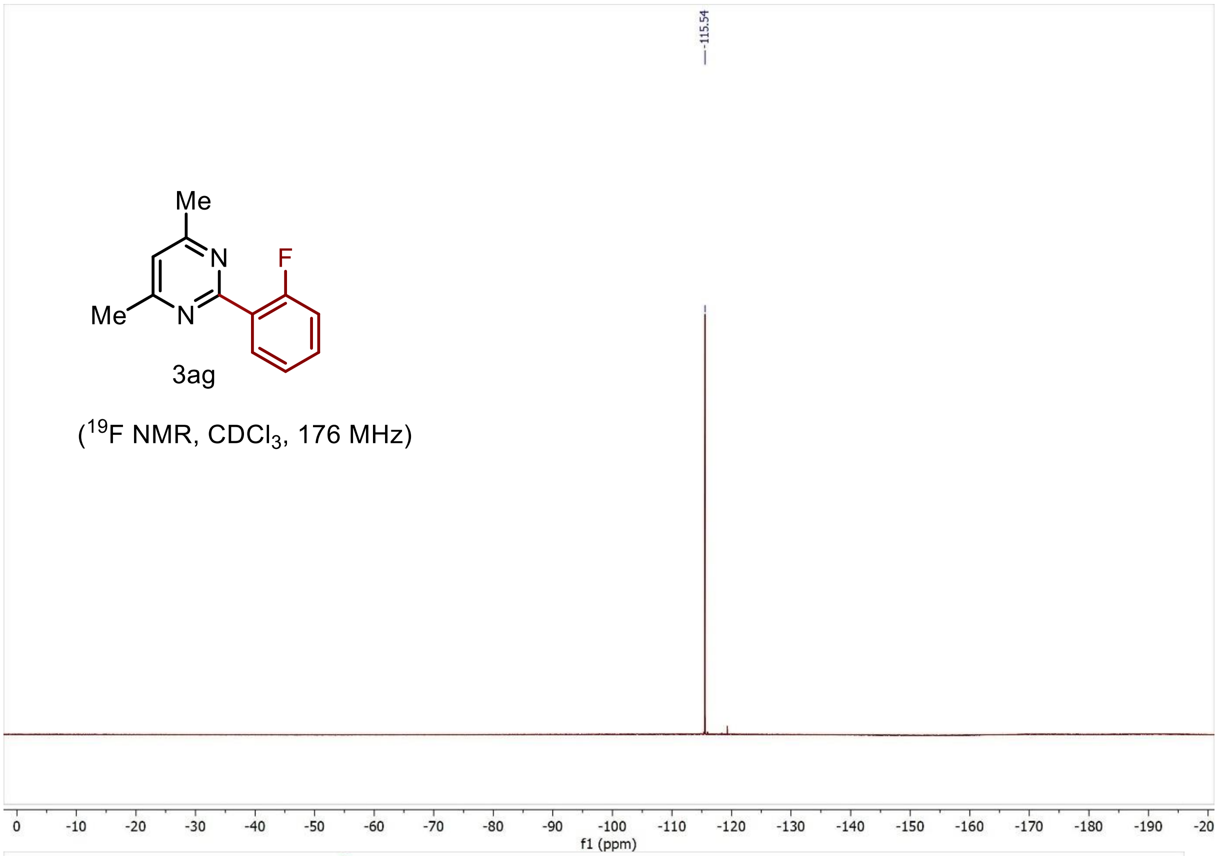

-115.54
Me
N
F
Me
N
3ag
(19F NMR, CDCl3, 176 MHz)
f1 (ppm)

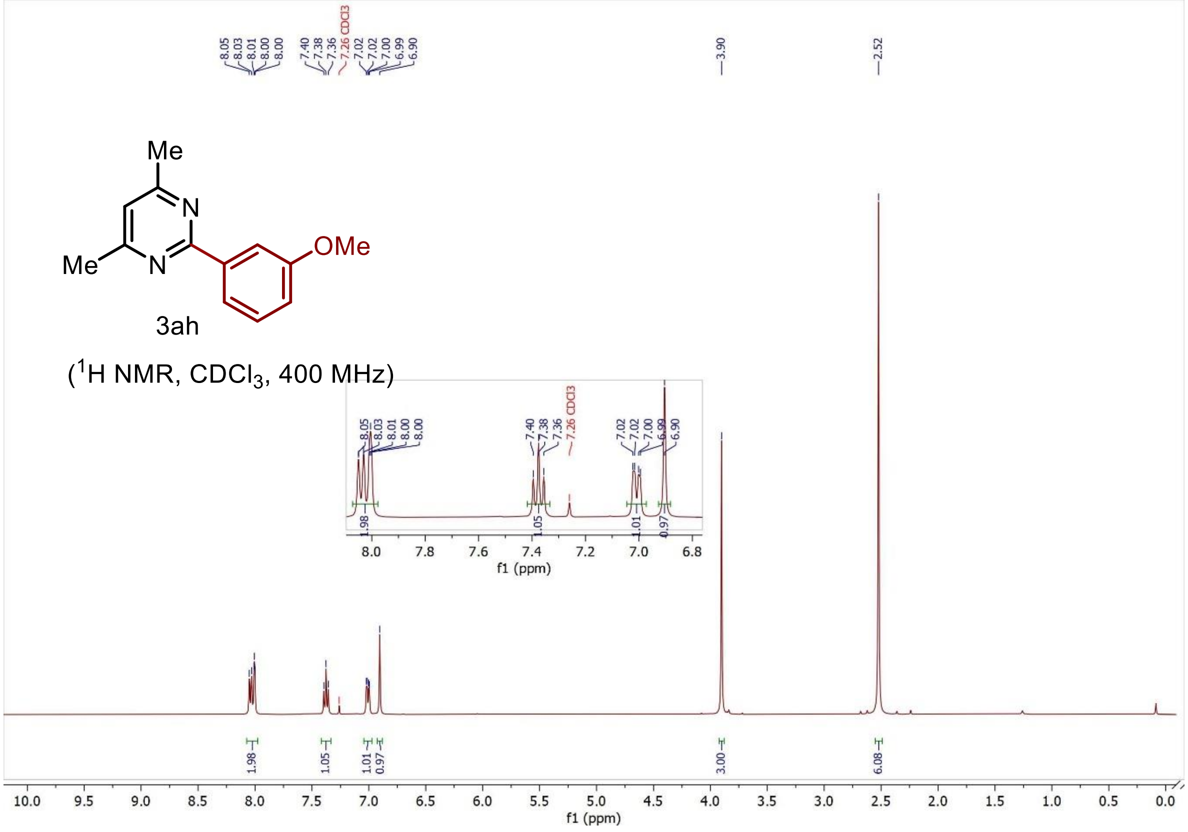

8.05
8.03
8.01
8.00
8.00
7.40
7.38
7.36
7.26 CDCl3
7.02
7.02
7.00
6.99
6.90
3.90
2.52
Me
N
OMe
Me
N
3ah
(1H NMR, CDCl3, 400 MHz)
1.98
1.05
1.01
0.97
3.00
6.08
f1 (ppm)

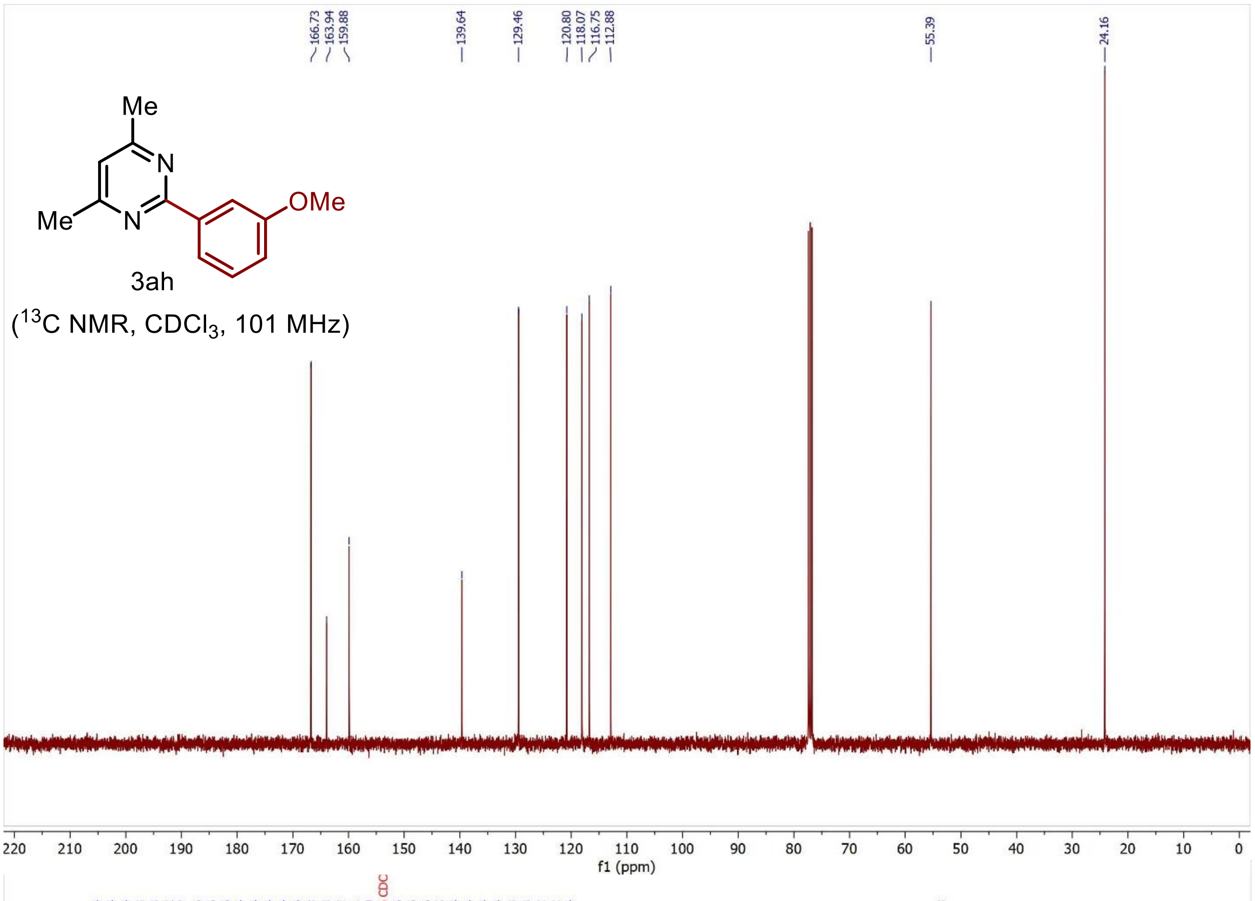
166.73
163.94
159.88
139.64
129.46
120.80
118.07
116.75
112.88
55.39
24.16
Me
N
OMe
Me
N
3ah
(13C NMR, CDCl3, 101 MHz)
220 210 200 190 180 170 160 150 140 130 120 110 100 90 80 70 60 50 40 30 20 10 0
f1 (ppm)

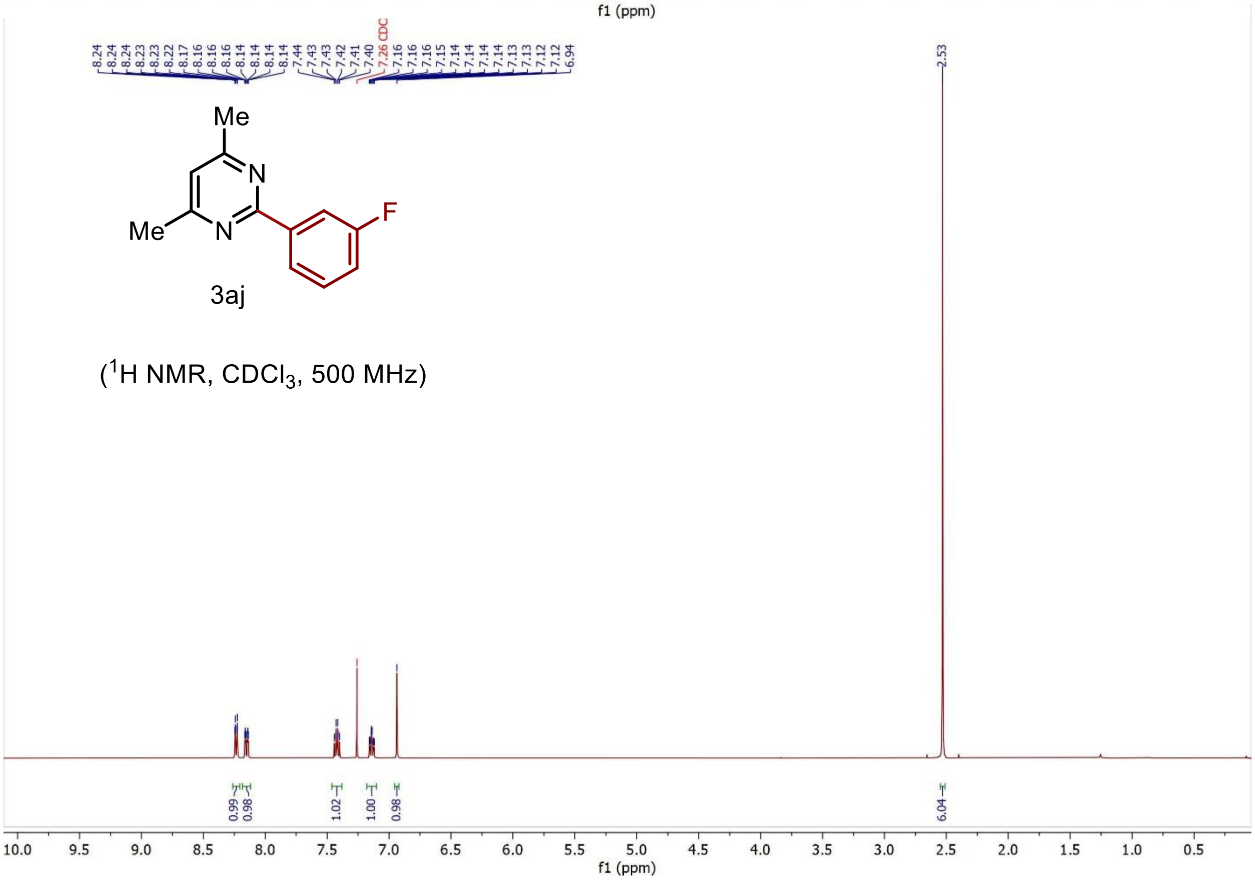
8.24
8.24
8.24
8.23
8.22
8.17
8.16
8.16
8.16
8.14
8.14
8.14
8.14
7.44
7.43
7.43
7.42
7.41
7.40
7.26 CDC
7.16
7.16
7.15
7.14
7.14
7.14
7.14
7.13
7.13
7.12
7.12
6.94
2.53
Me
N
F
Me
N
3aj
(1H NMR, CDCl3, 500 MHz)
0.99
0.98
1.02
1.00
0.98
6.04
10.0 9.5 9.0 8.5 8.0 7.5 7.0 6.5 6.0 5.5 5.0 4.5 4.0 3.5 3.0 2.5 2.0 1.5 1.0 0.5
f1 (ppm)

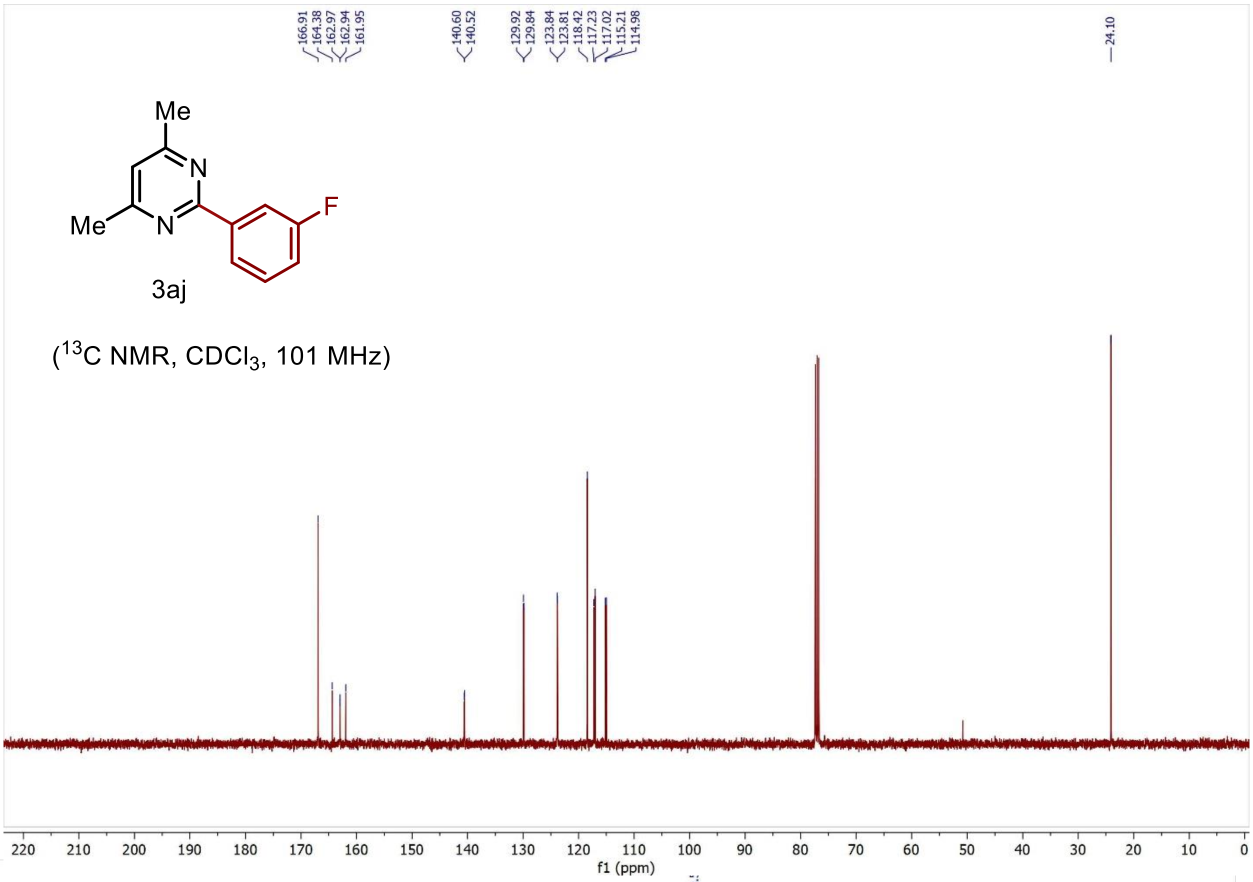
166.91
164.38
162.97
162.94
161.95
140.60
140.52
129.92
129.84
123.84
123.81
118.42
117.23
117.02
115.21
114.98
24.10
Me
N
F
Me
N
3aj
(^{13}C NMR, $CDCl_3$, 101 MHz)
f1 (ppm)

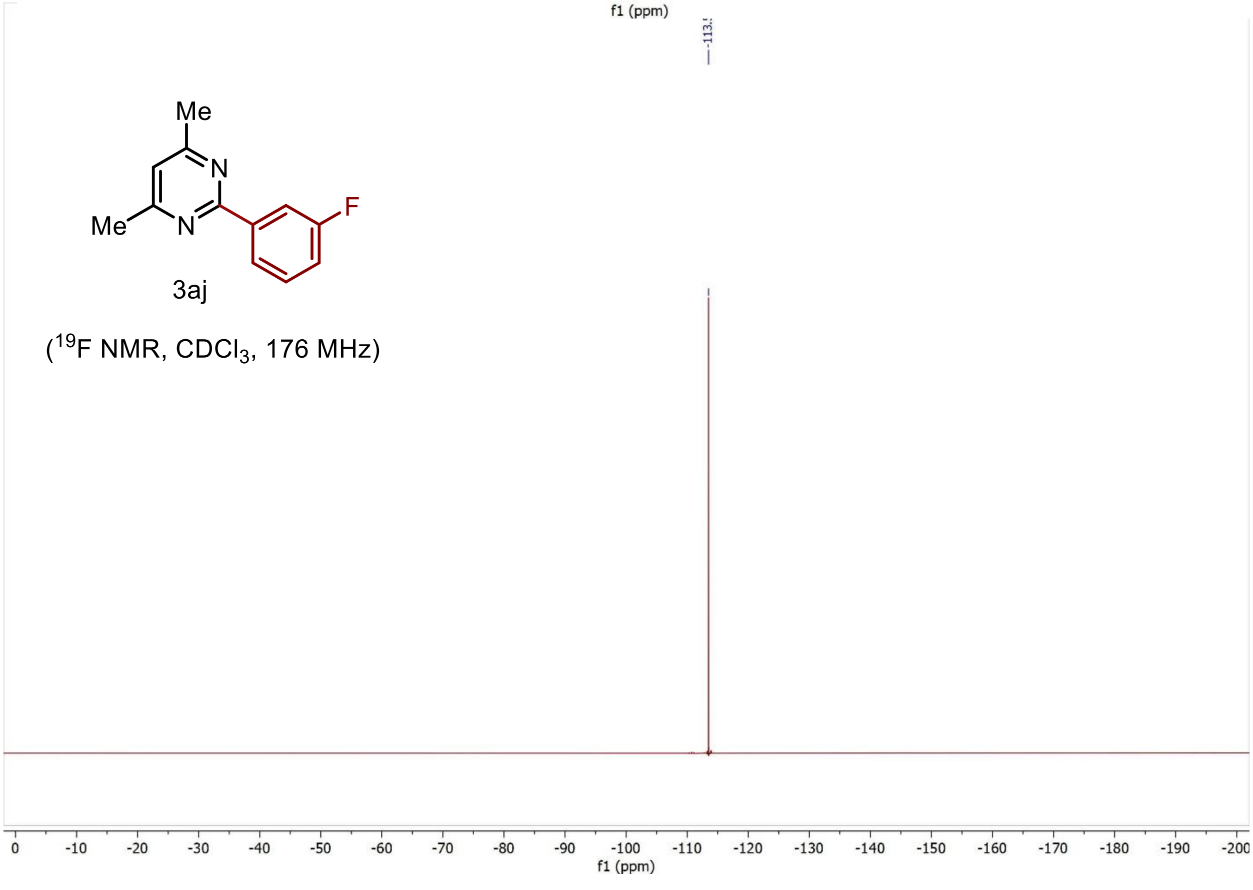
Me
N
F
Me
N
3aj
(^{19}F NMR, $CDCl_3$, 176 MHz)
f1 (ppm)

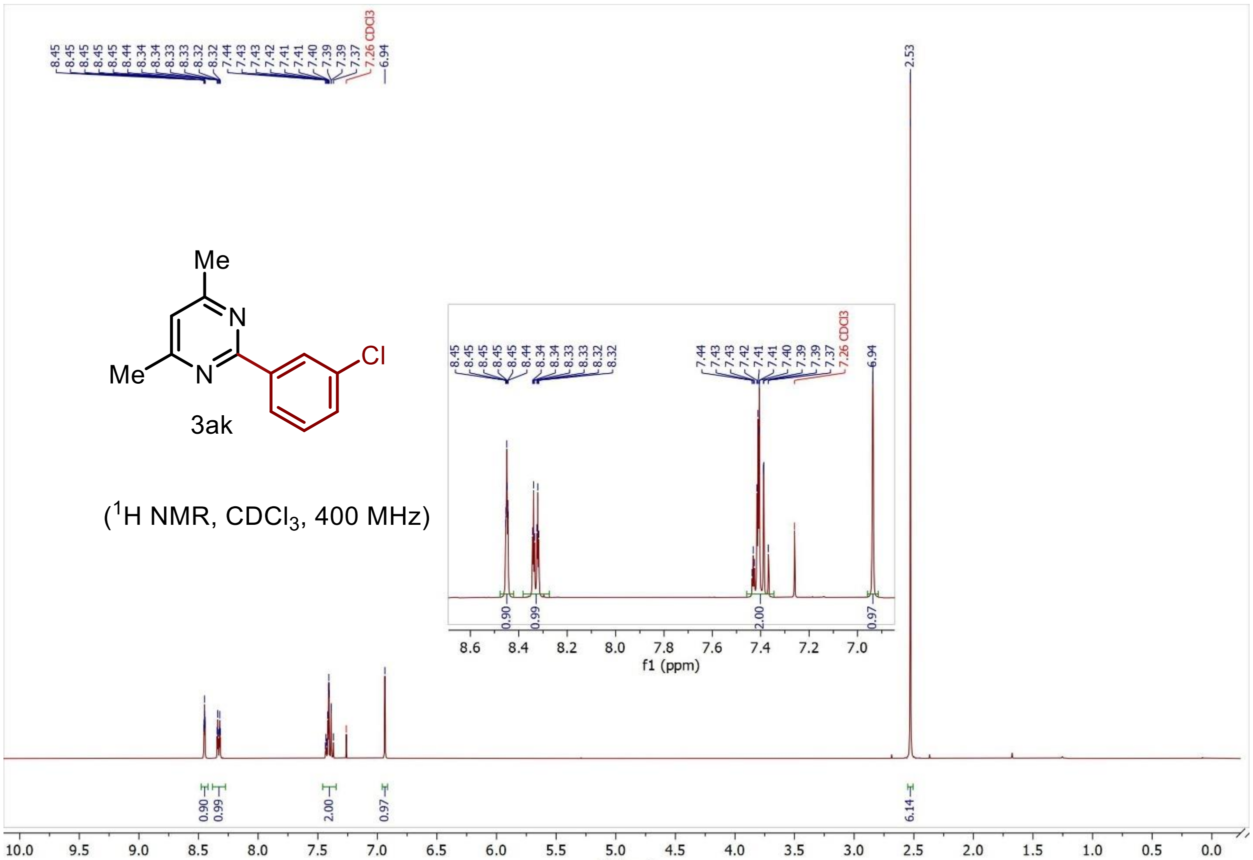
8.45 8.45 8.45 8.45 8.44 8.34 8.34 8.33 8.33 8.32 8.32 7.44 7.43 7.43 7.42 7.41 7.41 7.40 7.39 7.39 7.37 7.26 CDCl3 6.94
2.53
Me
N
N
Me
Cl
3ak
(^{1}H NMR, $CDCl_3$, 400 MHz)
0.90 0.99 2.00 0.97 6.14
f1 (ppm)

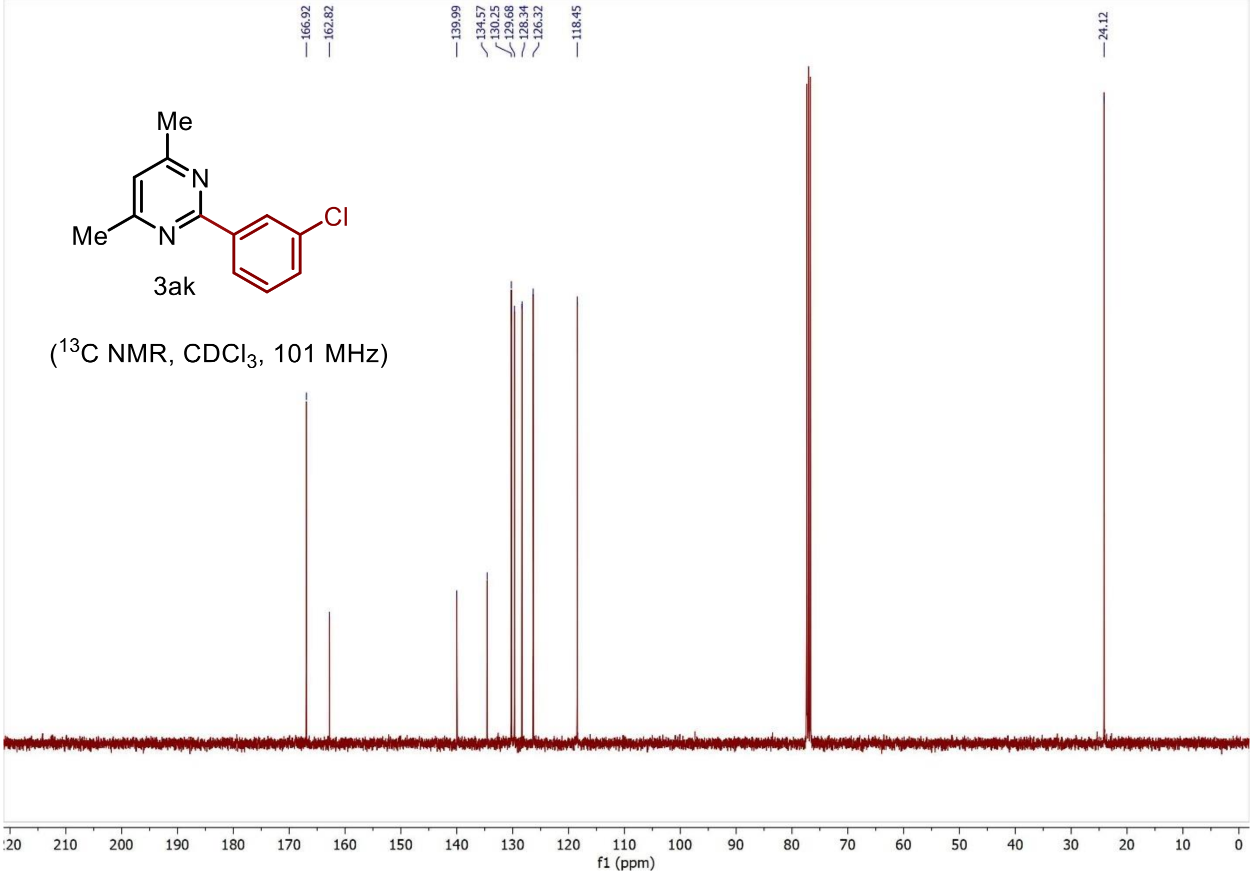
166.92 162.82 139.99 134.57 130.25 129.68 128.34 126.32 118.45 24.12
Me
N
N
Me
Cl
3ak
(^{13}C NMR, $CDCl_3$, 101 MHz)
f1 (ppm)

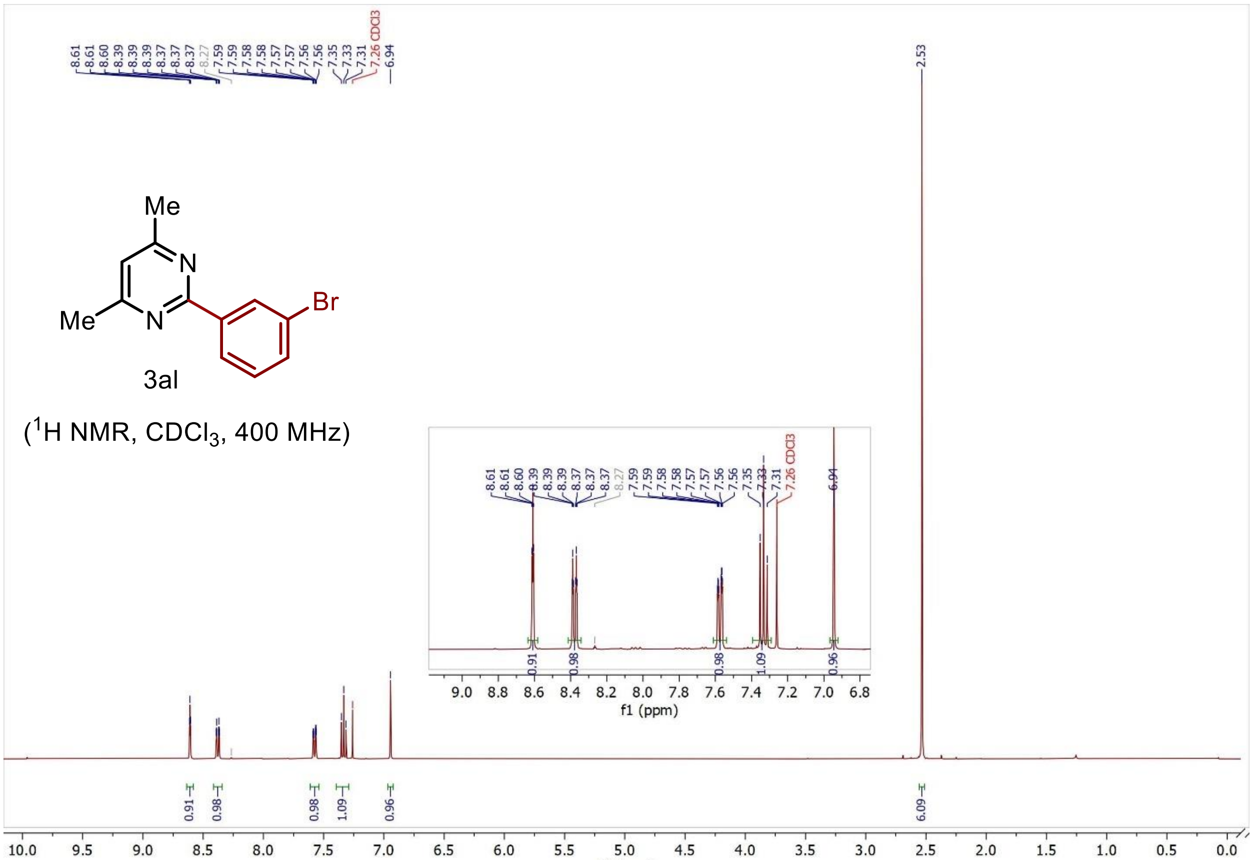

Me
N
Me
N
Br
3al
(¹H NMR, CDCl₃, 400 MHz)
8.61 8.61 8.60 8.39 8.39 8.39 8.37 8.37 8.37 8.27 7.59 7.59 7.58 7.58 7.57 7.57 7.56 7.56 7.35 7.33 7.31 7.26 CDCl3 6.94
2.53
0.91 0.98 0.98 1.09 0.96 6.09
f1 (ppm)

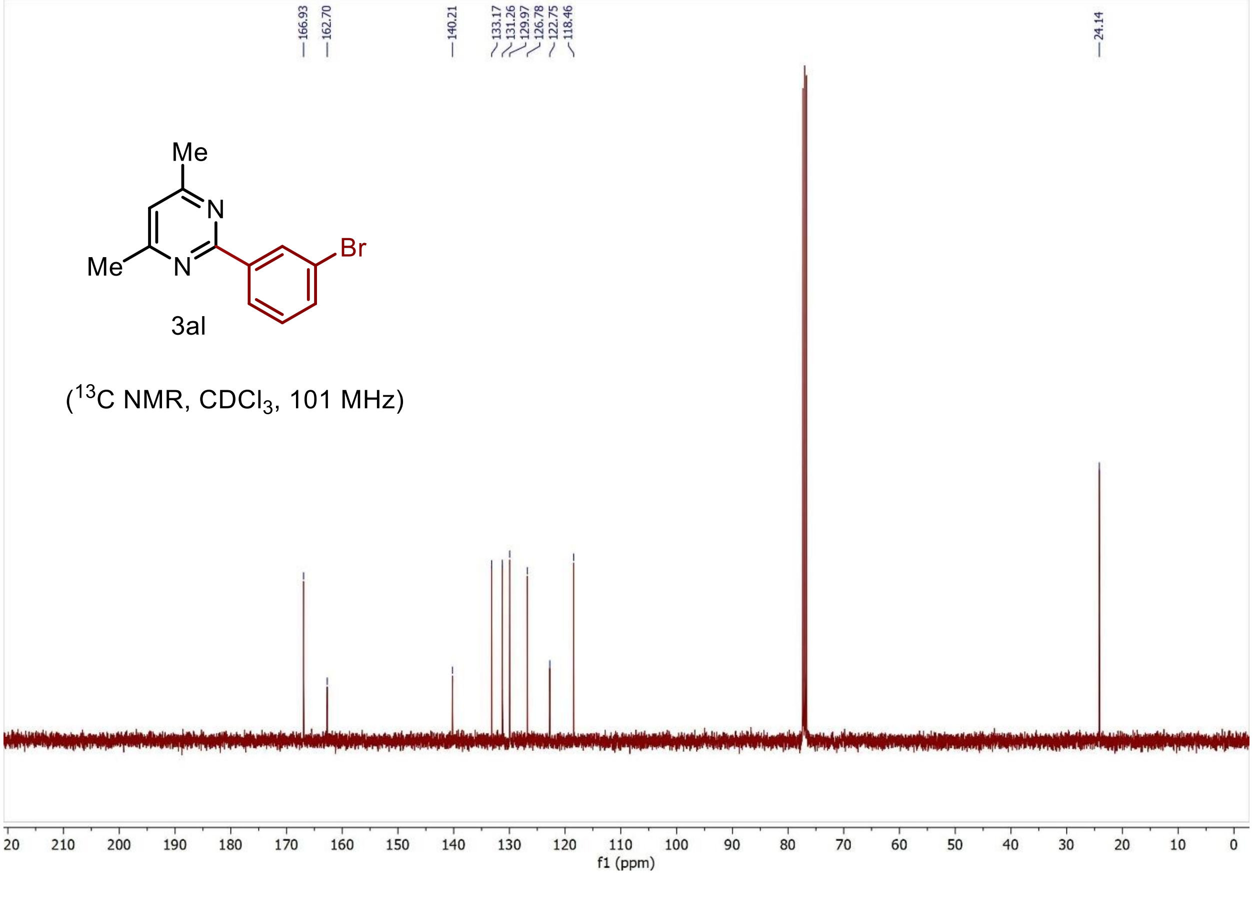

166.93 162.70 140.21 133.17 131.26 129.97 126.78 122.75 118.46 24.14
Me
N
Me
N
Br
3al
(¹³C NMR, CDCl₃, 101 MHz)
f1 (ppm)

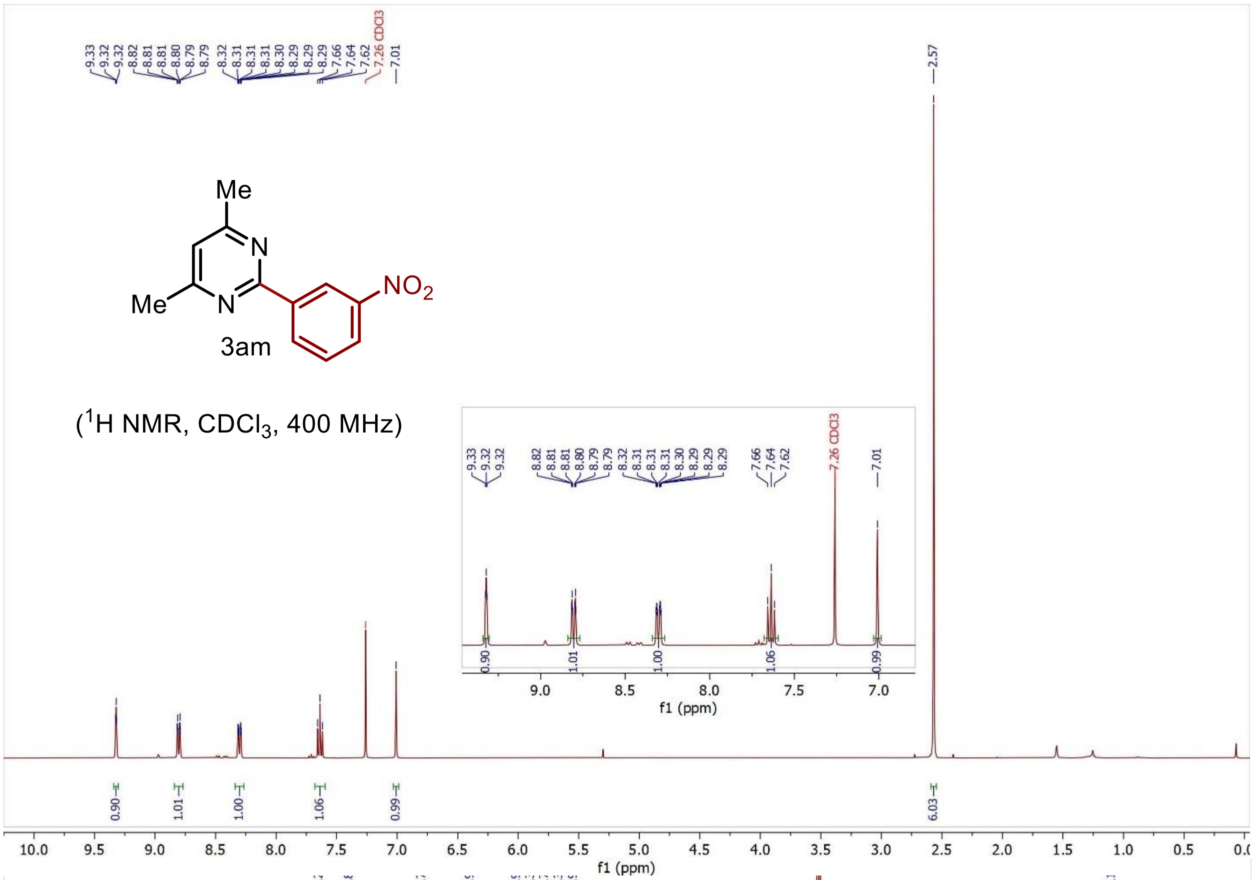
9.33
9.32
9.32
8.82
8.81
8.81
8.80
8.79
8.79
8.32
8.31
8.31
8.31
8.30
8.29
8.29
8.29
7.66
7.64
7.62
7.26 CDCl3
7.01
2.57
Me
N
Me
N
NO2
3am
(1H NMR, CDCl3, 400 MHz)
0.90
1.01
1.00
1.06
0.99
6.03
f1 (ppm)

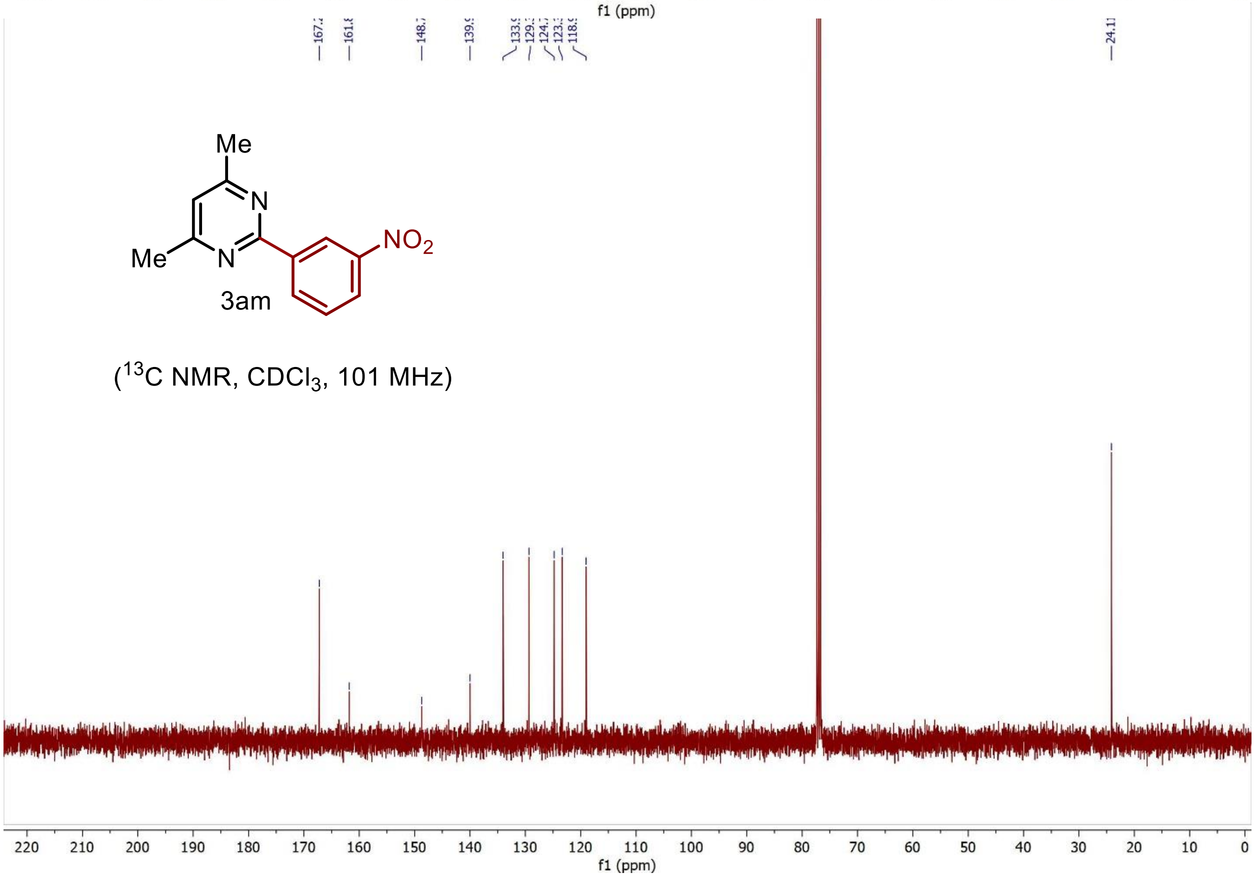
167.2
161.8
148.7
139.9
133.9
129.5
124.7
123.3
118.9
24.11
Me
N
Me
N
NO2
3am
(13C NMR, CDCl3, 101 MHz)
f1 (ppm)

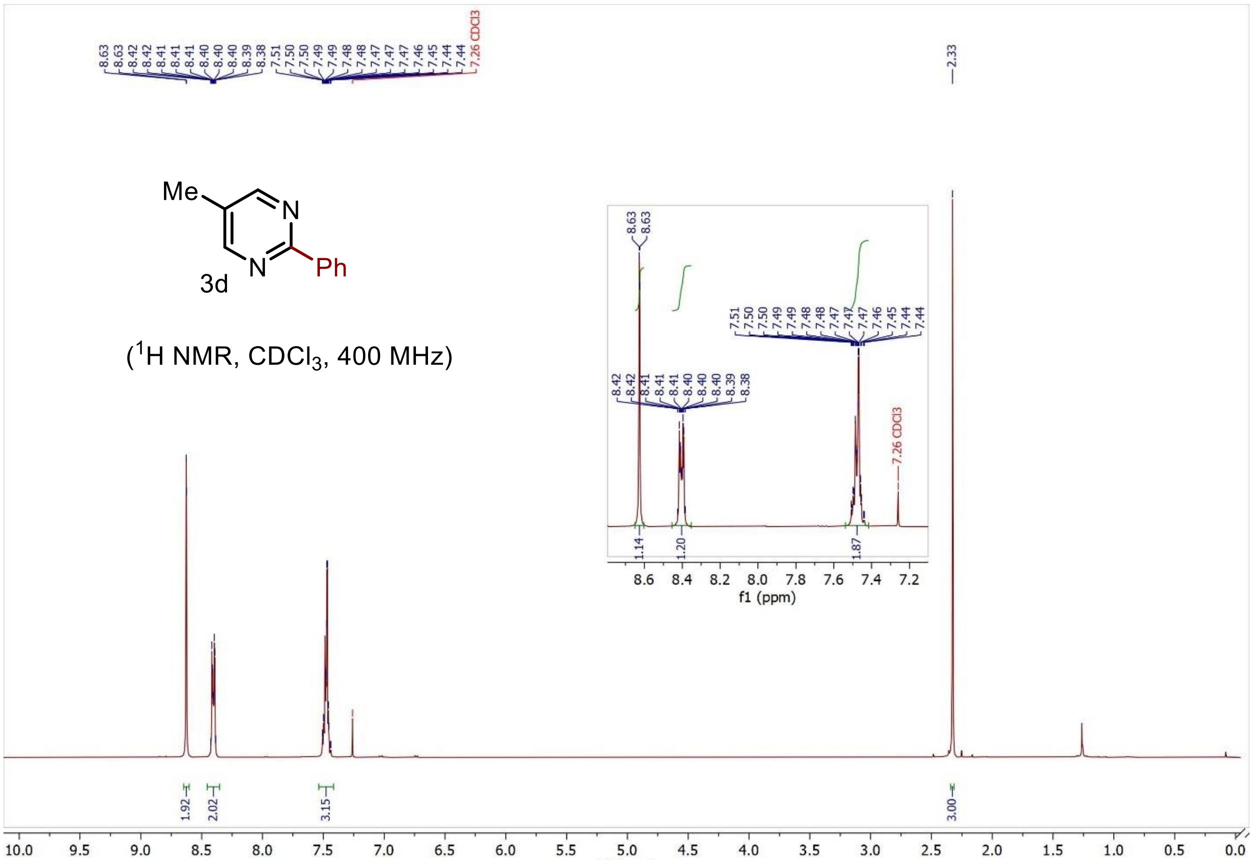
8.63
8.63
8.42
8.42
8.41
8.41
8.40
8.40
8.40
8.39
8.38
7.51
7.50
7.50
7.49
7.49
7.48
7.48
7.47
7.47
7.47
7.46
7.45
7.44
7.44
7.26 CDCl3
2.33
Me
N
N
Ph
3d
(1H NMR, CDCl3, 400 MHz)
1.14
1.20
1.87
f1 (ppm)
1.92
2.02
3.15
3.00

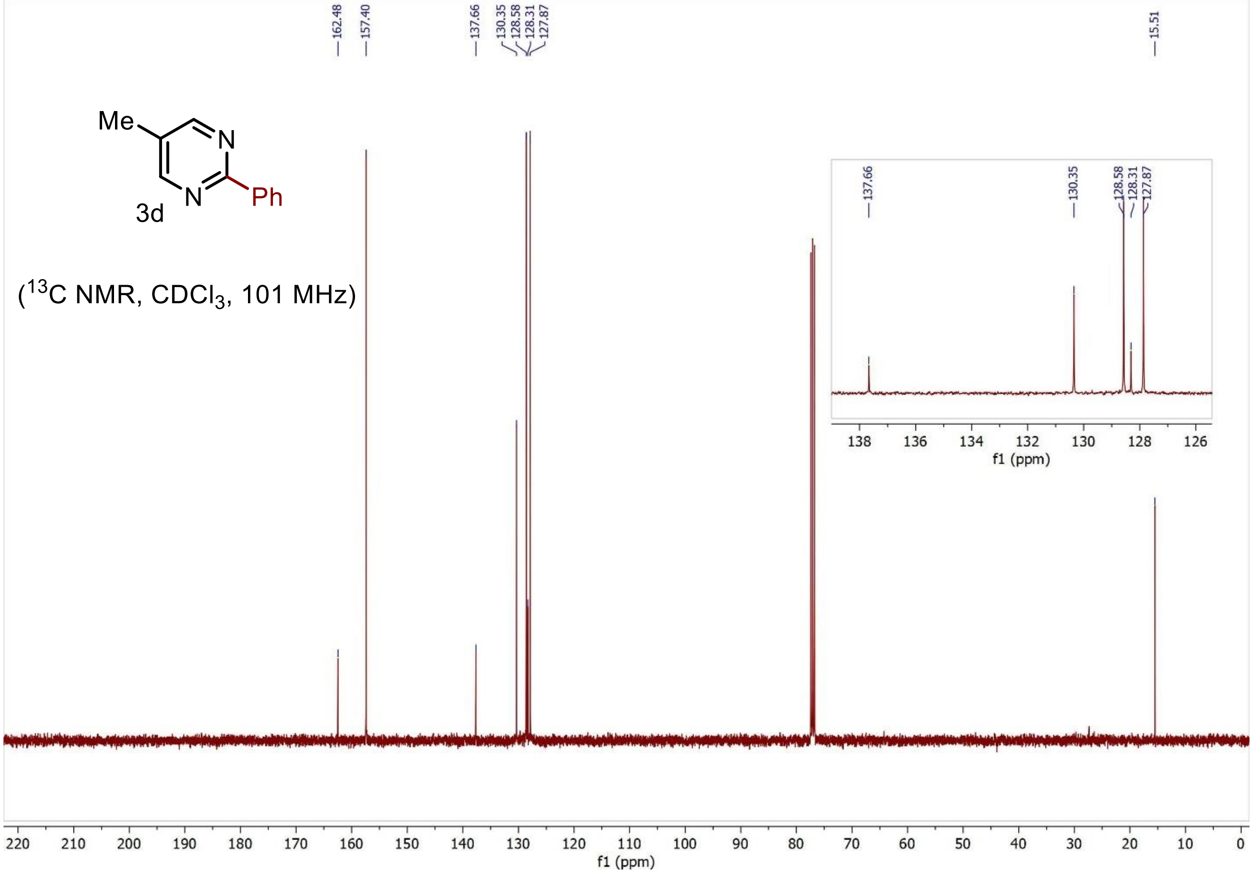
162.48
157.40
137.66
130.35
128.58
128.31
127.87
15.51
Me
N
N
Ph
3d
(13C NMR, CDCl3, 101 MHz)
f1 (ppm)
f1 (ppm)

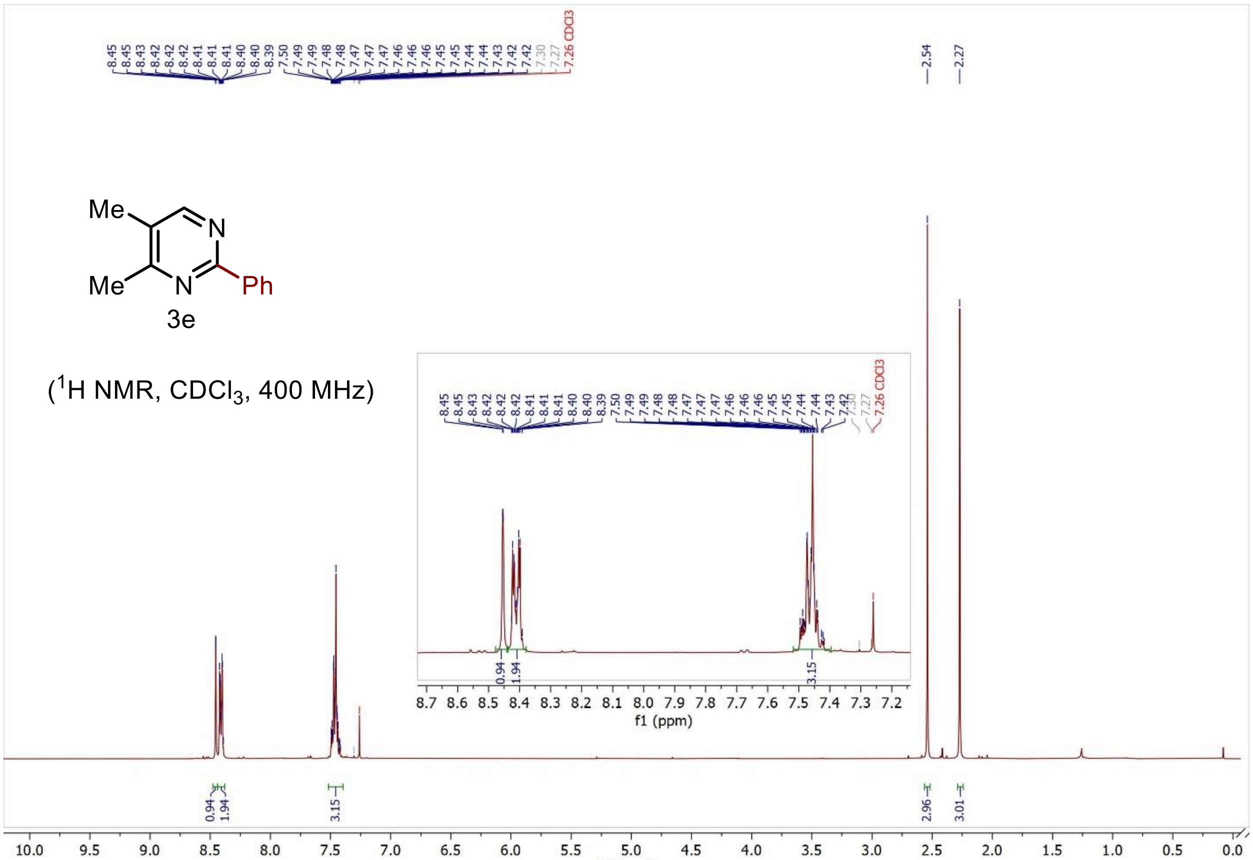
8.45 8.45 8.43 8.42 8.42 8.41 8.41 8.41 8.40 8.40 8.39 7.50 7.49 7.49 7.48 7.48 7.47 7.47 7.46 7.46 7.45 7.44 7.44 7.43 7.42 7.42 7.30 7.27 7.26 CDCl3
2.54
2.27
Me
N
Me
N
Ph
3e
(1H NMR, CDCl3, 400 MHz)
0.94
1.94
3.15
2.96
3.01
f1 (ppm)

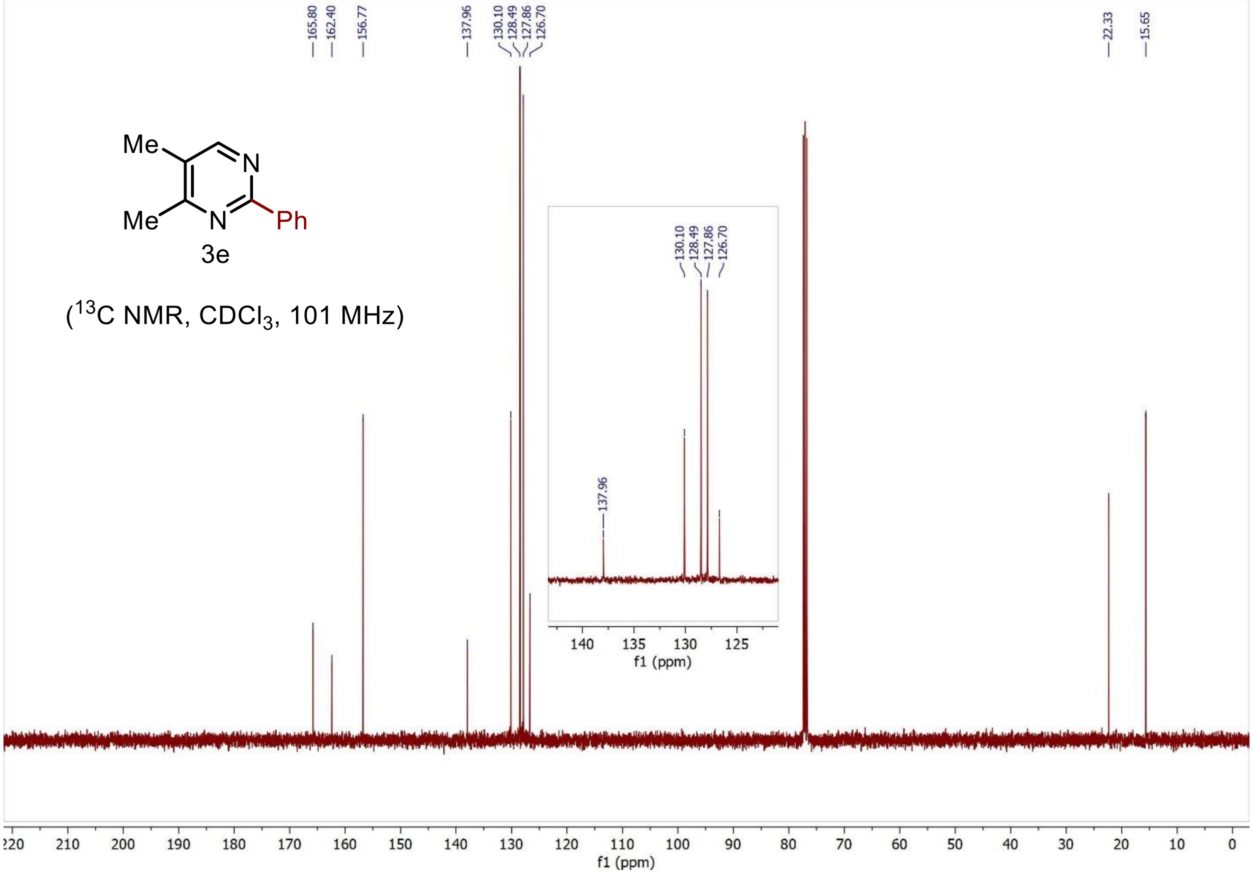
165.80
162.40
156.77
137.96
130.10
128.49
127.86
126.70
22.33
15.65
Me
N
Me
N
Ph
3e
(13C NMR, CDCl3, 101 MHz)
f1 (ppm)

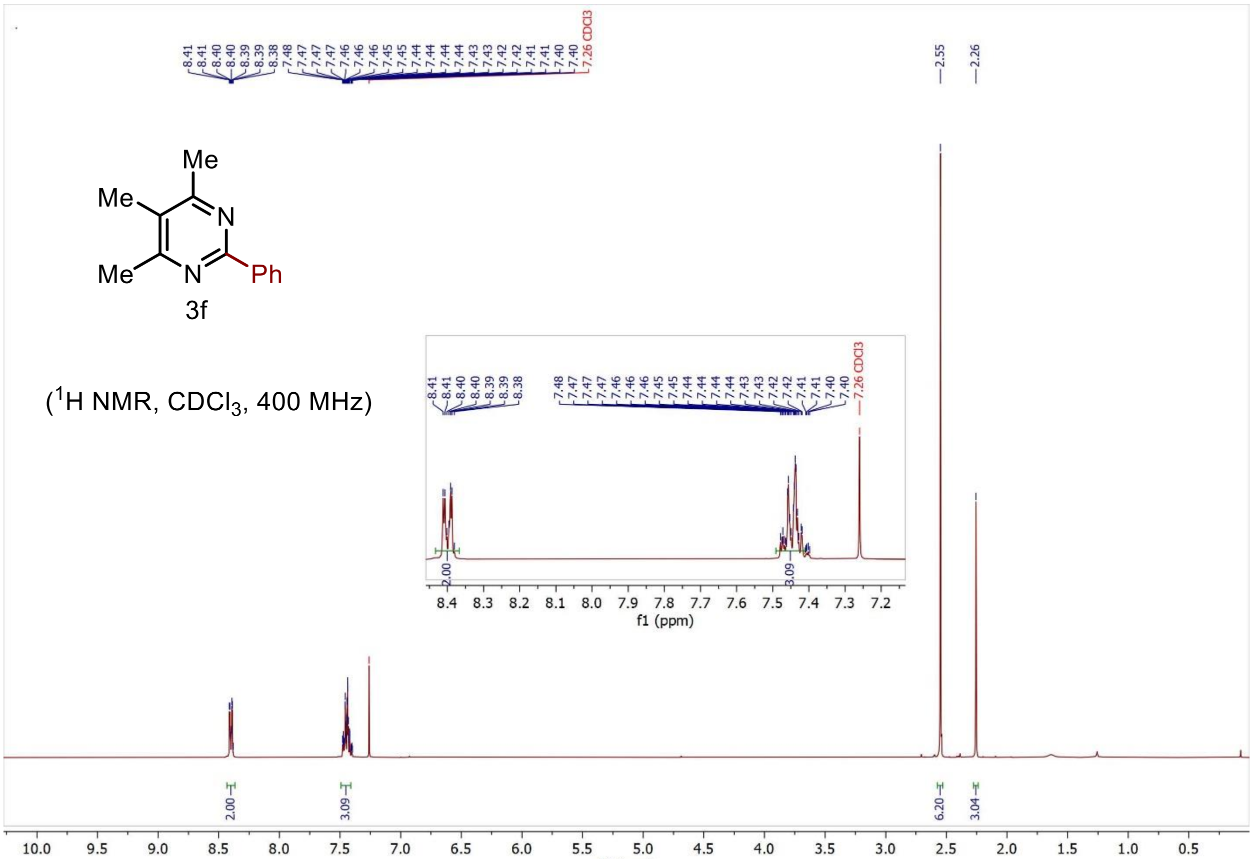
Me
Me
N
Me
N
Ph
3f
(1H NMR, CDCl3, 400 MHz)
8.41 8.41 8.40 8.40 8.39 8.39 8.38 7.48 7.47 7.47 7.46 7.46 7.46 7.45 7.45 7.44 7.44 7.44 7.44 7.43 7.43 7.42 7.42 7.41 7.41 7.40 7.40 7.26 CDCl3
2.55
2.26
2.00
3.09
6.20
3.04
f1 (ppm)

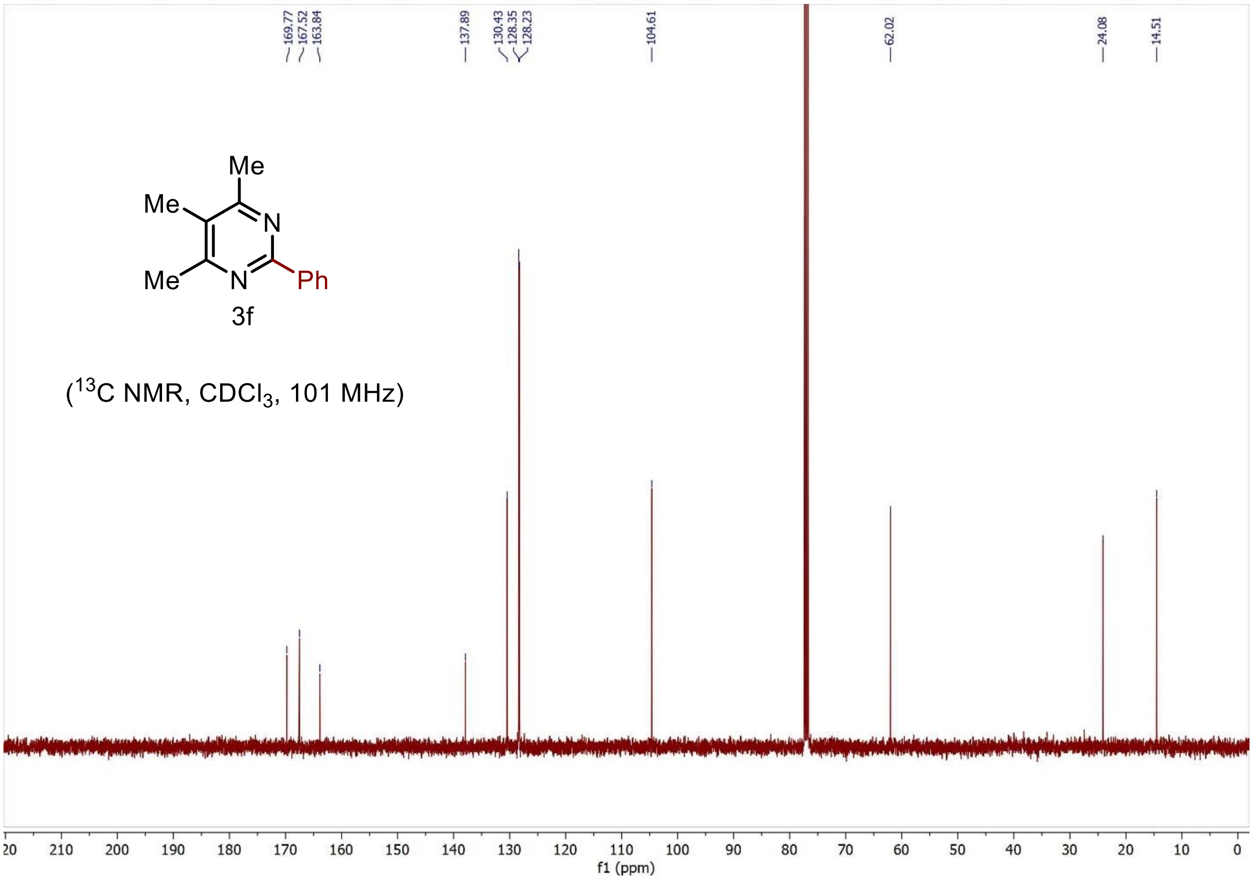
Me
Me
N
Me
N
Ph
3f
(13C NMR, CDCl3, 101 MHz)
169.77 167.52 163.84
137.89
130.43 128.35 128.23
104.61
62.02
24.08
14.51
f1 (ppm)

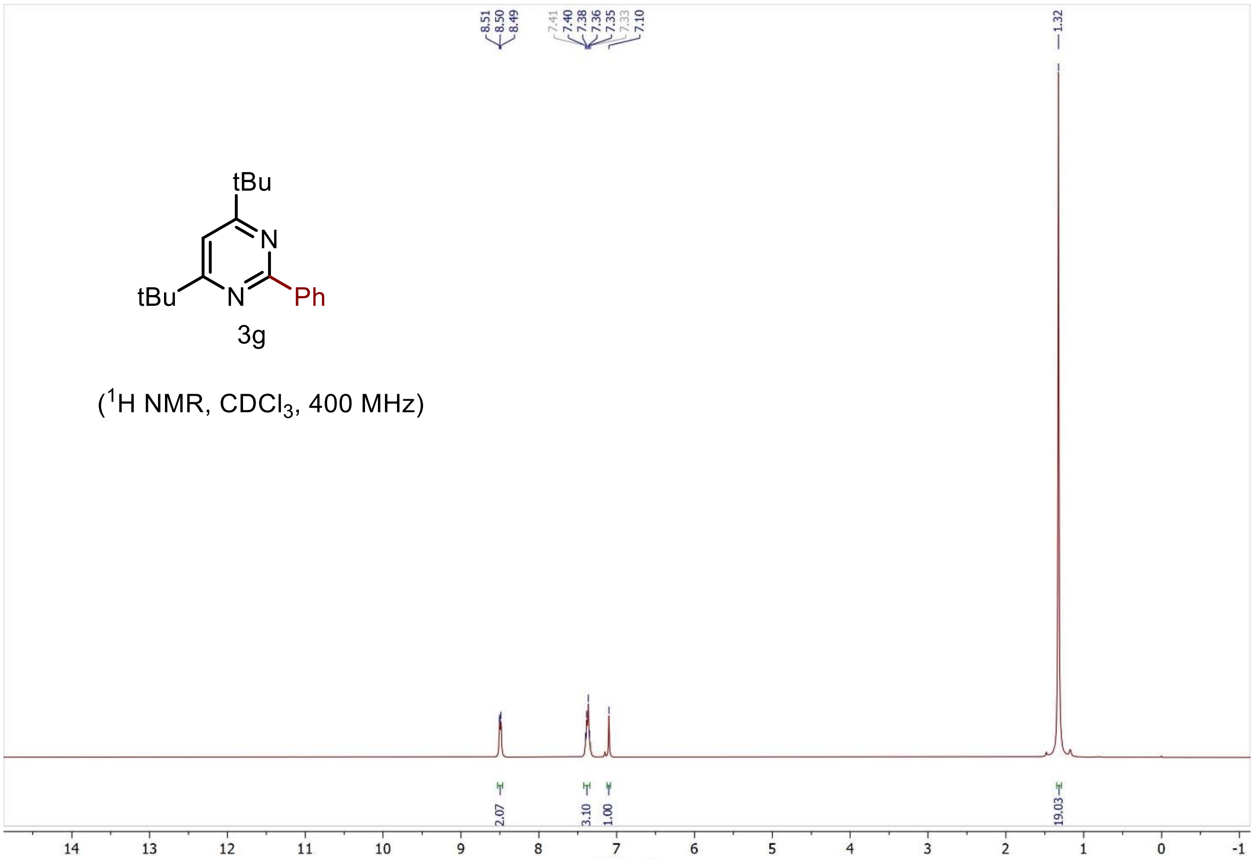

8.51
8.50
8.49
7.41
7.40
7.38
7.36
7.35
7.33
7.10
1.32
tBu
N
tBu
N
Ph
3g
(^{1}H NMR, $CDCl_3$, 400 MHz)
2.07
3.10
1.00
19.03
14 13 12 11 10 9 8 7 6 5 4 3 2 1 0 -1

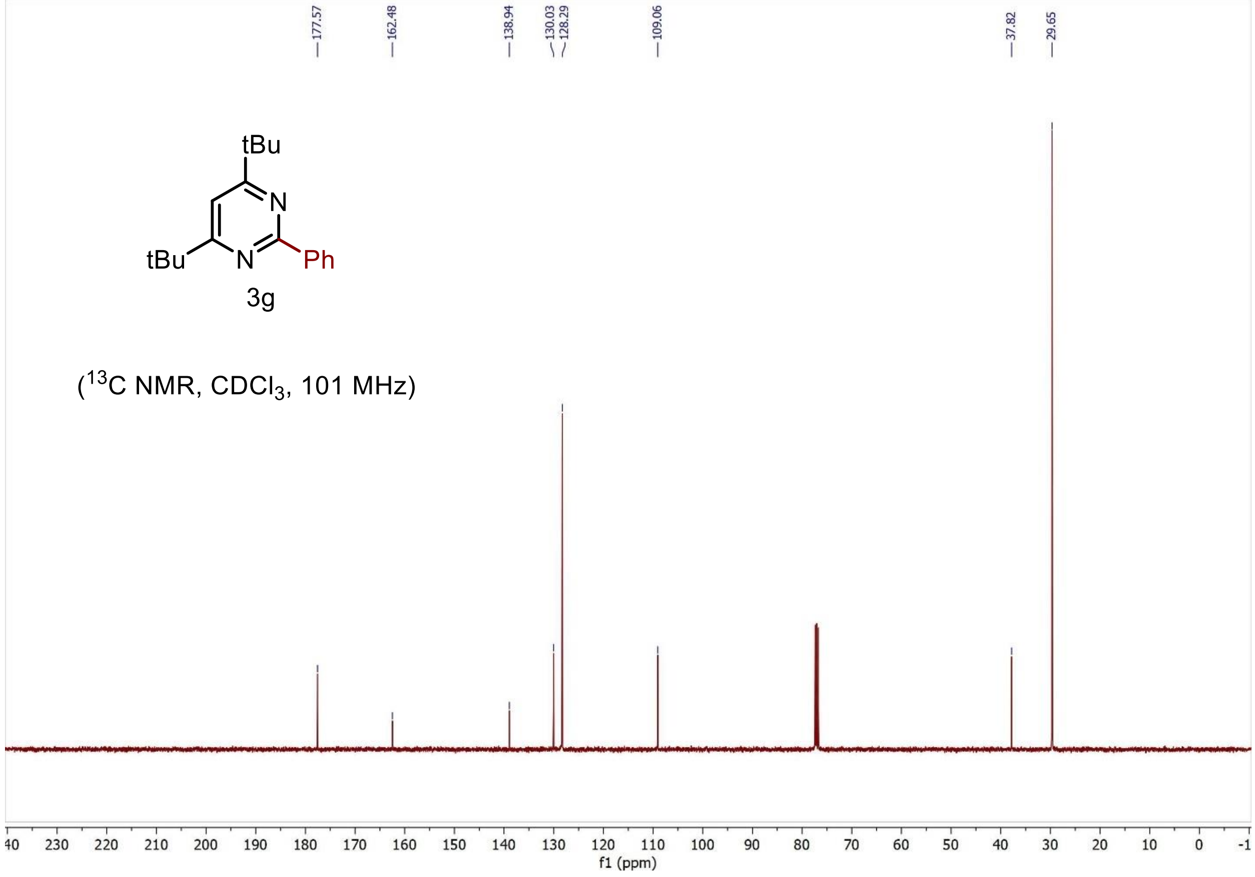

177.57
162.48
138.94
130.03
128.29
109.06
37.82
29.65
tBu
N
tBu
N
Ph
3g
(^{13}C NMR, $CDCl_3$, 101 MHz)
40 230 220 210 200 190 180 170 160 150 140 130 120 110 100 90 80 70 60 50 40 30 20 10 0 -1
f1 (ppm)

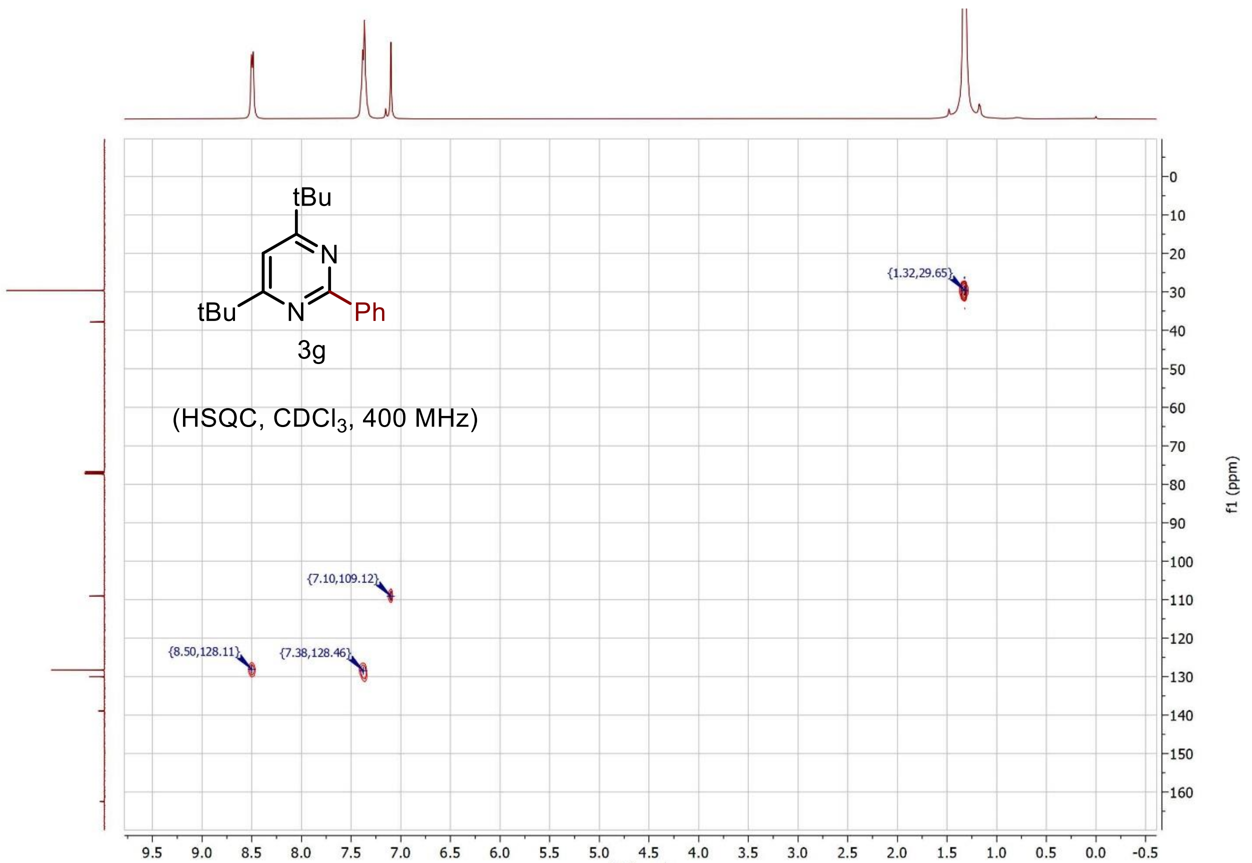

tBu
N
tBu
N
Ph
3g
(HSQC, CDCl$_3$, 400 MHz)
{1.32,29.65}
{7.10,109.12}
{8.50,128.11}
{7.38,128.46}
f1 (ppm)

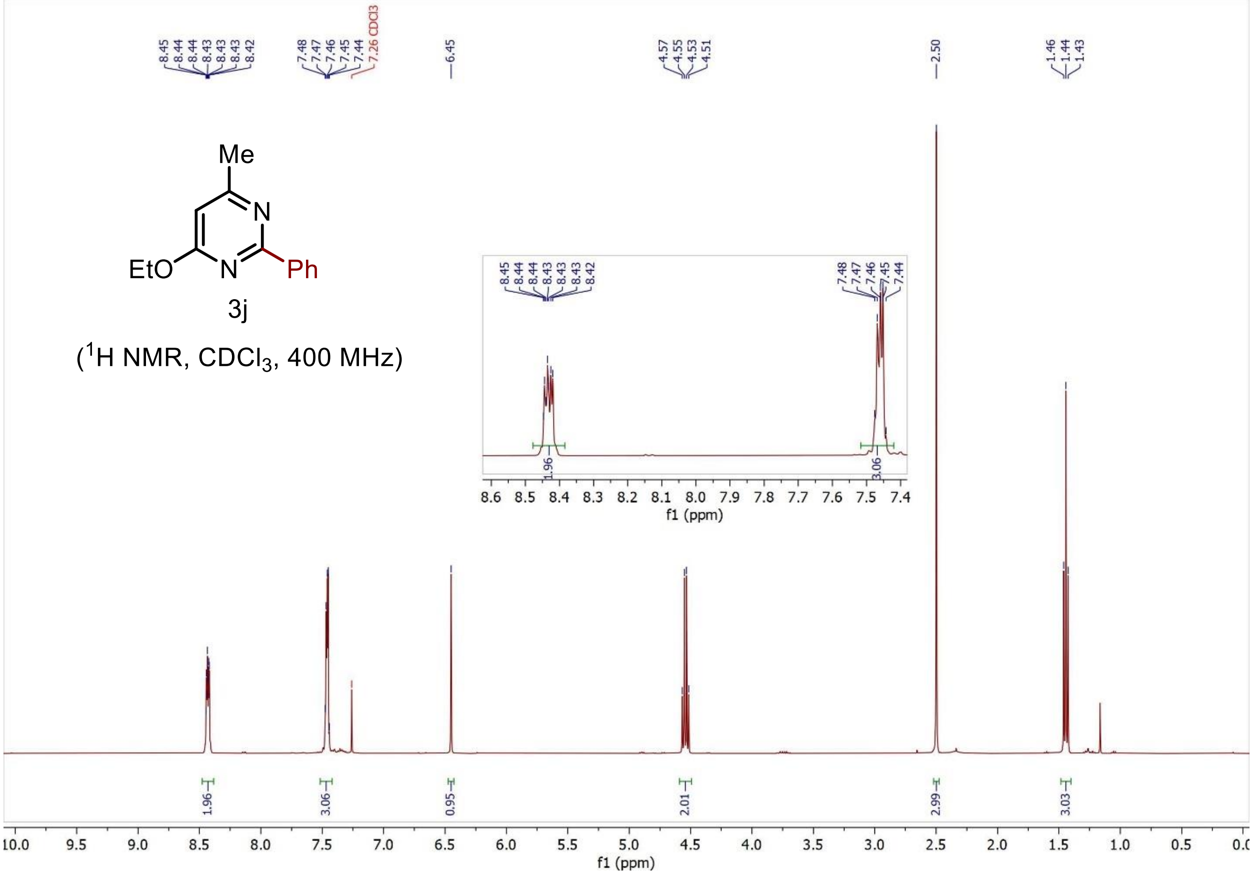

8.45 8.44 8.44 8.43 8.43 8.43 8.42
7.48 7.47 7.46 7.45 7.44
7.26 CDCl3
6.45
4.57 4.55 4.53 4.51
2.50
1.46 1.44 1.43
Me
N
EtO
N
Ph
3j
(^{1}H NMR, CDCl$_3$, 400 MHz)
1.96
3.06
0.95
2.01
2.99
3.03
f1 (ppm)

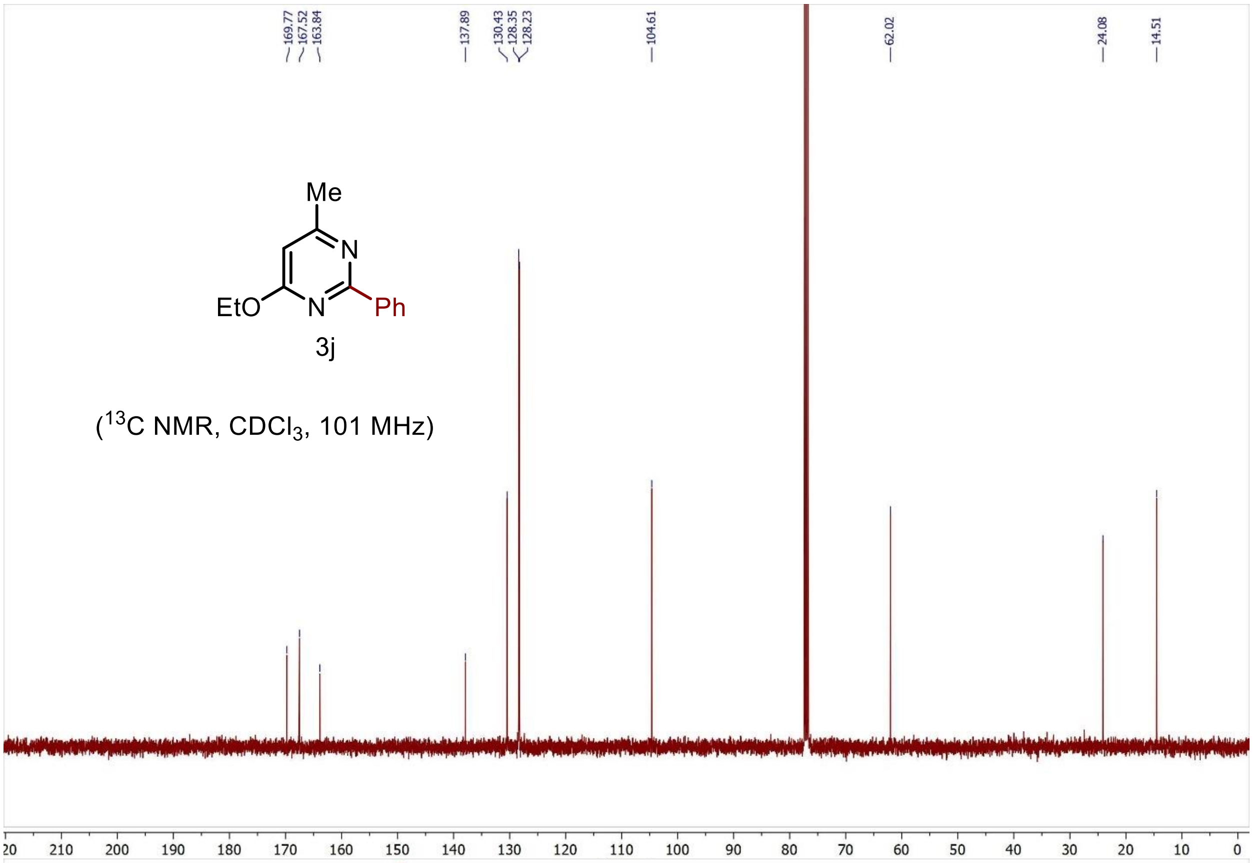
169.77
167.52
163.84
137.89
130.43
128.35
128.23
104.61
62.02
24.08
14.51
Me
N
EtO
N
Ph
3j
(13C NMR, CDCl3, 101 MHz)

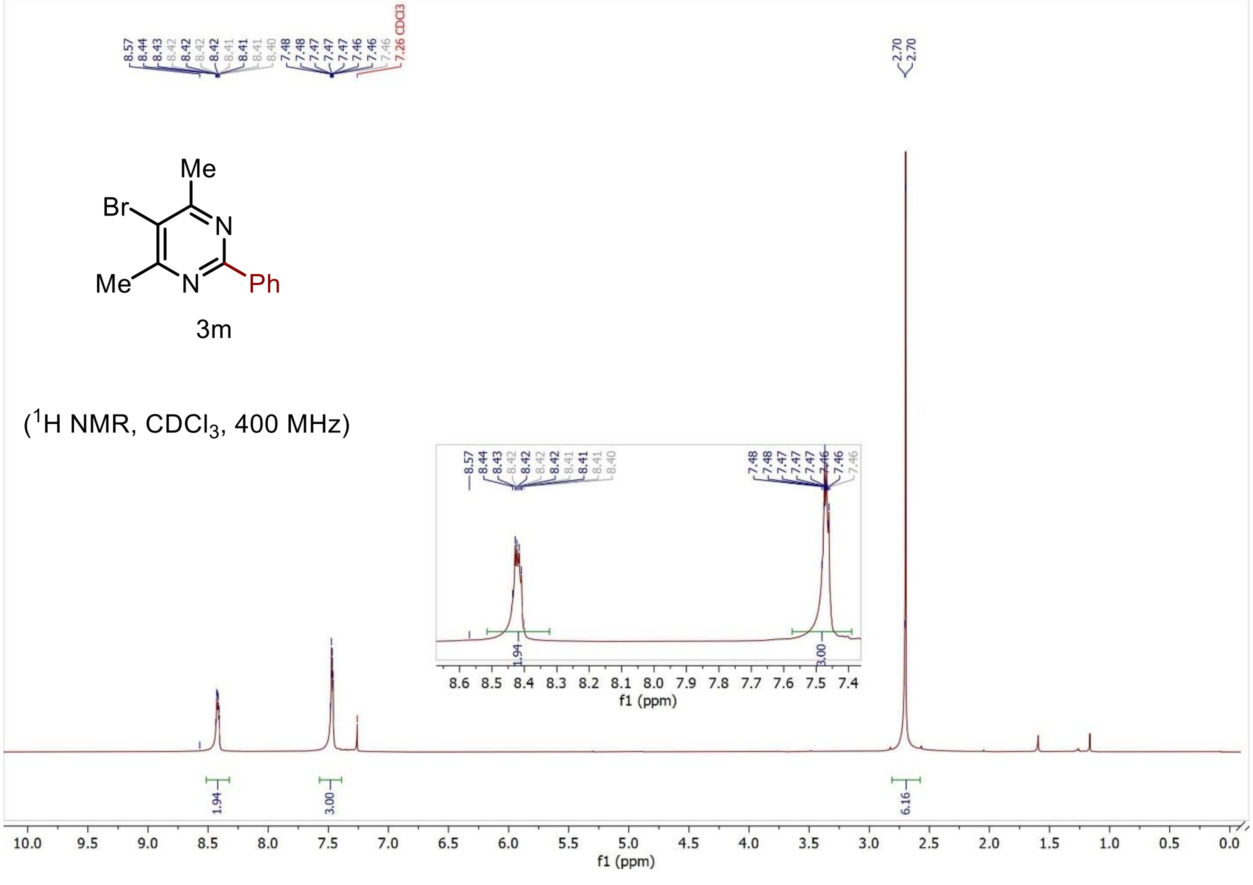
Me
Br
N
Me
N
Ph
3m
(1H NMR, CDCl3, 400 MHz)
7.26 CDCl3
2.70
2.70
1.94
3.00
6.16
f1 (ppm)

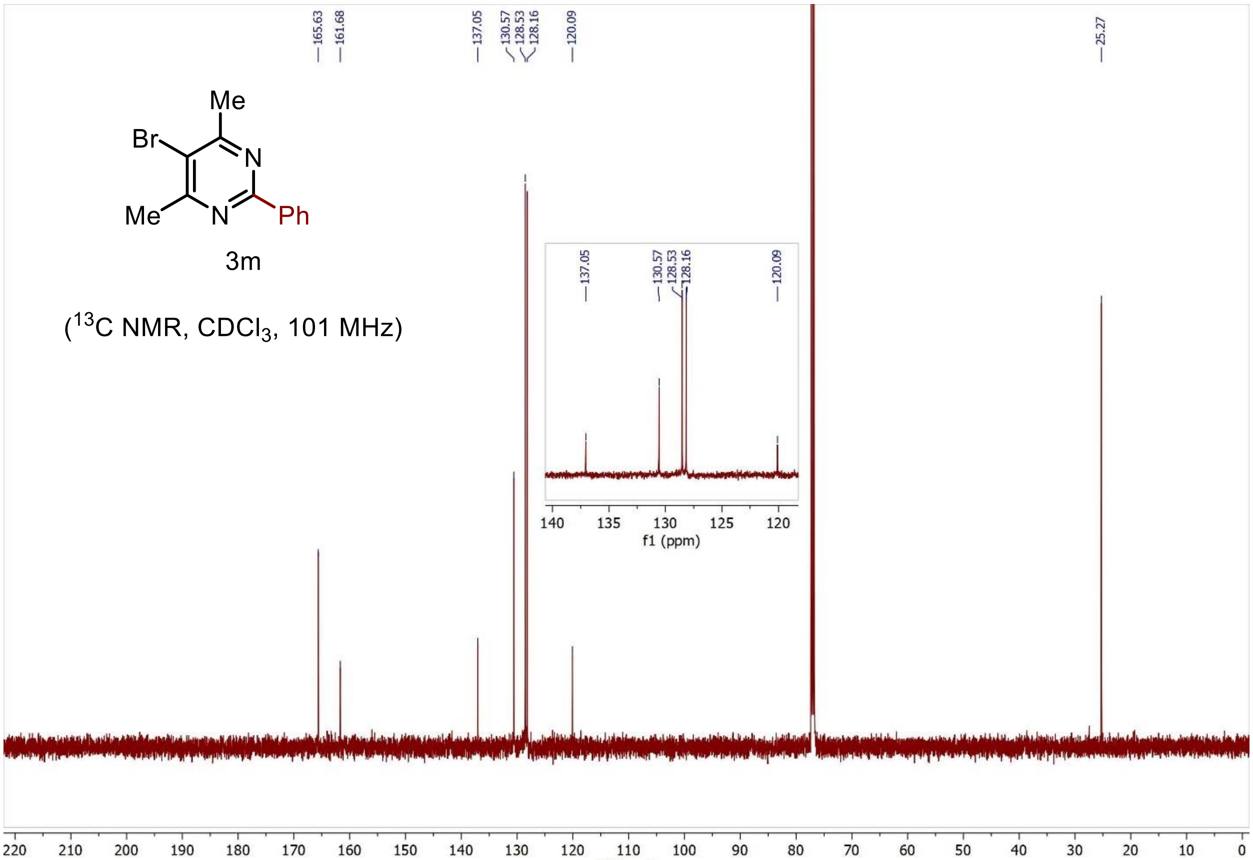

165.63
161.68
137.05
130.57
128.53
128.16
120.09
25.27
Me
Br
N
Me
N
Ph
3m
(13C NMR, CDCl3, 101 MHz)
f1 (ppm)

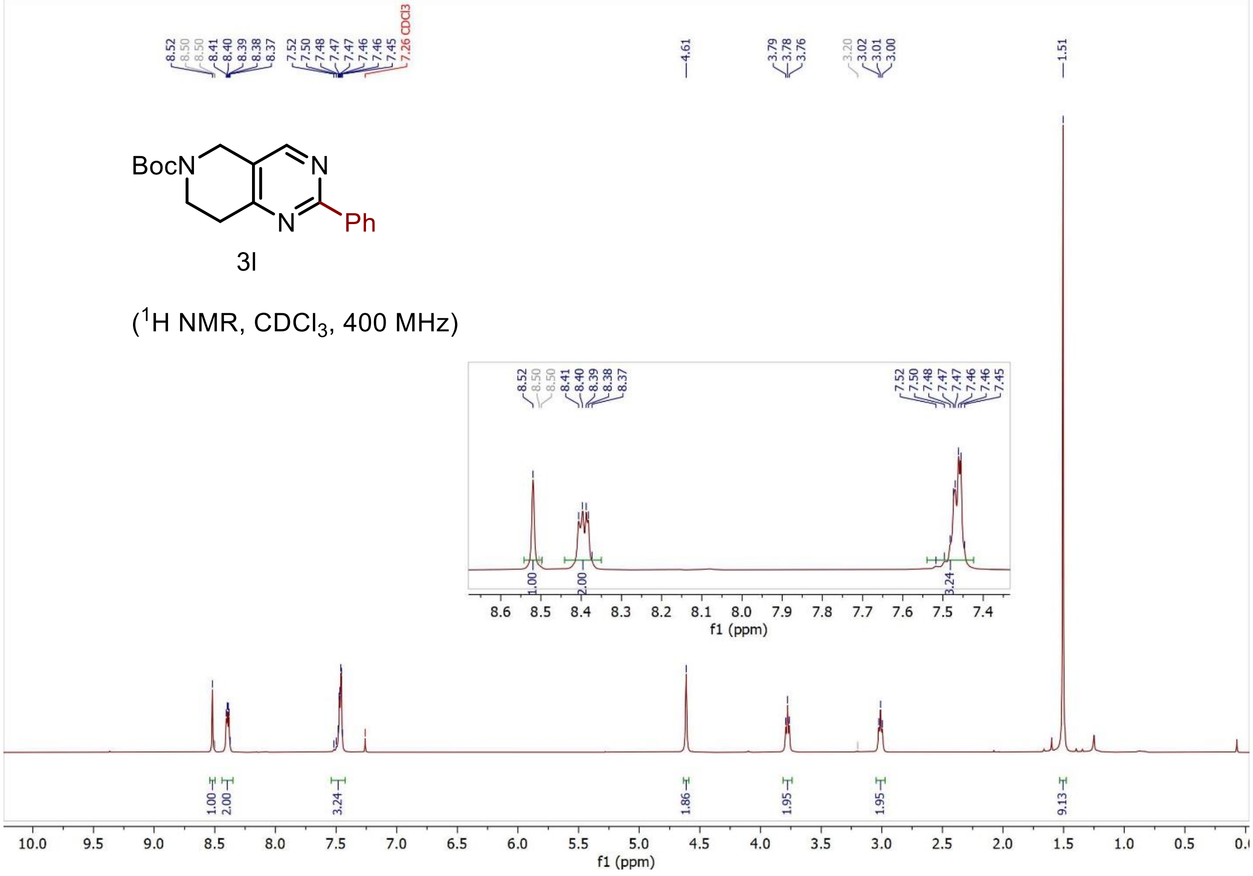

8.52
8.50
8.50
8.41
8.40
8.39
8.38
8.37
7.52
7.50
7.48
7.47
7.47
7.46
7.46
7.45
7.26 CDCl3
4.61
3.79
3.78
3.76
3.20
3.02
3.01
3.00
1.51
BocN
N
N
Ph
3l
(1H NMR, CDCl3, 400 MHz)
1.00
2.00
3.24
1.86
1.95
1.95
9.13
f1 (ppm)

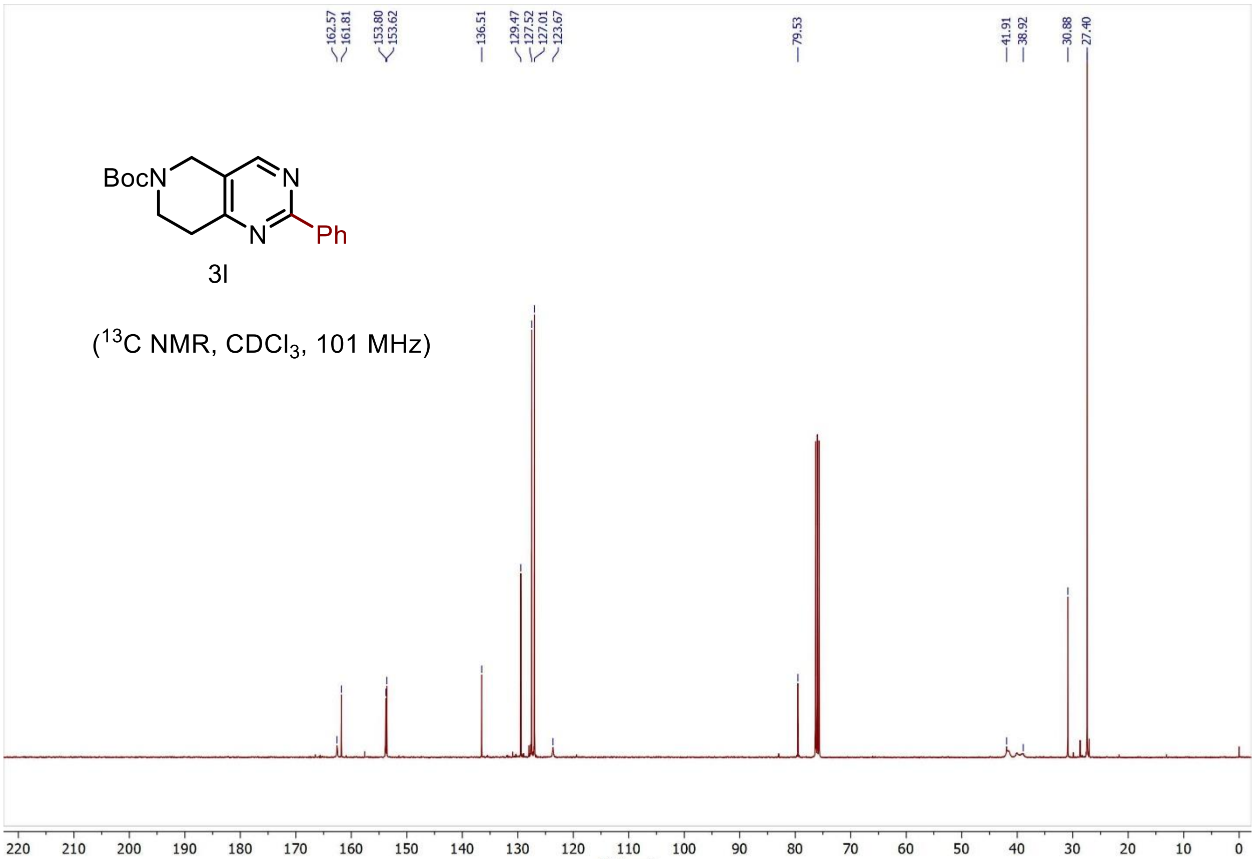

162.57
161.81
153.80
153.62
136.51
129.47
127.52
127.01
123.67
79.53
41.91
38.92
30.88
27.40
BocN
N
N
Ph
3l
(^{13}C NMR, $CDCl_3$, 101 MHz)
220 210 200 190 180 170 160 150 140 130 120 110 100 90 80 70 60 50 40 30 20 10 0

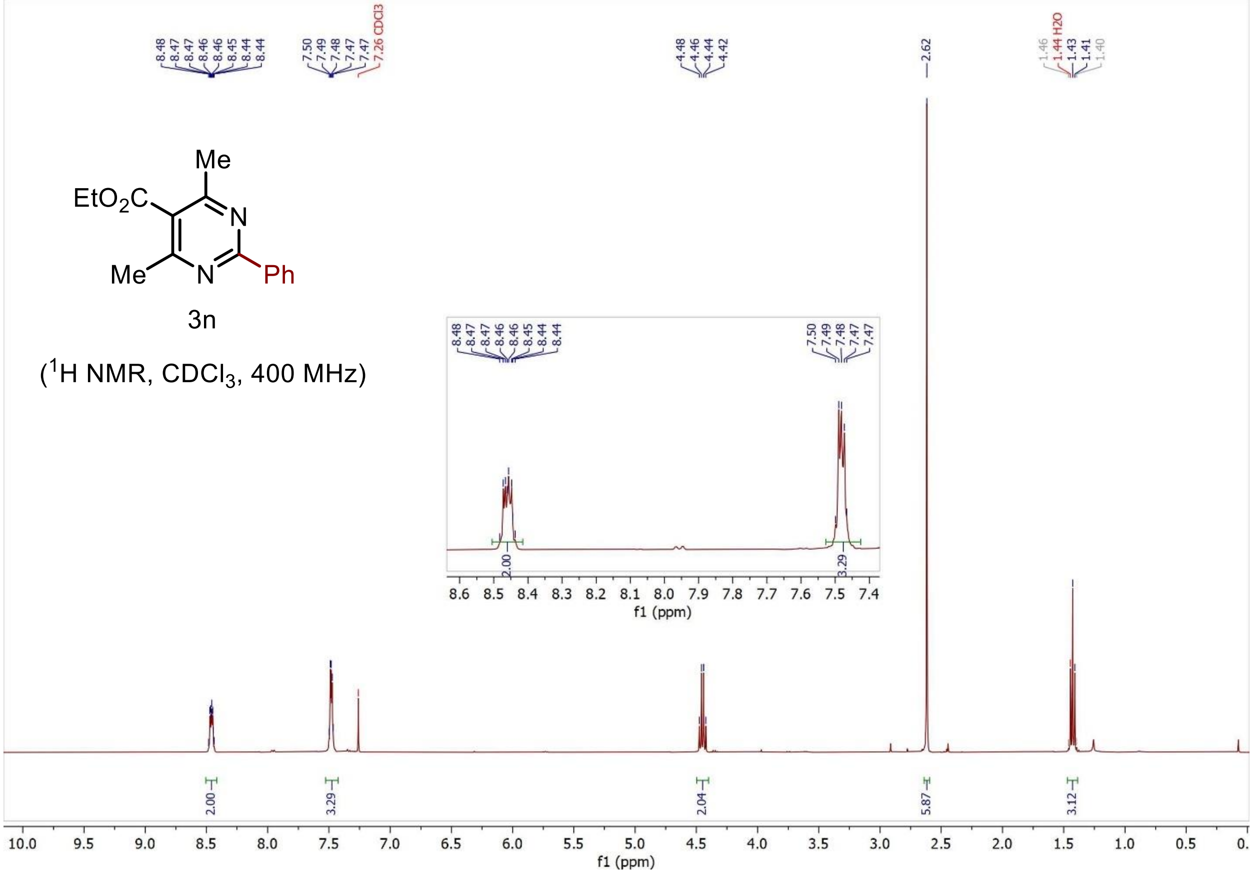

8.48 8.47 8.47 8.46 8.46 8.45 8.44 8.44
7.50 7.49 7.48 7.48 7.47 7.47
7.26 CDCl3
4.48 4.46 4.44 4.42
2.62
1.46 1.44 H2O 1.43 1.41 1.40
Me
EtO_2C
N
Me
N
Ph
3n
(^{1}H NMR, $CDCl_3$, 400 MHz)
2.00
3.29
8.6 8.5 8.4 8.3 8.2 8.1 8.0 7.9 7.8 7.7 7.6 7.5 7.4
f1 (ppm)
2.00
3.29
2.04
5.87
3.12
10.0 9.5 9.0 8.5 8.0 7.5 7.0 6.5 6.0 5.5 5.0 4.5 4.0 3.5 3.0 2.5 2.0 1.5 1.0 0.5 0.
f1 (ppm)

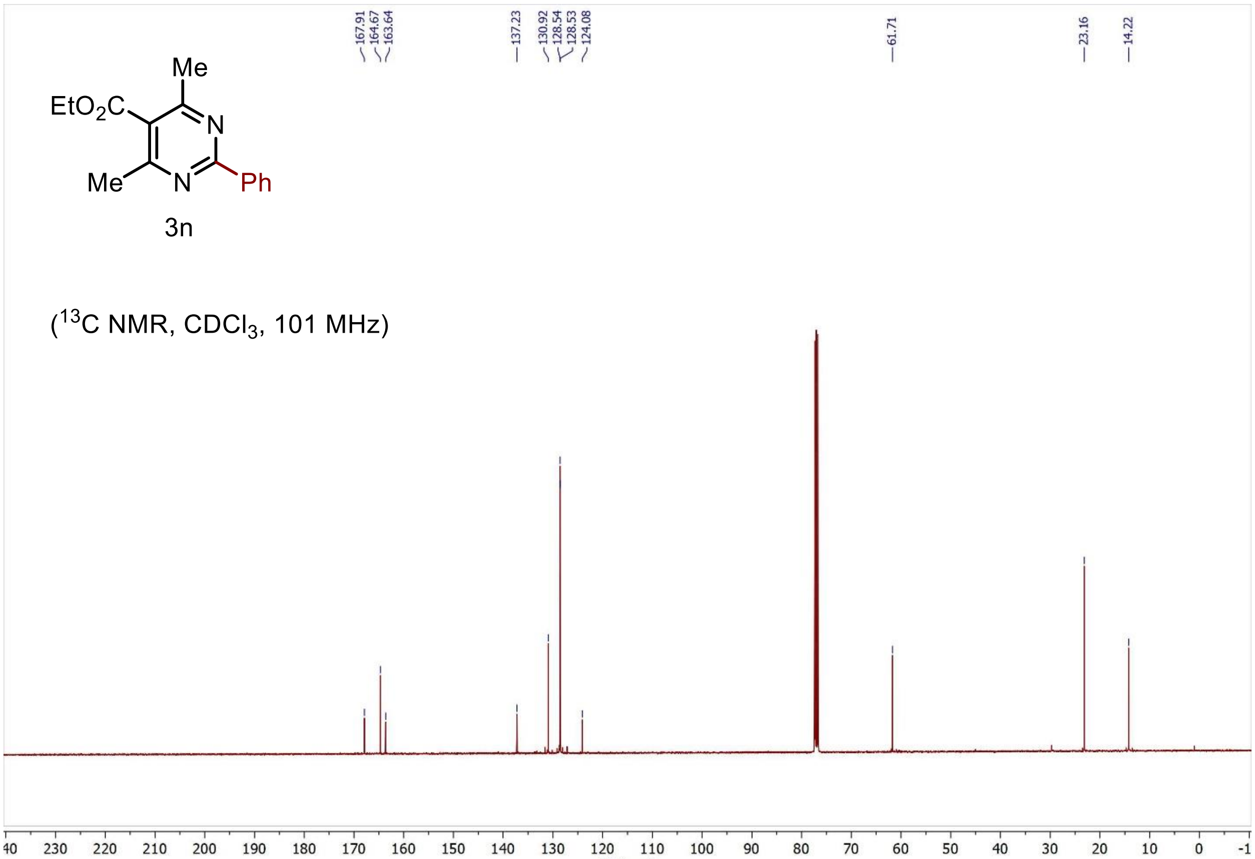

EtO2C
Me
N
Me
N
Ph
3n
(13C NMR, CDCl3, 101 MHz)
167.91
164.67
163.64
137.23
130.92
128.54
128.53
124.08
61.71
23.16
14.22

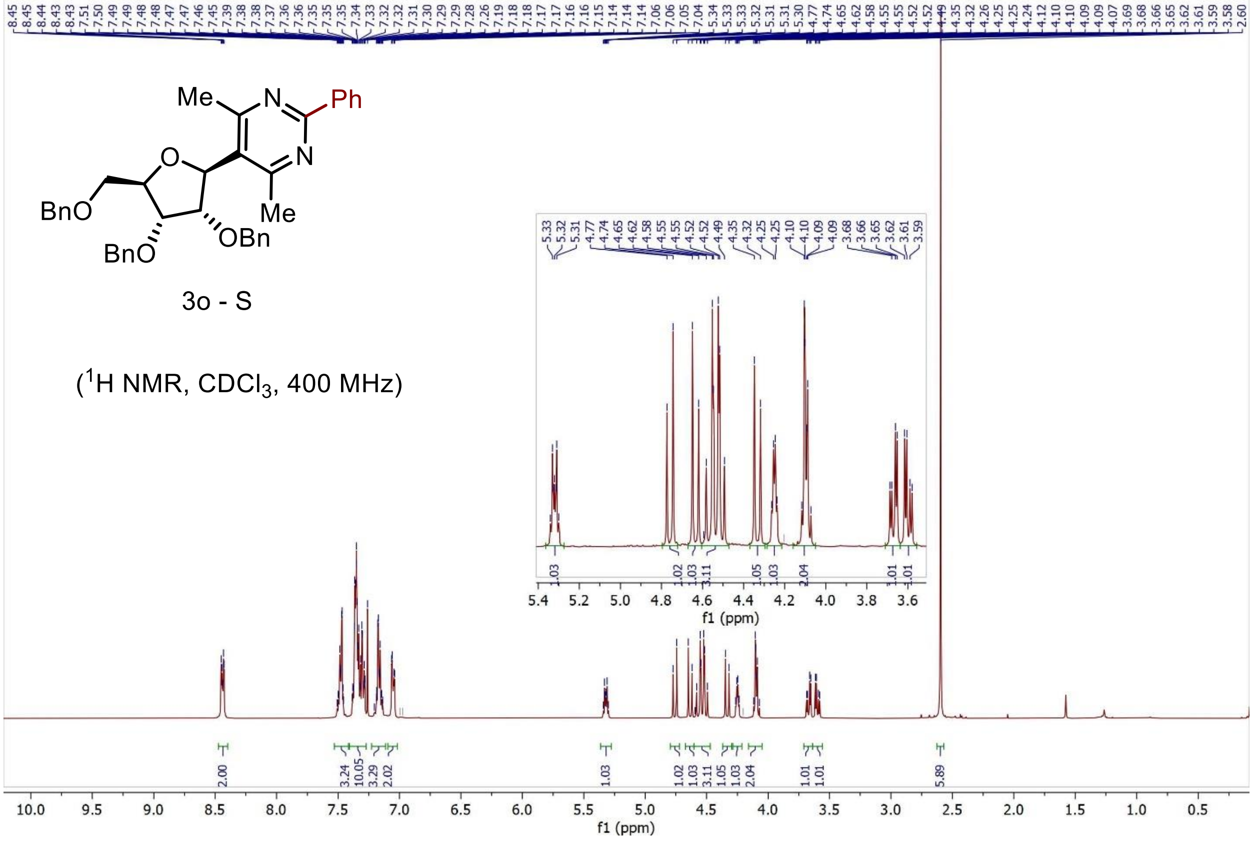

Me
N
Ph
N
Me
O
BnO
BnO
OBn
3o - S
(1H NMR, CDCl3, 400 MHz)
f1 (ppm)

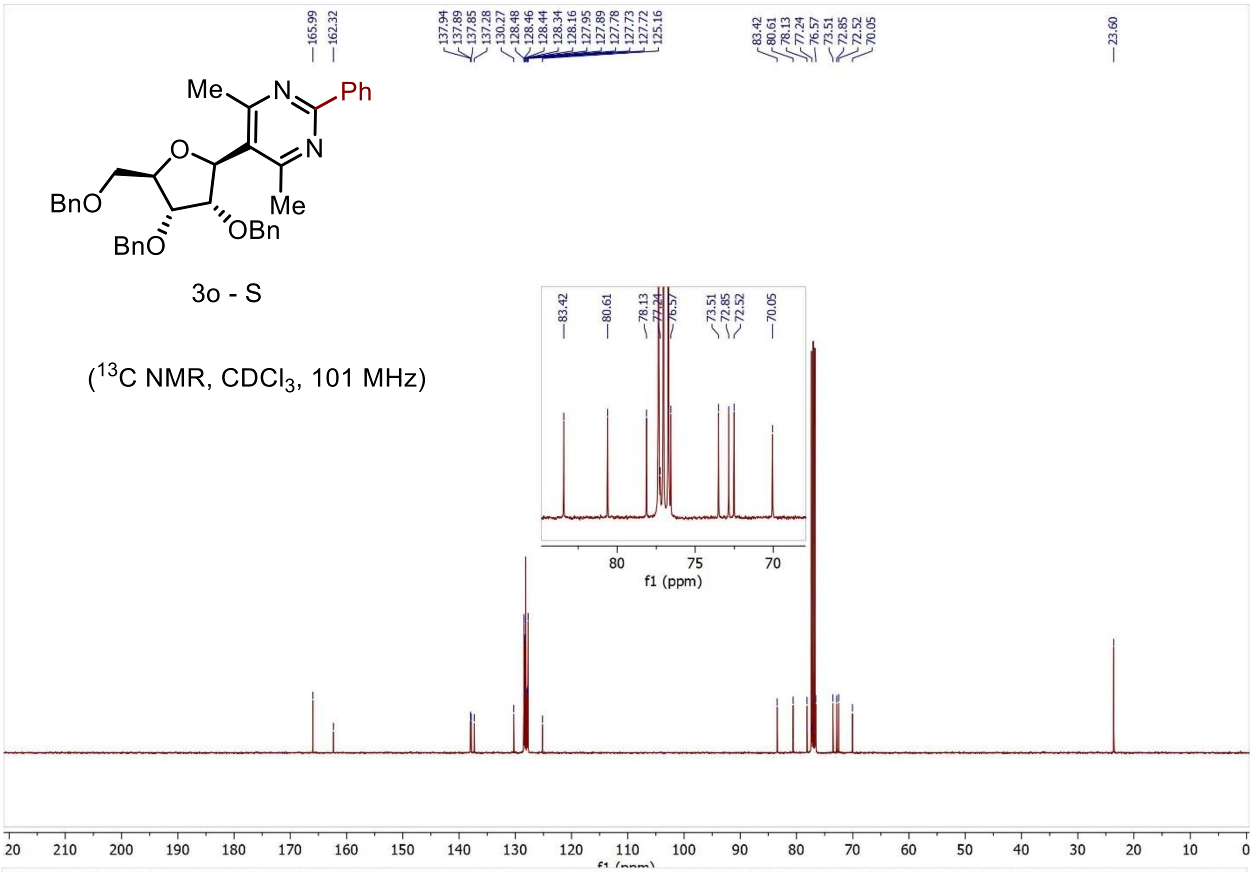
3o - S
(^{13}C NMR, CDCl$_3$, 101 MHz)
f1 (ppm)

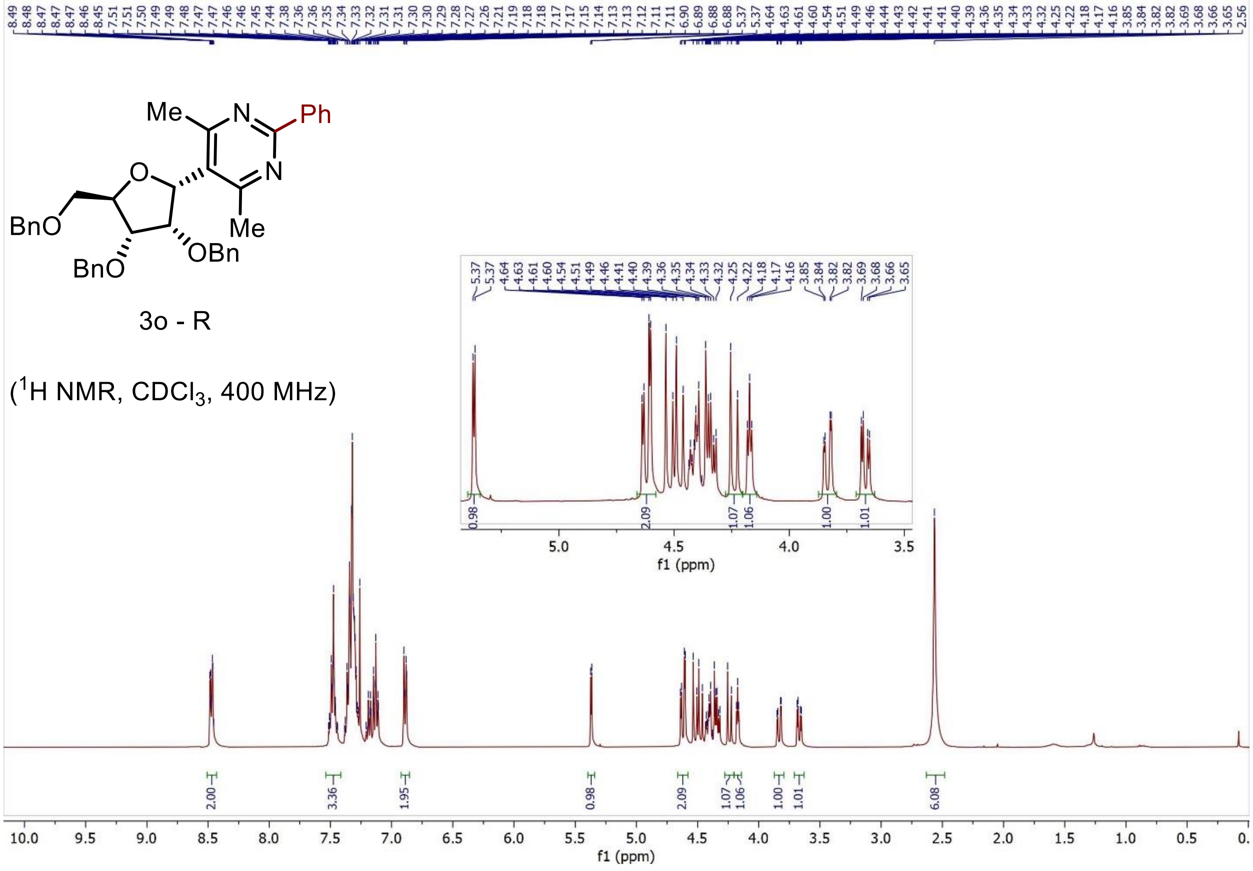
3o - R
(^{1}H NMR, CDCl$_3$, 400 MHz)
f1 (ppm)

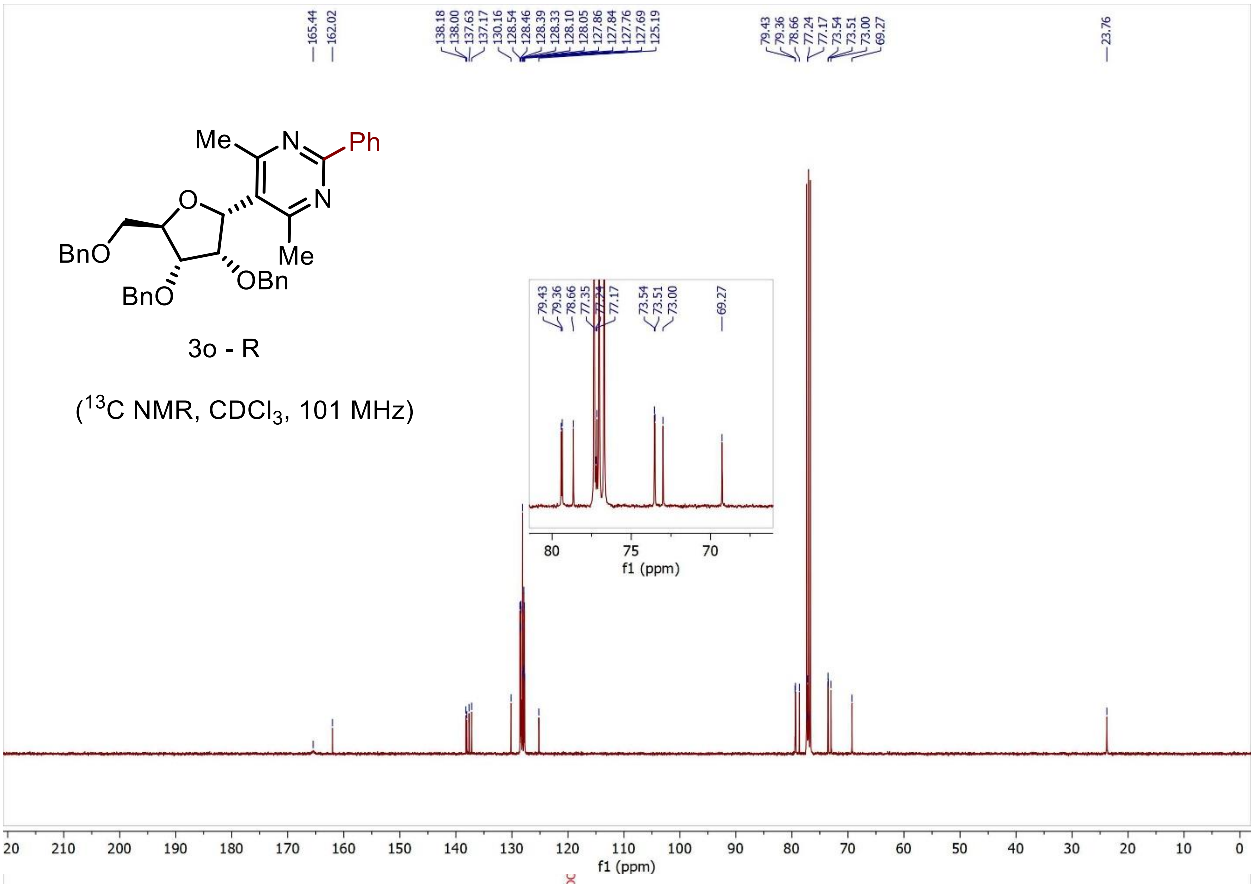

3o - R

(^{13}C NMR, $CDCl_3$, 101 MHz)

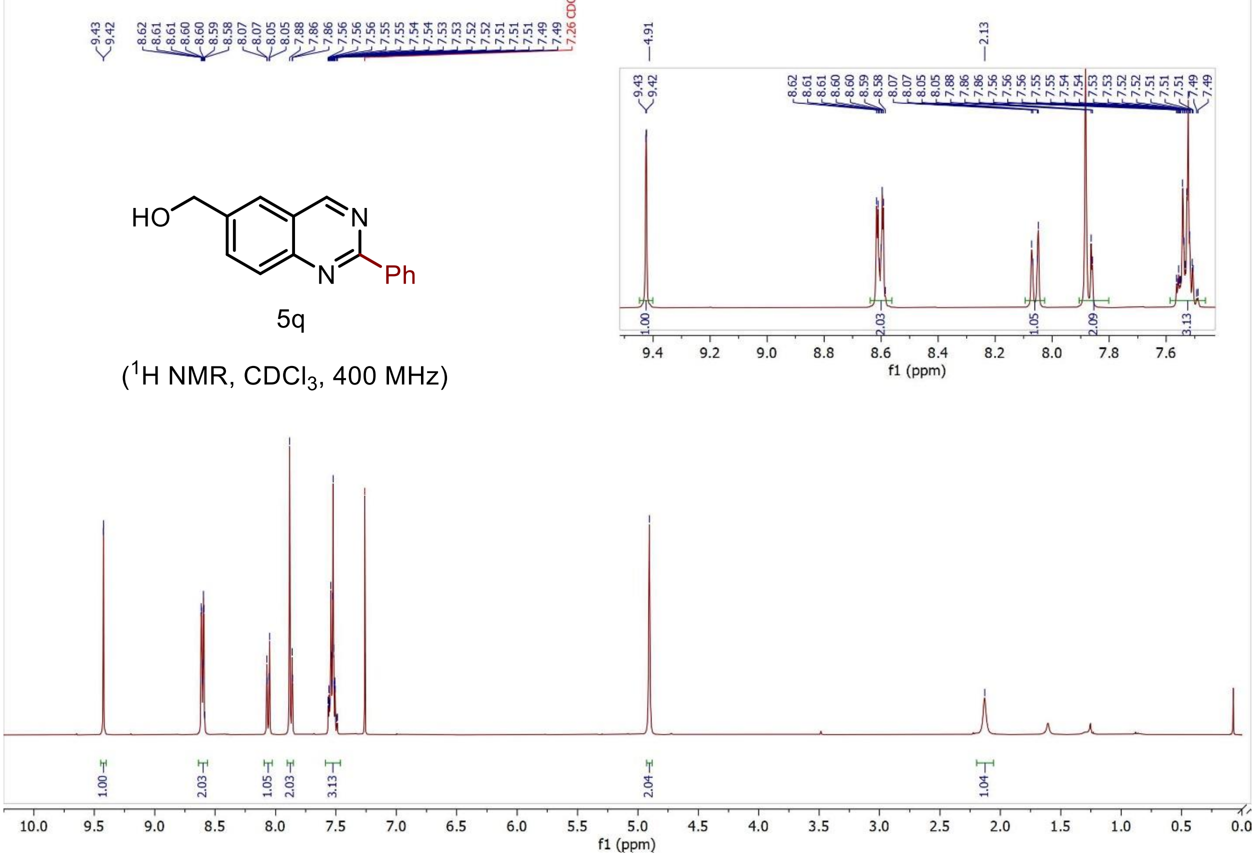

5q

(^{1}H NMR, $CDCl_3$, 400 MHz)

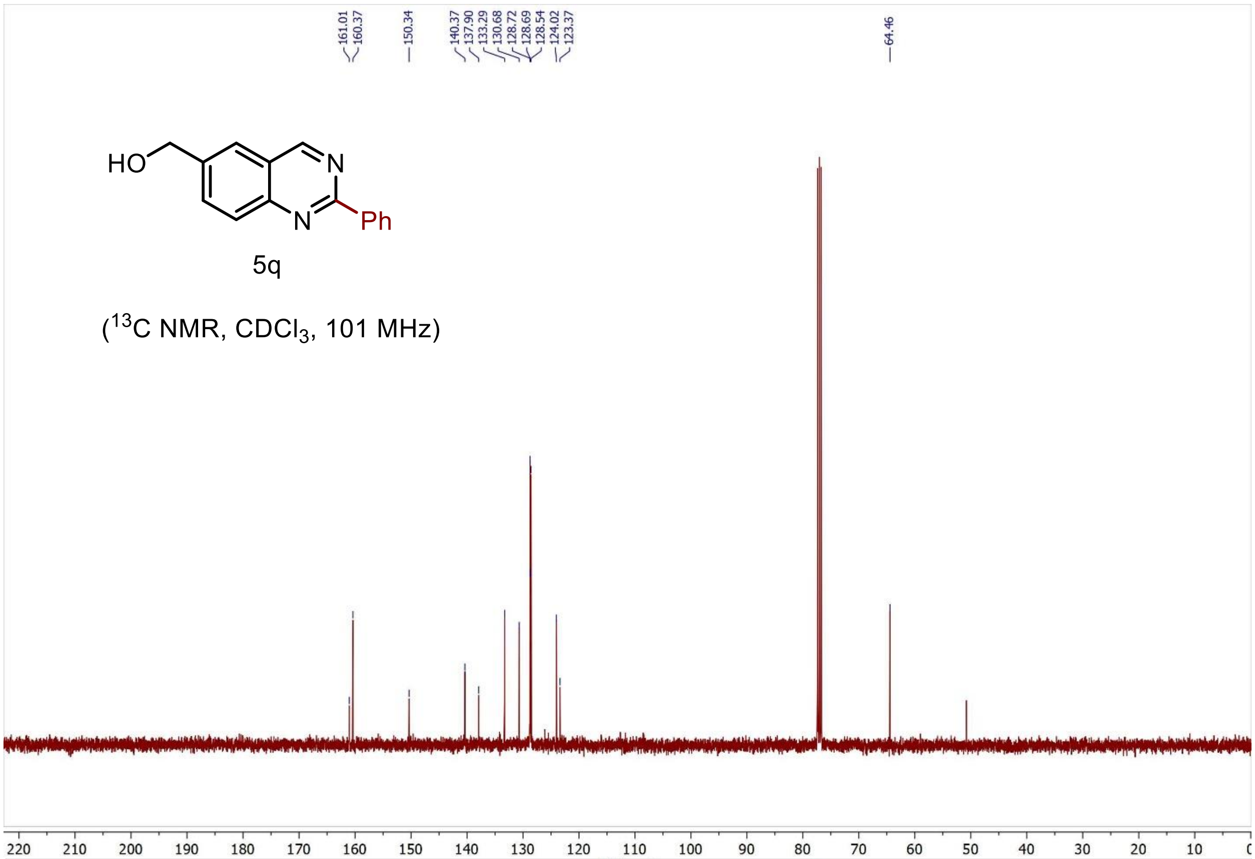
HO
N
N
Ph
5q
(13C NMR, CDCl3, 101 MHz)
161.01
160.37
150.34
140.37
137.90
133.29
130.68
128.72
128.69
128.54
124.02
123.37
64.46

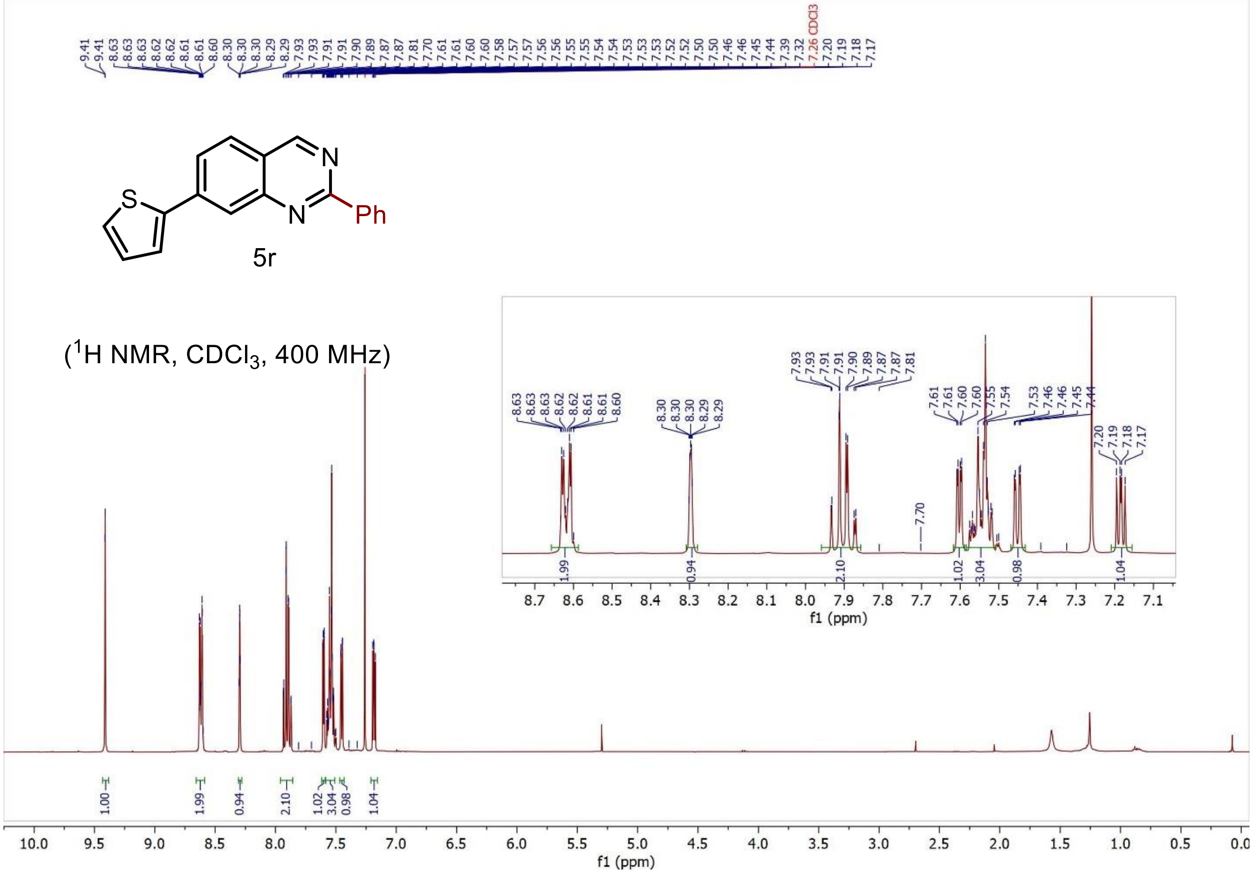
S
N
N
Ph
5r
(1H NMR, CDCl3, 400 MHz)
7.26 CDCl3
f1 (ppm)

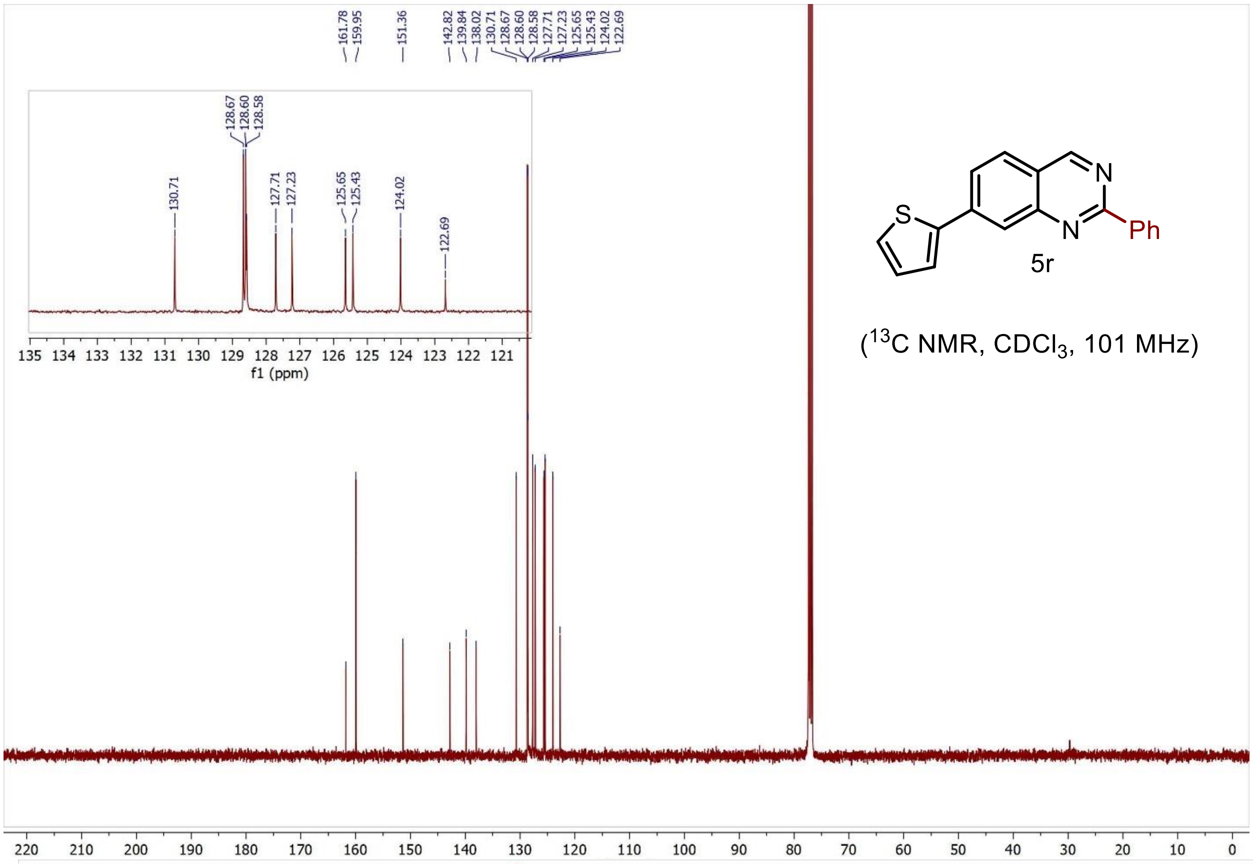

5r

(^{13}C NMR, $CDCl_3$, 101 MHz)

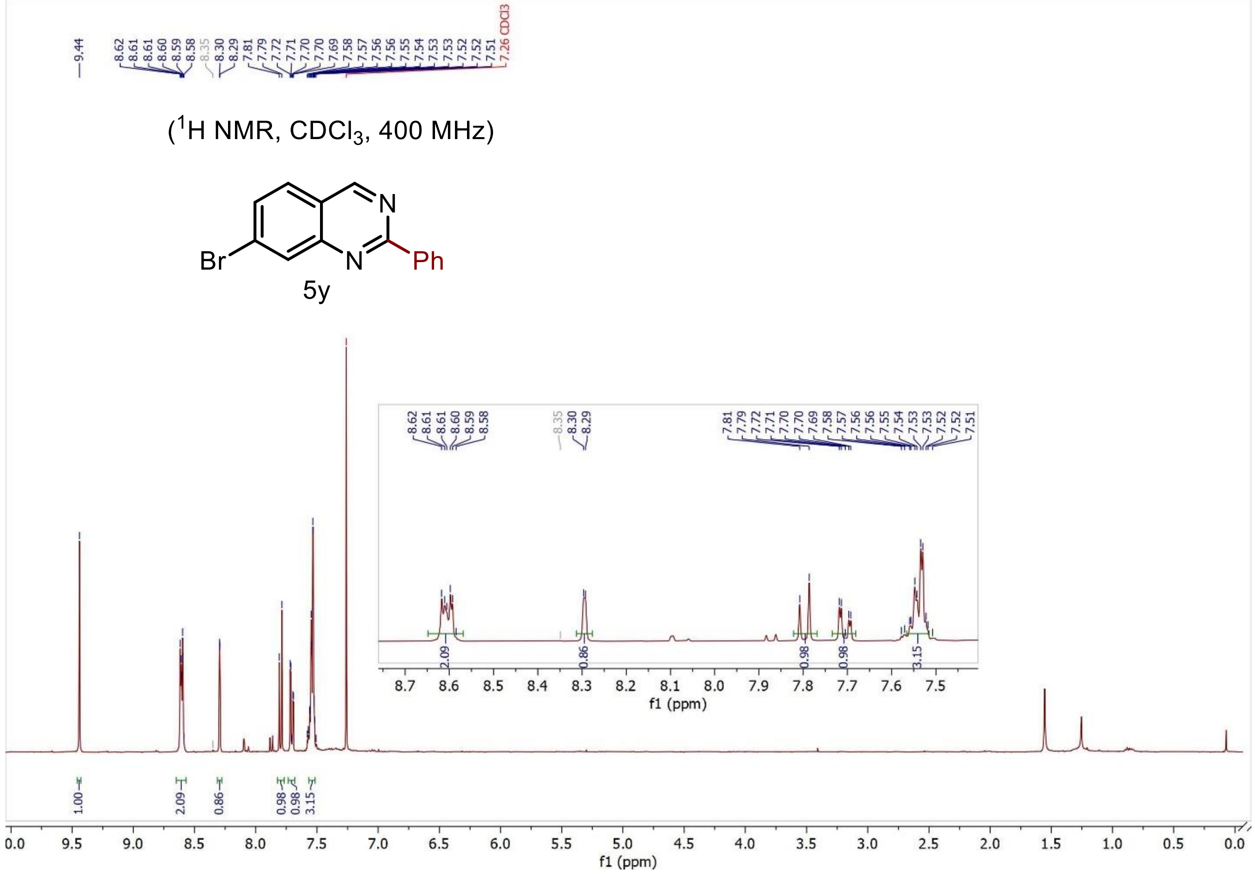

(^{1}H NMR, $CDCl_3$, 400 MHz)

5y

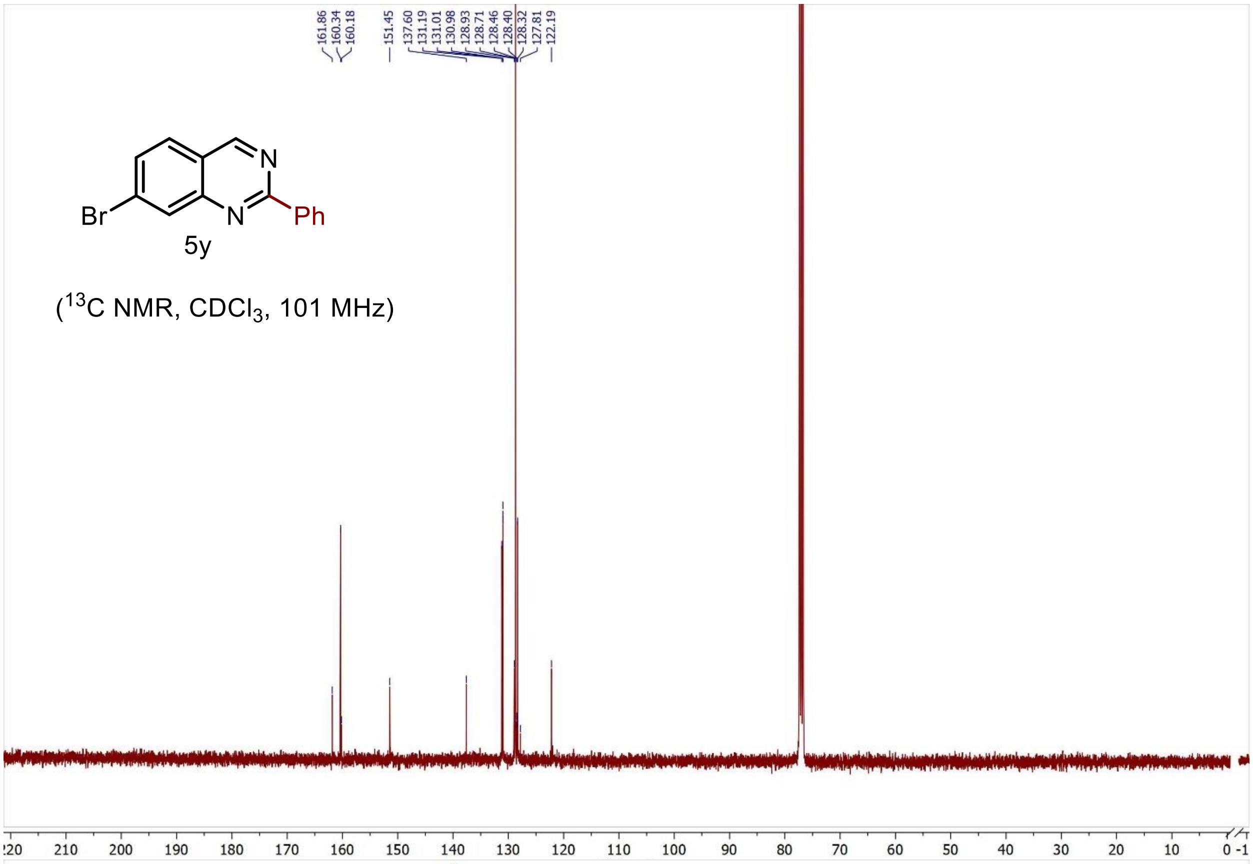
161.86
160.34
160.18
151.45
137.60
131.19
131.01
130.98
128.93
128.71
128.46
128.40
128.32
127.81
122.19
Br
N
N
Ph
5y
(13C NMR, CDCl3, 101 MHz)

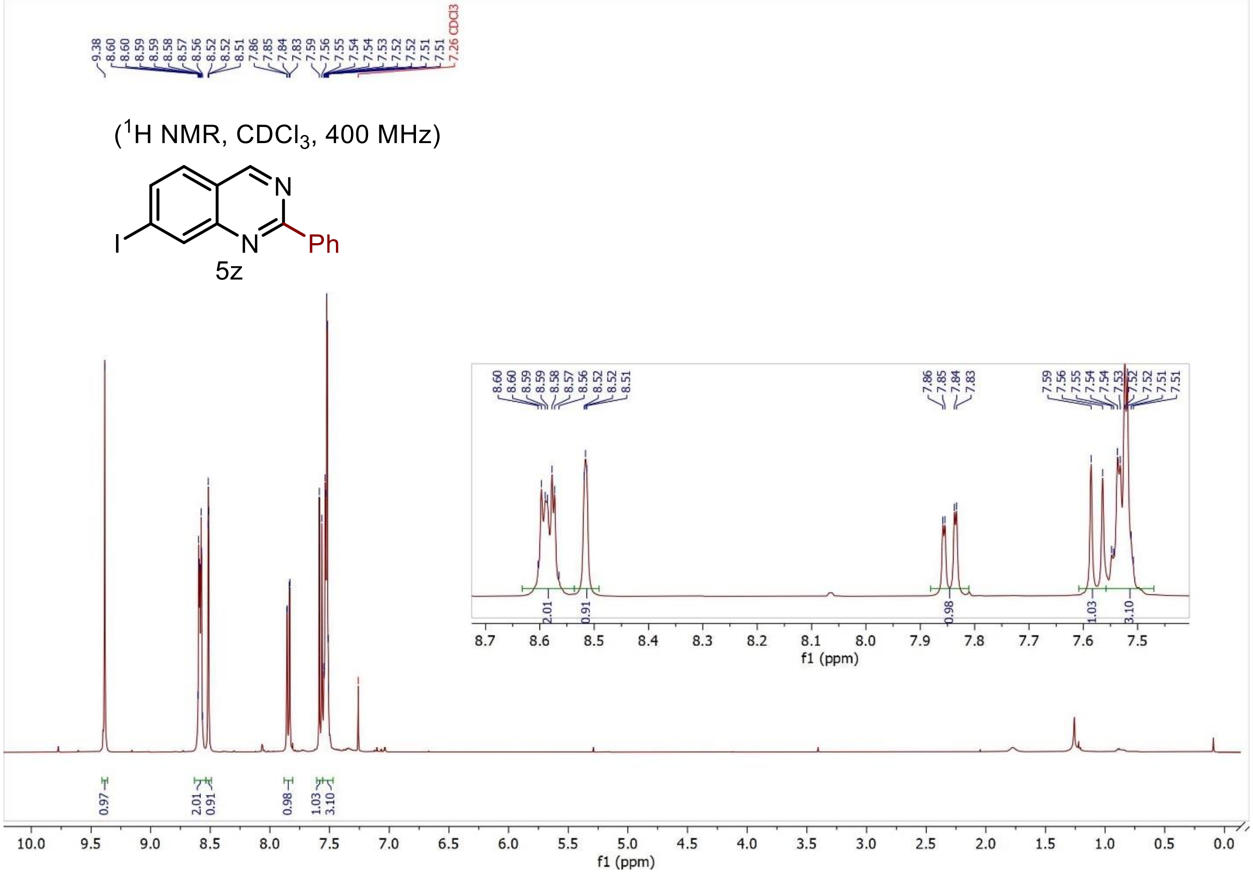
(1H NMR, CDCl3, 400 MHz)
I
N
N
Ph
5z
f1 (ppm)

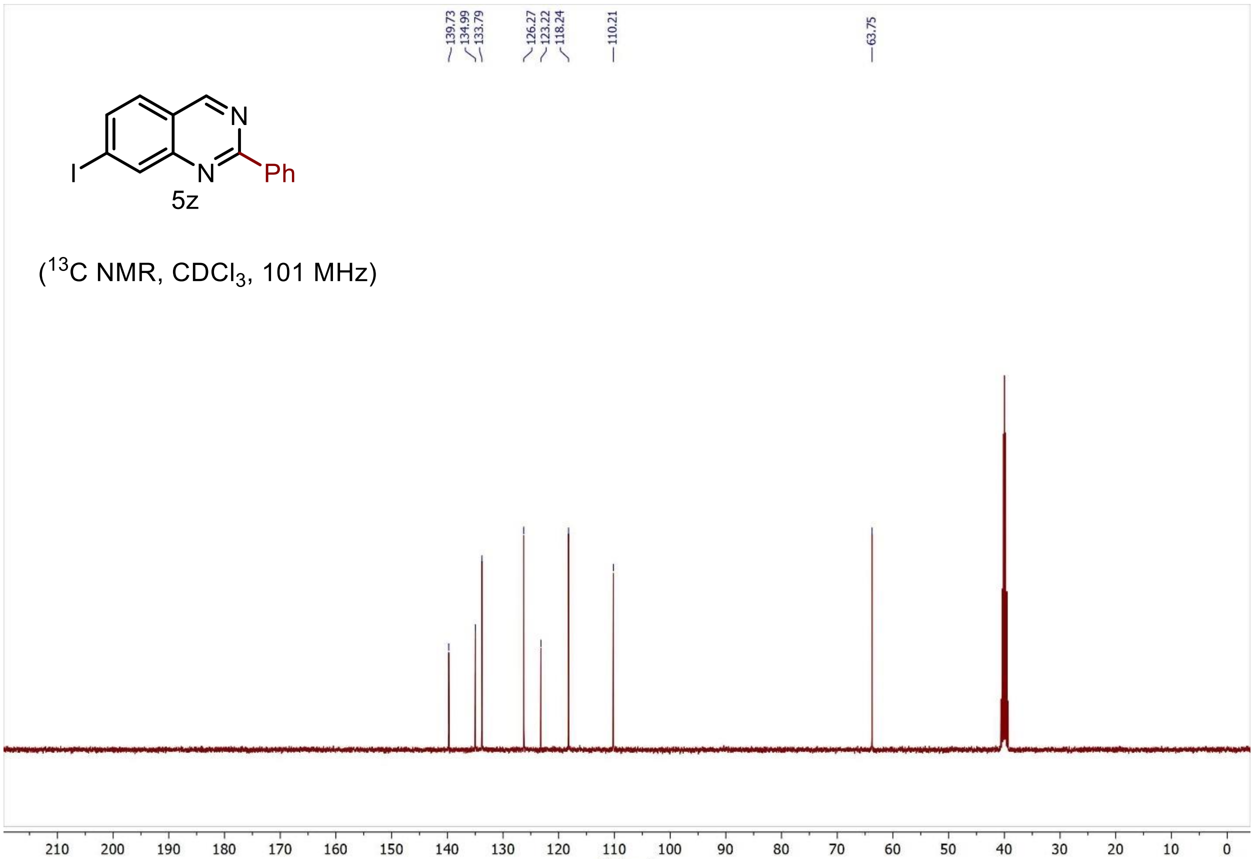
139.73
134.99
133.79
126.27
123.22
118.24
110.21
63.75
I
N
N
Ph
5z
(^{13}C NMR, CDCl$_3$, 101 MHz)
210 200 190 180 170 160 150 140 130 120 110 100 90 80 70 60 50 40 30 20 10 0

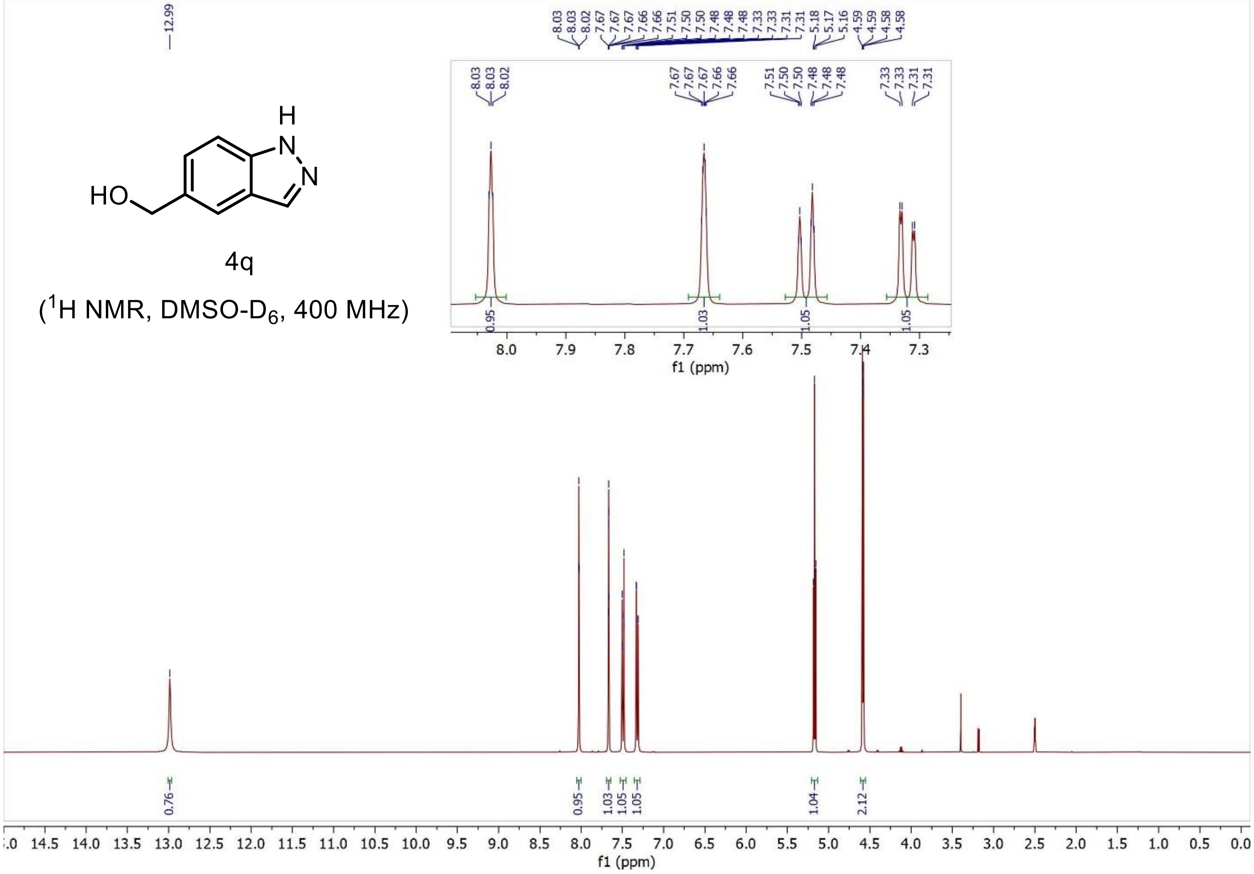
12.99
8.03 8.03 8.02 7.67 7.67 7.67 7.66 7.66 7.51 7.50 7.50 7.48 7.48 7.48 7.33 7.33 7.31 7.31 5.18 5.17 5.16 4.59 4.59 4.58 4.58
H
N
N
HO
4q
(^{1}H NMR, DMSO-D$_6$, 400 MHz)
0.95
1.03
1.05
1.05
8.0 7.9 7.8 7.7 7.6 7.5 7.4 7.3
f1 (ppm)
0.76
0.95
1.03
1.05
1.05
1.04
2.12
14.5 14.0 13.5 13.0 12.5 12.0 11.5 11.0 10.5 10.0 9.5 9.0 8.5 8.0 7.5 7.0 6.5 6.0 5.5 5.0 4.5 4.0 3.5 3.0 2.5 2.0 1.5 1.0 0.5 0.0
f1 (ppm)

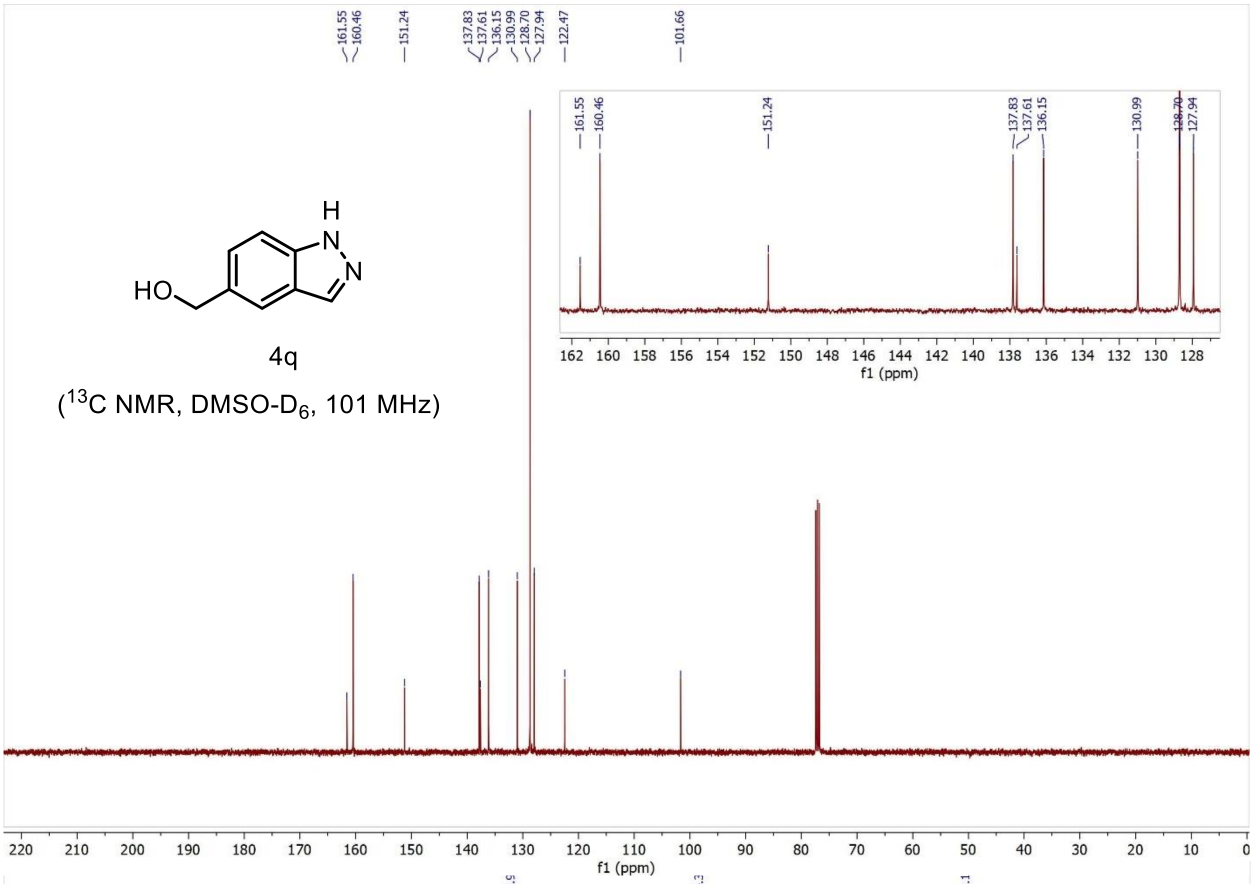
161.55
160.46
151.24
137.83
137.61
136.15
130.99
128.70
127.94
122.47
101.66
HO
H
N
N
4q
(^{13}C NMR, DMSO-D$_6$, 101 MHz)
f1 (ppm)

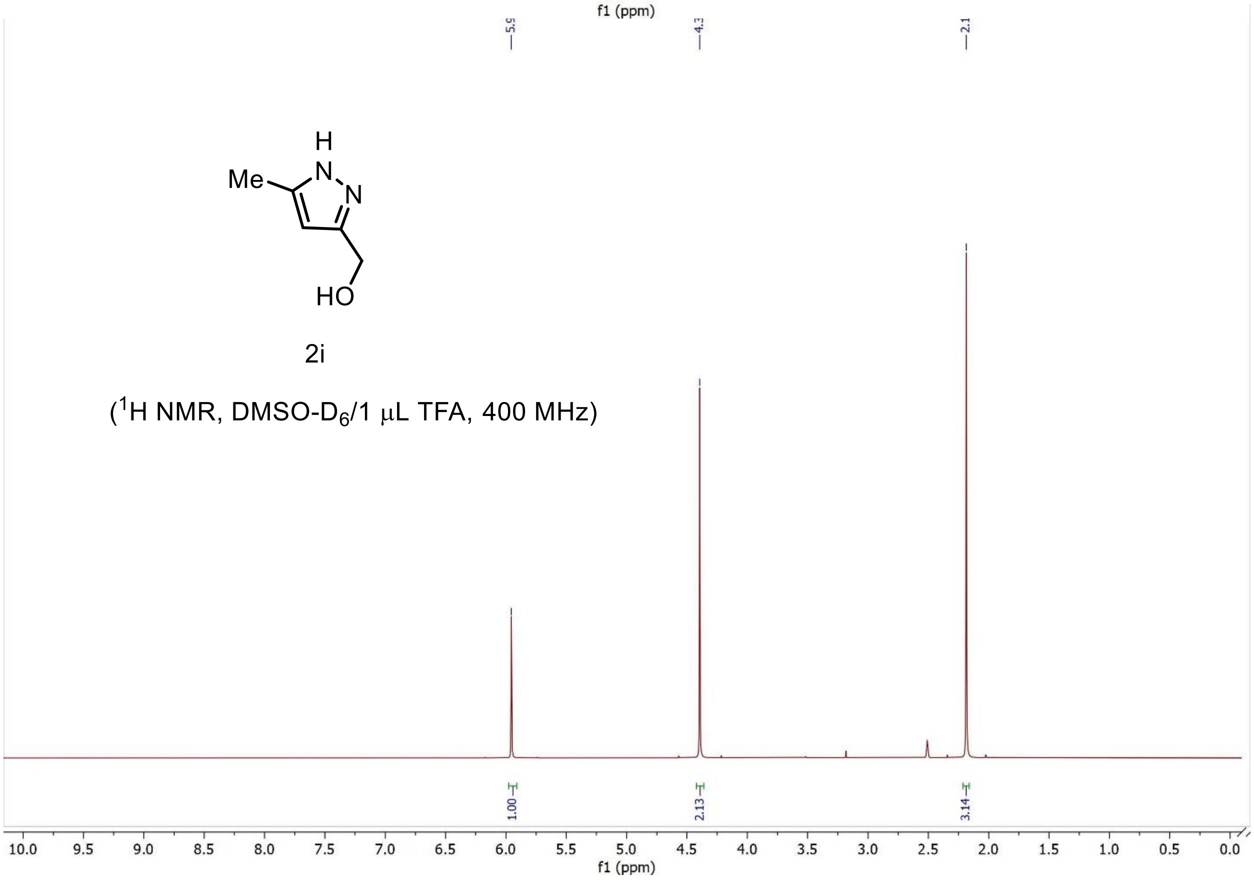
5.9
4.3
2.1
H
N
N
Me
HO
2i
(^{1}H NMR, DMSO-D$_6$/1 μL TFA, 400 MHz)
1.00
2.13
3.14
f1 (ppm)

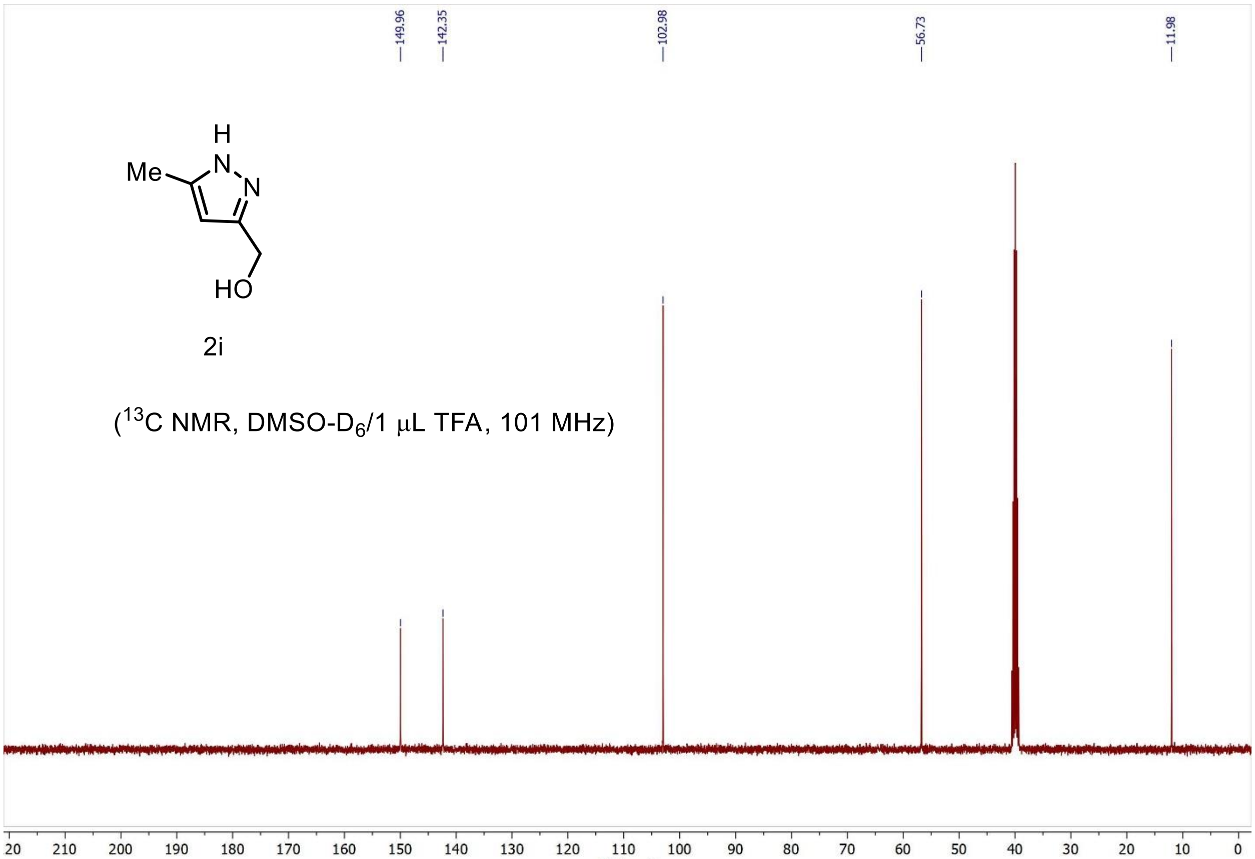
149.96
142.35
102.98
56.73
11.98
Me
H
N
N
HO
2i
(^{13}C NMR, DMSO-D$_6$/1 μL TFA, 101 MHz)

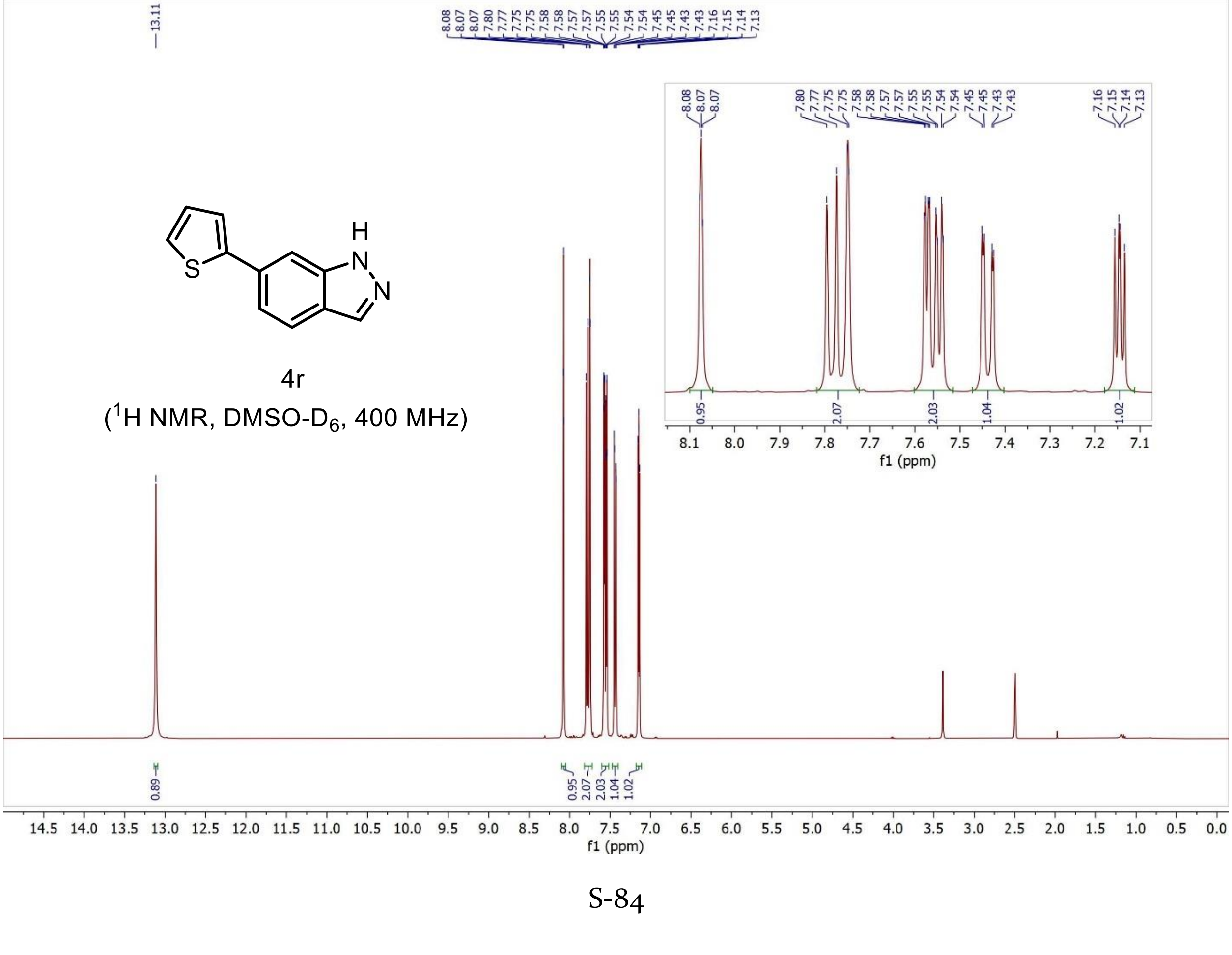
13.11
8.08 8.07 8.07 7.80 7.77 7.75 7.75 7.58 7.58 7.57 7.57 7.55 7.55 7.54 7.54 7.45 7.45 7.43 7.43 7.16 7.15 7.14 7.13
S
H
N
N
4r
(^{1}H NMR, DMSO-D$_6$, 400 MHz)
0.95 2.07 2.03 1.04 1.02
f1 (ppm)
0.89
f1 (ppm)

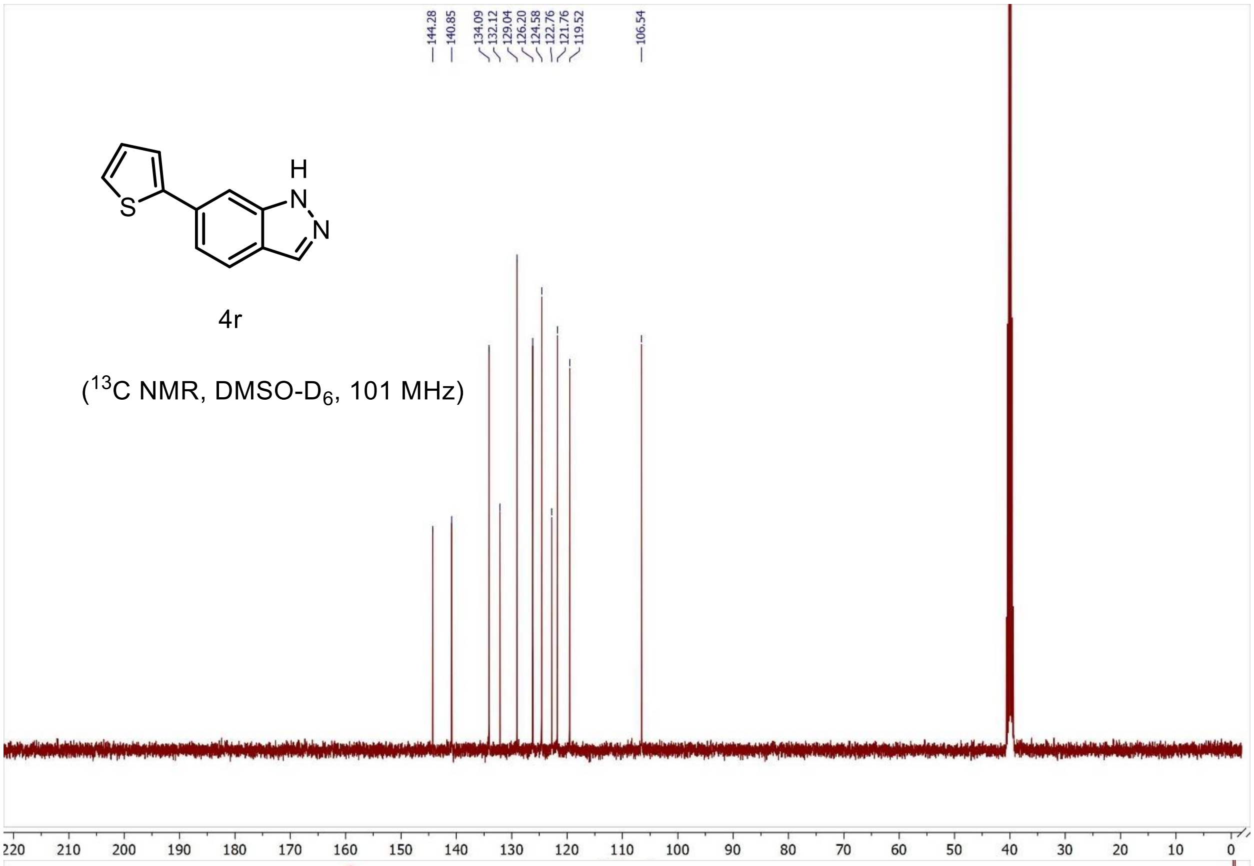

144.28
140.85
134.09
132.12
129.04
126.20
124.58
122.76
121.76
119.52
106.54
H
N
N
S
4r
(^{13}C NMR, DMSO-D$_6$, 101 MHz)
220 210 200 190 180 170 160 150 140 130 120 110 100 90 80 70 60 50 40 30 20 10 0

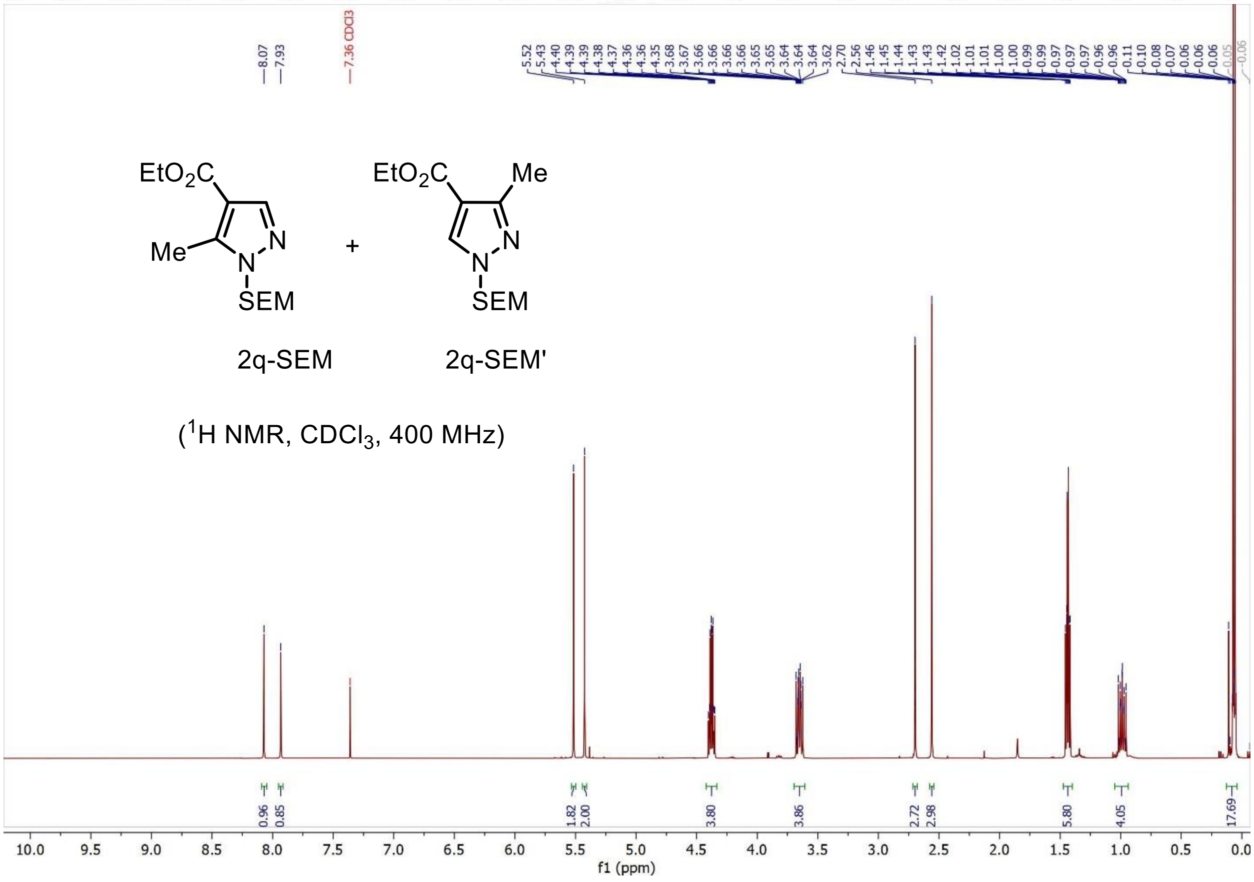

8.07
7.93
7.36 CDCl3
EtO$_2$C
Me
N
N
SEM
+
2q-SEM
2q-SEM'
(^{1}H NMR, CDCl$_3$, 400 MHz)
0.96
0.85
1.82
2.00
3.80
3.86
2.72
2.98
5.80
4.05
17.69
f1 (ppm)

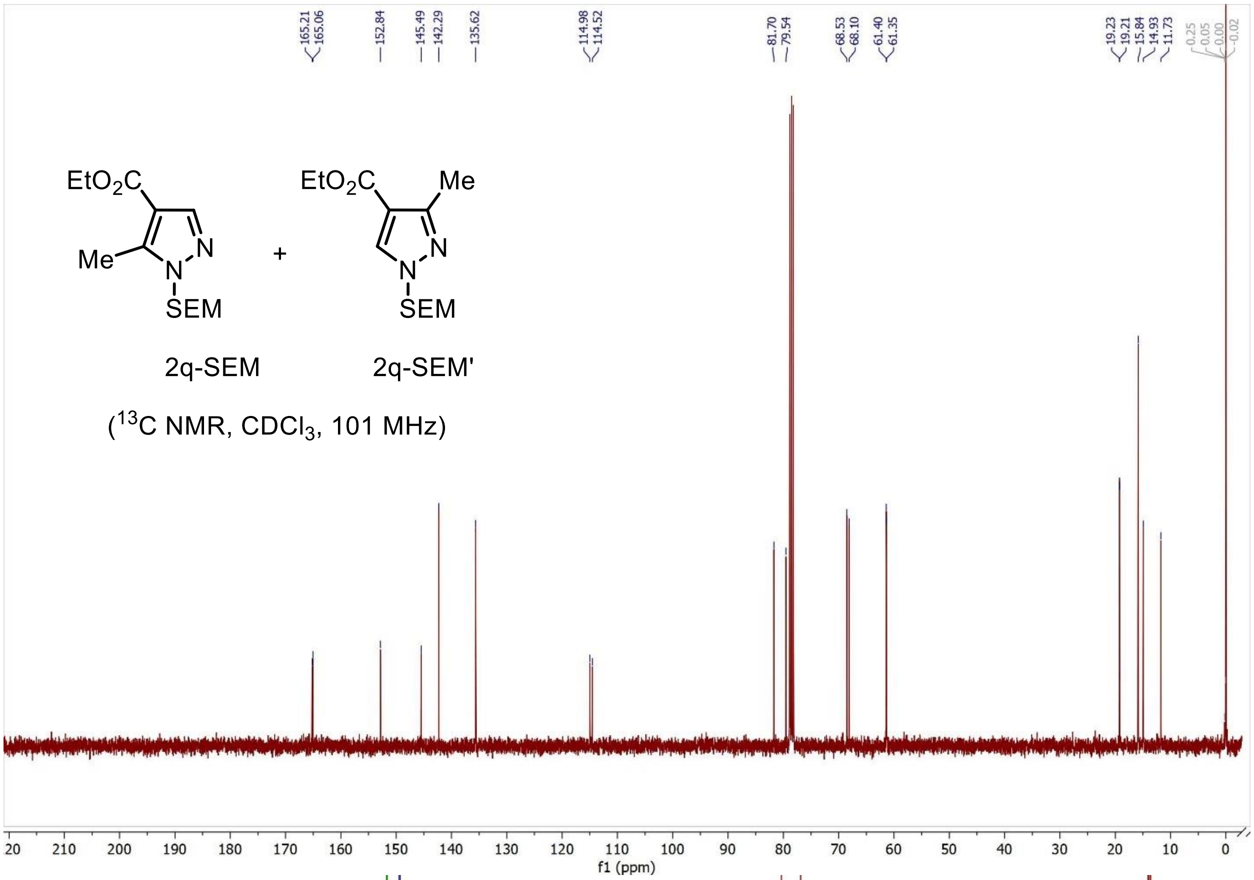

165.21
165.06
152.84
145.49
142.29
135.62
114.98
114.52
81.70
79.54
68.53
68.10
61.40
61.35
19.23
19.21
15.84
14.93
11.73
EtO2C
Me
N
N
SEM
+
2q-SEM
2q-SEM'
(13C NMR, CDCl3, 101 MHz)
f1 (ppm)

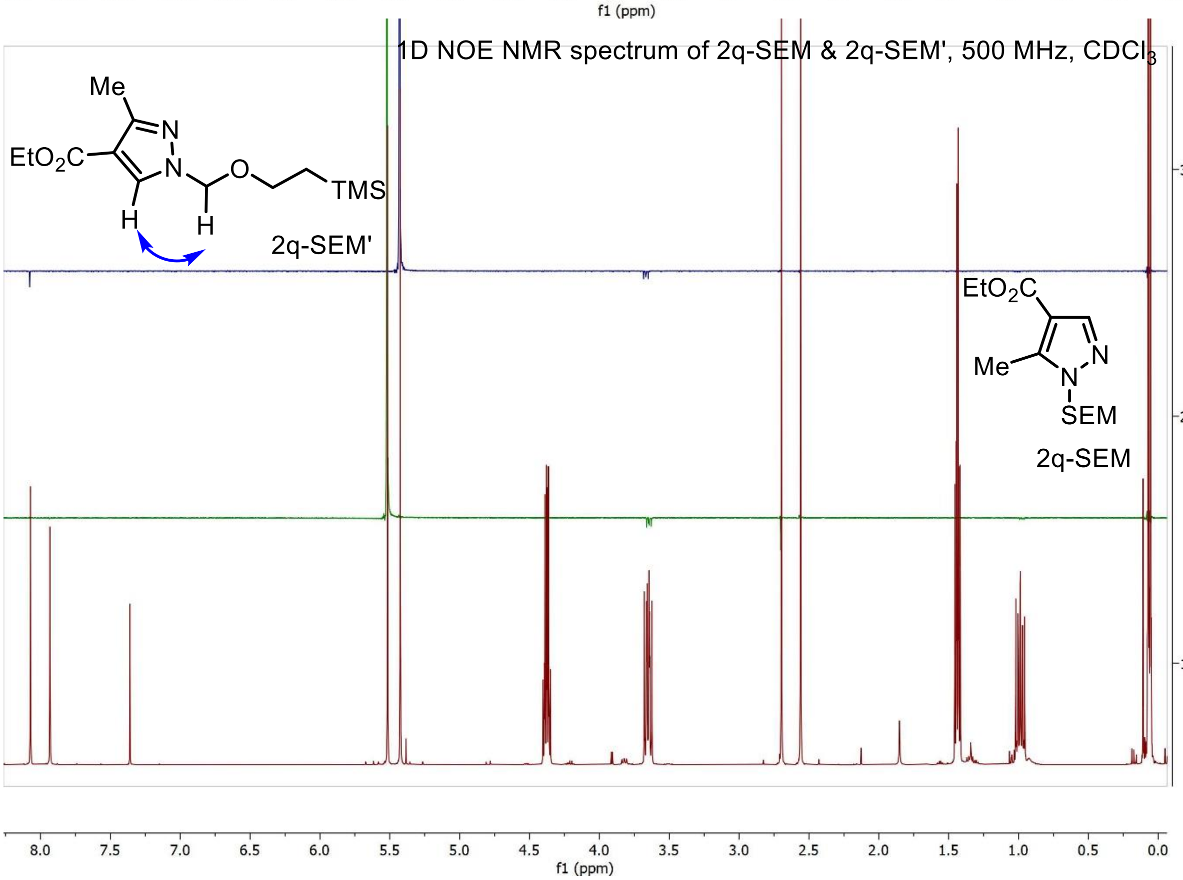

1D NOE NMR spectrum of 2q-SEM & 2q-SEM', 500 MHz, CDCl3
Me
N
EtO2C
O
TMS
H
2q-SEM'
Me
SEM
2q-SEM
f1 (ppm)

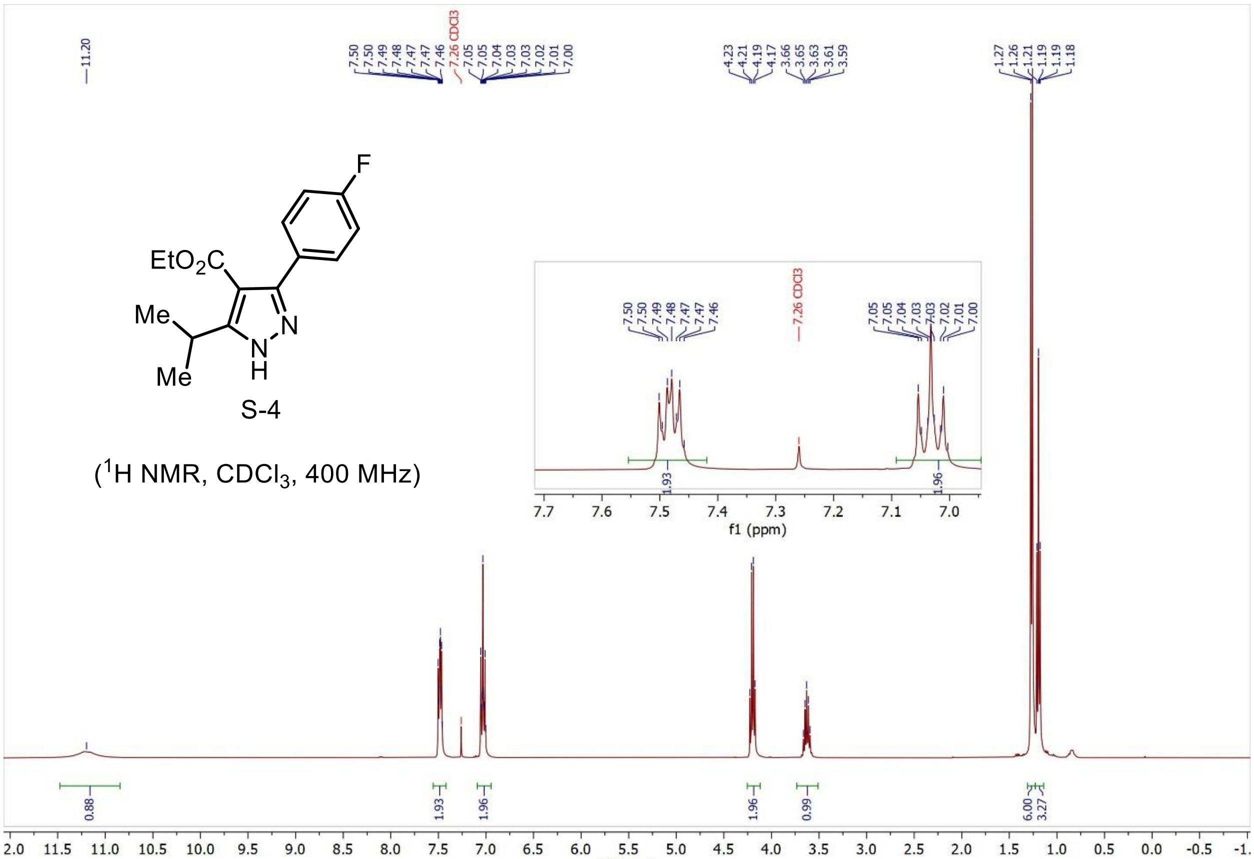
S-4
(1H NMR, CDCl3, 400 MHz)
11.20
7.50 7.50 7.49 7.48 7.47 7.47 7.46 7.26 CDCl3 7.05 7.05 7.04 7.03 7.03 7.02 7.01 7.00
4.23 4.21 4.19 4.17 3.66 3.65 3.63 3.61 3.59
1.27 1.26 1.21 1.19 1.19 1.18
0.88 1.93 1.96 1.96 0.99 6.00 3.27
f1 (ppm)

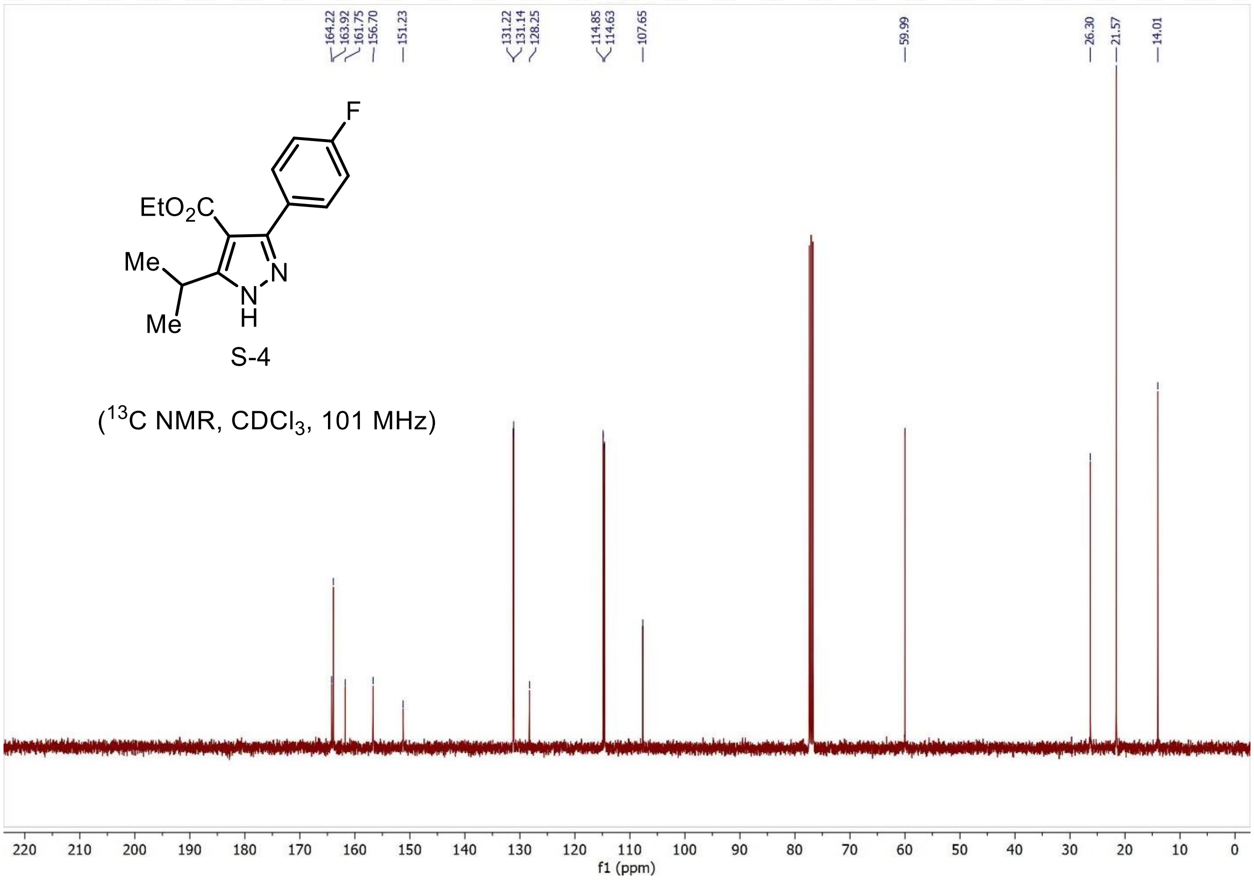
S-4
(13C NMR, CDCl3, 101 MHz)
164.22 163.92 161.75 156.70 151.23 131.22 131.14 128.25 114.85 114.63 107.65 59.99 26.30 21.57 14.01
f1 (ppm)

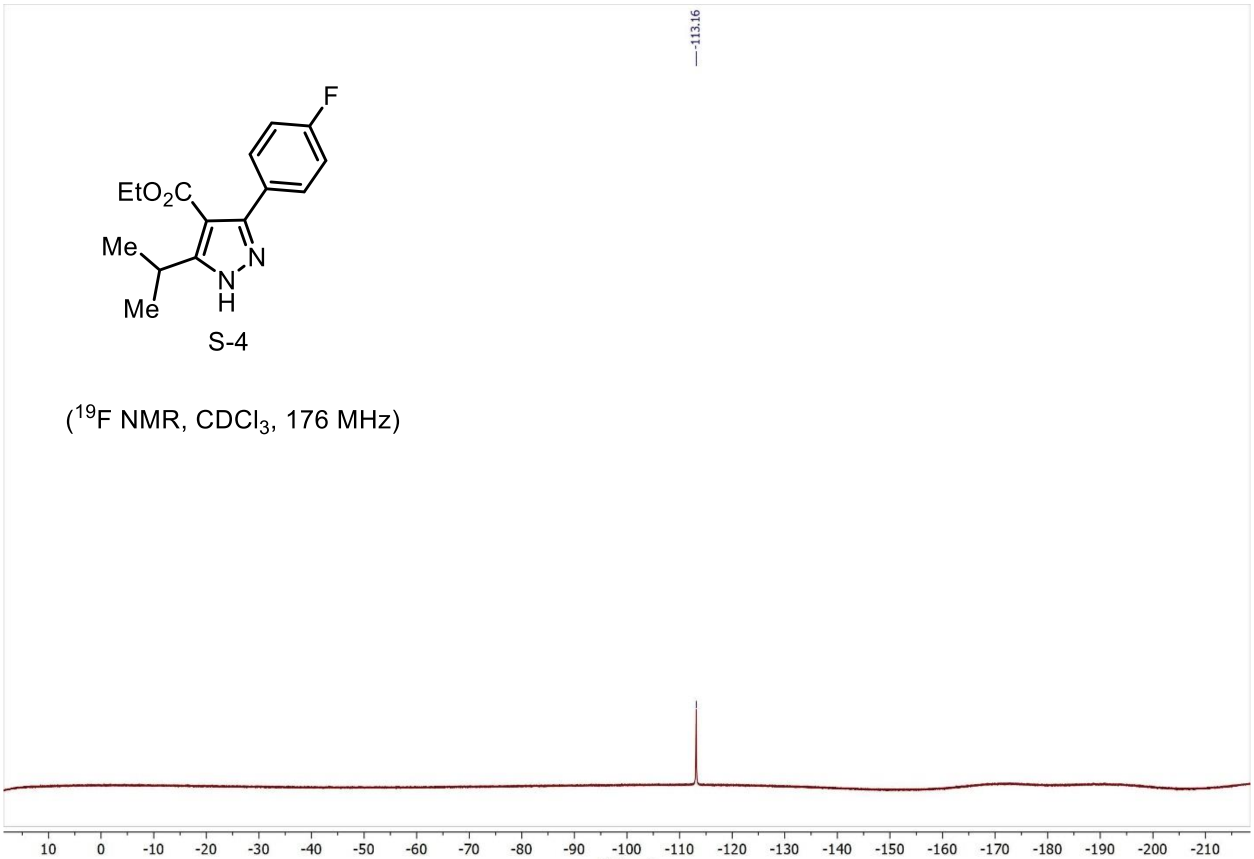
F
EtO2C
Me
N
N
H
Me
S-4
(19F NMR, CDCl3, 176 MHz)
-113.16

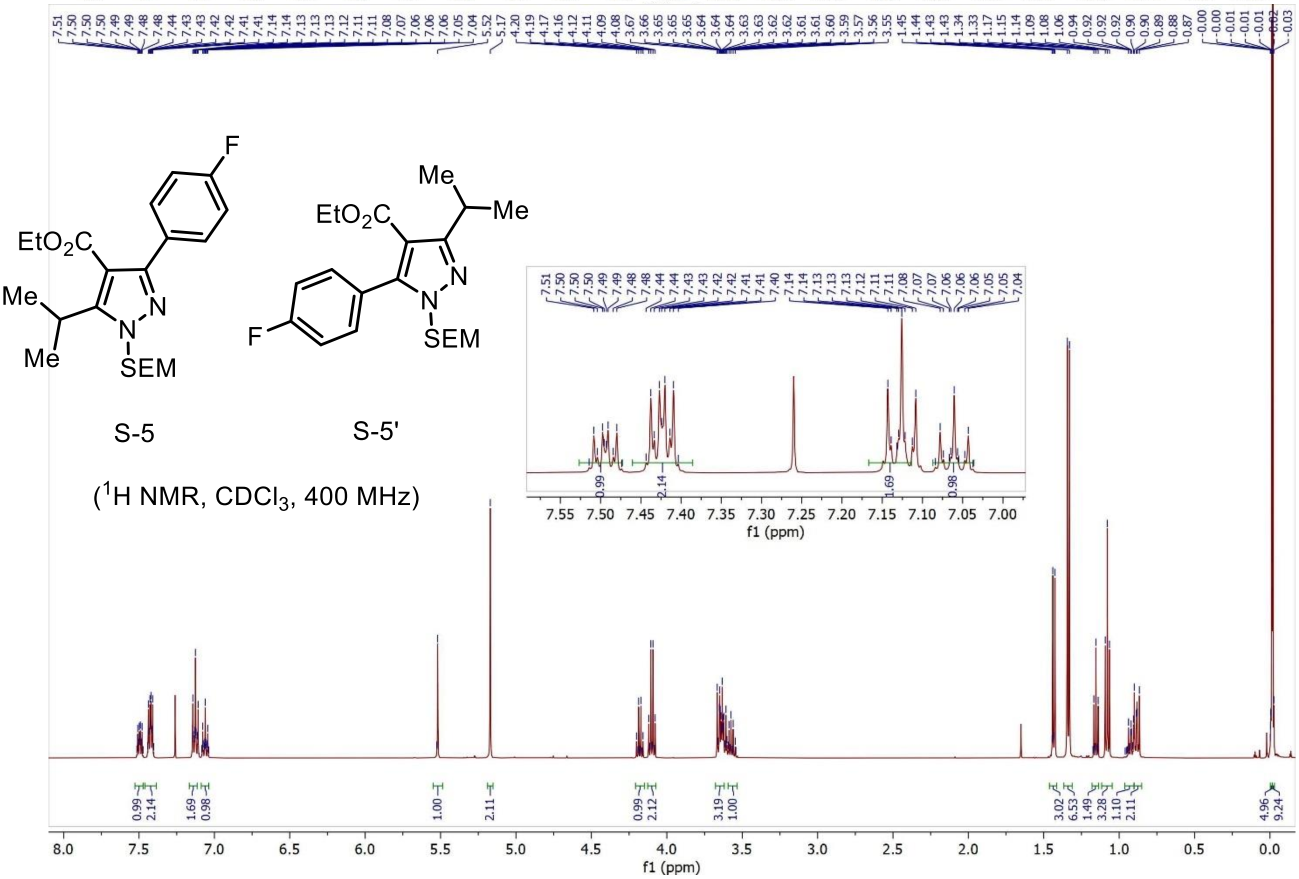
F
EtO2C
Me
N
N
Me
SEM
S-5
Me
Me
EtO2C
N
N
F
SEM
S-5'
(1H NMR, CDCl3, 400 MHz)
f1 (ppm)

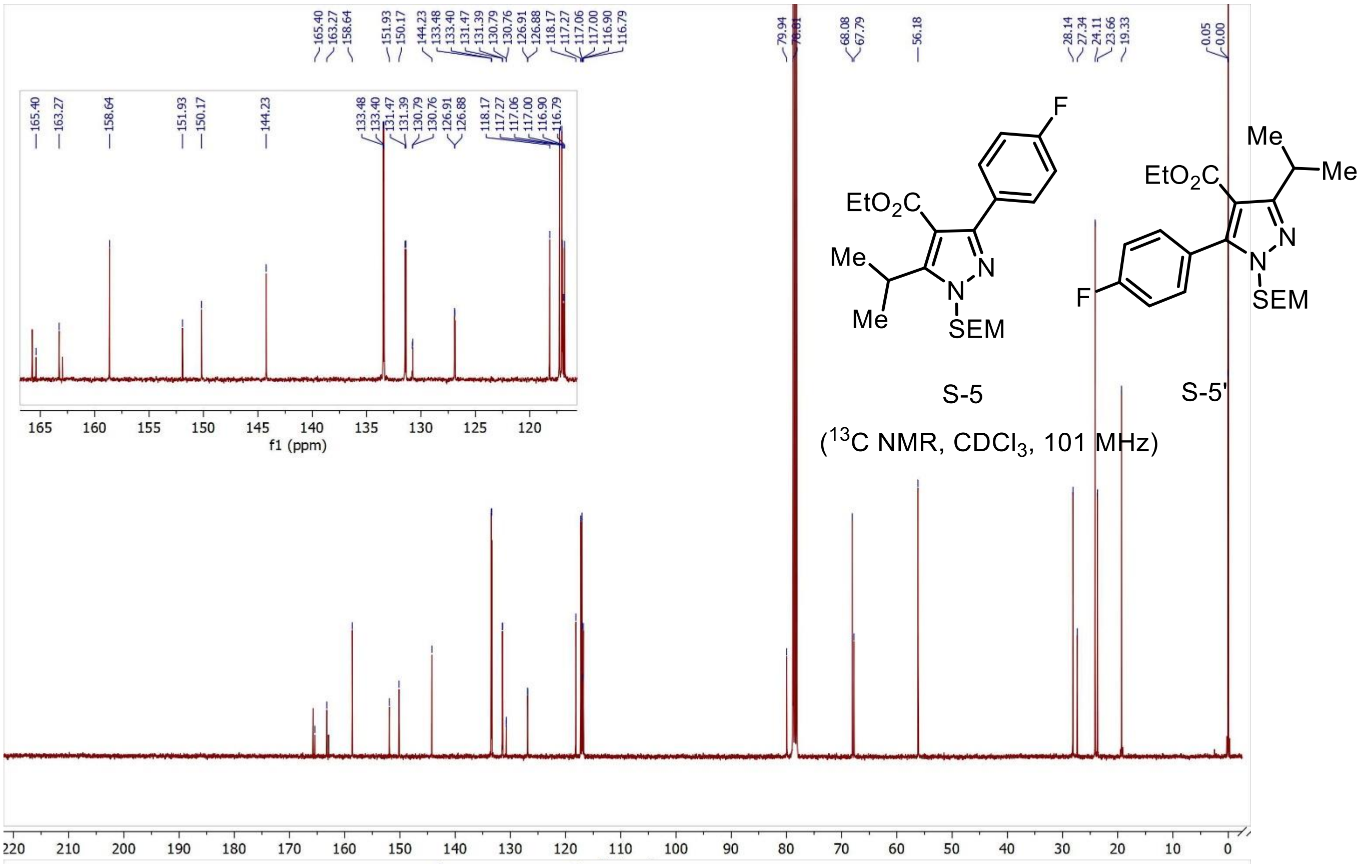

165.40
163.27
158.64
151.93
150.17
144.23
133.48
133.40
131.47
131.39
130.79
130.76
126.91
126.88
118.17
117.27
117.06
117.00
116.90
116.79
79.94
78.81
68.08
67.79
56.18
28.14
27.34
24.11
23.66
19.33
0.05
0.00
f1 (ppm)
EtO2C
Me
F
N
SEM
S-5
S-5'
(^{13}C NMR, $CDCl_3$, 101 MHz)

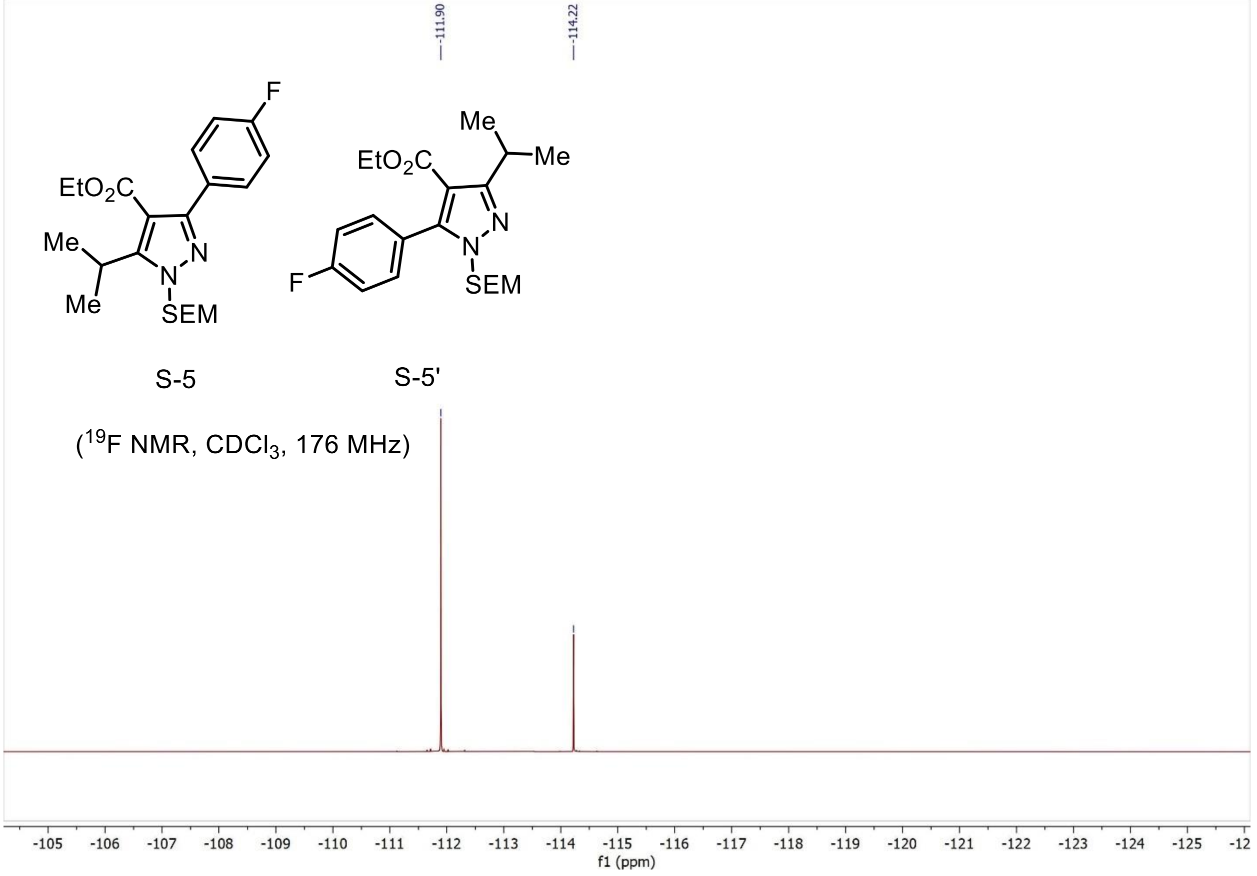

-111.90
-114.22
EtO2C
Me
F
N
SEM
S-5
S-5'
(^{19}F NMR, $CDCl_3$, 176 MHz)
f1 (ppm)

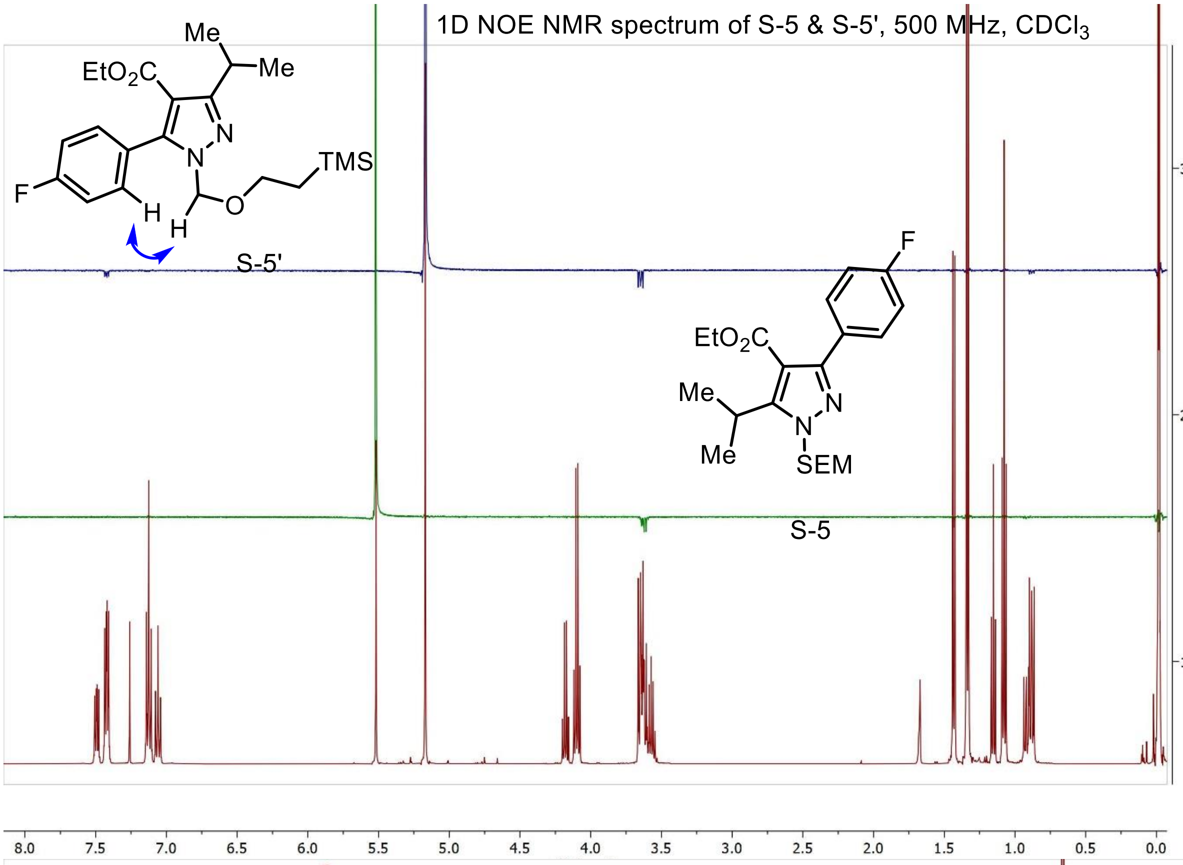
1D NOE NMR spectrum of S-5 & S-5', 500 MHz, CDCl3
EtO2C
Me
Me
N
N
TMS
O
F
H
H
S-5'
F
EtO2C
Me
Me
N
N
SEM
S-5

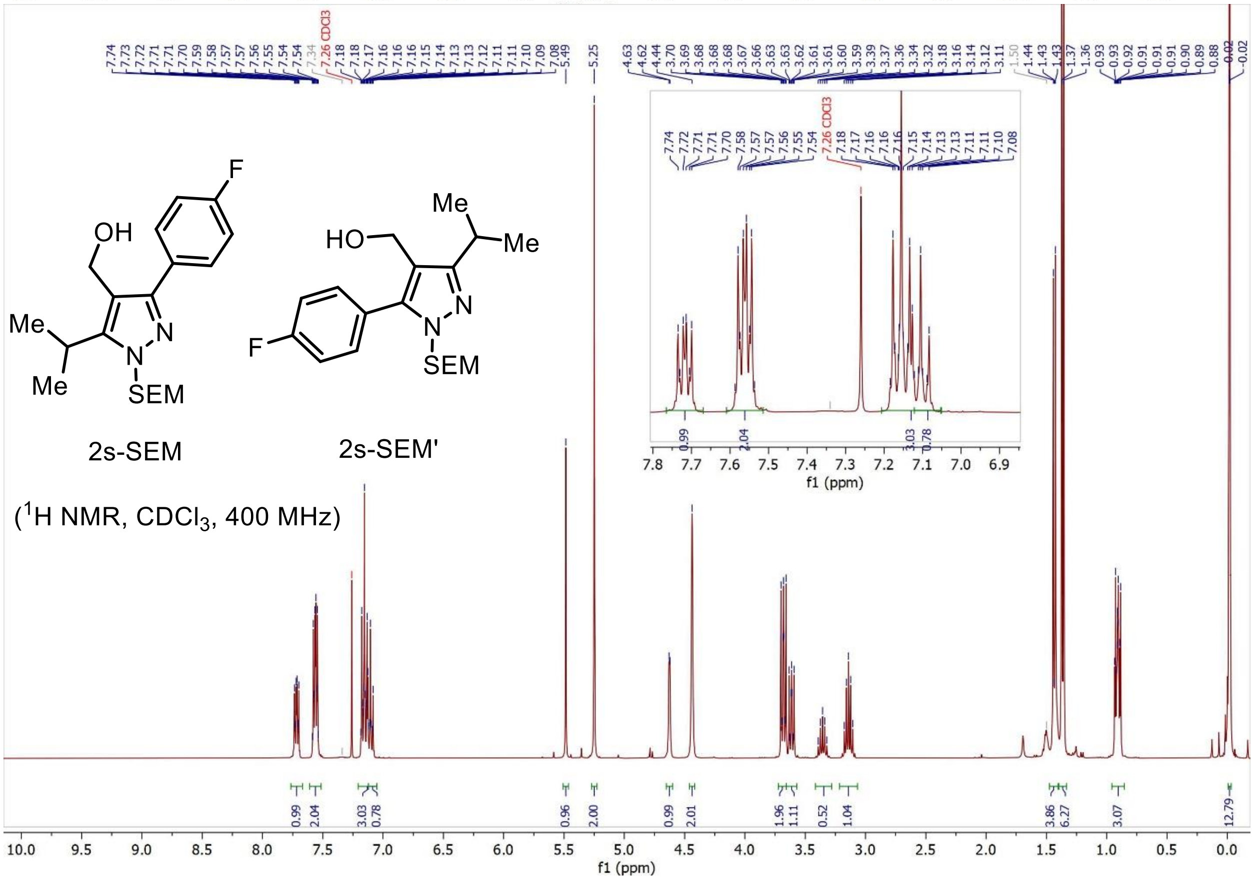
OH
F
Me
Me
N
N
SEM
2s-SEM
HO
Me
Me
F
N
N
SEM
2s-SEM'
(1H NMR, CDCl3, 400 MHz)
f1 (ppm)

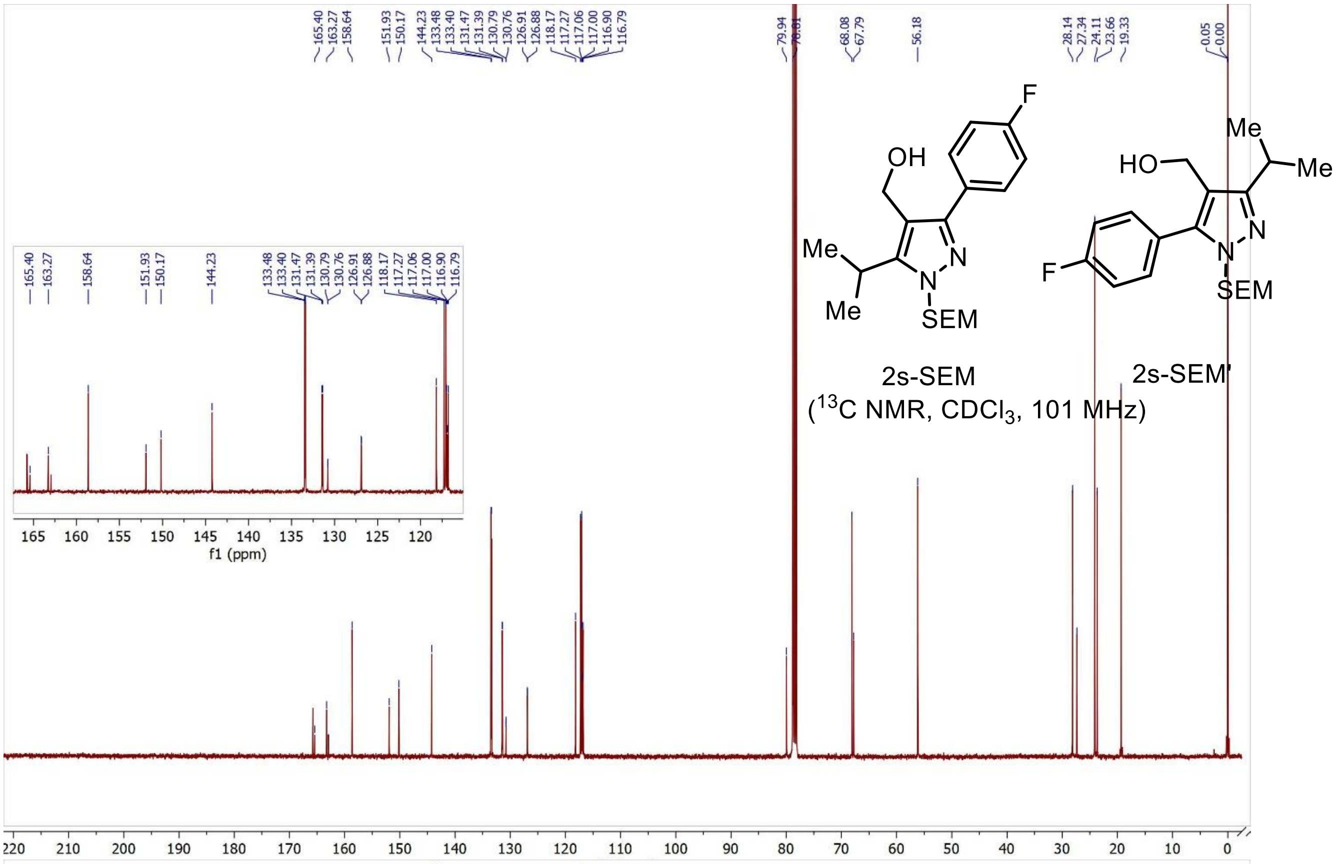

2s-SEM
2s-SEM'
(13C NMR, CDCl3, 101 MHz)

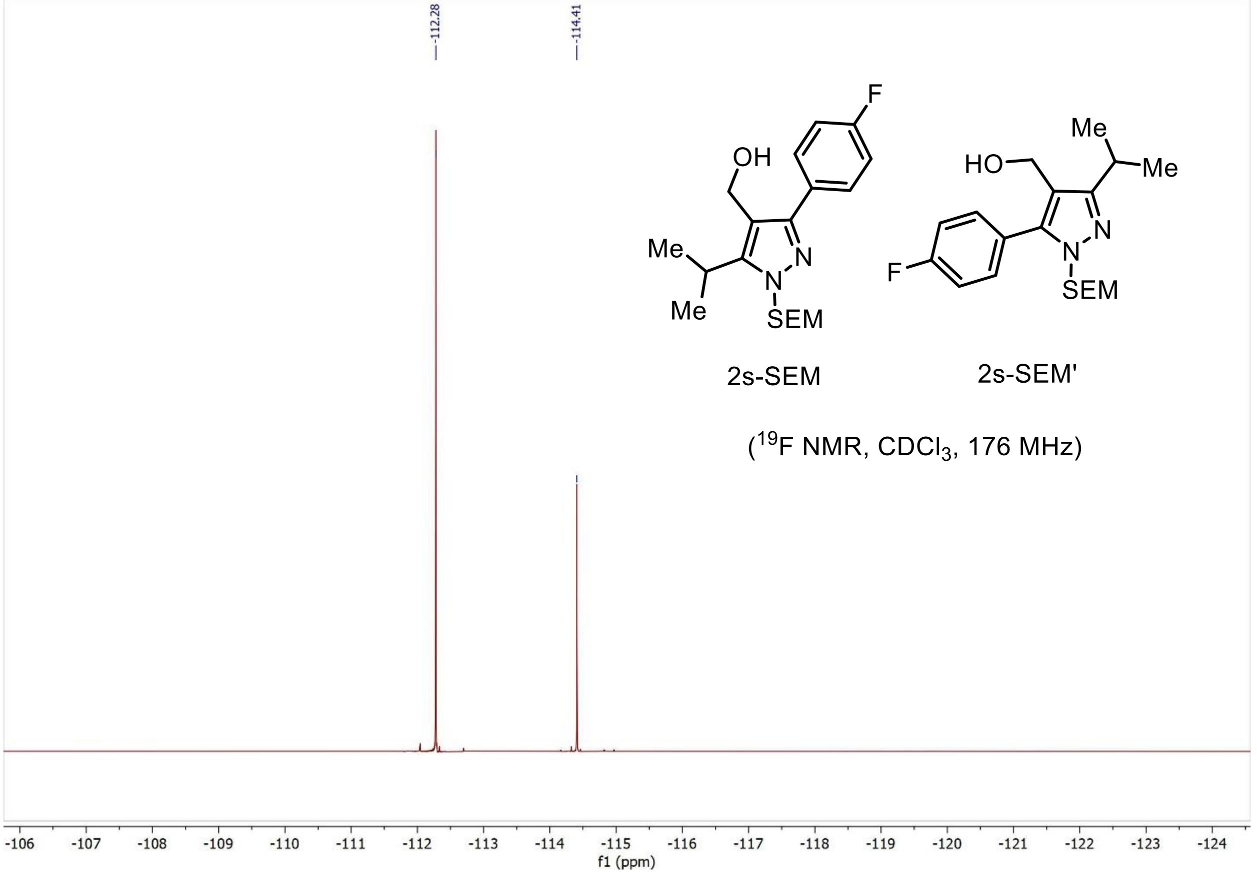

2s-SEM
2s-SEM'
(19F NMR, CDCl3, 176 MHz)
f1 (ppm)

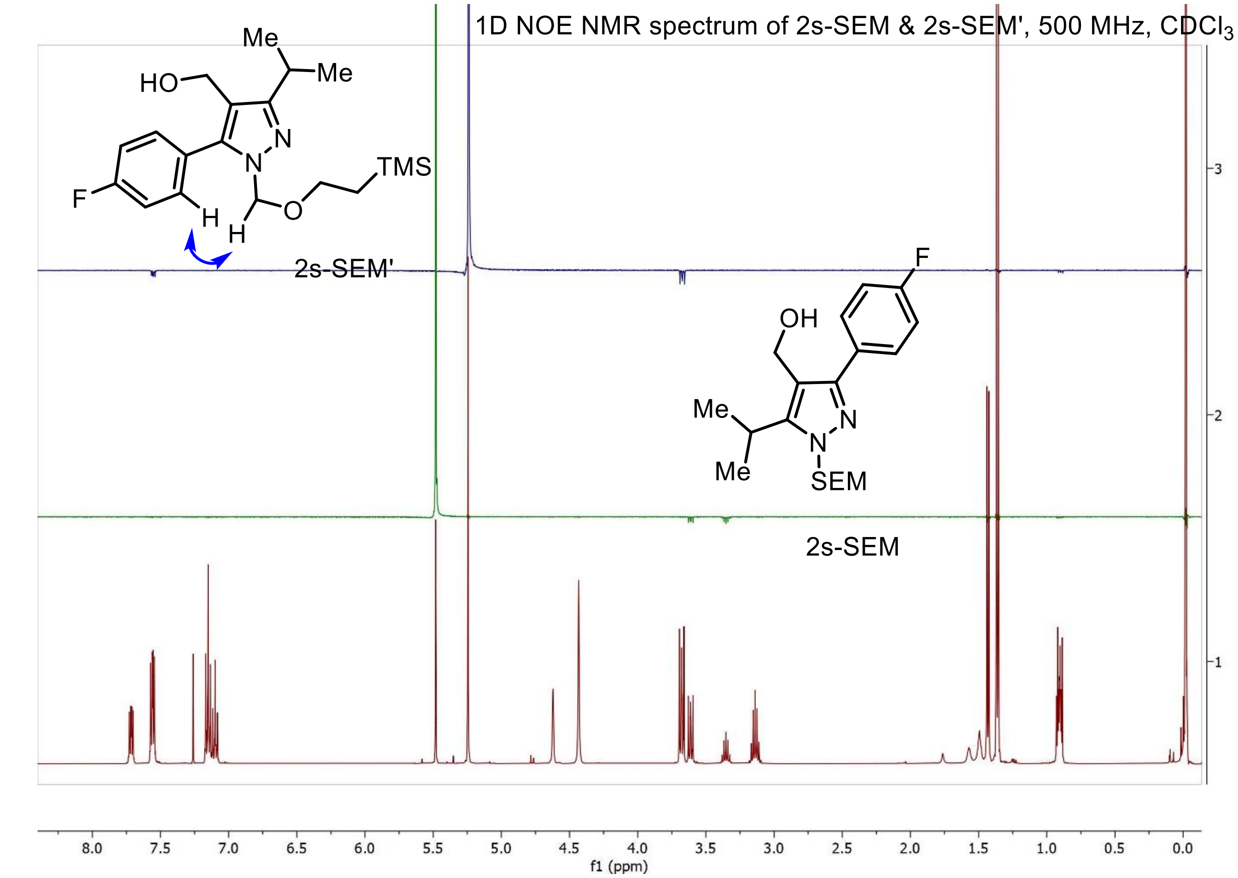
1D NOE NMR spectrum of 2s-SEM & 2s-SEM', 500 MHz, CDCl$_3$
2s-SEM'
2s-SEM
f1 (ppm)

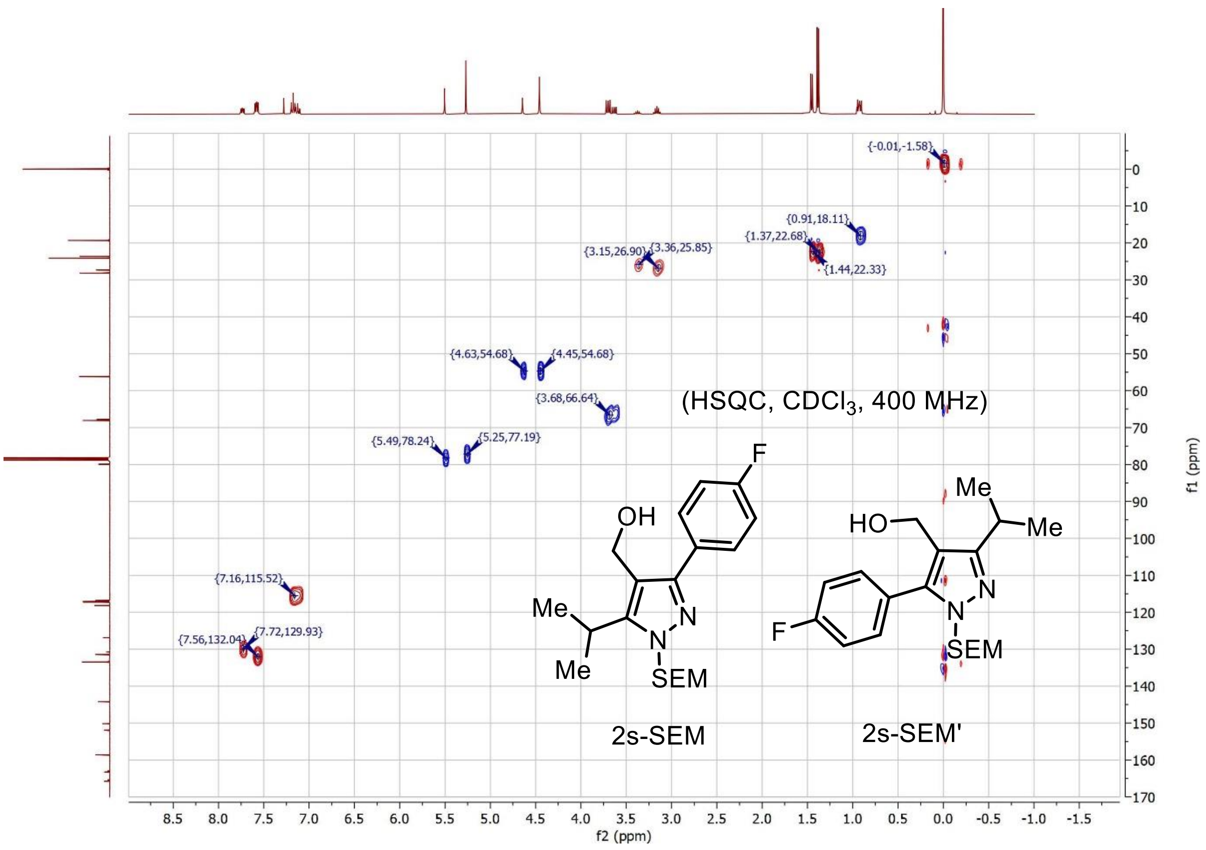
(HSQC, CDCl$_3$, 400 MHz)
{-0.01,-1.58}
{0.91,18.11}
{1.37,22.68}
{1.44,22.33}
{3.15,26.90}
{3.36,25.85}
{4.63,54.68}
{4.45,54.68}
{3.68,66.64}
{5.49,78.24}
{5.25,77.19}
{7.16,115.52}
{7.56,132.04}
{7.72,129.93}
2s-SEM
2s-SEM'
f2 (ppm)
f1 (ppm)

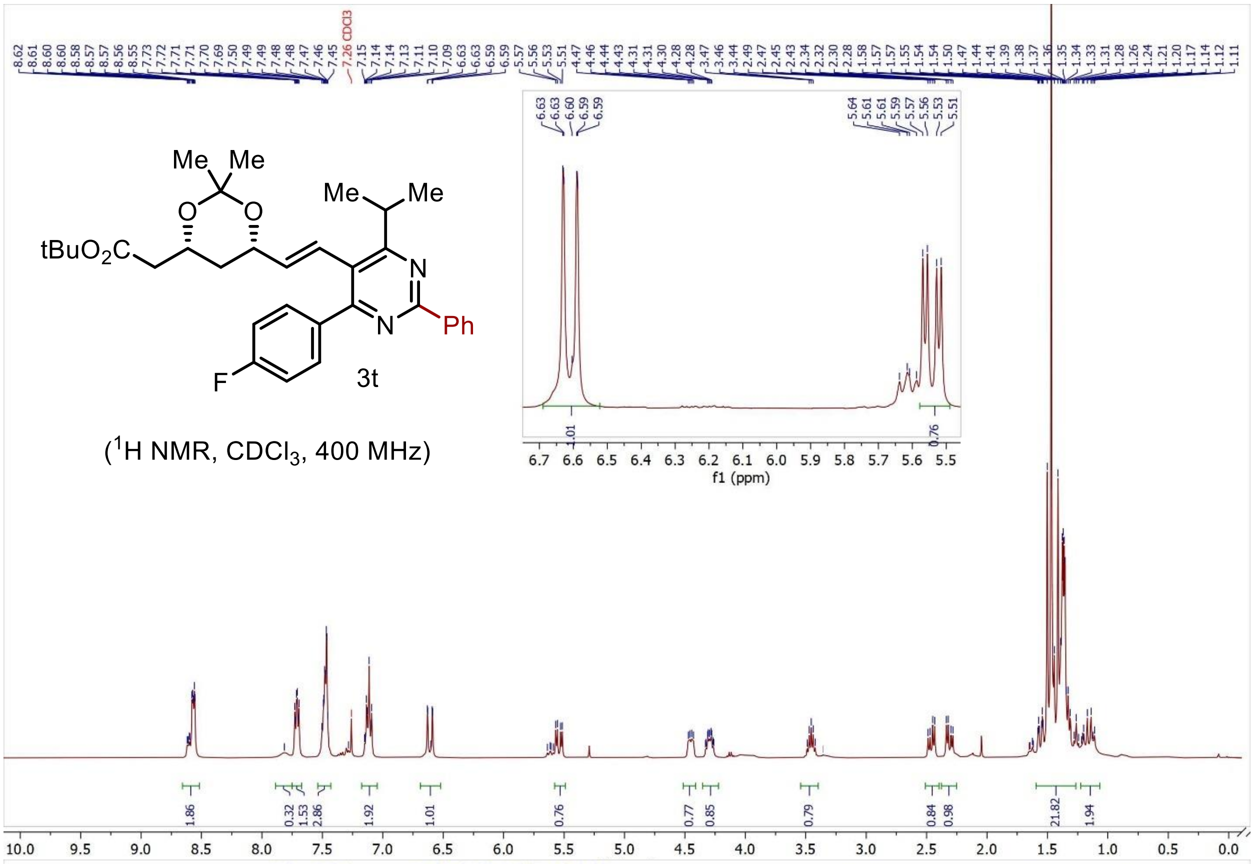
tBuO2C
Me Me
Me Me
N
N
Ph
F
3t
(1H NMR, CDCl3, 400 MHz)
f1 (ppm)

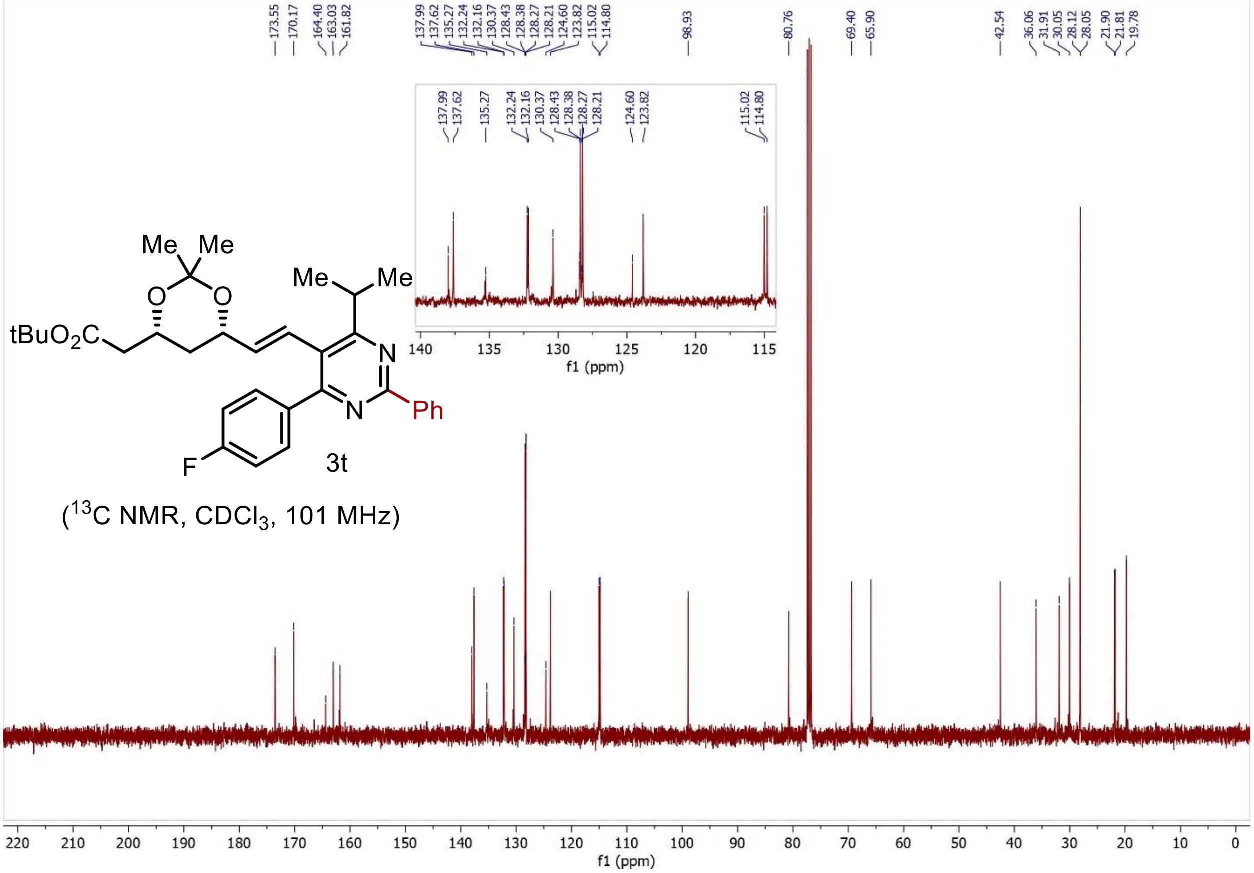
tBuO2C
Me Me
Me Me
N
N
Ph
F
3t
(13C NMR, CDCl3, 101 MHz)
f1 (ppm)

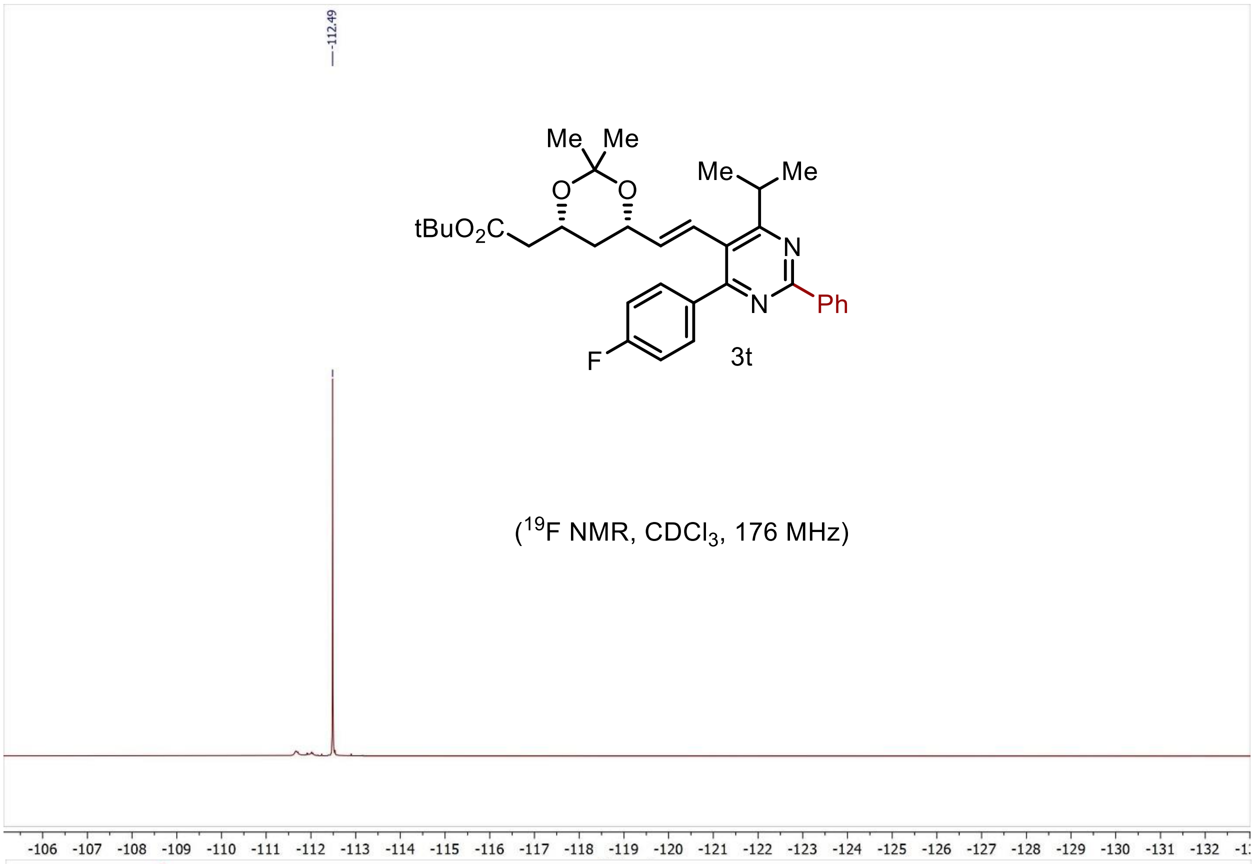

Me
Me
O
O
Me
Me
tBuO2C
N
N
Ph
F
3t
(19F NMR, CDCl3, 176 MHz)

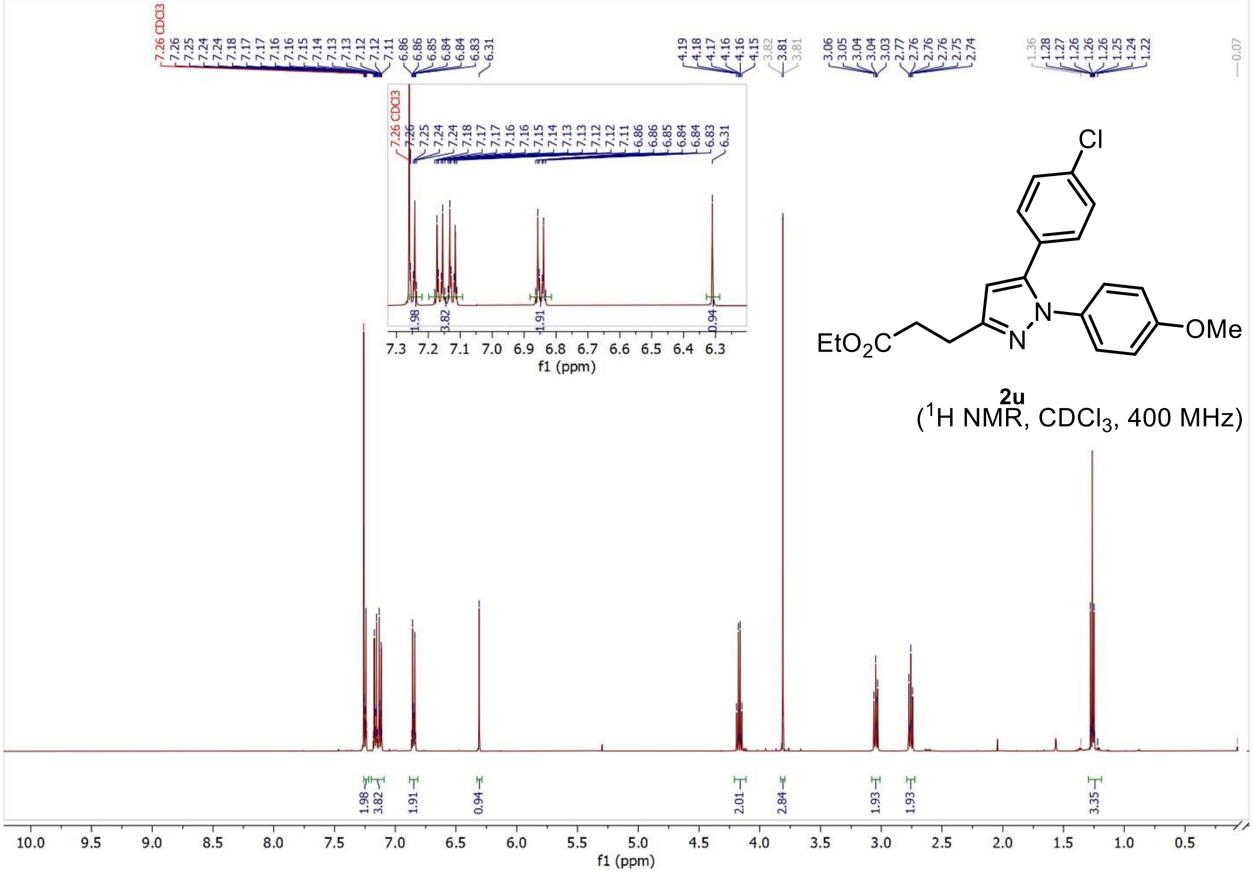

Cl
N
N
EtO2C
OMe
2u
(1H NMR, CDCl3, 400 MHz)
f1 (ppm)

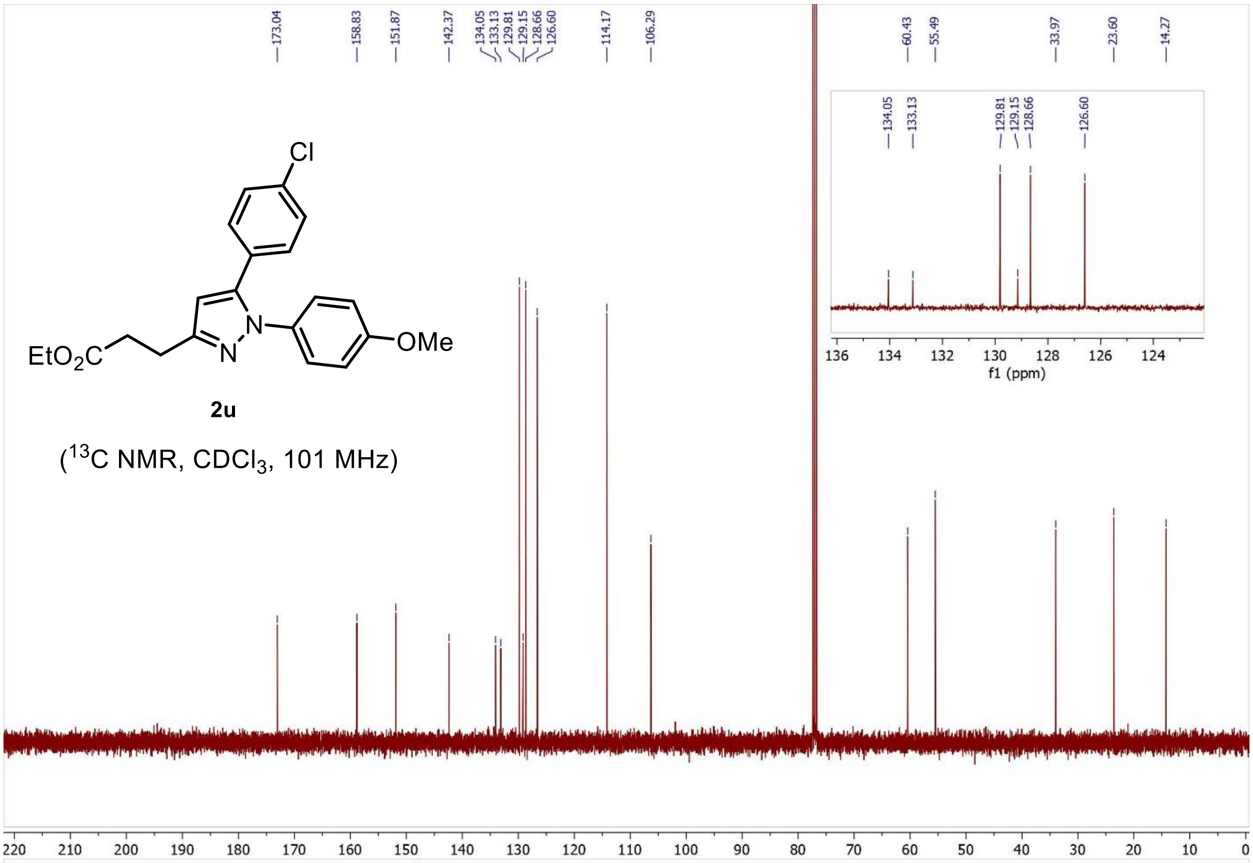

2u

(^{13}C NMR, $CDCl_3$, 101 MHz)

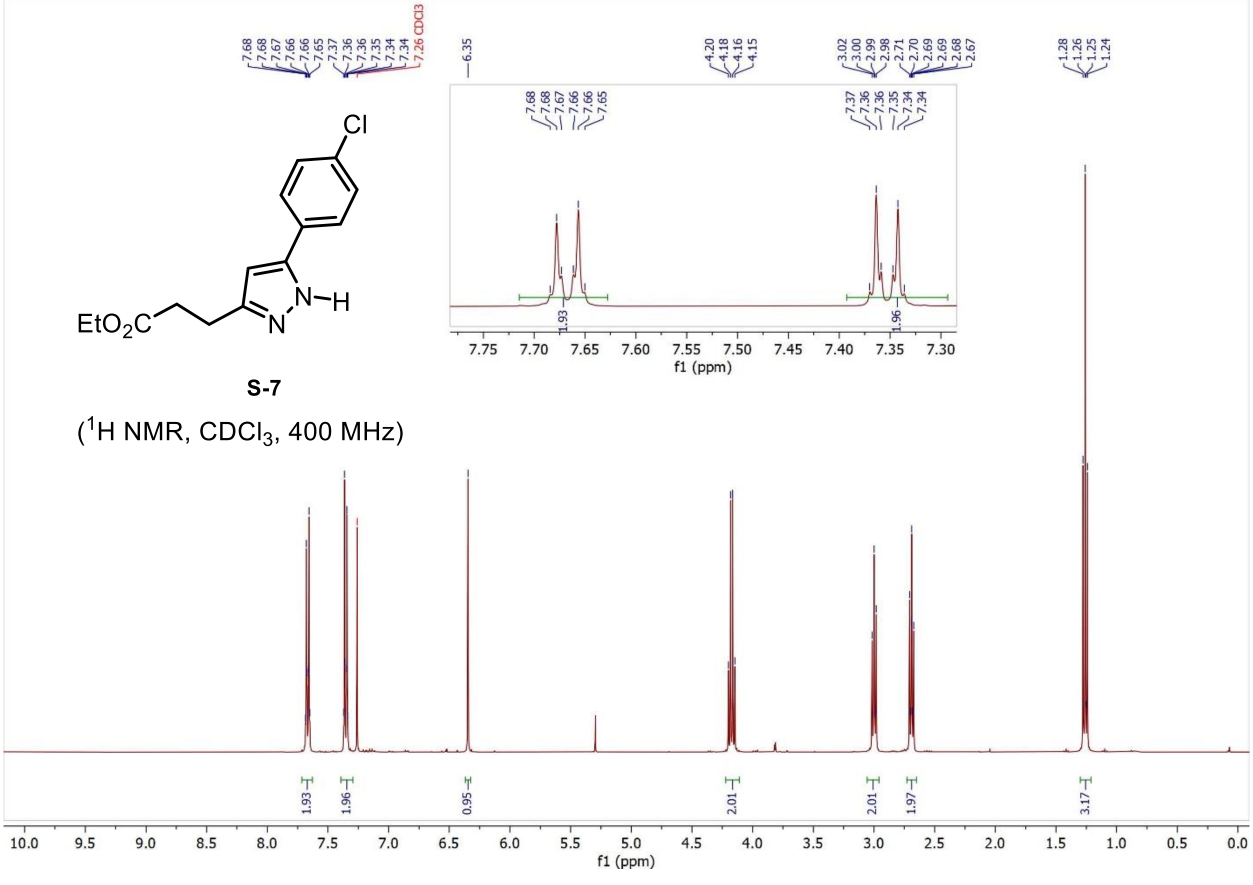

S-7

(^{1}H NMR, $CDCl_3$, 400 MHz)

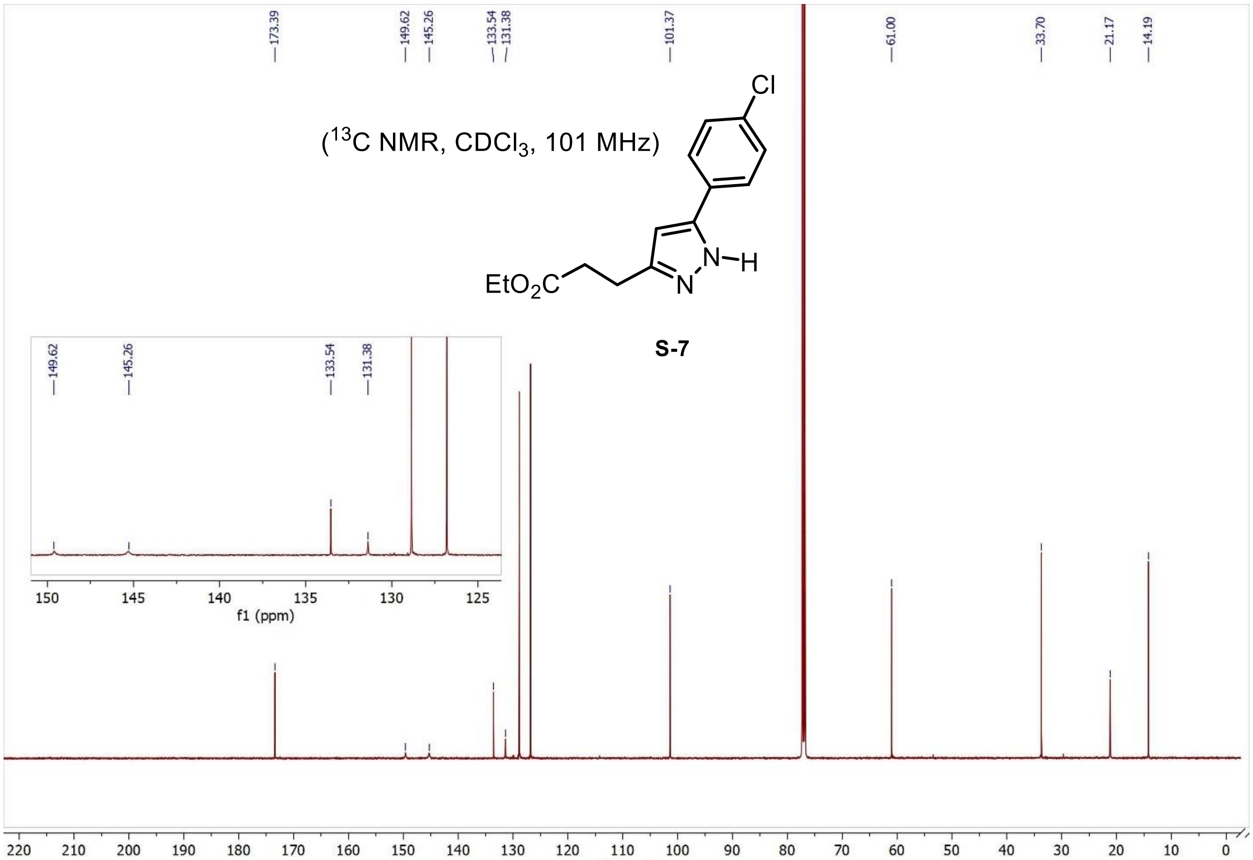
(13C NMR, CDCl3, 101 MHz)
S-7
EtO2C
N–H
N
Cl
f1 (ppm)

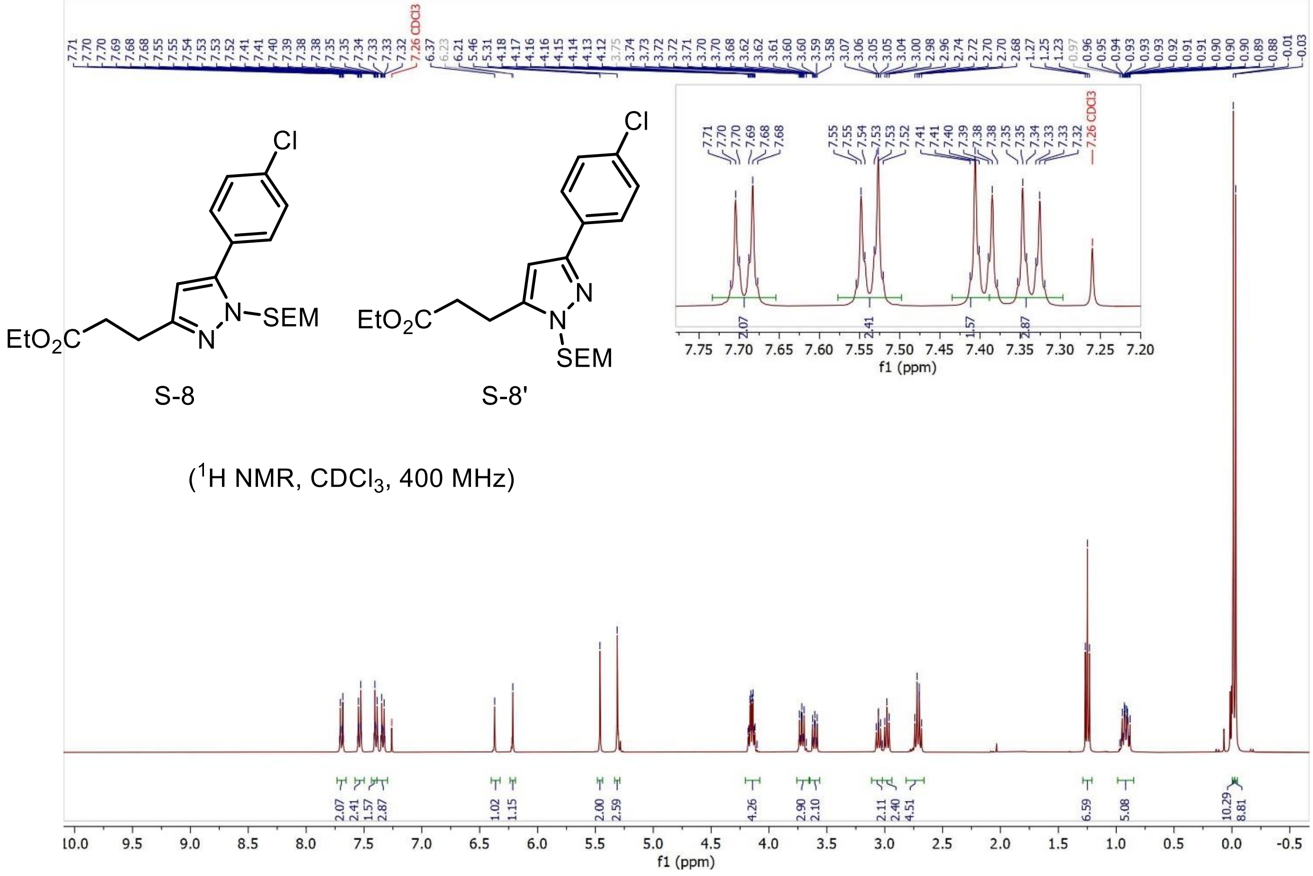
S-8
S-8'
EtO2C
N–SEM
SEM
Cl
(1H NMR, CDCl3, 400 MHz)
f1 (ppm)

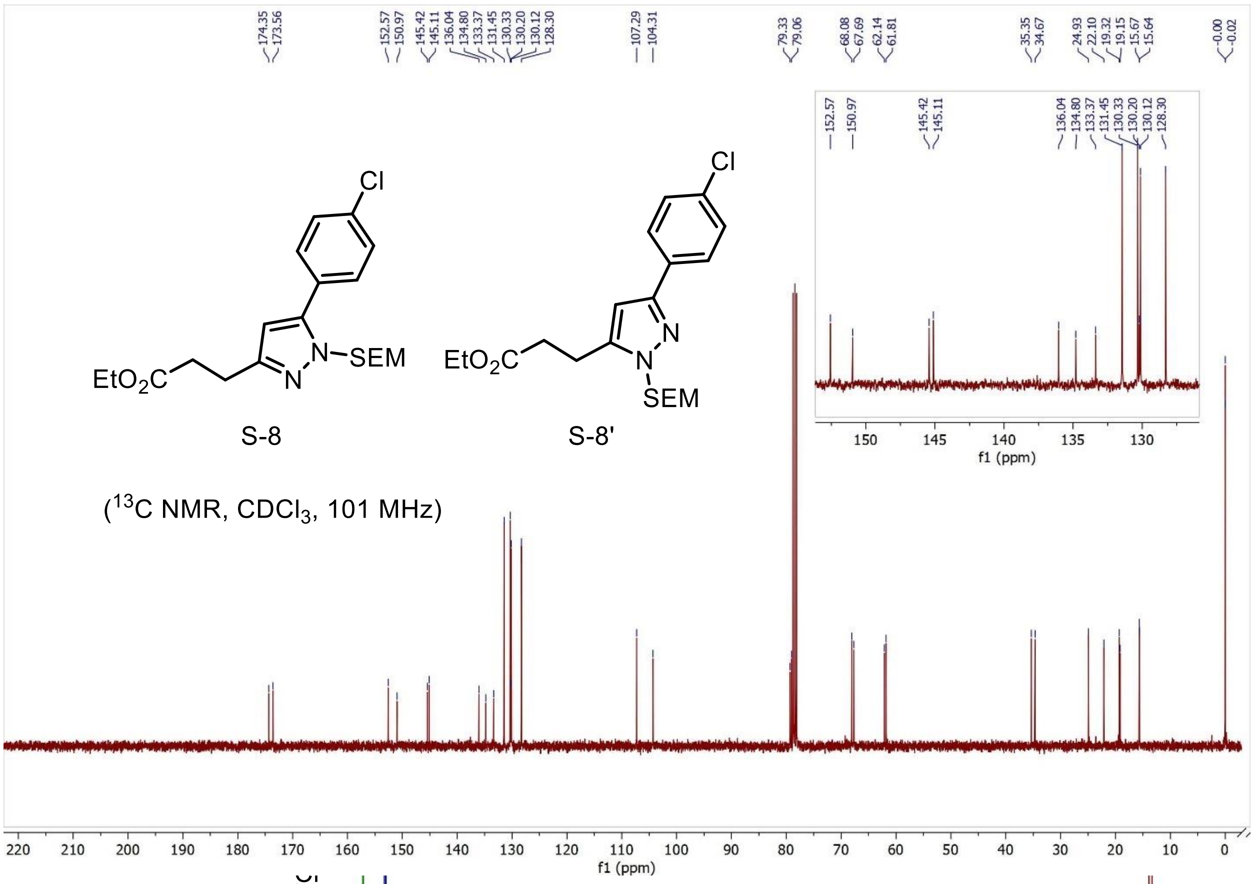

174.35
173.56
152.57
150.97
145.42
145.11
136.04
134.80
133.37
131.45
130.33
130.20
130.12
128.30
107.29
104.31
79.33
79.06
68.08
67.69
62.14
61.81
35.35
34.67
24.93
22.10
19.32
19.15
15.67
15.64
-0.00
-0.02
Cl
EtO2C
N
N
SEM
S-8
S-8'
(13C NMR, CDCl3, 101 MHz)
f1 (ppm)

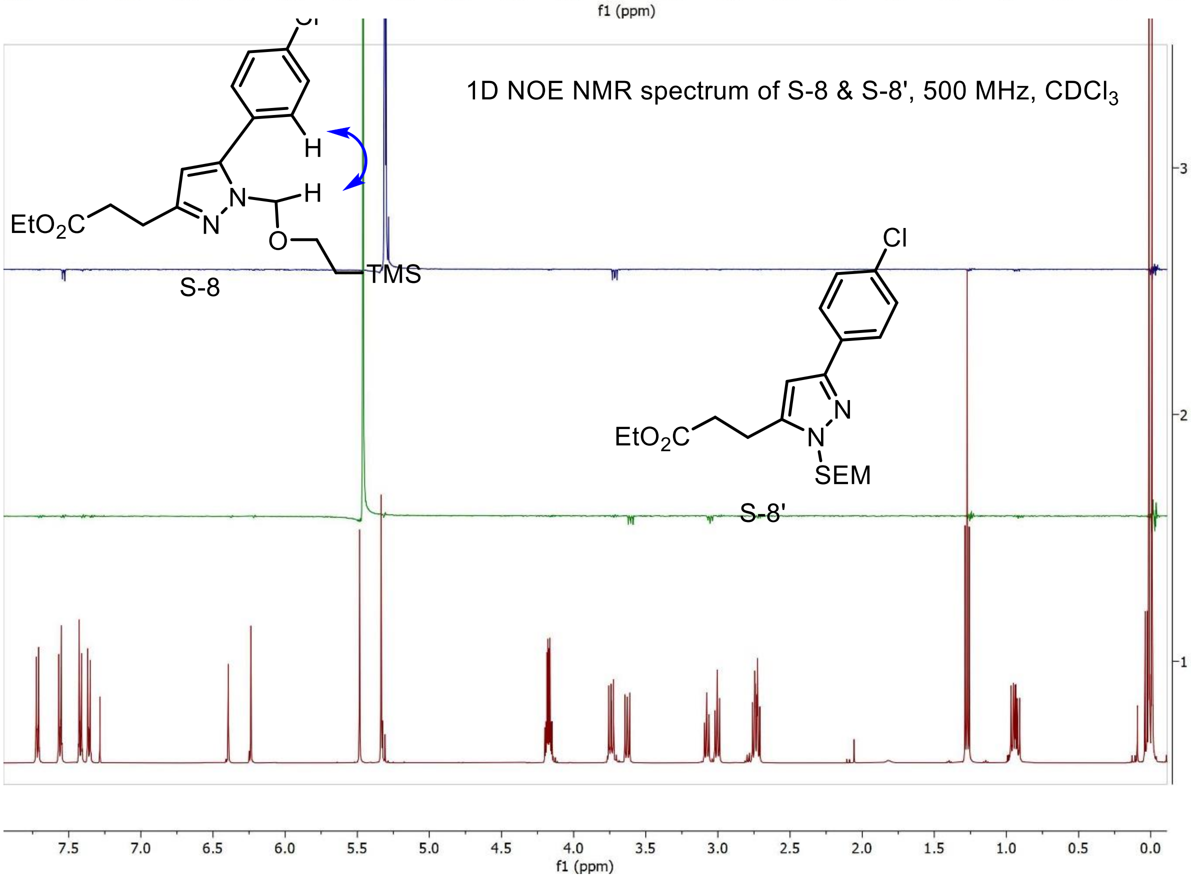

1D NOE NMR spectrum of S-8 & S-8', 500 MHz, CDCl3
EtO2C
N
H
O
TMS
S-8
Cl
SEM
S-8'
f1 (ppm)

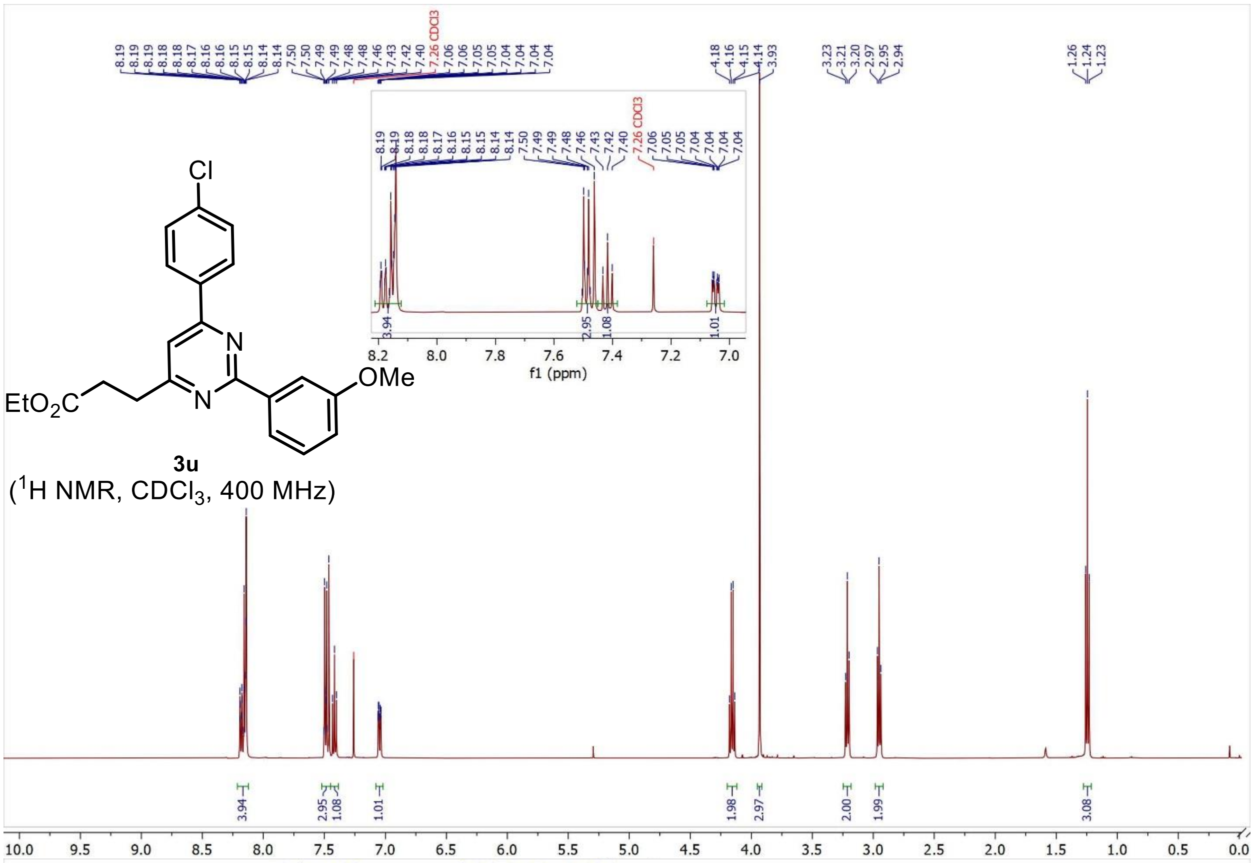

3u
(^{1}H NMR, $CDCl_3$, 400 MHz)

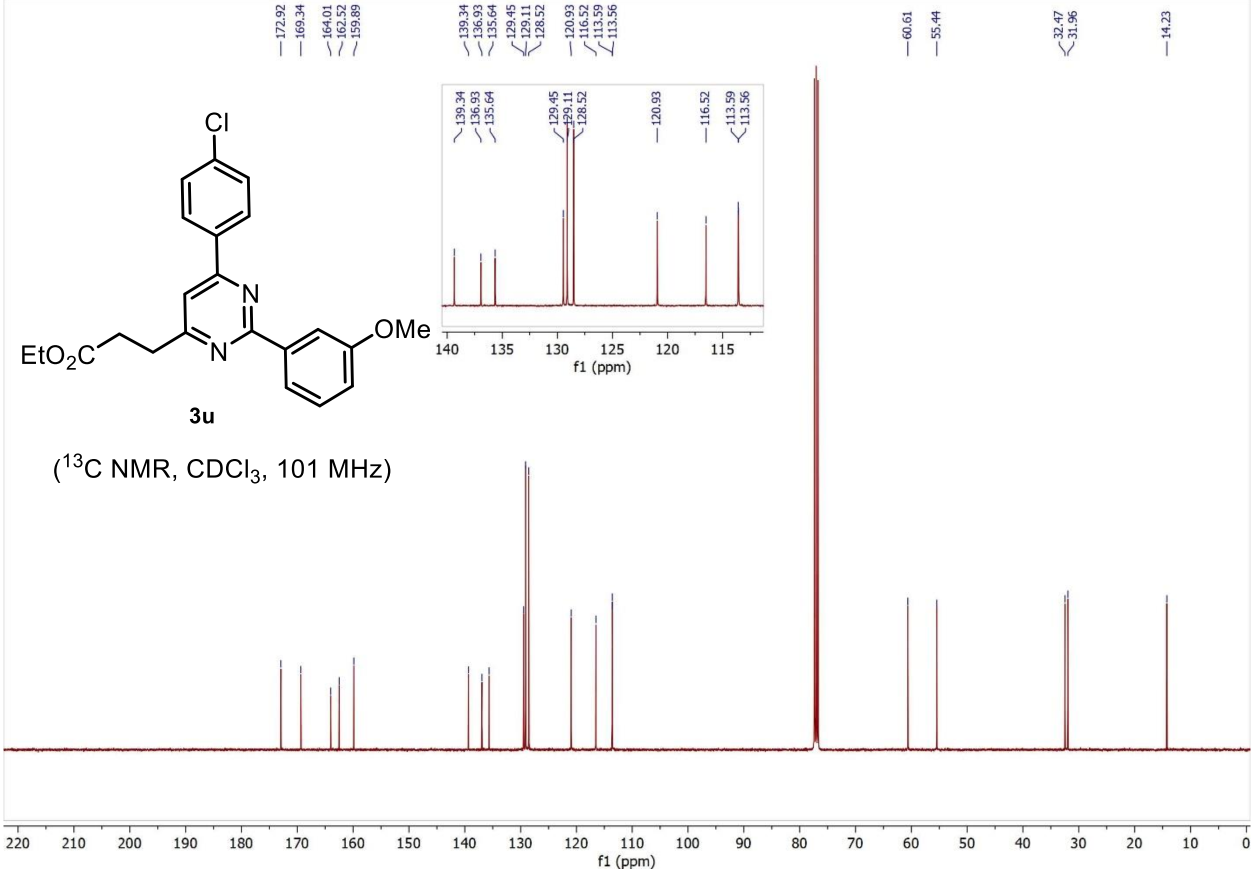

3u

(^{13}C NMR, $CDCl_3$, 101 MHz)

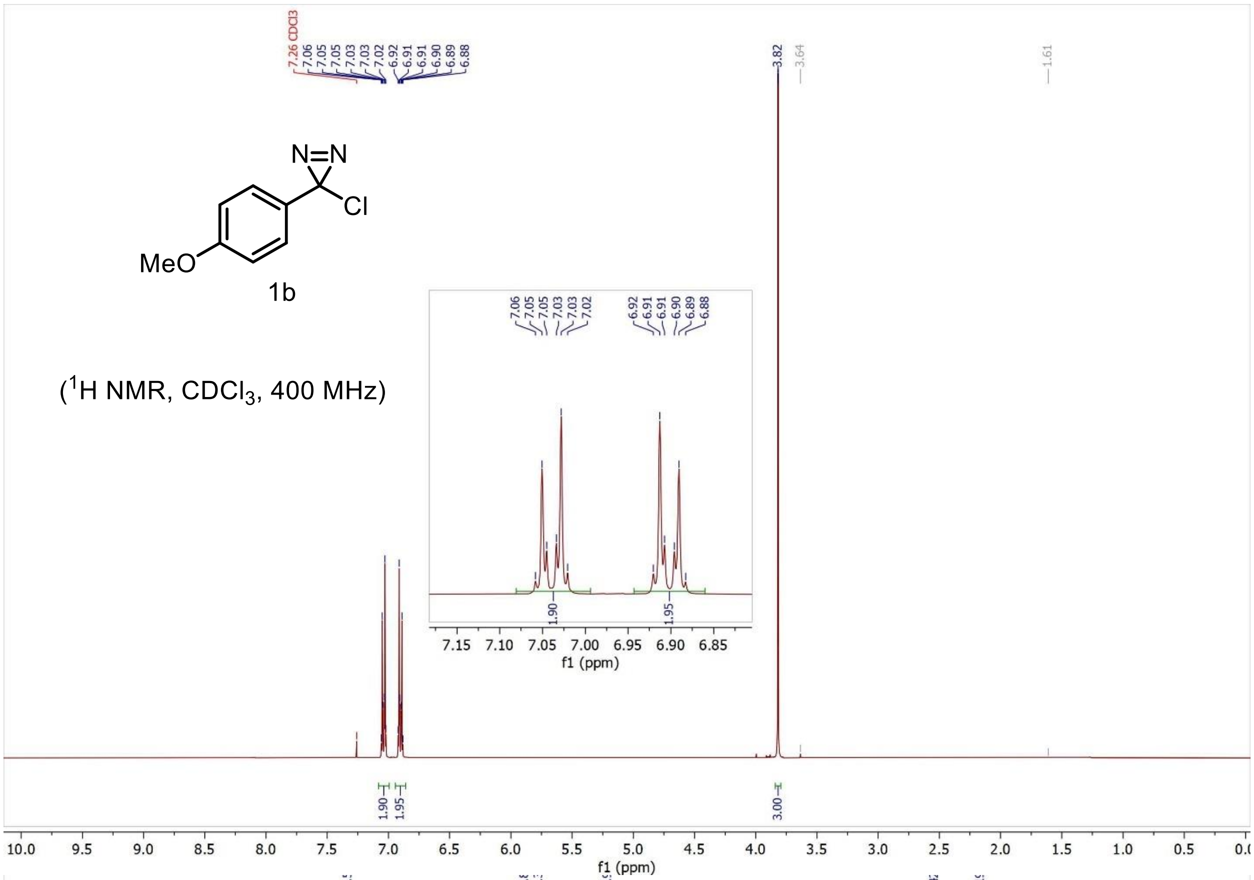

7.26 CDCl3
7.06
7.05
7.05
7.03
7.03
7.02
6.92
6.91
6.91
6.90
6.89
6.88
3.82
3.64
1.61
N=N
Cl
MeO
1b
(1H NMR, CDCl3, 400 MHz)
1.90
1.95
3.00
f1 (ppm)

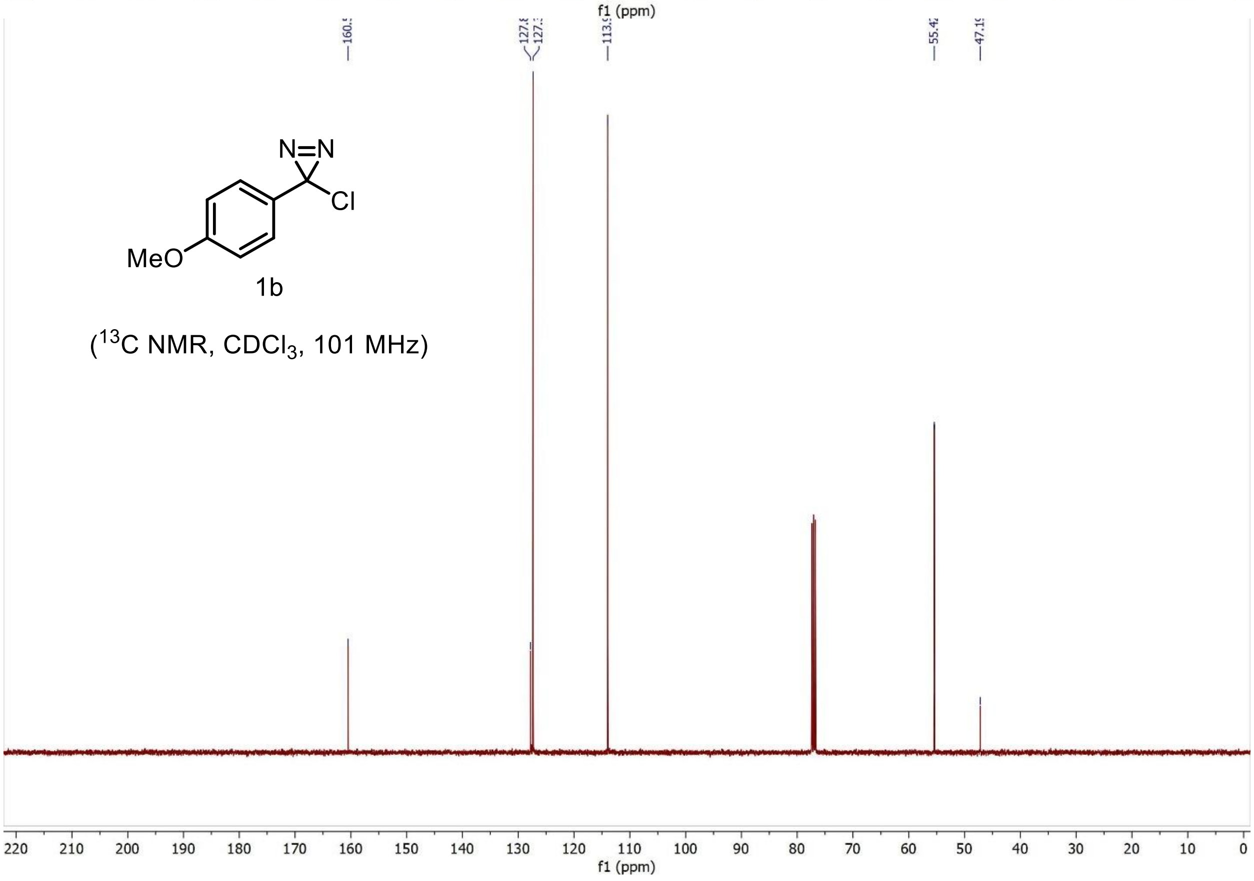

160.5
55.4
47.1
N=N
Cl
MeO
1b
(13C NMR, CDCl3, 101 MHz)
f1 (ppm)

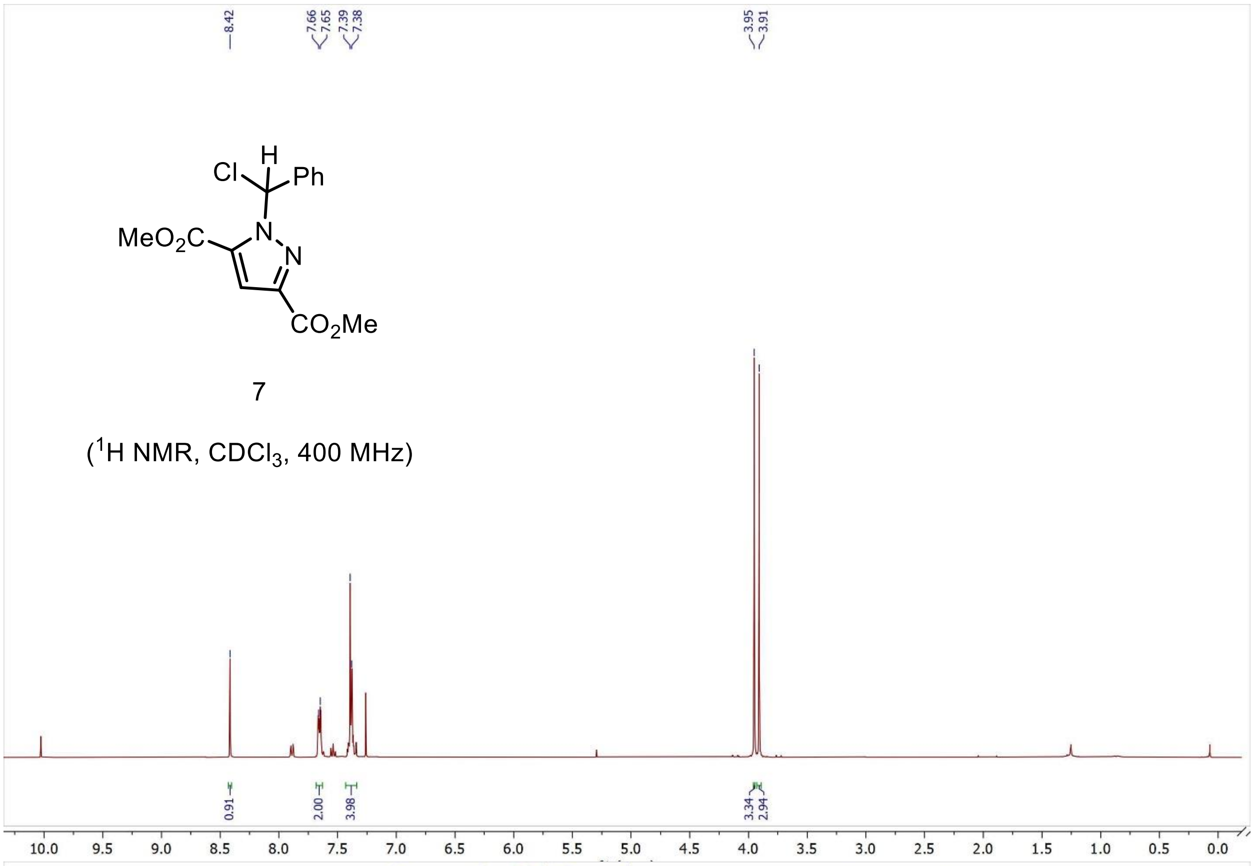
8.42
7.66
7.65
7.39
7.38
3.95
3.91
Cl
H
Ph
MeO2C
N
N
CO2Me
7
(1H NMR, CDCl3, 400 MHz)
0.91
2.00
3.98
3.34
2.94
10.0 9.5 9.0 8.5 8.0 7.5 7.0 6.5 6.0 5.5 5.0 4.5 4.0 3.5 3.0 2.5 2.0 1.5 1.0 0.5 0.0

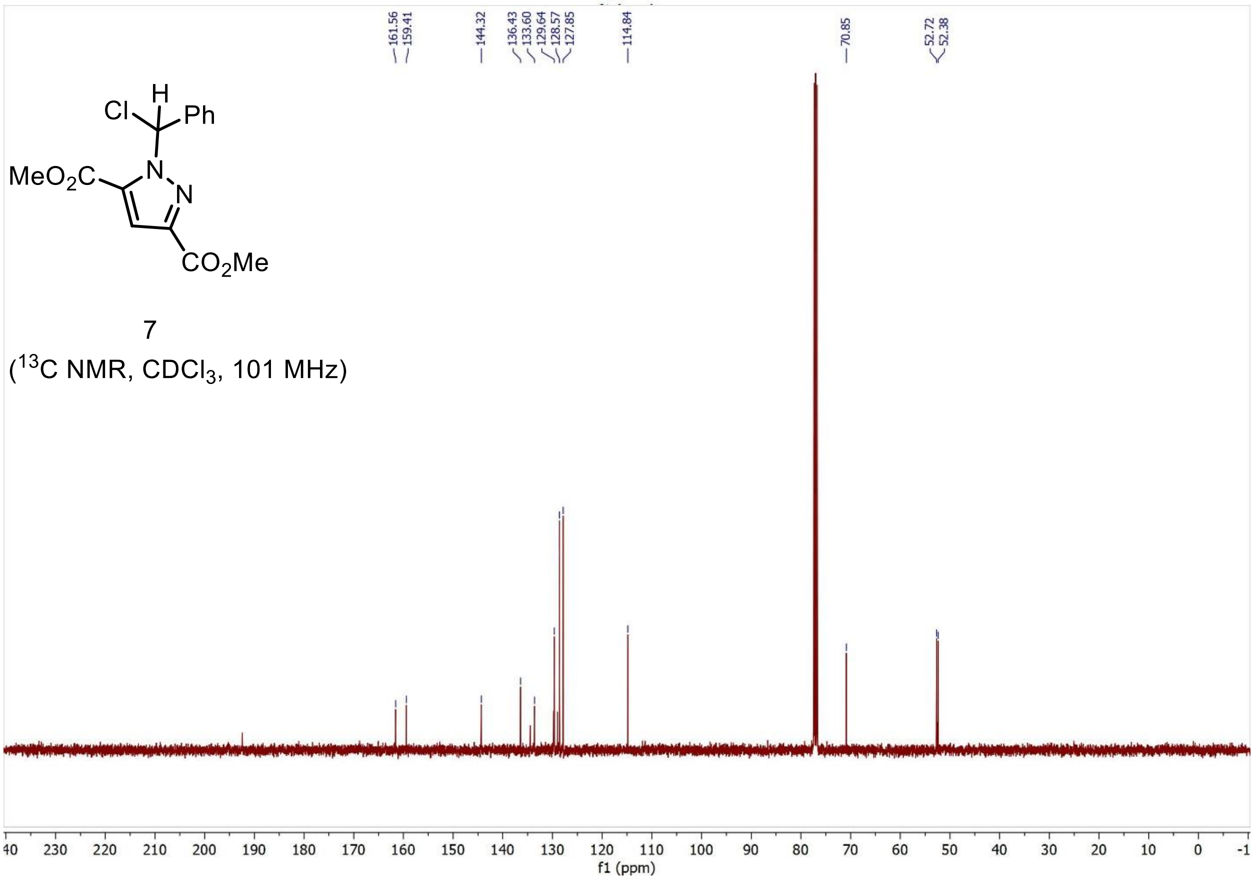
161.56
159.41
144.32
136.43
133.60
129.64
128.57
127.85
114.84
70.85
52.72
52.38
Cl
H
Ph
MeO2C
N
N
CO2Me
7
(13C NMR, CDCl3, 101 MHz)
240 230 220 210 200 190 180 170 160 150 140 130 120 110 100 90 80 70 60 50 40 30 20 10 0 -10
f1 (ppm)

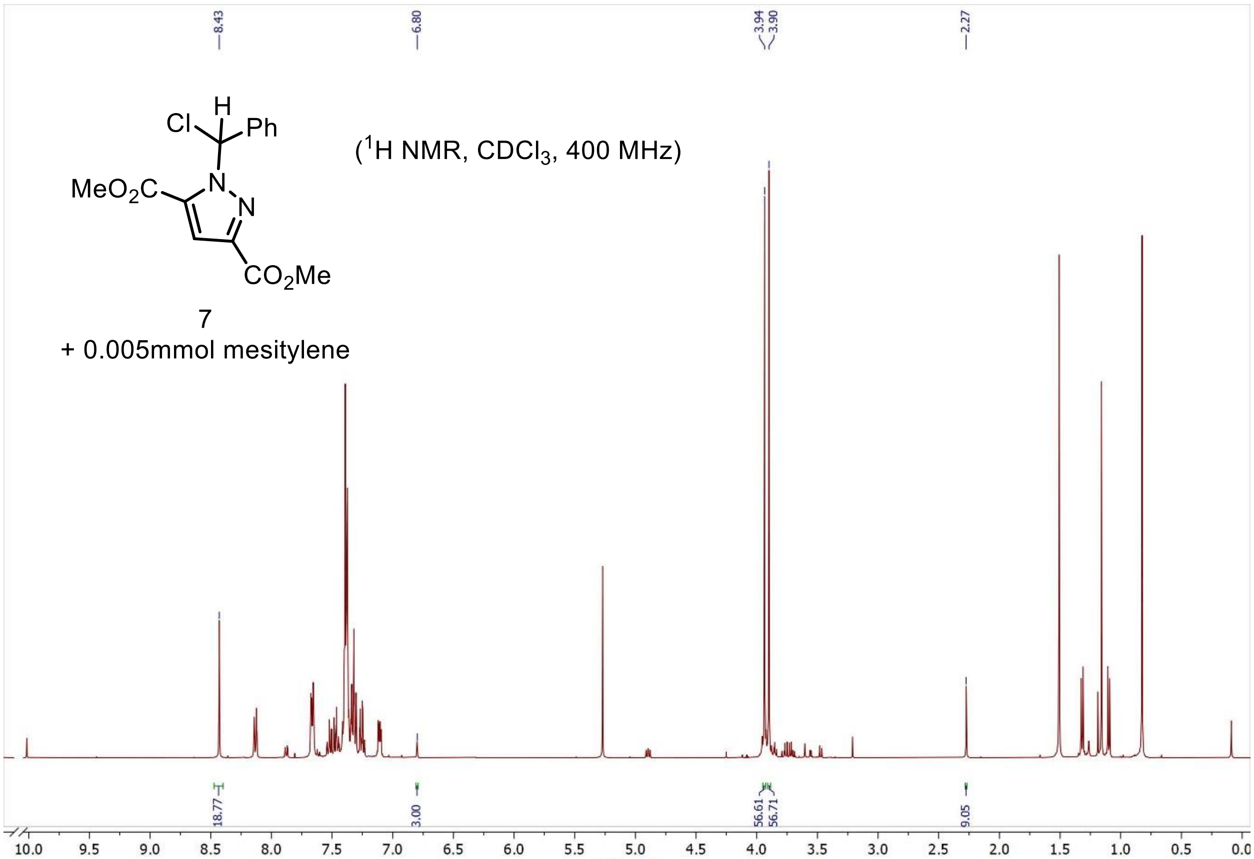
Cl
H
Ph
MeO2C
N
N
CO2Me
7
+ 0.005mmol mesitylene
(1H NMR, CDCl3, 400 MHz)

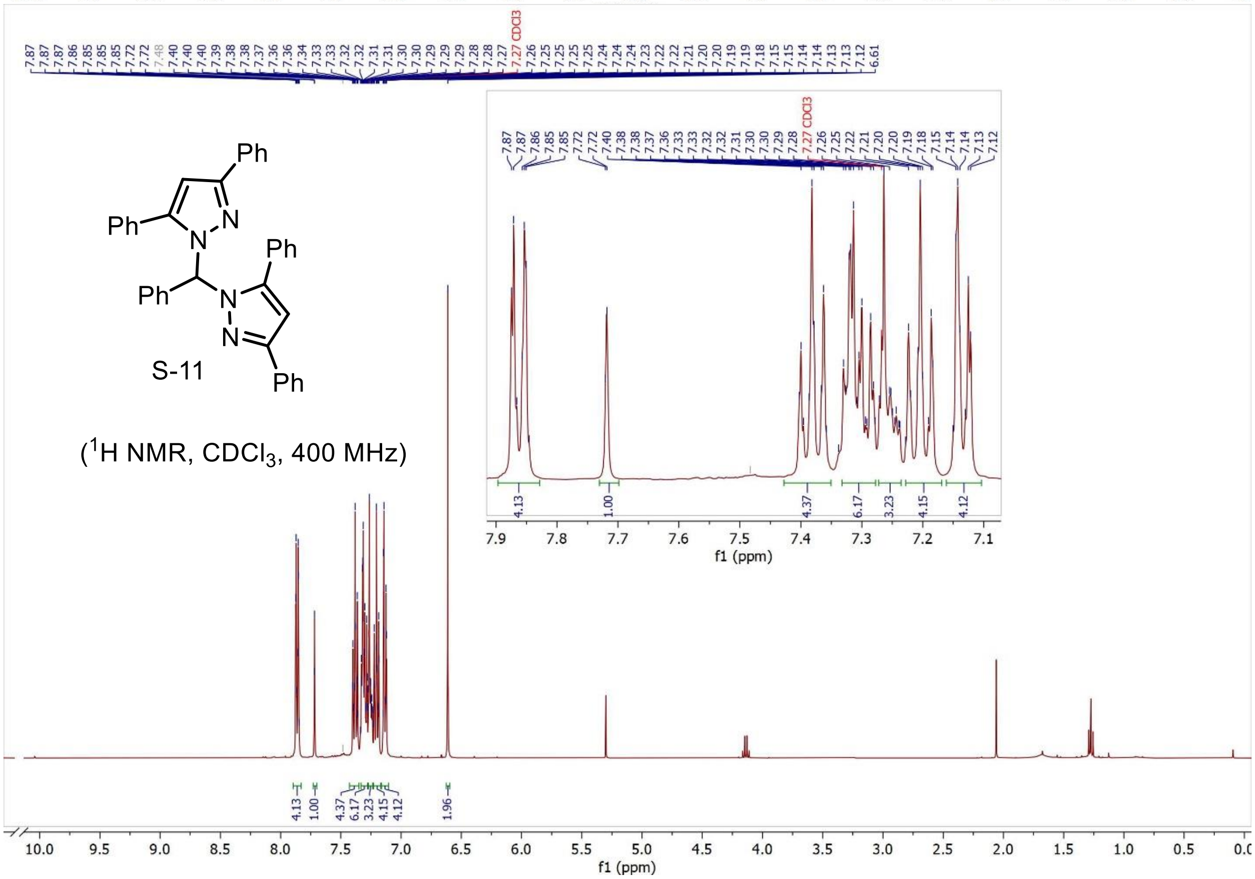
Ph
N
Ph
S-11
(1H NMR, CDCl3, 400 MHz)
f1 (ppm)

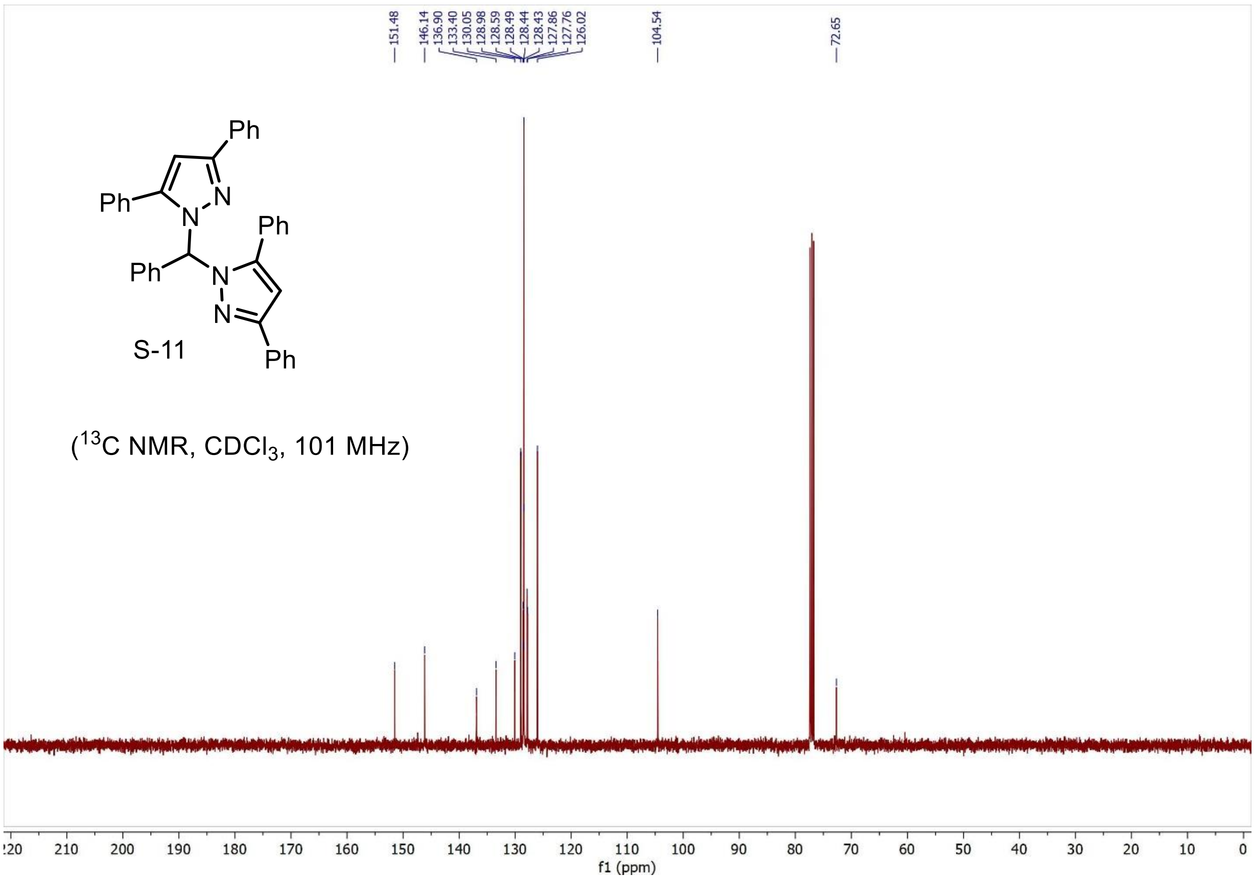
151.48
146.14
136.90
133.40
130.05
128.98
128.59
128.49
128.44
128.43
127.86
127.76
126.02
104.54
72.65
Ph
Ph
N
N
Ph
Ph
N
N
S-11
Ph
(^{13}C NMR, CDCl$_3$, 101 MHz)
220
210
200
190
180
170
160
150
140
130
120
110
100
90
80
70
60
50
40
30
20
10
0
f1 (ppm)
